# Golden Molecular Tweezers: Dinuclear Corannulene–Au(I) Acetylide Hosts for Fullerene Binding

**DOI:** 10.1021/acs.inorgchem.5c05787

**Published:** 2026-04-01

**Authors:** Nerea Álvarez-Llorente, Alberto Diez-Varga, Eric Masson, Héctor Barbero, Celedonio M. Álvarez

**Affiliations:** † GIR MIOMeT, IU CINQUIMA/Química Inorgánica, Facultad de Ciencias, 16782 Universidad de Valladolid, Valladolid E47011, Spain; ‡ Department of Chemistry and Biochemistry, 1354 Ohio University, Athens, Ohio 45701, United States

## Abstract

A series of homonuclear bimetallic organometallic complexes bearing two Au­(I) atoms and two corannulene fragments have been synthesized, and their fullerene recognition properties were extensively studied in solution. The tether groups were diphosphine ligands with variable rigidity (namely, dppe, dppf, dppbenz, and xantphos) to cover a range of potential semisupported intramolecular aurophilic interactions. Experimental results showed that neither extreme flexibility nor rigidity favors fullerene binding, and in those cases where the metallophilic contact is present and assured by ligand design, this force might hinder the supramolecular assembly formation. Computational calculations clearly indicate that the aurophilic interaction is just another force that is playing a moderate role within the manifold forces involved in the recognition process. The most notable finding corresponded to host **CAudppf**, which exhibited the highest experimental performance toward fullerene binding within the studied family due to its good preorganization in a tweezer-like conformation despite the lack of proper aurophilic contact. Thorough theoretical studies strongly suggested that the Au­(I)–Au­(I) distance can be shortened upon complexation, effectively turning on the metallophilic interactions.

## Graphical abstract

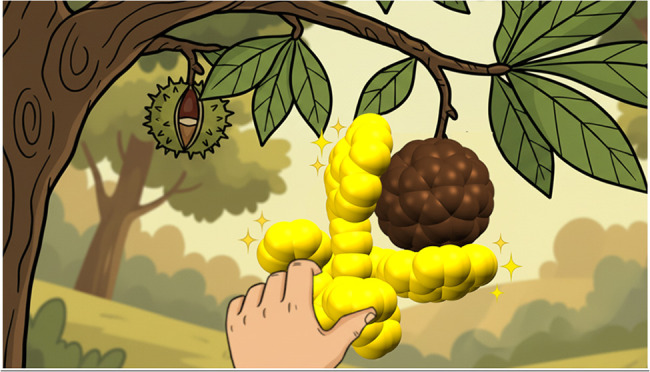

## Introduction

Fullerenes constitute a paradigmatic class of carbon nanostructures whose recognition by molecular hosts has attracted sustained attention due to their relevance in materials science, optoelectronics, and supramolecular chemistry.^1^ Among the different strategies developed for fullerene binding, concave–convex complementarity between host and guest has emerged as one of the most effective and conceptually elegant approaches. In this context, corannulene, a bowl-shaped polycyclic aromatic hydrocarbon, stands out as an exceptionally well-suited motif: its curved π-surface closely matches the convex topology of fullerenes, enabling strong dispersion-driven interactions and efficient surface contact. As a result, corannulene-based receptors have repeatedly demonstrated enhanced affinity and selectivity toward fullerenes compared to planar aromatic systems.^2^

Over the past years, we have systematically investigated corannulene-based supramolecular hosts for fullerene recognition, with a particular emphasis on understanding how host preorganization, rigidity, and cooperative effects influence binding strength.^3^ These studies have consistently shown that well-defined architectures, in which corannulene units are held in favorable relative orientations, significantly outperform more flexible or poorly organized systems. In addition to geometric complementarity, tether or group assistance—arising from secondary interactions or synergistic structural constraints—plays a decisive role by maximizing guest surface coverage and minimizing entropic penalties upon complexation. Such design principles have been validated across a wide range of molecular scaffolds and have also been corroborated by independent reports^2^ in the literature, establishing corannulene as a benchmark platform for fullerene host–guest chemistry.

Despite this extensive body of work, the incorporation of transition-metal centers into corannulene-based receptors has so far been primarily exploited as a means to impose specific coordination geometries (square-planar,^3a^ tetrahedral,^3e^ or octahedral)^3h^ or to lock the relative orientation of aromatic units. In these systems, the metal acts essentially as a structural element, and the potential influence of metal–metal interactions on supramolecular recognition processes has not been explicitly addressed. Aurophilic interactions—attractive contacts between closed-shell Au­(I) centers—represent a distinctive feature of gold chemistry and have been extensively studied both experimentally and theoretically.^4^ These interactions, typically observed at Au···Au distances shorter than the sum of van der Waals radii,^5^ arise from a subtle interplay of relativistic effects,^6^ orbital mixing, dispersion, and electron correlation.^7^ While aurophilic contacts are most commonly identified in the solid state, multinuclear Au­(I) complexes can also sustain intramolecular interactions in solution, depending critically on the nature, rigidity, and topology of the bridging ligands.^8^ Consequently, aurophilicity is best regarded as a structure-dependent phenomenon rather than an intrinsic or universally operative bonding motif.

In the present work, we merge these two research lines by examining homodinuclear Au­(I) acetylide complexes bearing corannulene units and bridged by diphosphine ligands of varying flexibility. Rather than treating aurophilic interactions as the primary object of investigation, we consider them as a potential structural contributor to host preorganization. If the bridging ligand is highly flexible and the energetic cost of deformation outweighs any stabilizing metal–metal interaction, the system remains poorly preorganized and no intramolecular Au­(I)–Au­(I) contact is expected (Figure 1a), resulting in suboptimal fullerene binding. Conversely, when the bridging ligand is sufficiently rigid to promote a moderately preorganized architecture (likely promoting intramolecular aurophilic contacts) such interactions could potentially enhance fullerene recognition (Figure 1b). It is also possible, however, that such a packed conformation hinders the inclusion complex formation, depending on how the spatial arrangement of the corannulene units are affected.

**Figure 1:**
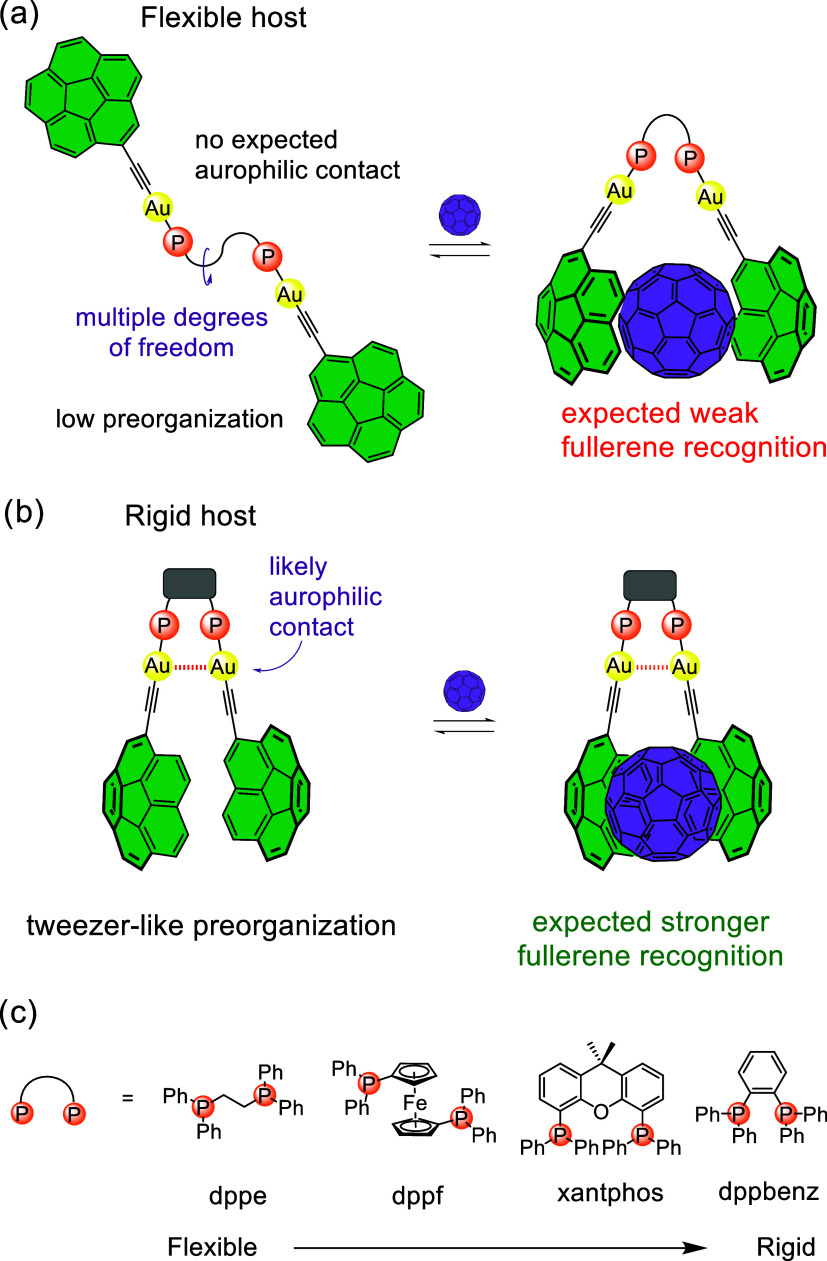
Schematic representation of homodinuclear Au­(I) acetylide complexes with fullerene recognition capabilities presented in this work. (a) A remarkably flexible phosphine ligand with high conformational penalty to establish a tweezer-like conformation. (b) A notably rigid phosphine ligand with excellent preorganization for fullerene recognition and Au–Au interaction prior to supramolecular binding. (c) Diphosphine bridging ligands employed in this study.

In this work we systematically vary the diphosphine backbone to adjust flexibility (dppe being the diphosphine ligand with the highest number of degrees of freedom, and dppbenz with the lowest, Figure 1c). Their recognition properties are compared with appropriate mononuclear (fragment AuPPh_3_, lacking a tweezer-like arrangement) and pyrene-based (planar aromatic groups with no concave-convex complementarity with fullerenes) reference systems. This study aims to clarify how metal-centered structural features interplay with concave–convex complementarity and host preorganization in governing fullerene binding behavior.

## Results and Discussion

### Synthesis of Target Complexes

The synthetic design relies on a straightforward protocol in which the diphosphine ligand is first attached to a chlorinated metal center prior to halogen substitution by the corresponding acetylide (Scheme 1). Thus, HAuCl_4_ was reduced in ethanol with tetrahydrothiophene to yield [AuCl­(tht)] (Scheme 1a).^9^ This common precursor was then utilized in two synthetic pathways. In the first route, chloro­(triphenylphosphine)­gold­(I) (**ClAuPPh**_**3**_) was prepared (Scheme 1b) by the simple addition of PPh_3_,^10^ followed by chlorido substitution with the corresponding polycyclic aromatic acetylide to afford the monogold complexes **PAuPPh**_**3**_ and **CAuPPh**_**3**_, obtained in 84% and 66% yields, respectively (Scheme 1d). In the second route, different bis-chloro­(organophosphine)­gold­(I) compounds (namely, **ClAudppe**, **ClAudppf**, **ClAudppbenz**, and **ClAuxantphos**) were prepared (Scheme 1c). This pathway was further divided into two approaches: for pyrene derivatives, in situ deprotection of trimethylsilyl acetylene using tetrabutylammonium fluoride (TBAF) easily afforded complexes **PAudppe**, **PAudppf**, **PAudppbenz**, and **PAuxantphos** in good yields (87% average yield, see the Experimental Section for more details) (Scheme 1e). However, this method was revealed to be ineffective for corannulene derivatives. Instead, the terminal acetylene precursor, obtained by desilylation of the TMS-alkyne, was employed with sodium methoxide as a base, furnishing the final compounds **CAudppe**, **CAudppf**, **CAudppbenz**, and **CAuxantphos**, with comparable efficiency (86% average yield, see the Experimental Section for more details) (Scheme 1f).

**Scheme 1:**
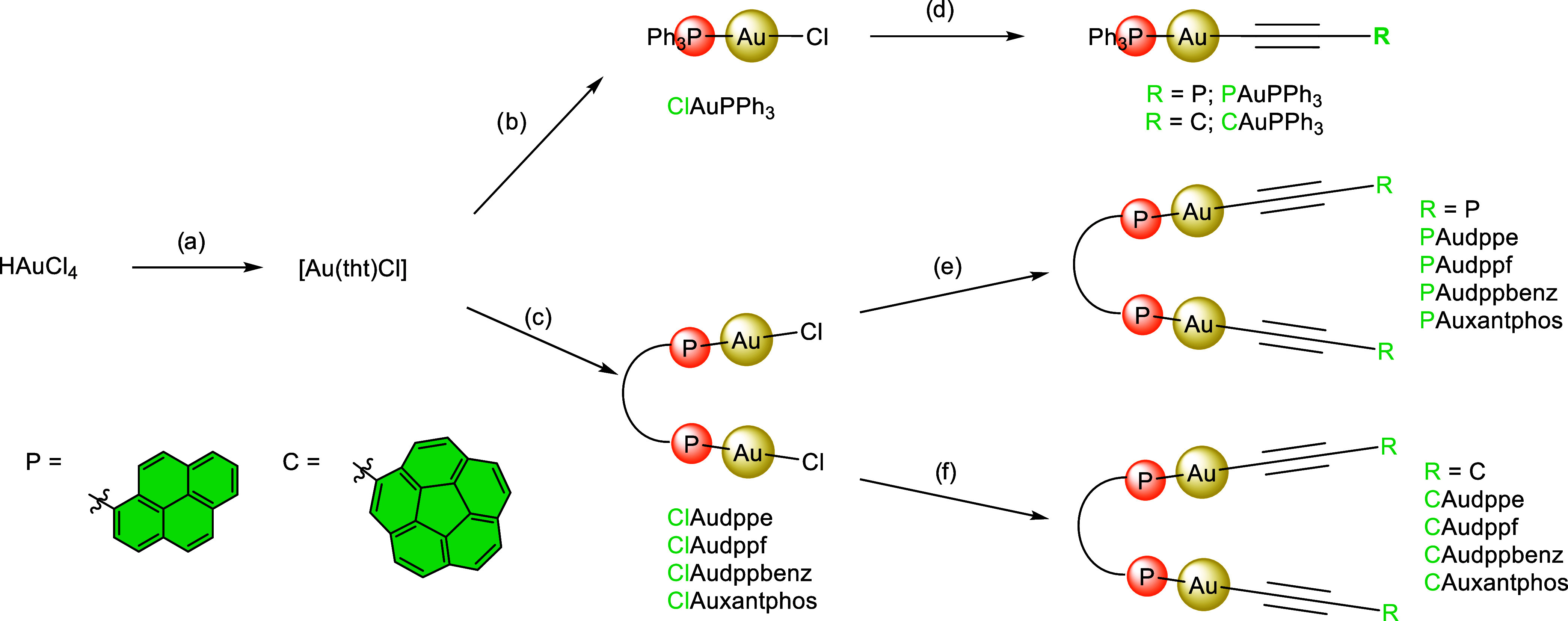
Preparation of the Whole Family of Gold­(I) Phosphine Complexes Reported in this Work^a^

a Reagents and conditions: (a) tetrahydrothiophene, EtOH, r.t. (b) 1.0 equiv triphenylphosphine, CH_2_Cl_2_, r.t. (c) 0.5 equiv diphosphine ligand, CH_2_Cl_2_, r.t. (d) 1.1 equiv (trimethylsilyl)­ethynyl-PAH, TBAF, EtOH, reflux. (e) 2.1 equiv 1-(trimethylsilyl)­ethynylpyrene, TBAF, EtOH, reflux. (f) 2.1 equiv 1-ethynylcorannulene, NaOMe, CH_2_Cl_2_/MeOH, 45 °C.

### Characterization of Gold­(I) Complexes

All complexes were fully characterized in solution by spectroscopic methods, as well as by mass spectrometry. Additionally, pyrene complexes **PAuPPh**_**3**_ and **PAudppf** were characterized in the solid state by X-ray diffraction.

The formation of the compounds was confirmed by ^31^P NMR spectroscopy. All spectra exhibited a single signal between 32.0 and 42.2 ppm, shifted downfield relative to the parent complexes, which resonate between 23.6 and 33.2 ppm (see the Experimental Section for more details). In the ^1^H NMR spectra, aromatic protons appeared over a broad spectral window between 8.4 and 6.4 ppm (Figure 2). Corannulene complexes displayed characteristic signals corresponding to both the polycyclic aromatic hydrocarbon and the organophosphine ligands. Pyrene complexes exhibited similar spectral features (see Supporting Information for more details).

**Figure 2:**
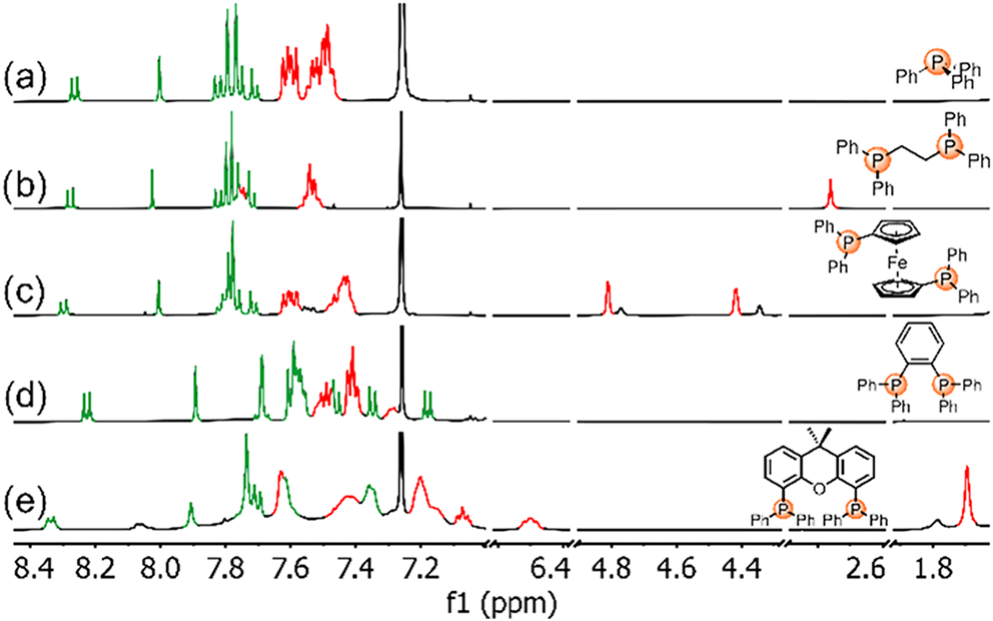
^1^H NMR (298 K, 500 MHz, CDCl_3_) spectra of complexes (a) **CAuPPh**_**3**_, (b) **CAudppe**, (c) **CAudppf**, (d) **CAudppbenz**, and (e) **CAuxantphos**. Peaks corresponding to the organophosphine and corannulene protons are colored in red and green, respectively.

Complexes **CAuPPh**_**3**_, **CAudppe**, and **CAudppf** showed sharp signals (Figure 2a–c), whereas **CAudppbenz** and **CAuxantphos** exhibited broadened signals (Figure 2d,e), especially in the latter, as a consequence of extraordinary steric hindrance and lack of mobility of the polycyclic aromatic ends. During the analysis of the ^1^H NMR spectrum of compound **CAudppf**, a notable feature was observed: Two distinct sets of cyclopentadienyl signals were present (Figure 2c), with no additional peaks indicating the presence of impurities. To investigate this phenomenon, a 2D NMR ^1^H–^1^H EXSY experiment was performed (Figure S63), revealing chemical exchange between the two pairs of cyclopentadienyl signals. This finding might suggest that the resonances arise from two conformations in this system (*syn* and *anti*). Variable temperature (VT) NMR experiments were conducted in CDCl_3_ and toluene-*d*_8_ (Figures S86 and S87). The small singlets of the minor component broadened below 25 °C reaching extinction at −25 °C in deuterated chloroform, whereas only one set of singlets was observed throughout the entire temperature range tested in toluene-*d*_8_ (25 °C–85 °C). This could indicate that the conformational exchange process (*syn* → *anti*) is feasible and occurs through cyclopentadienyl rotations along the axis containing both Cp centroids and Fe. Other potential exchanges (e.g., via CCPAu dihedral angle torsion) are apparently disfavored due to higher energy barriers (44 kcal/mol as estimated by computational methods, Figures S153 and S154).

Single crystals of pyrene complex **PAudppf** could be obtained, permitting its characterization by X-ray diffraction. Surprisingly, this compound crystallizes in a preferred *syn* conformation (Figure 3b,c), in contrast to previously reported Au­(I) complexes with dppf ligands and PAH substituents, which typically adopt an *anti* conformation.^11^

**Figure 3:**
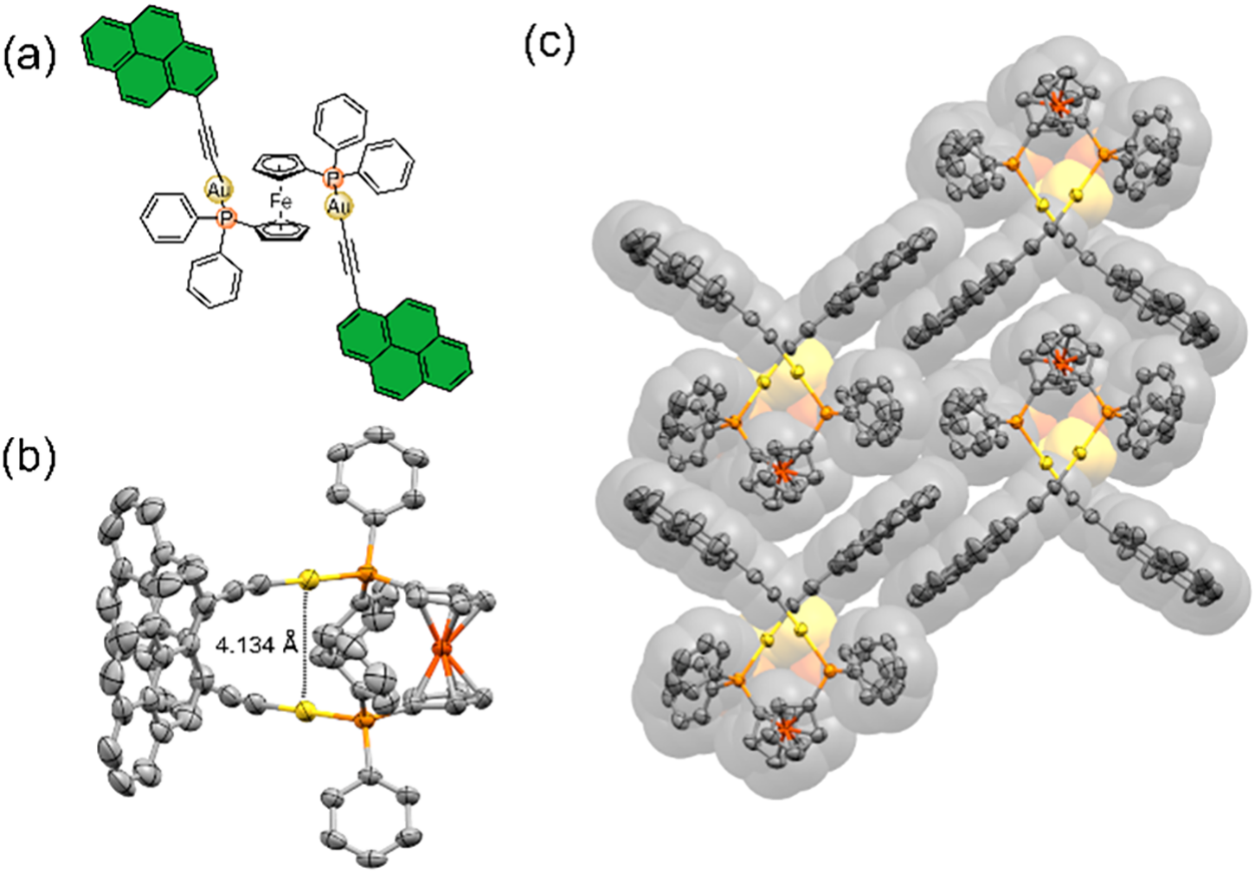
(a) Structure of complex **PAudppf**. (b) Solid state structure of **PAudppf**. (c) Representation of the crystal lattice showing the π-π stacking of the pyrene units.

The intramolecular Au­(I)–Au­(I) distance of 4.134 Å exceeds the threshold for aurophilic interactions^5b,8a^ clearly indicating that this force is not responsible for this particular arrangement. Analysis of the packing motif revealed large areas of π–π interactions between the pyrene units of adjacent molecules, forming columnar assemblies (Figure 3c). If this feature is explained by means of packing forces it could also be manifested in nondiluted solutions, where some degree of aggregation might occur. Moreover, it can be assumed that compound **CAudppf** exhibits the same property due to enhanced concave-convex complementarity between corannulene surfaces.^12^ These dispersion-based forces seem predominant and stronger than the ones exerted in a potential *anti* conformation, therefore providing the unexpected *syn* conformer without the need of favoring aurophilic contact. Another distinctive feature was observed in complex **CAuxantphos**, whose ^1^H NMR spectrum exhibited an additional broad signal at 8.06 ppm (Figure 2e), which was in chemical exchange with the doublet at 8.34 ppm, as revealed by ^1^H–^1^H EXSY experiment (Figure S76). Variable temperature (VT) ^1^H NMR experiments in CDCl_3_ revealed an intriguing behavior (Figure 4a,b). At low temperatures, the most deshielded proton at 8.4 ppm sharpened within the range between 15 °C and −10 °C, then broadened again before coalescing around −40 °C. Below this temperature, two broad signals emerged. A similar trend was observed for the corannulene singlet at 7.9 ppm, which coalesced at −45 °C, yielding two distinct singlets at lower temperatures. The energy barrier (Δ*G* ^‡^) for this process was estimated to be 11.2 kcal/mol, a relatively low value typically overcome at room temperature. This dynamic process likely corresponds to the rotation of the corannulene moiety along the C≡C–C axis (Figure 4a), which is hindered at low temperatures due to steric congestion from the two bulky PAH substituents in proximity, strengthened by guaranteed aurophilic interactions associated with the geometry of the xantphos ligand.

**Figure 4:**
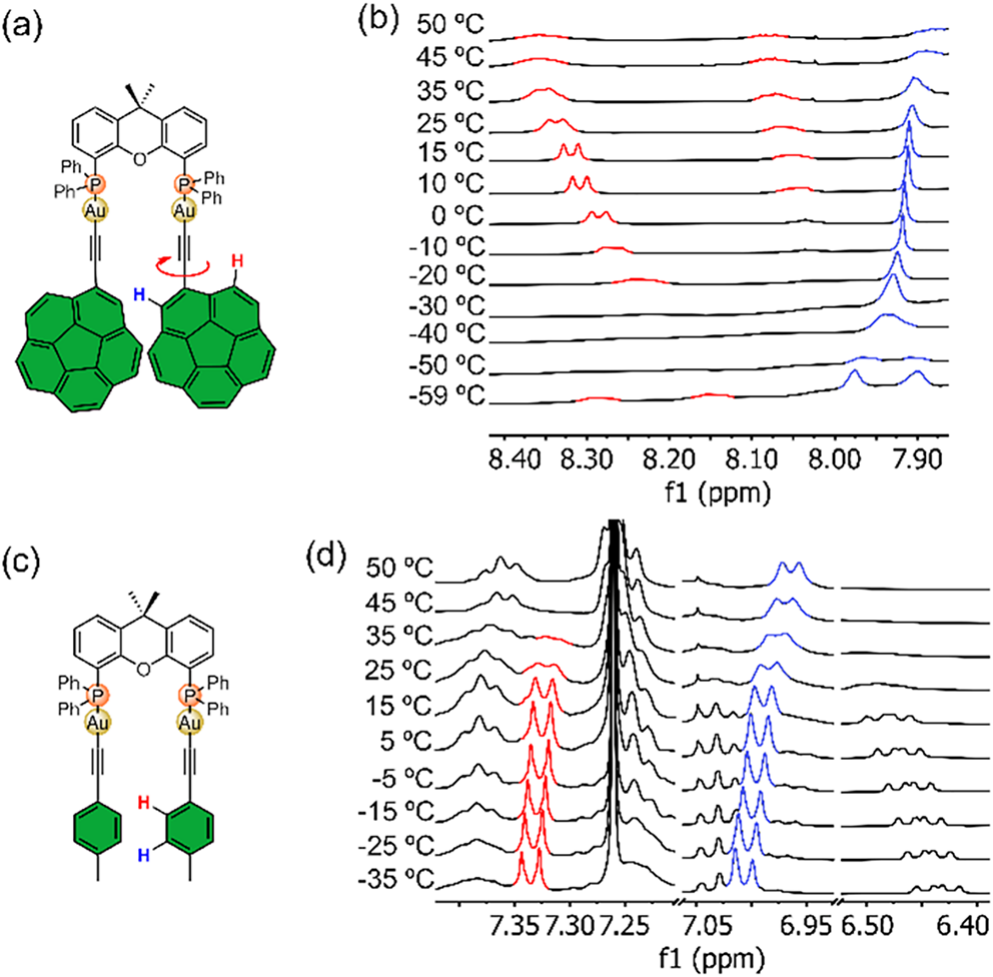
(a) Structure of CAuxantphos (the arrow indicates the rotation along the C≡C–C axis attributed to the low-temperature dynamic process). (b) ^1^H NMR VT experiments (500 MHz, CDCl_3_) of complex **CAuxantphos**. (c) Structure of complex ***p*** **-tolylAuxantphos**. (d) ^1^H NMR VT experiments (500 MHz, CDCl_3_) of complex ***p*** **-tolylAuxantphos**.

To verify this hypothesis, a complex with a smaller substituent, ***p*** **-tolylAuxantphos** (Figure 4c), was synthesized using *p*-tolylacetylene as a ligand, following the same procedure as for the dicorannulene complexes (see Scheme 1). VT ^1^H NMR experiments in CDCl_3_ (Figure 4d) showed no spectral changes upon cooling, supporting the conclusion that the dynamic behavior observed in **CAuxantphos** compound at low temperatures arises from the steric hindrance imposed to the rotation of the corannulene units. At temperatures above room temperature, the signals broaden further in both cases, but no coalescence was observed. This second process could be associated with ring puckering distortions in the xanthene moiety pertaining to the diphosphine ligand. A similar behavior has been reported for digold­(I) complexes bearing aromatic substituents and may be attributed to fluxionality or aggregation.^13^ Furthermore, single crystals suitable for X-ray diffraction were obtained for compound *p*-tolylAuxantphos, enabling its characterization in the solid state (Figure 5). The Au­(I)–Au­(I) distance of 3.027 Å confirms the presence of aurophilic interactions. Although the solid-state structures of PAH derivatives with the xantphos ligand could not be determined, it is reasonable to assume that they adopt a similar geometry in which the diphosphine ligand promotes aurophilic interactions.

**Figure 5:**
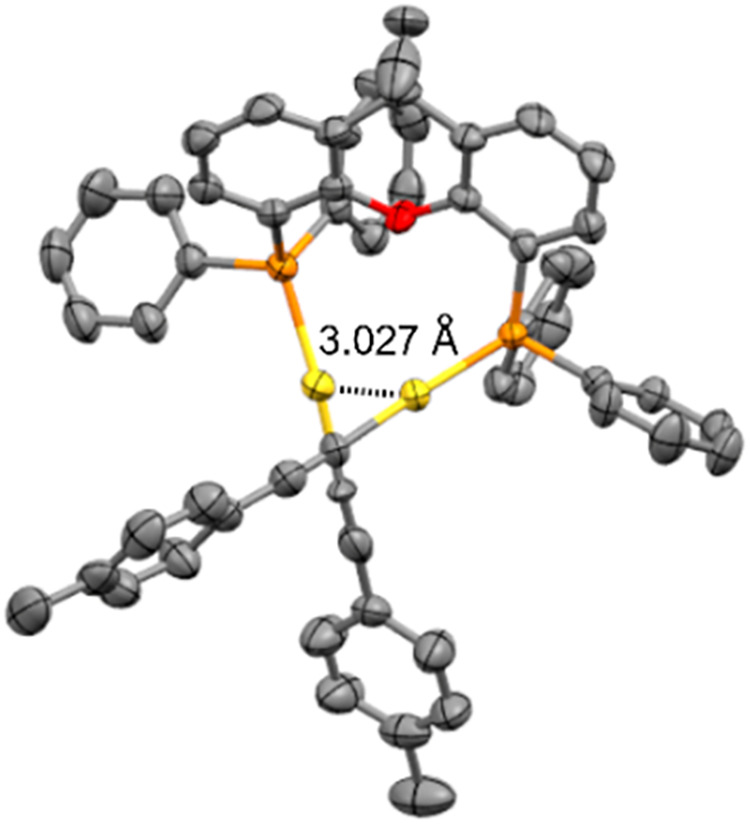
X-ray diffraction structure of complex ***p*** **-tolylAuxantphos**.

UV–vis absorption spectra of pyrene complexes in dichloromethane exhibited structured bands (Figures S101 and S103), whereas corannulene derivatives displayed broader bands (Figures S102 and S103). The absorption bands below 300 nm were attributed to ligand-centered π–π* transitions of the phosphine and acetylide ligands, in agreement with literature reports.^14^ Bands appearing between 350 and 400 nm are assigned to LMCT transitions from the ethynyl PAH ligands to the metal center. This excited state (S_1_) is common for aryl ligands with low ionization potential and high LUMO energy.^15^

The emission spectra of pyrene complexes (Figures S104 and S106) displayed structured bands in the range between 350 and 450 nm, whereas those of corannulene complexes appear again as broader bands within the same wavelength interval (Figures S105 and S106). These bands are normally ascribed to fluorescence from the singlet excited state. Additionally, low-intensity bands within the range of 550 to 750 nm are also observed for complexes **CAudppbenz** and **CAuxantphos**, (Figure S105), tentatively assigned to phosphorescence from the triplet excited state where the gold centers (heavy atoms with strong spin–orbit coupling) contribute through σ­(Au–P)–π*­(C≡C) transitions.^16^ Emission studies performed under deareated conditions produced moderate enhancement of described bands, suggesting certain involvement of gold (Figures S109 and S110).

Cyclic voltammetry (CV) and square-wave voltammetry (SWV) experiments in DMF were also performed (Figures S111, S112, and S113 and Table S1). Au­(I) complexes typically exhibit a single irreversible, two-electron oxidation corresponding to Au­(III)/Au­(I) transformation.^17^ Oxidative scans of pyrene derivatives revealed an irreversible oxidation at approximately 0.90 V (vs Fc/Fc^+^), assigned to mentioned process. However, no significant differences in redox potential were observed between the mononuclear complex **PAuPPh**_**3**_ and the dinuclear complexes. Interestingly, a maximum cathodic shift of 50 mV was observed for compound **PAuxantphos** suggesting potential aurophilic contact in solution^18^ as anticipated from the topology of the xantphos ligand. Unfortunately, these experiments did not allow us to unambiguously determine the presence of Au­(I)···Au­(I) interaction on complex **PAudppf** in solution, which emerged as the only uncertain example within the series investigated in this work, given that such an interaction was not found in the solid state. For corannulene derivatives, cyclic voltammograms did not show oxidation peaks within the electrochemical window, except for the Fe­(III)/Fe­(II) oxidation in complex **CAudppf**, which precluded further analysis. A computational estimation of the oxidation potential for such a transformation showed that corannulene derivatives would require an additional oxidative voltage of ca. 1.95 V with respect to the oxidation of the pyrene derivatives, clearly indicating that it would not be observable within the current electrochemical window (see the Supporting Information for more details). Attempts to use solvents with wider electrochemical windows, such as acetonitrile, were unsuccessful due to solubility limitations.

### Host–Guest Chemistry with Fullerenes

Given the structure of the synthesized compounds, featuring two corannulene units capable of acting as a tweezer for fullerenes, and considering previous literature reports, association constants in the range of 10^–3^ to 10^–5^ M^–1^ in toluene-*d*_8_ were anticipated.^1c^ This solvent was chosen for comparison purposes with previous studies. Additionally, it provides good fullerene solubility and ensures complete solubility of the host at the titration conditions (see Supporting Information for more details). Considering the expected association constant range, NMR spectroscopy was deemed an appropriate technique for their determination.^19^ Indeed, the stepwise addition of aliquots of C_60_ and C_70_ to the hosts induced changes in various chemical shifts of aromatic protons (Figure 6), indicating a fast exchange regime and confirming the supramolecular interaction. In contrast, pyrene derivatives exhibited no such spectral changes, highlighting the essential role of corannulene curvature in facilitating supramolecular binding. Regarding the stoichiometry of the adducts, the presence of a single binding cavity suggests a 1:1 host–guest complexation model, which was employed for nonlinear regression analysis.^19^ The identity of claimed inclusion complexes was further confirmed by low- and high-resolution mass spectrometry (see the Supporting Information). The results are summarized in Table 1. Surprisingly, the association constants obtained for these complexes are slightly lower than those reported for other metal-based hosts developed by our group, including Cu­(I)-based tweezers (1.2 × 10^3^ M^–1^ in CD_2_Cl_2_)^3e^ and Pt­(II)-based hosts (4.6 × 10^3^ M^–1^ in toluene-*d*_8_).^3a^ They also fall short compared to purely organic hosts, such as Chen’s helicene (2.8 × 10^3^ M^–1^ in toluene-*d*_8_),^20^ our series of organic tweezers (1.3 × 10^3^ M^–1^ to 4.7 × 10^3^ M^–1^ in toluene-*d*_8_)^1b,1c^ ^,d,f,g^ and Sygula’s buckycatchers (3.2 × 10^3^ M^–1^ in toluene-*d*_8_ to 1.0 × 10^4^ M^–1^ in chlorobenzene-*d*_5_).^2b,2d,2e,21^ However, these values are comparable to those recently reported for our ionic Ru­(II)-based multitopic hosts (3.5 × 10^2^ M^–1^ in toluene-*d*_8_).^3h^ The latter behavior can however be explained by the existence of partial inhibition due to strong ion pairing in the solvent.

**Figure 6:**
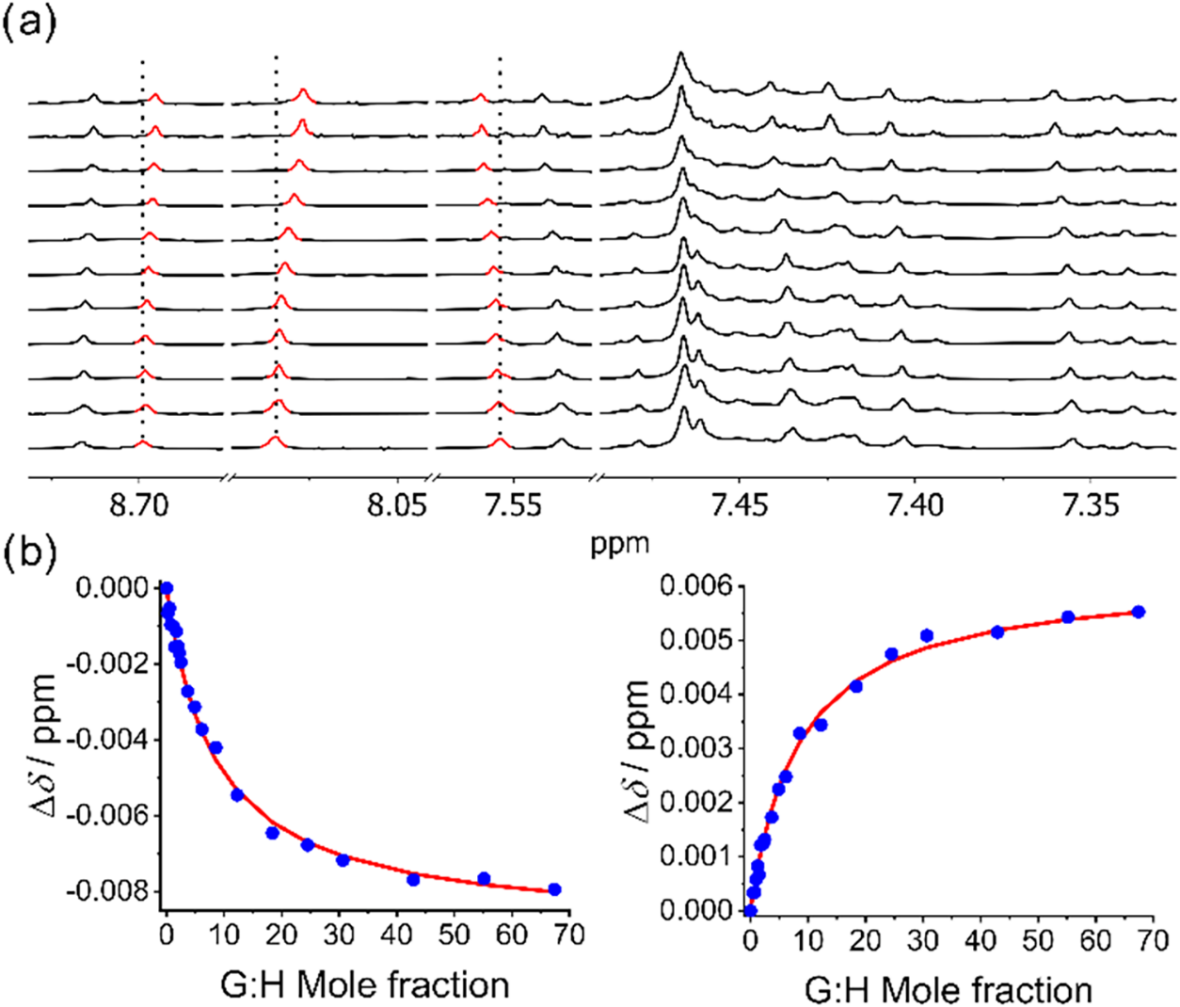
(a) Stacked ^1^H NMR (298 K, 500 MHz, toluene-*d*_8_) spectra of complex **CAudppf** with variable concentrations of C_70_. Most significant changes in the chemical shifts of aromatic signals have been highlighted in red. (b) Variation of chemical shifts (Δδ) for two different signals versus the guest mole fraction (blue points) along with the fitted binding isotherm obtained by nonlinear regression (red line).

**Table 1:** Association Constants Obtained for the Supramolecular Adducts between Prepared Hosts and Fullerenes after Nonlinear Regression Fittings to a 1:1 Stoichiometry Model

complex	*K*_a_ vs C_60_ (M^–1^)	*K*_a_ vs C_70_ (M^–1^)
**CAudppe**	(6.85 ± 0.18) × 10^2^	(7.72 ± 0.31) × 10^2^
**CAudppf**	(9.64 ± 0.23) × 10^2^	(9.44 ± 0.19) × 10^2^
**CAudppbenz**	(2.89 ± 0.04) × 10^2^	(5.93 ± 0.05) × 10^2^
**CAuxantphos**	(1.18 ± 0.01) × 10^2^	(8.23 ± 0.17) × 10^2^

The first and most remarkable finding is the fact that there is not an immediate trend in fullerene affinity based on ligand rigidity, as hypothesized in the introduction section (Figure 1). Association constants for host **CAudppe** are moderately low, as expected by the flexibility of the diphosphine bridging ligand (therefore, a high deformation energy penalty is paid) and the lack of aurophilic interactions capable of fixing a tweezer-like geometry. Interestingly, the affinity is then raised by host **CAudppf** whose *syn* geometry, at least in the solid state, allows a suitable preorganization for fullerene binding with no preference for C_60_ or C_70_. A more striking observation is the experimental association constants of hosts **CAudppbenz** and **CAuxantphos**, which were expected to exhibit enhanced performance due to the proximity of their corannulene moieties and ensured Au­(I)···Au­(I) contacts by design, yet their affinity decreased, even below host **CAudppe**. This outcome may be attributed a priori to insufficient preorganization of their cavities owing to the excess of proximity between corannulene units, lack of flexibility for adaptation and facilitated dispersion-based intramolecular interactions. These results show that the strongest affinity for C_60_ belongs to host **CAudppf**, with a binding constant approaching 10^3^ M^–1^. A similar trend was observed for C_70_ adducts, with binding constants slightly higher than those obtained for C_60_ in most cases, as the hosts can adapt more efficiently to the ellipsoidal shape of C_70_.^2b,2d,2e,3a,2b,2c,2e–2g^ Notably, **CAuxantphos** exhibited remarkable selectivity for C_70_, displaying a *K*_C70_/*K*_C60_ ratio of ca. 7. Overall, these findings indicate no significant differences in fullerene binding across the various hosts based on the presence or absence of aurophilic interactions, suggesting that these contacts have a minimal impact on the recognition process. In fact, hosts exhibiting favorable metallophilic interactions were among the weakest fullerene receptors, highlighting that aurophilic contacts do not necessarily enhance host–guest affinity in these systems.

An unexpected observation arose from the analysis of the mixture of complex **CAuPPh**_**3**_ with C_70_, as progressive changes in the chemical shifts of certain host signals were detected upon incremental addition of C_70_ (Figure S118). This behavior was not observed for C_60_, as the host’s signals remained unchanged (Figure S117). The use of this compound, as stated above, was deemed solely for comparison purposes as (1) it bears only one unit of corannulene, and (2) it lacks intramolecular aurophilic interactions. Therefore, its capability as a host for fullerene recognition is expected to be negligible and only plausible upon aggregation in concentrated solutions. Moreover, only a few monocorannulene derivatives, with extended π surfaces, have demonstrated measurable interactions with fullerenes.^2a,2i,22^ Thus, a blank experiment was carried out and confirmed that the observed chemical shift variations were not attributable to dilution effects (Figure S121). Nonlinear regression analysis of the NMR titration data, based on a 1:1 binding model, yielded an association constant of (4.4 ± 0.1) × 10^2^ M^–1^ in toluene-*d*_8_. This is the first example of a nonextended monocorannulene host to show recognition toward C_70_ that is measurable in solution. However, the origin of this affinity might come from subsequent favorable interactions with a second host molecule. To further investigate this binding mode, a 2:1 stoichiometry model was considered and analyzed using four different fitting approaches (flavors).^19,23^ Although the association constants (*K*_a_) values were of the same order of magnitude across all cases, the covariance factors (cov_fit_ < 3) indicated no statistically significant preference for the 2:1 model (Table S3). Nonetheless, computed geometries for this ternary model showed that the arrangement is plausible (Figure S156) and both stoichiometries (1:1 and 2:1) have been detected by mass spectrometry (Figure S120).

Several experiments (CV and VT-NMR) were carried out to experimentally detect potential new intramolecular aurophilic interactions in the supramolecular adducts in solution, but those were inconclusive.

### Computational Studies

To gain further insight into the titration results described above, density functional theory (DFT) calculations were performed to optimize the structures of the hosts and their corresponding supramolecular adducts. The geometry optimization of complex **PAudppf** was carried out using various computational methods, and the resulting geometries were compared to the available crystal structure (see Supporting Information for more details). Among the tested methods, PBE0-D3BJ/LANL2DZ//Def2TZVP/PCM (toluene)^24^ provided the best agreement with the experimental data and was therefore selected for the optimization of the entire family of corannulene-based tweezers and their corresponding supramolecular adducts. The optimized structures of the host–guest assemblies revealed that all hosts are capable of adopting a suitable conformation to accommodate a fullerene molecule in a tweezer-like fashion, with the concave face of the corannulene substituents covering a significant portion of the guest surface (Figure 7), except for the case of C_60_@**CAudppbenz** adduct, whose corannulene units are arranged in a bent conformation (Figure 7c). These structural arrangements are expected to maximize dispersion interaction, thereby contributing to the overall stability of the supramolecular adducts.

**Figure 7:**
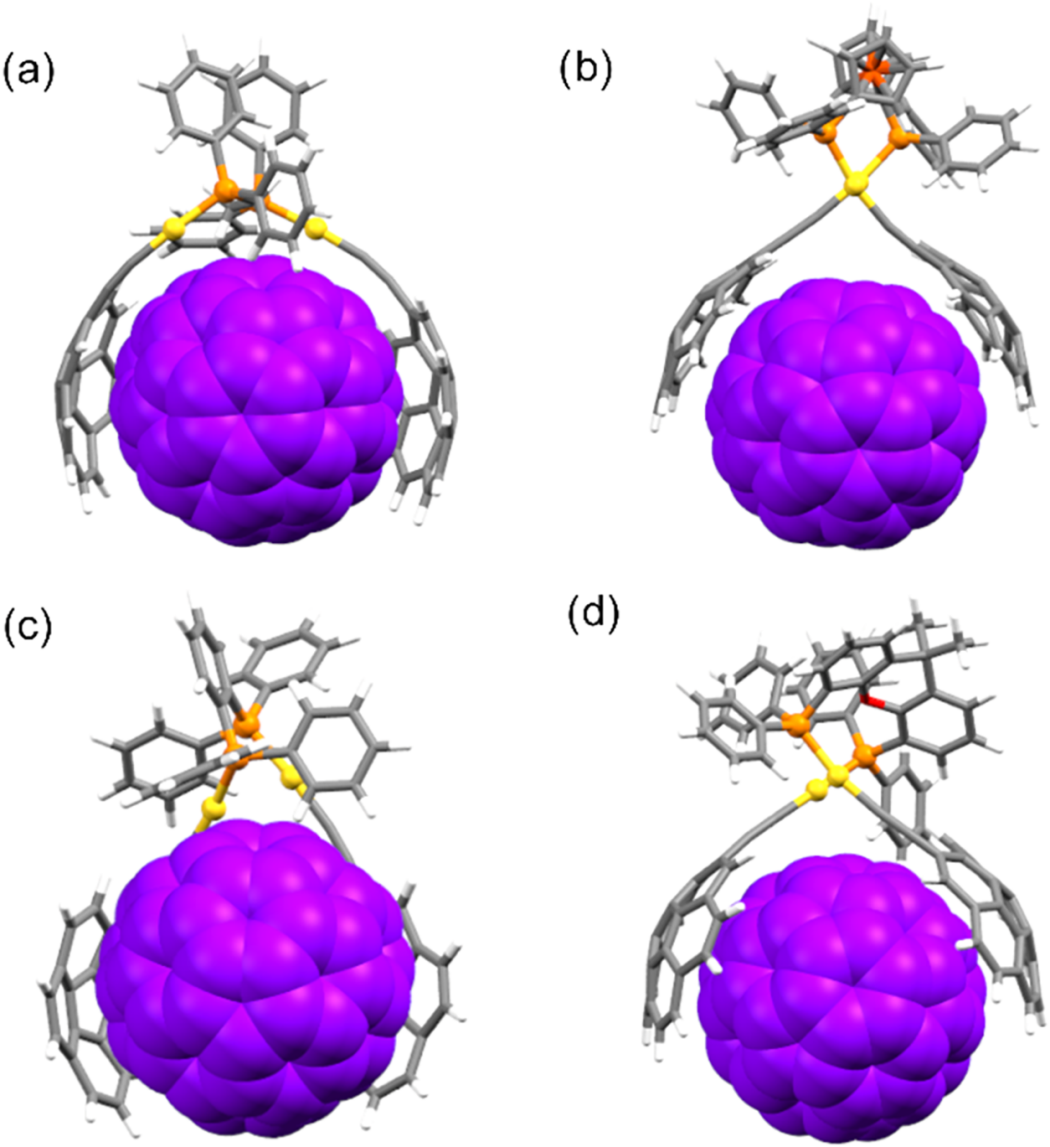
PBE0-D3BJ/LANL2DZ//Def2TZVP/PCM-(toluene) optimized structures of the supramolecular adducts formed between C_60_ and (a) **CAudppe**, (b) **CAudppf**, (c) **CAudppbenz**, and (d) **CAuxantphos**.

Regarding the Au­(I)–Au­(I) distances of the optimized supramolecular assemblies (Table 2), the most remarkable finding is the shortening of the Au­(I)–Au­(I) distance in the C_60_@**CAudppf** assembly (3.31 Å vs 4.13 Å in the solid-state structure) possibly suggesting a potential aurophilic contact turn-on upon supramolecular recognition. Despite the fact that this effect could not be indirectly observed by experimentation, it is a notable result considering that the Au­(I)–Au­(I) distance in the C_60_@**CAuxantphos** assembly almost negligibly changes from 3.03 Å in the crystal structure to 3.10 Å in the computed adduct. Intramolecular gold interaction energies were calculated according to the empirical eq 1.^8d,16a,25^

1 $$E_{\mathrm{Au-Au}}=1.27\times 10^{6}e^{-3.5d\left(\mathrm{Au-Au}\right)}$$

**Table 2:** Intramolecular Au­(I)–Au­(I) Distances in Computed C_60_ Adducts of Reported Hosts in this Work^a^

assembly	*d*(Au–Au) (Å)	*E*_Au–Au_ (kcal·mol^–1^)
**C**_**60**_ **@CAudppe**	6.50	0
**C**_**60**_ **@CAudppf**	3.31	–2.9
**C**_**60**_ **@CAudppbenz**	3.00	–6.0
**C**_**60**_ **@CAuxantphos**	3.10	–8.3

a Empirical aurophilic interaction energy based on eq 1 and computed distances.

As expected, the most stabilizing energies belong to **CAudppbenz**- and **CAuxantphos**-based complexes owing to these diphosphine ligands structures which promote intramolecular gold contacts. It is worth noticing that this effect is maintained in the supramolecular adduct geometries. The interaction energy of the C_60_@**CAudppf** assembly is moderate, consistent with the Au­(I)–Au­(I) distance shortening observed upon adduct formation, whereas it is near zero for C_60_@**CAudppe** assembly (Table 2), likely due to a poorly preorganized host structure. This means that a potentially attractive aurophilic interaction is rather low to compensate for the deformation energy penalty (see below).

Noncovalent interaction (NCI) plots^26^ further corroborate these findings, revealing extended weak (van der Waals) contacts between the inner surface of the corannulene units and the outer surface of the fullerene (Figure 8). Additionally, NCI plots for C_60_@**CAudppe** and C_60_@**CAudppbenz** adducts identified weak interactions between the guest and the C≡C–Au–phosphine moiety of the tweezer, suggesting an additional contribution for the stability of the adducts. More importantly, a critical point, based on AIM theory^27^ (see Supporting Information for more details), between gold atoms was found for all assemblies except for that of C_60_@**CAudppe** (Figure 8).

**Figure 8:**
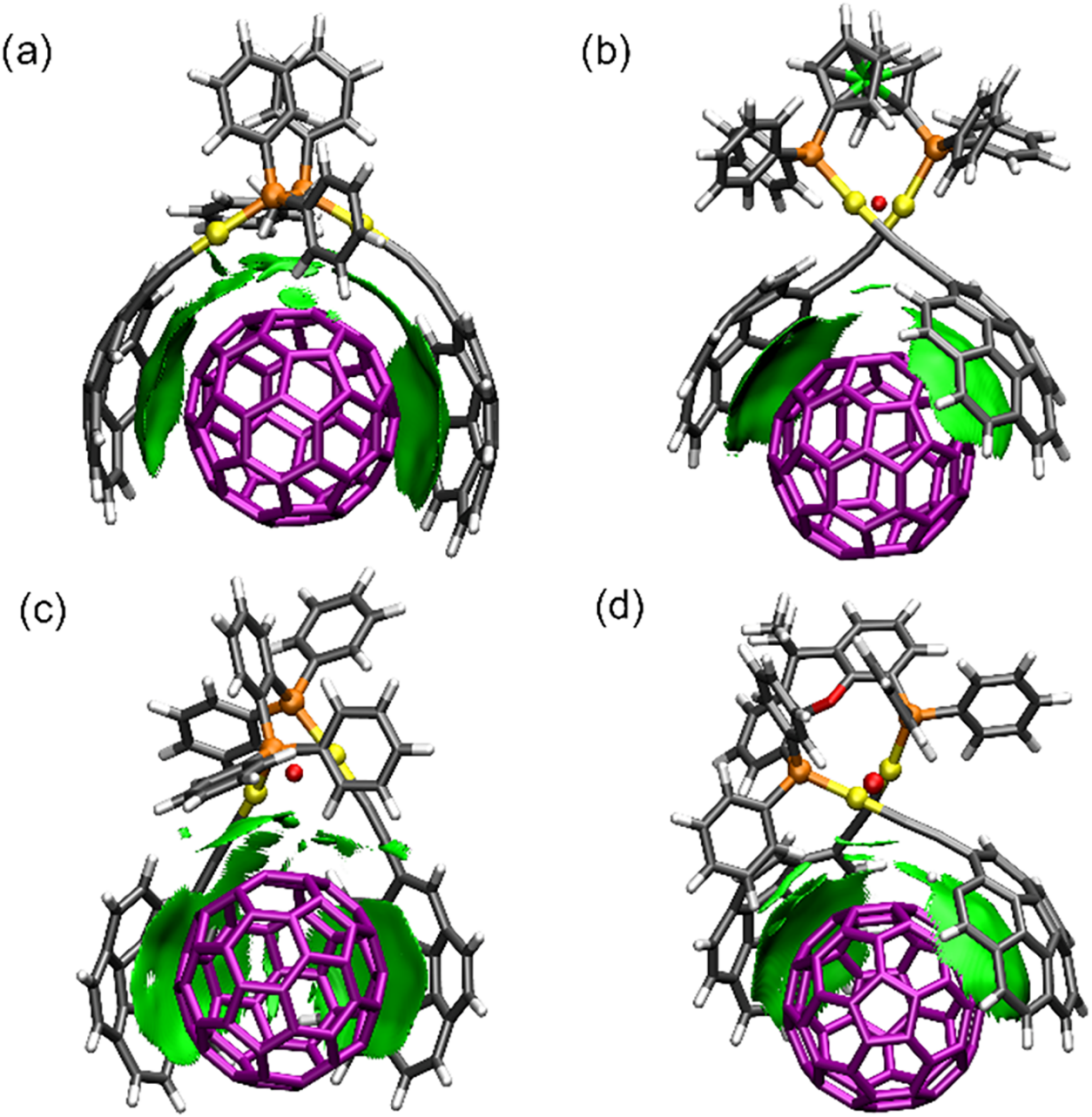
Gradient isosurfaces (isovalue = 0.3 au) and AIM critical points (in red) between two Au atoms for (a) C_60_@**CAudppe** and (b) C_60_@**CAudppf**, (c) C_60_@**CAudppbenz**, and (d) C_60_@**CAuxantphos** assemblies. Densities within the interval −0.02 < *r* < 0 are exclusively shown because they represent vdW interactions. Green color indicates weak attraction.

Interaction energies between the different hosts and C_60_ were determined using the Counterpoise method^28^ (see the Supporting Information for more details). All Counterpoise-corrected interaction energies fall within the expected range for singly functionalized corannulene hosts (typically −17.03 kcal/mol per corannulene unit).^29^ A rather higher interaction energy was observed for the **CAudppe** and **CAudppbenz** complexes. This can be attributed to additional contributions from interactions involving the noncorannulene regions of the host molecules, as evidenced by the NCI plots (Table 3).

**Table 3:** Summary of Counterpoise-Corrected Interaction Energies (*E*_int_) and Deformation Energies (*E*_def_) for the Assemblies Formed between the Hosts and Fullerene C_60_

host	*E*_int_ (kcal mol^–1^)	*E*_def_ (kcal mol^–1^)
**CAudppe**	–45.4	10.4
**CAudppf**	–33.9	7.2
**CAudppbenz**	–42.1	10.0
**CAuxantphos**	–38.3	14.0

Given that the electronic interaction energy is not solely responsible for the observed experimental behavior, we also carried out calculations of electronic deformation energies, according to eq 2, to account for the energy penalty that every host has to pay in order to establish the expected tweezer-like arrangement for fullerene recognition. This factor is especially important^2b,3g^ in flexible hosts such as C_60_@**CAudppe**, for instance.

2 $$E_{\mathrm{def}}=E_{H}\left(\mathrm{HG}\right)-E_{H}\left(H\right)$$

*H* and *G* stand for host and guest, respectively; subscript denotes treated fragment (host in all cases), whereas the optimized geometry whose fragments are used is given in parentheses (see the Supporting Information for more details).

As expected from the experimental and computational data, host **CAudppf** exhibited the lowest value, supporting our earlier statement that it possesses the most preorganized cavity. Interestingly, hosts **CAudppbenz** and **CAuxantphos** show deformation energies similar to that of complex **CAudppe**, indicating that the energy penalty paid by a remarkable rigid host with favorable aurophilic interactions falls within the same range as the deformation penalty of a very flexible host. Two main conclusions can be extracted from all the findings so far: (1) preformed aurophilic interactions do not necessarily contribute to the formation of a tweezer-like structure as it might even exert an opposing force, (2) well-preorganized hosts are better receptors, regardless the existence of aurophilic contact. Nonetheless, C_60_@**CAudppbenz** assembly shows a different computed geometry when compared to the other members of the family, as pointed out above (Figure 7c). In order to appropriately compare all fullerene adducts, a tweezer-like structure was optimized at the same level of theory and treated according to already described formalism (see the Supporting Information for more details). The resulting assembly shows a lower electronic interaction energy (−29.8 kcal/mol) and a much higher deformation energy (27.5 kcal/mol). These values clearly indicate that such a host is incapable of establishing the desired tweezer-like conformation as the energy penalty paid by the host is comparable to the interaction energy with C_60_.

To gain further insight into the energetics involved in the recognition process, Energy Decomposition Analysis (EDA)^30^ was carried out at the same theory level. The decomposition scheme follows the terms described in eq 3.

3 $$E_{\mathrm{int}}=V_{\mathrm{elstat}}+E_{\mathrm{Pauli}}+E_{\mathrm{oi}}+E_{\mathrm{disp}}$$

Where *V*_elstat_ refers to the electrostatic classical-like Coulombic interactions, *E*_Pauli_ represents the Pauli exchange-type repulsions between filled orbitals, *E*_oi_ (orbital interactions or charge transfer) cover the attractive interactions between filled and unfilled orbitals of different fragments and *E*_disp_ corresponds to London-type weak interactions between polarizable electron clouds. Computed energies are represented in Figure 9.

**Figure 9:**
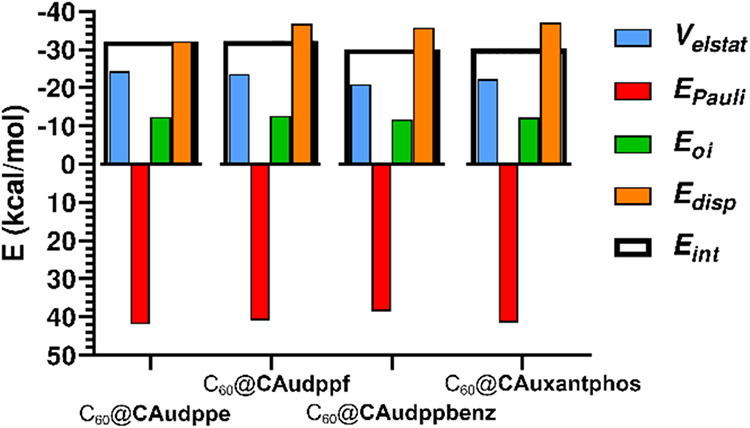
Graph bar of the different contributions to the interaction energy between molecular tweezer hosts and C_60_ according to EDA scheme.

As expected from the nature of these hosts, the highest contribution to the overall stabilization belongs to the dispersion term which accounts for approximately half of the attractive interaction forces, followed by electrostatic interactions and, finally, a small contribution from orbital interactions, suggesting a very weak charge-transfer complex in the ground state. Resulting interaction energies obtained from this scheme provides more reasonable values around −30 kcal/mol and accurately reproduces the trend observed in the experimental association constants for C_60_ recognition, being **CAudppf** the best host, followed by complex **CAudppe**. Moreover, compounds **CAudppbenz** and **CAuxantphos** possess the lowest interaction energy, as observed experimentally.

This decomposition method was also applied to decipher the nature of the intramolecular aurophilic interaction for all hosts in their corresponding assemblies. In order to do so, Cockroft’s fragmentation analysis was carried out^7b^ (see the Supporting Information for more details about the dissection scheme) Results show that dispersive forces are much less important than electrostatic and orbital interactions, as a consequence of the remarkable electron correlation effects present in this metal owing to strong relativistic effects. This has been already shown by Cockroft^7b^ and Nitsch and Guerra,^7a^ confirming the latest findings in this regard. In terms of the overall intramolecular aurophilic interaction energy, the trend resulted as follows (in kcal/mol): 0.1, −5.9, −6.1, and −7.9 for **CAudppe**, **CAudppf**, **CAudppbenz**, and **CAuxantphos**, respectively. These values correlate moderately well (Figure S150) with the ones estimated by using the empirical eq 1 (Table 2) being near zero for host **CAudppe** and high for hosts **CAudppbenz** and **CAuxantphos**. For complex **CAudppf** it was found that EDA furnishes a more stabilizing situation than the empirical estimation. In all cases the values fall within the 5–15 kcal/mol range and are moderately low when compared with the dispersion interactions established between fullerene and corannulene moieties in the supramolecular adducts. Aurophilic interaction energies were also computed by carrying out the Interacting Quantum Atoms (IQA) formalism^31^ that makes use of the Quantum Theory of Atoms in Molecules (QTAIM). Its robustness rely on the independence from the theoretical framework and the fragment definition. Moreover, it has proven excellent results in gold-containing species.^32^ The trend in Au­(I)–Au­(I) interaction is as follows (in kcal/mol): 3.3, −8.8, −16.0, and −19.8 for **CAudppe**, **CAudppf**, **CAudppbenz** and **CAuxantphos**, respectively. These higher values, compared to EDA results, indicate a stronger interaction. Nonetheless the variation resembles a quasi-linear behavior along the series of hosts (Figure S151).

## Conclusions

It has been experimentally demonstrated that fullerene recognition with corannulene-based molecular tweezers bearing two Au­(I) atoms primarily depends on host preorganization. The potential Au­(I)–Au­(I) contact plays a small role within the manifold forces that are at stake since hosts with assured aurophilic interactions due to diphosphine design (the case of hosts **CAudppbenz** and **CAuxantphos**) did not substantially increase recognition capabilities. This is due to their intrinsic rigidity and lack of adaptation capacity. In fact, this feature could pose a countereffect. On the other hand, extremely flexible hosts (the case of complex **CAuddpe**), with absolutely no driving force to establish intramolecular Au­(I)–Au­(I) interaction, show moderate fullerene recognition. This is expected due to the strong deformation penalty that must be paid owing to their poor preorganization. The most interesting host turned out to be complex **CAudppf**. The stable tweezer-like conformation in solution (*syn* conformation, as experimentally observed in solid state) or a fast Cp rotation, it is well preorganized despite no intramolecular metallophilic interaction. It provided the best experimental association constants, showed the best electronic interaction energy with fullerenes and the lowest deformation penalty, being the best host for fullerenes of the family. Additionally, extensive computational calculations strongly suggested that an intramolecular aurophilic contact could potentially arise upon fullerene complexation in **CAudppf** host due to Au­(I)–Au­(I) distance shortening (not observed for any of the other members of the family), therefore contributing to a small further stabilization of the supramolecular complex.

## Experimental Section

### General Experimental Techniques

All reagents were purchased from commercial sources and used without further purification. 1-Bromocorannulene was acquired from Synoi Chemicals (http://synoichemicals.uva.es/). Solvents were of analytical grade or spectrophotometric grade. They were either used as purchased or dried according to procedures described elsewhere.^33^ Reactions were performed under an inert atmosphere with standard Schlenk techniques. Purifications by centrifugation were performed in a Nahita 2600. The NMR spectra were recorded on a 400 MHz Agilent NMR, a 500 MHz Agilent DD2 instrument equipped with a OneNMR probe, or a 500 MHz Agilent DD2 instrument equipped with a cold probe. NMR titrations were recorded on a 500 MHz Agilent DD2 instrument equipped with a cold probe in the Laboratory of Instrumental Techniques (LTI) Research Facilities, University of Valladolid. ^1^H, ^13^C, and ^31^P NMR chemical shifts (*d*) are reported in parts per million (ppm) and are referenced to tetramethylsilane (TMS) using the residual solvent peak as an internal reference. Coupling constants (*J*) are reported in Hz. Standard abbreviations are used to indicate multiplicity: s, singlet; d, doublet; t, triplet; and m, multiplet. For broad signals, the label *br* is reported. ^1^H and ^13^C peak assignments were performed using 2D NMR methods (DQFCOSY, band-selective ^1^H–^13^C HSQC, band-selective ^1^H–^13^C HMBC, ^1^H–^31^P HMBC). Due to the low solubility, some carbon signals were detected indirectly via ^1^H–^13^C HSQC/HMBC experiments and labeled as *in*. High-resolution mass spectra were recorded at the mass spectrometry service of the LTI, University of Valladolid and at the mass spectrometry service of the University of Burgos. A MALDI-TOF system (Bruker Autoflex Speed), a MS-TOF system (Bruker Maxis Impact) and MS-QTOF (6545 Q-TOF Agilent) with electrospray ionization (positive and negative ESI) were utilized. Steady-state UV/vis absorption spectroscopy was carried out on a PerkinElmer Lambda 265 spectrophotometer, whereas emission spectroscopy was performed on a Cary Eclipse (Agilent) fluorescence spectrophotometer using quartz cuvettes with a path length of 1 cm in DCM as the solvent. Cyclic voltammetry was carried out at room temperature using a PalmSens4 potentiostat, with a 0.10 M solution of tetrabutylammonium hexafluorophosphate (NBu_4_PF_6_) as the supporting electrolyte in DMF as the solvent at a scan rate of 100 mV/s in all of the experiments. The analyte concentration was 1 mM. Solutions were deaerated with a nitrogen stream prior to each measurement. Experiments were performed in a one-compartment cell equipped with a round glassy carbon electrode (diameter of 3 mm), a silver wire counter electrode, and a Ag/AgCl wire as pseudo- reference electrode. The working electrode was cleaned using mechanical polishing on a surface with a water-alumina slurry.^34^ All potentials were referenced against the ferrocene/ferrocenium couple (Fc/Fc^+^) after each experiment and plotted with IUPAC convention. Diffraction data were collected using an Oxford Diffraction Supernova diffractometer equipped with an Atlas CCD area detector and a four-circle kappa goniometer. For the data collection, Mo or Cu microfocused sources with multilayer optics were used. When necessary, crystals were mounted directly from solution using perfluorohydrocarbon oil to prevent atmospheric oxidation, hydrolysis, and solvent loss. Data integration, scaling, and empirical absorption correction were performed using the CrysAlisPro software package. The structure was solved by direct methods and refined by full-matrix-least-squares against F2 with SHELX in OLEX2. Non-hydrogen atoms were refined anisotropically, and hydrogen atoms were placed at idealized positions and refined using the riding model. Graphics were made using OLEX2 and MERCURY. 1-Trimethylsilylacetylene arenes and 1-ethynylcorannulene were synthesized following reported methods.^3e,35^ Chloro­(organophosphine) gold­(I) complexes were prepared according to literature procedures.^10,36^

### Method A for Preparation of Au­(I) Acetylide Complexes

The corresponding trimethylsilylacetylene (1.1 equiv per gold atom) and chloro­(organophosphine) gold­(I) (1.0 equiv, 50 μmol) complex were dissolved under inert atmosphere in dry EtOH so that the concentration of the parent gold­(I) complex is 5 mM. Tetra-*n*-butylammonium fluoride (TBAF) (1 M in THF, 3 equiv per trimethylsilylacetylene) was added to the solution and the mixture was heated to reflux for 4 h. The resulting Au­(I) acetylide complex precipitates as a yellow/orange solid, which was separated from the solution by centrifugation, washed with 3 portions of EtOH and 3 portions of *n*-hexane, and dried under vacuum.

### Method B for Preparation of Au­(I) Acetylide Complexes

The corresponding aryl acetylene (1.1 equiv per gold atom), chloro­(organophosphine) gold­(I) complex (1.0 equiv, 20 μmol), and NaOMe (3 equiv per gold atom) were dissolved under inert atmosphere in 1:1 DCM/MeOH mixture so that the concentration of the parent gold­(I) complex is 4 mM. The mixture was heated at 45 °C overnight. Then, the solvent was removed under vacuum, and the residue was redissolved in 2 mL of DCM, transferred to a separatory funnel, and washed with H_2_O (2 × 2 mL). The organic layer was separated, dried with anhydrous MgSO_4_, filtered and concentrated in a rotary evaporator. The solid was dissolved with the minimum amount of DCM (typically 1 mL) and hexane was carefully added to the solution. The resulting Au­(I) acetylide complex precipitates as a yellow/orange solid, which was separated by centrifugation, washed with 3 portions of *n*-hexane, and dried under vacuum.

#### PAuPPh_3_

Method A was followed using 1-[(trimethylsilyl)­ethynyl]­pyrene and [AuCl­(PPh_3_)]. Isolated as a yellow solid (29 mg, 84% yield). ^1^H NMR (500 MHz, CDCl_3_) δ: 8.87 (d, *J* = 9.1 Hz, 1H, H^10^), 8.21 (d, *J* = 7.9 Hz, 1H, H^2^), 8.17 (d, *J* = 7.6 Hz, 1H, H^8^), 8.13 (d, *J* = 7.6 Hz, 1H, H^6^), 8.12 (d, *J* = 9.1 Hz, 1H, H^9^), 8.18–8.10 (m, 3H, H^8^ + H^6^ + H^9^), 8.06 (d, *J* = 7.9 Hz, 1H, H^3^), 8.02 (s, 2H, H^4^ + H^5^), 7.98 (t, *J* = 7.6 Hz, 1H, H^7^), 7.66–7.59 (m, 6H, H^20^), 7.55–7.47 (m, 9H, H^21^ + H^22^). ^31^P NMR (162 MHz, CDCl_3_) δ: 42.31. ^13^C­{^1^H} NMR (101 MHz, CDCl_3_) δ: 134.4 (d, ^2^ *J*_13C–31P_ = 13.8 Hz, C^20^), 132.3 (C^11^), 131.6 (d, ^4^ *J*_13C–31P_ = 2.4 Hz, C^22^), 131.4 (C^13^), 131.3 (C^14^), 130.6 (C^2^), 130.2 (C^12^), 129.8 (d, ^1^ *J*_13C–31P_ = 55.8 Hz, C^19^), 129.2 (d, ^3^ *J*_13C–31P_ = 11.3 Hz, C^21^), 127.5 (C^9^), 127.32 (C^4^), 127.26 (C^5^), 126.8 (C^10^), 125.8 (C^7^), 125.0 (C^8^), 124.9 (C^6^), 124.5 (C^15^ + C^16^), 124.4 (C^3^), 102.6 (C^17^). HRMS (MALDI-TOF): *m*/*z* = 684.1306 [M]^+^, calculated 684.1276 for C_36_H_24_AuP.

#### PAudppe

Method A was followed using 1-[(trimethylsilyl)­ethynyl]­pyrene and [(AuCl)_2_dppe]. Isolated as a yellow solid (55 mg, 89% yield). ^1^H NMR (500 MHz, CDCl_3_) δ: 8.89 (d, *J* = 9.1 Hz, 2H, H^10^), 8.23 (d, *J* = 7.9 Hz, 2H, H^2^), 8.17 (d, *J* = 7.6 Hz, 2H, H^8^), 8.15 (d, *J* = 7.6 Hz, 2H, H^6^), 8.13 (d, *J* = 9.1 Hz, 2H, H^9^), 8.09 (d, *J* = 7.9 Hz, 2H, H^3^), 8.04 (d, *J* = 9.0 Hz, 2H, H^4^), 8.02 (d, *J* = 9.0 Hz, 2H, H^5^), 7.99 (t, *J* = 7.6 Hz, 2H, H^7^), 7.85–7.69 (m, 8H, H^21^), 7.54 (m, 12H, H^22^ + H^23^), 2.80 (br s, 4H, H^19^). ^31^P NMR (202 MHz, CDCl_3_) δ: 39.98. ^13^C­{^1^H} NMR (126 MHz, CDCl_3_) δ: 133.5 (t, ^2^ *J*_13C–31P_ = 7.0 Hz, C^21^), 132.4 (C^11^), 132.2 (br s, C^23^), 131.4 (C^13^), 131.3 (C^14^), 130.5 (C^2^), 130.3 (C^12^), 129.6 (t, ^3^ *J*_13C–31P_ = 5.8 Hz, C^22^), 128.9 (t, ^1^ *J*_13C–31P_ = 24.0 Hz, C^20^) 127.6 (C^9^), 127.4 (C^4^), 127.3 (C^5^), 126.7 (C^10^), 125.9 (C^7^), 125.1 (C^8^), 125.0 (C^6^), 124.6 (C^16^), 124.50 (C^15^), 124.46 (C^3^), 119.9 (C^1^), 102.9 (C^17^), 24.1 (dd, ^1^ *J*_13C–31P_ = 21.6, 15.8 Hz, C^19^). HRMS (MALDI-TOF): *m*/*z* = 1242.2050 [M]^+^, calculated 1242.2088 for C_62_H_42_Au_2_P_2_.

#### PAudppf

Method A was followed using 1-[(trimethylsilyl)­ethynyl]­pyrene and [(AuCl)_2_dppf]. Isolated as an orange solid (64 mg, 92% yield). ^1^H NMR (400 MHz, CDCl_3_) δ: 8.91 (d, *J* = 9.1 Hz, 2H, H^10^), 8.21 (d, *J* = 7.9 Hz, 2H, H^2^), 8.15 (d, *J* = 7.4 Hz, 2H, H^8^), 8.14 (d, *J* = 7.4 Hz, 2H, H^6^), 8.09 (d, *J* = 9.1 Hz, 2H, H^9^), 8.07 (d, *J* = 7.9 Hz, 2H, H^3^), 8.02 (s, 4H, H^4^ + H^5^), 7.98 (t, *J* = 7.4 Hz, 2H, H^7^), 7.67–7.58 (m, 8H, H^23^), 7.52–7.39 (m, 12H, H^25^ + H^24^), 4.85 (br s, 4H, H^21^), 4.43 (br s, 4H, H^20^). ^31^P NMR (162 MHz, CDCl_3_) δ: 36.77. ^13^C­{^1^H} NMR (101 MHz, CDCl_3_) δ: 133.8 (d, ^2^ *J*_13C–31P_ = 14.0 Hz, C^23^), 132.4 (C^11^), 131.5 (br s, C^25^), 131.4 (d, ^1^ *J*_13C–31P_ = 57.3 Hz, C^22^), 131.4 (C^13^), 131.3 (C^14^), 130.5 (C^2^), 130.2 (C^12^), 129.0 (d, ^3^ *J*_13C–31P_ = 11.3 Hz, C^24^), 127.5 (C^9^), 127.3 (C^4^), 127.3 (C^5^), 126.8 (C^10^), 125.9 (C^7^), 125.0 (C^8^), 124.9 (C^6^), 124.6 (C^16^), 124.5 (C^15^), 124.4 (C^3^), 120.2 (C^1^), 75.1 (C^21^), 75.0 (d, ^2^ *J*_13C–31P_ = 21.1 Hz, C^20^), 72.1 (d, ^1^ *J*_13C–31P_ = 64.1 Hz, C^19^). HRMS (ESI-TOF): *m*/*z* = 1421.1626 [M + Na]^+^, calculated 1421.1650 for C_70_H_46_Au_2_FeNaP_2_. *m*/*z* = 1437.1372 [M + K]^+^, calculated 1437.1389 for C_70_H_46_Au_2_FeKP_2_.

#### PAudppbenz

Method A was followed using 1-[(trimethylsilyl)­ethynyl]­pyrene and [(AuCl)_2_dppbenz]. Isolated as a yellow solid (55 mg, 85% yield). ^1^H NMR (500 MHz, CDCl_3_) δ: 8.94 (d, *J* = 9.1 Hz, 2H, H^10^), 8.14 (d, *J* = 7.9 Hz, 2H, H^2^), 8.04 (d, *J* = 7.4 Hz, 2H, H^6^), 7.96 (d, *J* = 7.9 Hz, 2H, H^3^), 7.95 (s, 4H, H^4^ + H^5^), 7.84 (t, *J* = 7.4 Hz, 2H, H^7^), 7.81 (d, *J* = 7.4 Hz, 2H, H^8^), 7.68 (d, *J* = 9.1 Hz, 2H, H^9^), 7.65–7.59 (m, 8H, H^23^), 7.56–7.48 (m, 6H, H^21^ + H^25^), 7.46–7.41 (m, 8H, H^24^), 7.35–7.28 (m, 2H, H^20^). ^31^P NMR (202 MHz, CDCl_3_) δ: 34.27. ^13^C­{^1^H} NMR (126 MHz, CDCl_3_) δ: 137.0 (t, ^2^ *J*_13C–31P_ = 7.0 Hz, C^20^), 134.8 (t, ^2^ *J*_13C–31P_ = 7.0 Hz, C^23^), 132.4 (C^11^), 131.7 (C^25^), 131.4 (C^21^), 131.32 (C^14^), 131.27 (C^13^), 130.3 (C^2^), 130.1 (C^q^), 129.9 (C^q^), 129.7 (C^12^), 129.2 (t, ^3^ *J*_13C–31P_ = 5.7 Hz, C^24^), 127.4 (C^10^), 127.3 (C^4^), 127.2 (C^9^), 126.8 (C^5^), 125.5 (C^7^), 124.7 (C^8^), 124.5 (C^15^ + C^16^), 124.4 (C^6^), 124.2 (C^3^), 121.4 (C^1^), 103.7 (C^17^, *in*). HRMS (ESI-TOF): *m*/*z* = 1313.1978 [M + Na]^+^, calculated 1313.1985 for C_66_H_42_Au_2_NaP_2_. *m*/*z* = 1329.1723 [M + K]^+^, calculated 1329.1725 for C_66_H_42_Au_2_KP_2_.

#### PAuxantphos

Method A was followed using 1-[(trimethylsilyl)­ethynyl]­pyrene and [(AuCl)_2_xantphos]. Isolated as a yellow solid (57 mg, 80% yield). ^1^H NMR (500 MHz, CDCl_3_) δ: 9.07 (d, *J* = 9.0 Hz, 2H, H^10^), 8.16 (d, *J* = 7.8 Hz, 2H, H^2^), 8.08 (d, *J* = 7.4 Hz, 2H, H^6^), 8.00 (d, *J* = 7.8 Hz, 4H, H^3^ + H^8^), 7.97 (s, 2H, H^4^ + H^5^), 7.91 (t, *J* = 7.4 Hz, 2H, H^7^), 7.71 (d, *J* = 9.0 Hz, 2H, H^9^), 7.63 (d, *J* = 7.46 Hz, 2H, H^22^), 7.49 (br, 4H, H^30^), 7.37 (br, 8H, H^28^), 7.23 (br s, 8H, H^29^), 7.08 (t, *J* = 7.4 Hz, 2H, H^21^), 6.53 (dd, 2H, H^20^), 1.70 (s, 6H, H^26^). ^31^P NMR (162 MHz, CDCl_3_) δ: 32.09. ^13^C­{^1^H} NMR (126 MHz, CDCl_3_) δ: 153.2 (C^24^, *in*), 134.7 (d, *J* = 14.3 Hz, C^30^), 133.0 (C^20^), 132.5 (C^11^) 131.6 (C^23^, *in*), 131.4 (C^13^ + C^14^), 131.0 (C^28^), 130.7 (C^27^, *in*), 130.2 (C^2^), 129.6 (C^12^), 128.9 (C^22^), 128.8 (C^29^), 127.7 (C^10^), 127.4 (C^4^), 127.2 (C^9^), 126.7 (C^5^), 125.6 (C^7^), 124.6–123.9 (C^3^ + C^6^ + C^8^ + C^15^ + C^16^ + C^21^), 121.9 (C^1^), 118.1 (C^19^, *in*), 104.1 (C^17^, *in*) 34.8 (C^25^, *in*), 31.1 (C^26^). HRMS (ESI-TOF): *m*/*z* = 1197.2201 [M–C≡C-pyr]^+^, calculated 1197.1964 for C_57_H_41_Au_2_OP_2_.

#### CAuPPh_3_

Method A was followed using 1-[(trimethylsilyl)­ethynyl]­corannulene and [AuCl­(PPh_3_)]. Isolated as a yellow solid (24 mg, 66% yield). ^1^H NMR (500 MHz, CDCl_3_) δ: 8.26 (d, *J* = 8.7 Hz, 1H, H^10^), 8.00 (s, 1H, H^2^), 7.82 (d, *J* = 8.7 Hz, 1H, H^9^), 7.79 (s, 2H, H^7^ + H^8^), 7.77 (s, 2H, H^5^ + H^6^), 7.76 (d, *J* = 8.7 Hz, 1H, H^4^), 7.71 (d, *J* = 8.7 Hz, 1H, H^3^), 7.64–7.57 (m, 6H, H^24^), 7.56–7.44 (m, 9H, H^26^ + H^25^). ^31^P NMR (162 MHz, CDCl_3_) δ: 42.22. ^13^C­{^1^H} NMR (101 MHz, CDCl_3_) δ: 136.2 (C^18^), 135.8 (C^17^), 135.7 (C^16^), 135.1 (C^20^), 134.6 (C^19^), 134.4 (d, ^2^ *J*_13C–31P_ = 13.8 Hz, C^24^), 132.0 (C^11^), 131.6 (d, ^4^ *J*_13C–31P_ = 1.9 Hz, C^26^), 131.1 (C^2^), 131.0 (C^15^), 130.9 (C^14^), 130.8 (C^13^), 130.7 (C^12^), 129.8 (d, ^1^ *J*_13C–31P_ = 55.8 Hz, C^23^), 129.2 (d, ^3^ *J*_13C–31P_ = 11.3 Hz, C^25^), 127.2–126.8 (m, C^4^–C^10^), 126.7 (C^3^), 123.5 (C^1^). HRMS (MALDI-TOF): *m*/*z* = 732.1286 [M]^+^, calculated 732.1276 for C_40_H_24_AuP.

#### CAudppe

Method B was followed using 1-(ethynyl)­corannulene and [(AuCl)_2_dppe]. Isolated as a yellow solid (24 mg, 91% yield). ^1^H NMR (500 MHz, CDCl_3_) δ: 8.27 (d, *J* = 8.8 Hz, 2H, H^10^), 8.02 (s, 2H, H^2^), 7.82 (d, *J* = 8.8 Hz, 2H, H^9^), 7.80 (s, 4H, H^7^ + H^8^), 7.78 (s, 4H, H^5^ + H^6^), 7.77 (d, *J* = 8.7 Hz, 2H, H^4^), 7.76–7.73 (m, 8H, H^25^), 7.72 (d, *J* = 8.7 Hz, 2H, H^3^), 7.57–7.50 (m, 12H, H^26^ + H^27^), 2.76 (br s, 4H, H^23^). ^31^P NMR (202 MHz, CDCl_3_) δ: 39.86. ^13^C­{^1^H} NMR (126 MHz, CDCl_3_) δ: 136.2 (C^18^), 135.8 (C^17^), 135.7 (C^16^), 135.1 (C^20^), 134.7 (C^19^), 133.5 (t, ^2^ *J*_13C–31P_ = 6.8 Hz, C^25^), 132.2 (C^27^), 131.9 (C^11^), 131.1 (C^2^), 131.0 (C^15^), 130.82 (C^12^ + C^12^), 130.79 (C^13^), 129.6 (t, ^3^ *J*_13C–31P_ = 5.6 Hz, C^26^), 128.8 (t, ^1^ *J*_13C–31P_ = 27.1 Hz, C^24^), 127.3–126.8 (C^4^–C^10^), 126.7 (C^3^), 123.3 (C^1^), 24.0 (C^23^). HRMS (MALDI-TOF): *m*/*z* = 1338.2061 [M]^+^, calculated 1338.2088 for C_70_H_42_Au_2_P_2_.

#### CAudppf

Method B was followed using 1-(ethynyl)­corannulene and [(AuCl)_2_dppf]. Isolated as an orange solid (28 mg, 92% yield). ^1^H NMR (500 MHz, CDCl_3_) δ: 8.30 (d, *J* = 8.8 Hz, 2H, H^10^), 8.00 (s, 2H, H^2^), 7.82 (d, *J* = 8.8 Hz, 2H, H^9^), 7.79 (s, 4H, H^7^ + H^8^), 7.78 (s, 4H, H^5^ + H^6^), 7.77 (d, *J* = 8.7 Hz, 2H, H^4^), 7.71 (d, *J* = 8.7 Hz, 2H, H^3^), 7.64–7.56 (m, 8H, H^27^), 7.49–7.39 (m, 12H, H^28^ + H^29^), 4.81 (br s, 4H, H^25^), 4.42 (br s, 4H, H^24^). ^31^P NMR (162 MHz, CDCl_3_) δ: 36.66. ^13^C­{^1^H} NMR (126 MHz, CDCl_3_) δ: 136.2 (C^18^), 135.8 (C^17^), 135.7 (C^16^), 135.1 (C^20^), 134.6 (C^19^), 133.7 (d, ^2^ *J*_13C–31P_ = 14.1 Hz, C^27^), 132.0 (C^11^), 131.5 (br s, C^29^), 131.3 (d, ^1^ *J*_13C–31P_ = 53.2 Hz, C^26^), 131.0 (C^15^), 130.93 (C^2^), 130.87 (C^14^), 130.8 (C^13^), 130.7 (C^12^), 129.0 (d, ^3^ *J*_13C–31P_ = 11.4 Hz, C^28^), 127.1–126.9 (C^4^ – C^10^), 126.7 (C^3^), 75.1 (d, ^2^ *J*_13C–31P_ = 2,7 Hz, C^24^), 75.0 (C^25^) 72.1 (C^23^, ^1^ *J*_13C–31P_ = 67.2 Hz, *in*). HRMS (ESI-TOF): *m*/*z* = 1517.1690 [M + Na]^+^, calculated 1517.1650 for C_78_H_46_Au_2_FeNaP_2_. *m*/*z* = 1533.1451 [M + Na]^+^, calculated 1533.1390 for C_78_H_46_Au_2_FeKP_2_.

#### CAudppbenz

Method B was followed using 1-(ethynyl)­corannulene and [(AuCl)_2_dppbenz]. Isolated as a yellow solid (24 mg, 86% yield). ^1^H NMR (500 MHz, CDCl_3_) δ: 8.23 (d, *J* = 8.7 Hz, 2H, H^10^), 7.89 (s, 2H, H^2^), 7.70 (d, *J* = 8.8 Hz, 2H, H^6^), 7.68 (d, *J* = 8.8 Hz, 2H, H^5^), 7.60 (d, *J* = 8.8 Hz, 4H, H^4^ + H^7^), 7.58–7.54 (m, 8H, H^27^), 7.53–7.47 (m, 6H, H^26^ + H^30^), 7.46 (d, *J* = 8.8 Hz, 2H, H^3^), 7.41 (m, 8H, H^28^), 7.35 (d, *J* = 8.8 Hz, 2H, H^8^), 7.32–7.27 (m, 2H, H^24^), 7.18 (d, *J* = 8.7 Hz, 2H, H^9^). ^31^P NMR (162 MHz, CDCl_3_) δ: 34.30. ^13^C­{^1^H} NMR (101 MHz, CDCl_3_) δ: 137.0 (t, ^2^ *J*_13C–31P_ = 6.8 Hz, C^24^), 135.9 (C^19^), 135.7 (C^20^), 135.3 (C^16^), 134.9 (C^17^), 134.8 (t, ^2^ *J*_13C–31P_ = 7.1 Hz, C^27^), 134.2 (C^18^), 132.3 (C^11^), 131.7 (C^29^), 131.3 (t, ^3^ *J*_13C–31P_ = 2.2 Hz, C^25^), 130.9 (C^12^), 130.7 (C^15^), 130.5 (C^14^), 130.4 (C^13^), 130.2 (C^2^) 129.8 (d, ^1^ *J*_13C–31P_ = 27.5 Hz, C^26^), 129.2 (t, ^3^ *J*_13C–31P_ = 5.7 Hz, C^28^), 127.6 (C^10^), 126.9 (C^8^), 126.71 (C^5^), 126.67 (C^4^), 126.65 (C^9^), 126.60 (C^6^), 126.58 (C^3^), 126.5 (C^7^), 124.6 (C^1^), 103.0 (C^21^). HRMS (ESI-TOF): *m*/*z* = 1409.1985 [M + Na]^+^, calculated 1409.1985 for C_74_H_42_Au_2_NaP_2_.

#### CAuxantphos

Method B was followed using 1-(ethynyl)­corannulene and [(AuCl)_2_xantphos]. Isolated as a yellow solid (23 mg, 76% yield). ^1^H NMR (500 MHz, CDCl_3_) δ: 8.34 (d, *J* = 8.6 Hz, 2H, H^10^), 7.91 (s, 2H, H^2^), 7.74 (s, 8H, 4H^cor^), 7.70 (d, *J* = 9.0 Hz, 2H, H^cor^), 7.62 (m, 4H, H^cor^ + H^26^), 7.42 (s, 8H, H^33^), 7.37–7.31 (m, 6H, H^9^ + H^34^), 7.20 (br t, 8H, H^32^), 7.07 (t, *J* = 7.7 Hz, 2H, H^25^), 6.53–6.47 (m, 2H, H^24^), 1.70 (s, 6H, H^30^). ^31^P NMR (162 MHz, CDCl_3_) δ: 31.92. ^13^C­{^1^H} NMR (126 MHz, CDCl_3_) δ: 153.0 (C^28^, *in*), 136.1 (C^q^), 135.8 (C^q^), 135.6 (C^16^), 135.1 (C^17^), 134.6 (d, ^3^ *J*_13C–31P_ = 12.6 Hz, C^33^), 134.3 (C^18^), 133.0 (br s, C^24^), 132.7 (br s, C^11^), 131.6 (br s, C^27^), 131.2 (C^q^), 131.0 (C^34^), 130.9 (C^15^), 130.6 (C^q^), 130.5 (C^q^), 129.9 (C^2^), 128.9 (C^32^), 128.8 (C^26^), 128.0 (C^10^), 127.0–126.5 (C^3^ + C^4^ + C^5^ + C^6^ + C^7^ + C^8^ + C^9^), 125.1 (C^1^, *in*), 124.2 (C^25^), 118.0 (C^23^, *in*), 103.6 (C^21^, *in*), 34.7 (C^29^, *in*), 31.2 (C^30^). HRMS (ESI-TOF): *m*/*z* = 1245.2411 [M–C≡C-cor]^+^, calculated 1245.1946 for C_61_H_41_Au_2_OP_2_.

#### *p*-TolylAuxantphos

Method B was followed using 1-ethynyl-4methylbenzene and [(AuCl)_2_xantphos]. Isolated as a white solid (20 mg, 85% yield). ^1^H NMR (500 MHz, CDCl_3_) δ: 7.56 (d, *J* = 7.6 Hz, 2H, H^11^), 7.45–7.35 (m, 12H, H^18^ + H^19^), 7.32 (d, *J* = 7.5 Hz, 2H, H^2^), 7.27–7.19 (m, 8H, H^17^), 7.04 (d, *J* = 7.6 Hz, 2H, H^10^), 6.98 (d, *J* = 7.5 Hz, 2H, H^3^), 6.49 (t, *J* = 7.6 Hz, 2H, H^9^), 2.29 (s, 6H, H^5^), 1.63 (s, 6H, H^15^). ^31^P NMR (202 MHz, CDCl_3_) δ 32.23 ^13^C (*in*) δ: 153.3 (C^13^), 134.6 (C^18^ or C^19^), 132.7 (C^9^), 131.5 (C^12^), 132.1 (C^2^), 130.8 (C^18^ or C^19^), 128.7 (C^17^), 128.5 (C^3^), 128.4 (C^11^), 124.1 (C^10^), 118.4 (C^8^), 105.0 (C^6^), 34.7 (C^14^), 30.6 (C^15^), 21.3 (C^5^). HRMS (ESI-TOF): *m*/*z* = 1225.2219 [M + Na]^+^, calculated 1225.2247 for C_57_H_46_Au_2_NaOP_2_.

## Supplementary Material



## Notes

NMR, HRMS, UV–vis absorption and emission spectra, cyclic voltammetry data, X-ray crystallographic data, association constants measurements, and additional computational calculations details (PDF)
