# Supplementary material for: Golden Molecular Tweezers: Dinuclear Corannulene–Au(I) Acetylide Hosts for Fullerene Binding
Source: Inorg Chem. 2026 Apr 1;65(14):7704–17. doi: 10.1021/acs.inorgchem.5c05787 (PMC13080973; doi:10.1021/acs.inorgchem.5c05787)
Supplement: Supplementary file 1 [file ic5c05787_si_001.pdf]

Golden molecular tweezers: dinuclear corannulene–Au(I) acetylide hosts for fullerene binding

Nerea Álvarez-Llorente,[a] Alberto Diez-Varga,[a] Eric Masson,[b] Héctor Barbero,[a,]* Celedonio M. Álvarez[a,]*

Table of contents

General methods 3

Synthetic procedures 4

Selected NMR spectra 8

^{1}H-NMR VT Experiments 63

High Resolution Mass Spectra 65

UV-Vis and emission spectra 71

Electrochemical studies 81

X-Ray crystallography structures 86

^{1}H NMR Titration Experiments 89

Computational Calculations details 106

References 130

[a] GIR MIOMeT, IU CINQUIMA/Química Inorgánica, Facultad de Ciencias, Universidad de Valladolid, Valladolid, E47011, Spain. E-mail: hector.barbero@uva.es, celedonio.alvarez@uva.es

[b] Department of Chemistry and Biochemistry, Ohio University, Athens, Ohio 45701, USA.

General methods

All reagents were purchased from commercial sources and used without further purification. 1-Bromocorannulene was acquired from Synoi Chemicals (http://synoichemicals.uva.es/). Solvents were of analytical grade or spectrophotometric grade. They were either used as purchased or dried according to procedures described elsewhere.[1] Reactions were performed under an inert atmosphere with standard Schlenk techniques. Purifications by centrifugation were performed in a Nahita 2600. The NMR spectra were recorded on a 400 MHz Agilent NMR, a 500 MHz Agilent DD2 instrument equipped with a OneNMR probe, or a 500 MHz Agilent DD2 instrument equipped with a cold probe. NMR titrations were recorded on a 500 MHz Agilent DD2 instrument equipped with a cold probe in the Laboratory of Instrumental Techniques (LTI) Research Facilities, University of Valladolid. ^{1}H, ^{13}C, and ^{31}P NMR chemical shifts (δ) are reported in parts per million (ppm) and are referenced to tetramethylsilane (TMS) using the residual solvent peak as an internal reference. Coupling constants (J) are reported in Hz. Standard abbreviations are used to indicate multiplicity: s, singlet; d, doublet; t, triplet; and m, multiplet. For broad signals, the label *br* is reported. ^{1}H and ^{13}C peak assignments were performed using 2D NMR methods (DQFCOSY, band-selective ^{1}H-^{13}C HSQC, band-selective ^{1}H-^{13}C HMBC, ^{1}H-^{31}P HMBC). Due to the low solubility, some carbon signals were detected indirectly via ^{1}H-^{13}C HSQC/HMBC experiments and labeled as *in*. High-resolution mass spectra were recorded at the mass spectrometry service of the LTI, University of Valladolid and at the mass spectrometry service of the University of Burgos. A MALDI-TOF system (Bruker Autoflex Speed), a MS-TOF system (Bruker Maxis Impact) and MS-QTOF (6545 Q-TOF Agilent) with electrospray ionization (positive and negative ESI) were utilized. Steady-state UV/Vis absorption spectroscopy was carried out on a Perkin Elmer Lambda 265 spectrophotometer, whereas emission spectroscopy was performed on a Cary Eclipse (Agilent) fluorescence spectrophotometer using quartz cuvettes with a path length of 1 cm in DCM as the solvent. Cyclic voltammetry was carried out at room temperature using a PalmSens4 potentiostat, with a 0.10 M solution of tetrabutylammonium hexafluorophosphate (NBu_4PF_6) as the supporting electrolyte in DMF as the solvent at a scan rate of 100 mV/s in all of the experiments. The analyte concentration was 1 mM. Solutions were deaerated with a nitrogen stream prior to each measurement. Experiments were performed in a one-compartment cell equipped with a round glassy carbon electrode (diameter of 3 mm), a silver wire counter electrode, and a Ag/AgCl wire as pseudo-reference electrode. The working electrode was cleaned using mechanical polishing on a surface with a water-alumina slurry.[2] All potentials were referenced against the ferrocene/ferrocenium couple (Fc/Fc^{+}) after each experiment and plotted with IUPAC convention. Diffraction data were collected using an Oxford Diffraction Supernova diffractometer equipped with an Atlas CCD area detector and a four-circle kappa goniometer. For the data collection, Mo or Cu micro-focused sources with multilayer optics were used. When necessary, crystals were mounted directly from solution using perfluorohydrocarbon oil to prevent atmospheric oxidation, hydrolysis, and solvent loss. Data integration, scaling, and empirical absorption correction were performed using the CrysAlisPro software package. The structure was solved by direct methods and refined by full-matrix-least-squares against F2 with SHELX in OLEX2. Non-hydrogen atoms were refined anisotropically, and hydrogen atoms were placed at idealized positions and refined using the riding model. Graphics were made using OLEX2 and MERCURY. 1-Trimethylsilylacetylene arenes and 1-ethynylcorannulene were synthesized following reported methods.[3] Chloro(organophosphine) gold(I) complexes were prepared according to literature procedures.[4]

Synthetic procedures

Method A for preparation of Au(I) acetylide complexes

The corresponding trimethylsilylacetylene (1.1 equiv. per gold atom) and chloro(organophosphine) gold(I) (1.0 equiv., 50 μmol) complex were dissolved under inert atmosphere in dry EtOH so that the concentration of the parent gold(I) complex is 5 mM. Tetra-n-butylammonium fluoride (TBAF) (1M in THF, 3 equiv. per trimethylsilylacetylene) was added to the solution and the mixture was heated to reflux for 4 h. The resulting Au(I) acetylide complex precipitates as a yellow/orange solid, which was separated from the solution by centrifugation, washed with 3 portions of EtOH and 3 portions of *n*-hexane, and dried under vacuum.

Method B for preparation of Au(I) acetylide complexes

The corresponding aryl acetylene (1.1 equiv. per gold atom), chloro(organophosphine) gold(I) complex (1.0 equiv., 20 μmol), and NaOMe (3 equiv. per gold atom) were dissolved under inert atmosphere in 1:1 DCM/MeOH mixture so that the concentration of the parent gold(I) complex is 4 mM. The mixture was heated at 45 °C overnight. Then, the solvent was removed under vacuum, and the residue was redissolved in 2 mL of DCM, transferred to a separatory funnel, and washed with H_2O (2 × 2 mL). The organic layer was separated, dried with anhydrous $MgSO_4$, filtered and concentrated in a rotary evaporator. The solid was dissolved with the minimum amount of DCM (typically 1 mL) and hexane was carefully added to the solution. The resulting Au(I) acetylide complex precipitates as a yellow/orange solid, which was separated by centrifugation, washed with 3 portions of hexane, and dried under vacuum.

$PAuPPh_3$. Method A was followed using 1-[(trimethylsilyl)ethynyl]pyrene and [AuCl(PPh_3)]. Isolated as a yellow solid (29 mg, 84% yield). ^{1}H NMR (500 MHz, $CDCl_3$) δ: 8.87 (d, *J* = 9.1 Hz, 1H, H^{10}), 8.21 (d, *J* = 7.9 Hz, 1H, H^2), 8.17 (d, *J* = 7.6 Hz, 1H, H^8), 8.13 (d, *J* = 7.6 Hz, 1H, H^6), 8.12 (d, *J* = 9.1 Hz, 1H, H^9) , 8.18 – 8.10 (m, 3H, H^8 + H^6 + H^9), 8.06 (d, *J* = 7.9 Hz, 1H, H^3), 8.02 (s, 2H, H^4 + H^5), 7.98 (t, *J* = 7.6 Hz, 1H, H^7), 7.66 – 7.59 (m, 6H, H^{20}), 7.55 – 7.47 (m, 9H, H^{21} + H^{22}). ^{31}P NMR (162 MHz, $CDCl_3$) δ: 42.31. ^{13}C{^{1}H} NMR (101 MHz, $CDCl_3$) δ: 134.4 (d, $^2J_{13C\text{-}31P}$ = 13.8 Hz, C^{20}), 132.3 (C^{11}), 131.6 (d, $^4J_{13C\text{-}31P}$ = 2.4 Hz, C^{22}), 131.4 (C^{13}), 131.3 (C^{14}), 130.6 (C^2), 130.2 (C^{12}), 129.8 (d, $^1J_{13C\text{-}31P}$ = 55.8 Hz, C^{19}), 129.2 (d, $^3J_{13C\text{-}31P}$ = 11.3 Hz, C^{21}), 127.5 (C^9), 127.32 (C^4), 127.26 (C^5), 126.8 (C^{10}), 125.8 (C^7), 125.0 (C^8), 124.9 (C^6), 124.5 (C^{15} + C^{16}), 124.4 (C^3), 102.6 (C^{17}). HRMS (MALDI-TOF): m/z = 684.1306 $[M]^+$, calculated 684.1276 for $C_{36}H_{24}AuP$.

PAudppe. Method A was followed using 1-[(trimethylsilyl)ethynyl]pyrene and [$(AuCl)_2$dppe]. Isolated as a yellow solid (55 mg, 89% yield). ^{1}H NMR (500 MHz, $CDCl_3$) δ: 8.89 (d, *J* = 9.1 Hz, 2H, H^{10}), 8.23 (d, *J* = 7.9 Hz, 2H, H^2), 8.17 (d, *J* = 7.6 Hz, 2H, H^8), 8.15 (d, *J* = 7.6 Hz, 2H, H^6), 8.13 (d, *J* = 9.1 Hz, 2H, H^9), 8.09 (d, *J* = 7.9 Hz, 2H, H^3), 8.04 (d, *J* = 9.0 Hz, 2H, H^4), 8.02 (d, *J* = 9.0 Hz, 2H, H^5), 7.99 (t, *J* = 7.6 Hz, 2H, H^7), 7.85 – 7.69 (m, 8H, H^{21}), 7.54 (m, 12H, H^{22} + H^{23}), 2.80 (br s, 4H, H^{19}). ^{31}P NMR (202 MHz, $CDCl_3$) δ: 39.98. ^{13}C{^{1}H} NMR (126 MHz, $CDCl_3$) δ: 133.5 (t, $^2J_{13C\text{-}31P}$ = 7.0 Hz, C^{21}), 132.4 (C^{11}), 132.2 (br s, C^{23}), 131.4 (C^{13}), 131.3 (C^{14}), 130.5 (C^2), 130.3 (C^{12}), 129.6 (t, $^3J_{13C\text{-}31P}$ = 5.8 Hz, C^{22}), 128.9 (t, $^1J_{13C\text{-}31P}$ = 24.0 Hz, C^{20}) 127.6 (C^9), 127.4 (C^4), 127.3 (C^5), 126.7 (C^{10}), 125.9 (C^7), 125.1 (C^8), 125.0 (C^6), 124.6 (C^{16}), 124.50 (C^{15}), 124.46 (C^3), 119.9 (C^1), 102.9 (C^{17}), 24.1

(dd, $^{1}J_{13C\text{-}31P}$ = 21.6, 15.8 Hz, C^{19}). HRMS (MALDI-TOF): m/z = 1242.2050 $[M]^+$, calculated 1242.2088 for $C_{62}H_{42}Au_2P_2$.

PAudppf. Method A was followed using 1-[(trimethylsilyl)ethynyl]pyrene and $[(AuCl)_2dppf]$. Isolated as an orange solid (64 mg, 92% yield). ^{1}H NMR (400 MHz, $CDCl_3$) δ: 8.91 (d, *J* = 9.1 Hz, 2H, H^{10}), 8.21 (d, *J* = 7.9 Hz, 2H, H^2), 8.15 (d, *J* = 7.4 Hz, 2H, H^8), 8.14 (d, *J* = 7.4 Hz, 2H, H^6), 8.09 (d, *J* = 9.1 Hz, 2H, H^9), 8.07 (d, *J* = 7.9 Hz, 2H, H^3), 8.02 (s, 4H, H^4 + H^5), 7.98 (t, *J* = 7.4 Hz, 2H, H^7), 7.67 – 7.58 (m, 8H, H^{23}), 7.52 – 7.39 (m, 12H, H^{25} + H^{24}), 4.85 (br s, 4H, H^{21}), 4.43 (br s, 4H, H^{20}). ^{31}P NMR (162 MHz, $CDCl_3$) δ: 36.77. $^{13}C\{^1H\}$ NMR (101 MHz, $CDCl_3$) δ: 133.8 (d, $^{2}J_{13C\text{-}31P}$ = 14.0 Hz, C^{23}), 132.4 (C^{11}), 131.5 (br s, C^{25}), 131.4 (d, $^{1}J_{13C\text{-}31P}$ = 57.3 Hz, C^{22}), 131.4 (C^{13}), 131.3 (C^{14}), 130.5 (C^2), 130.2 (C^{12}), 129.0 (d, $^{3}J_{13C\text{-}31P}$ = 11.3 Hz, C^{24}), 127.5 (C^9), 127.3 (C^4), 127.3 (C^5), 126.8 (C^{10}), 125.9 (C^7), 125.0 (C^8), 124.9 (C^6), 124.6 (C^{16}), 124.5 (C^{15}), 124.4 (C^3), 120.2 (C^1), 75.1 (C^{21}), 75.0 (d, $^{2}J_{13C\text{-}31P}$ = 21.1 Hz, C^{20}), 72.1 (d, $^{1}J_{13C\text{-}31P}$ = 64.1 Hz, C^{19}). HRMS (ESI-TOF): m/z = 1421.1626 $[M + Na]^+$, calculated 1421.1650 for $C_{70}H_{46}Au_2FeNaP_2$. m/z = 1437.1372 $[M + K]^+$, calculated 1437.1389 for $C_{70}H_{46}Au_2FeKP_2$.

PAudppbenz. Method A was followed using 1-[(trimethylsilyl)ethynyl]pyrene and $[(AuCl)_2dppbenz]$. Isolated as a yellow solid (55 mg, 85% yield). ^{1}H NMR (500 MHz, $CDCl_3$) δ: 8.94 (d, *J* = 9.1 Hz, 2H, H^{10}), 8.14 (d, *J* = 7.9 Hz, 2H, H^2), 8.04 (d, *J* = 7.4 Hz, 2H, H^6), 7.96 (d, *J* = 7.9 Hz, 2H, H^3), 7.95 (s, 4H, H^4 + H^5), 7.84 (t, *J* = 7.4 Hz, 2H, H^7), 7.81 (d, *J* = 7.4 Hz, 2H, H^8), 7.68 (d, *J* = 9.1 Hz, 2H, H^9), 7.65 – 7.59 (m, 8H, H^{23}), 7.56 – 7.48 (m, 6H, H^{21} + H^{25}), 7.46 – 7.41 (m, 8H, H^{24}), 7.35 – 7.28 (m, 2H, H^{20}). ^{31}P NMR (202 MHz, $CDCl_3$) δ: 34.27. $^{13}C\{^1H\}$ NMR (126 MHz, $CDCl_3$) δ: 137.0 (t, $^{2}J_{13C\text{-}31P}$ = 7.0 Hz, C^{20}), 134.8 (t, $^{2}J_{13C\text{-}31P}$ = 7.0 Hz, C^{23}), 132.4 (C^{11}), 131.7 (C^{25}), 131.4 (C^{21}), 131.32 (C^{14}), 131.27 (C^{13}), 130.3 (C^2), 130.1 (C^q), 129.9 (C^q), 129.7 (C^{12}), 129.2 (t, $^{3}J_{13C\text{-}31P}$ = 5.7 Hz, C^{24}), 127.4 (C^{10}), 127.3 (C^4), 127.2 (C^9), 126.8 (C^5), 125.5 (C^7), 124.7 (C^8), 124.5 (C^{15} + C^{16}), 124.4 (C^6), 124.2 (C^3), 121.4 (C^1), 103.7 (C^{17}, *in*). HRMS (ESI-TOF): m/z = 1313.1978 $[M + Na]^+$, calculated 1313.1985 for $C_{66}H_{42}Au_2NaP_2$. m/z = 1329.1723 $[M + K]^+$, calculated 1329.1725 for $C_{66}H_{42}Au_2KP_2$.

PAuxantphos. Method A was followed using 1-[(trimethylsilyl)ethynyl]pyrene and $[(AuCl)_2xantphos]$. Isolated as a yellow solid (57 mg, 80% yield). ^{1}H NMR (500 MHz, $CDCl_3$) δ: 9.07 (d, *J* = 9.0 Hz, 2H, H^{10}), 8.16 (d, *J* = 7.8 Hz, 2H, H^2), 8.08 (d, *J* = 7.4 Hz, 2H, H^6), 8.00 (d, *J* = 7.8 Hz, 4H, H^3 + H^8), 7.97 (s, 2H, H^4 + H^5), 7.91 (t, *J* = 7.4 Hz, 2H, H^7), 7.71 (d, *J* = 9.0 Hz, 2H, H^9), 7.63 (d, *J* = 7.46 Hz, 2H, H^{22}), 7.49 (br, 4H, H^{30}), 7.37 (br, 8H, H^{28}), 7.23 (br s, 8H, H^{29}), 7.08 (t, *J* = 7.4 Hz, 2H, H^{21}), 6.53 (dd, 2H, H^{20}), 1.70 (s, 6H, H^{26}). ^{31}P NMR (162 MHz, $CDCl_3$) δ: 32.09. $^{13}C\{^1H\}$ NMR (126 MHz, $CDCl_3$) δ: 153.2 (C^{24}, *in*), 134.7 (d, *J* = 14.3 Hz, C^{30}), 133.0 (C^{20}), 132.5 (C^{11}) 131.6 (C^{23}, *in*), 131.4 (C^{13} + C^{14}), 131.0 (C^{28}), 130.7 (C^{27}, *in*), 130.2 (C^2), 129.6 (C^{12}), 128.9 (C^{22}), 128.8 (C^{29}), 127.7 (C^{10}), 127.4 (C^4), 127.2 (C^9), 126.7 (C^5), 125.6 (C^7), 124.6 - 123.9 (C^3 + C^6 + C^8 + C^{15} + C^{16} + C^{21}), 121.9 (C^1), 118.1 (C^{19}, *in*), 104.1 (C^{17}, *in*) 34.8 (C^{25}, *in*), 31.1 (C^{26}). HRMS (ESI-TOF): m/z = 1197.2201 [M - C≡C-pyr]$^+$, calculated 1197.1964 for $C_{57}H_{41}Au_2OP_2$.

CAuPPh₃. Method A was followed using 1-[(trimethylsilyl)ethynyl]corannulene and $[AuCl(PPh_3)]$. Isolated as a yellow solid (24 mg, 66% yield). ^{1}H NMR (500 MHz, $CDCl_3$) δ: 8.26 (d, *J* = 8.7 Hz, 1H, H^{10}), 8.00 (s, 1H, H^2), 7.82 (d, *J* = 8.7 Hz, 1H, H^9), 7.79 (s, 2H, H^7 + H^8), 7.77 (s, 2H, H^5 + H^6), 7.76 (d, *J* = 8.7 Hz, 1H, H^4), 7.71 (d, *J* = 8.7 Hz, 1H, H^3), 7.64 – 7.57 (m, 6H, H^{24}), 7.56 – 7.44 (m, 9H, H^{26} + H^{25}). ^{31}P NMR (162 MHz, $CDCl_3$) δ: 42.22. $^{13}C\{^1H\}$ NMR (101 MHz, $CDCl_3$) δ: 136.2 (C^{18}), 135.8 (C^{17}), 135.7 (C^{16}), 135.1 (C^{20}), 134.6 (C^{19}), 134.4 (d, $^{2}J_{13C\text{-}31P}$ = 13.8 Hz, C^{24}), 132.0 (C^{11}), 131.6 (d, $^{4}J_{13C\text{-}31P}$ = 1.9 Hz, C^{26}), 131.1 (C^2), 131.0 (C^{15}), 130.9 (C^{14}), 130.8 (C^{13}), 130.7 (C^{12}), 129.8 (d, $^{1}J_{13C\text{-}31P}$

= 55.8 Hz, C^{23}), 129.2 (d, $^3J_{13C-31P}$ = 11.3 Hz, C^{25}), 127.2 – 126.8 (m, C^4 – C^{10}), 126.7 (C^3), 123.5 (C^1). HRMS (MALDI-TOF): m/z = 732.1286 $[M]^+$, calculated 732.1276 for $C_{40}H_{24}AuP$.

CAudppe. Method B was followed using 1-(ethynyl)corannulene and $[(AuCl)_2dppe]$. Isolated as a yellow solid (24 mg, 91% yield). ^1H NMR (500 MHz, $CDCl_3$) δ: 8.27 (d, *J* = 8.8 Hz, 2H, H^{10}), 8.02 (s, 2H, H^2), 7.82 (d, J = 8.8 Hz, 2H, H^9), 7.80 (s, 4H, H^7 + H^8), 7.78 (s, 4H, H^5 + H^6), 7.77 (d, *J* = 8.7 Hz, 2H, H^4), 7.76 – 7.73 (m, 8H, H^{25}), 7.72 (d, *J* = 8.7 Hz, 2H, H^3), 7.57 – 7.50 (m, 12H, H^{26} + H^{27}), 2.76 (br s, 4H, H^{23}). ^{31}P NMR (202 MHz, $CDCl_3$) δ: 39.86. $^{13}C\{^1H\}$ NMR (126 MHz, $CDCl_3$) δ: 136.2 (C^{18}), 135.8 (C^{17}), 135.7 (C^{16}), 135.1 (C^{20}), 134.7 (C^{19}), 133.5 (t, $^2J_{13C-31P}$ = 6.8 Hz, C^{25}), 132.2 (C^{27}), 131.9 (C^{11}), 131.1 (C^2), 131.0 (C^{15}), 130.82 (C^{12} + C^{12}), 130.79 (C^{13}), 129.6 (t, $^3J_{13C-31P}$ = 5.6 Hz, C^{26}), 128.8 (t, $^1J_{13C-31P}$ = 27.1 Hz, C^{24}), 127.3 – 126.8 (C^4 – C^{10}), 126.7 (C^3), 123.3 (C^1), 24.0 (C^{23}). HRMS (MALDI-TOF): m/z = 1338.2061 $[M]^+$, calculated 1338.2088 for $C_{70}H_{42}Au_2P_2$.

CAudppf. Method B was followed using 1-(ethynyl)corannulene and $[(AuCl)_2dppf]$. Isolated as an orange solid (28 mg, 92% yield). ^1H NMR (500 MHz, $CDCl_3$) δ: 8.30 (d, *J* = 8.8 Hz, 2H, H^{10}), 8.00 (s, 2H, H^2), 7.82 (d, *J* = 8.8 Hz, 2H, H^9), 7.79 (s, 4H, H^7 + H^8), 7.78 (s, 4H, H^5 + H^6), 7.77 (d, *J* = 8.7 Hz, 2H, H^4), 7.71 (d, *J* = 8.7 Hz, 2H, H^3), 7.64 – 7.56 (m, 8H, H^{27}), 7.49 – 7.39 (m, 12H, H^{28} + H^{29}), 4.81 (br s, 4H, H^{25}), 4.42 (br s, 4H, H^{24}). ^{31}P NMR (162 MHz, $CDCl_3$) δ: 36.66. $^{13}C\{^1H\}$ NMR (126 MHz, $CDCl_3$) δ: 136.2 (C^{18}), 135.8 (C^{17}), 135.7 (C^{16}), 135.1 (C^{20}), 134.6 (C^{19}), 133.7 (d, $^2J_{13C-31P}$ = 14.1 Hz, C^{27}), 132.0 (C^{11}), 131.5 (br s, C^{29}), 131.3 (d, $^1J_{13C-31P}$ = 53.2 Hz, C^{26}), 131.0 (C^{15}), 130.93 (C^2), 130.87 (C^{14}), 130.8 (C^{13}), 130.7 (C^{12}), 129.0 (d, $^3J_{13C-31P}$ = 11.4 Hz, C^{28}), 127.1 – 126.9 (C^4 – C^{10}), 126.7 (C^3), 75.1 (d, $^2J1_{3C-31P}$ = 2,7 Hz, C^{24}), 75.0 (C^{25}) 72.1 (C^{23}, $^1J_{13C-31P}$ = 67.2 Hz, *in*). HRMS (ESI-TOF): m/z = 1517.1690 [M + Na]$^+$, calculated 1517.1650 for $C_{78}H_{46}Au_2FeNaP_2$. m/z = 1533.1451 [M + Na]$^+$, calculated 1533.1390 for $C_{78}H_{46}Au_2FeKP_2$.

CAudppbenz. Method B was followed using 1-(ethynyl)corannulene and $[(AuCl)_2dppbenz]$. Isolated as a yellow solid (24 mg, 86% yield). ^1H NMR (500 MHz, $CDCl_3$) δ: 8.23 (d, *J* = 8.7 Hz, 2H, H^{10}), 7.89 (s, 2H, H^2), 7.70 (d, *J* = 8.8 Hz, 2H, H^6), 7.68 (d, *J* = 8.8 Hz, 2H, H^5), 7.60 (d, *J* = 8.8 Hz, 4H, H^4 + H^7), 7.58 – 7.54 (m, 8H, H^{27}), 7.53 – 7.47 (m, 6H, H^{26} + H^{30}), 7.46 (d, *J* = 8.8 Hz, 2H, H^3), 7.41 (m, 8H, H^{28}), 7.35 (d, *J* = 8.8 Hz, 2H, H^8), 7.32 – 7.27 (m, 2H, H^{24}), 7.18 (d, *J* = 8.7 Hz, 2H, H^9). ^{31}P NMR (162 MHz, $CDCl_3$) δ: 34.30. $^{13}C\{^1H\}$ NMR (101 MHz, $CDCl_3$) δ: 137.0 (t, $^2J_{13C-31P}$ = 6.8 Hz, C^{24}), 135.9 (C^{19}), 135.7 (C^{20}), 135.3 (C^{16}), 134.9 (C^{17}), 134.8 (t, $^2J_{13C-31P}$ = 7.1 Hz, C^{27}), 134.2 (C^{18}), 132.3 (C^{11}), 131.7 (C^{29}), 131.3 (t, $^3J_{13C-31P}$ = 2.2 Hz, C^{25}), 130.9 (C^{12}), 130.7 (C^{15}), 130.5 (C^{14}), 130.4 (C^{13}), 130.2 (C^2) 129.8 (d, $^1J_{13C-31P}$ = 27.5 Hz, C^{26}), 129.2 (t, $^3J_{13C-31P}$ = 5.7 Hz, C^{28}), 127.6 (C^{10}), 126.9 (C^8), 126.71 (C^5), 126.67 (C^4), 126.65 (C^9), 126.60 (C^6), 126.58 (C^3), 126.5 (C^7), 124.6 (C^1), 103.0 (C^{21}). HRMS (ESI-TOF): m/z = 1409.1985 [M + Na]$^+$, calculated 1409.1985 for $C_{74}H_{42}Au_2NaP_2$.

CAuxantphos. Method B was followed using 1-(ethynyl)corannulene and $[(AuCl)_2xantphos]$. Isolated as a yellow solid (23 mg, 76% yield). ^1H NMR (500 MHz, $CDCl_3$) δ: 8.34 (d, *J* = 8.6 Hz, 2H, H^{10}), 7.91 (s, 2H, H^2), 7.74 (s, 8H, $4H^{cor}$), 7.70 (d, *J* = 9.0 Hz, 2H, H^{cor}), 7.62 (m, 4H, H^{cor} + H^{26}), 7.42 (s, 8H, H^{33}), 7.37 – 7.31 (m, 6H, H^9 + H^{34}), 7.20 (br t, 8H, H^{32}), 7.07 (t, *J* = 7.7 Hz, 2H, H^{25}), 6.53 – 6.47 (m, 2H, H^{24}), 1.70 (s, 6H, H^{30}). ^{31}P NMR (162 MHz, $CDCl_3$) δ: 31.92. $^{13}C\{^1H\}$ NMR (126 MHz, $CDCl_3$) δ: 153.0 (C^{28}, *in*), 136.1 (C^q), 135.8 (C^q), 135.6 (C^{16}), 135.1 (C^{17}), 134.6 (d, $^3J_{13C-31P}$ = 12.6 Hz, C^{33}), 134.3 (C^{18}), 133.0 (br s, C^{24}), 132.7 (br s, C^{11}), 131.6 (br s, C^{27}), 131.2 (C^q), 131.0 (C^{34}), 130.9 (C^{15}), 130.6 (C^q), 130.5 (C^q), 129.9 (C^2), 128.9 (C^{32}), 128.8 (C^{26}), 128.0 (C^{10}), 127.0 – 126.5 (C^3 + C^4 + C^5 + C^6 + C^7 + C^8 + C^9), 125.1 (C^1, *in*), 124.2 (C^{25}), 118.0 (C^{23}, *in*), 103.6 (C^{21}, *in*), 34.7 (C^{29}, *in*), 31.2 (C^{30}). HRMS (ESI-TOF): m/z = 1245.2411 [M - C≡C-cor]$^+$, calculated 1245.1946 for $C_{61}H_{41}Au_2OP_2$.

p-tolylAuxantphos. Method B was followed using 1-ethynyl-4methylbenzene and [$(AuCl)_2$xantphos]. Isolated as a white solid (20 mg, 85% yield). ^{1}H NMR (500 MHz, $CDCl_3$) δ: 7.56 (d, *J* = 7.6 Hz, 2H, H^{11}), 7.45 – 7.35 (m, 12H, H^{18} + H^{19}), 7.32 (d, *J* = 7.5 Hz, 2H, H^{2}), 7.27 – 7.19 (m, 8H, H^{17}), 7.04 (d, *J* = 7.6 Hz, 2H, H^{10}), 6.98 (d, *J* = 7.5 Hz, 2H, H^{3}), 6.49 (t, *J* = 7.6 Hz, 2H, H^{9}), 2.29 (s, 6H, H^{5}), 1.63 (s, 6H, H^{15}). ^{31}P NMR (202 MHz, $CDCl_3$) δ 32.23 ^{13}C (*in*) δ: 153.3 (C^{13}), 134.6 (C^{18} or C^{19}), 132.7 (C^{9}), 131.5 (C^{12}), 132.1 (C^{2}), 130.8 (C^{18} or C^{19}), 128.7 (C^{17}), 128.5 (C^{3}), 128.4 (C^{11}), 124.1 (C^{10}), 118.4 (C^{8}), 105.0 (C^{6}), 34.7 (C^{14}), 30.6 (C^{15}), 21.3 (C^{5}). HRMS (ESI-TOF): m/z = 1225.2219 $[M + Na]^+$, calculated 1225.2247 for $C_{57}H_{46}Au_2NaOP_2$.

Selected NMR spectra

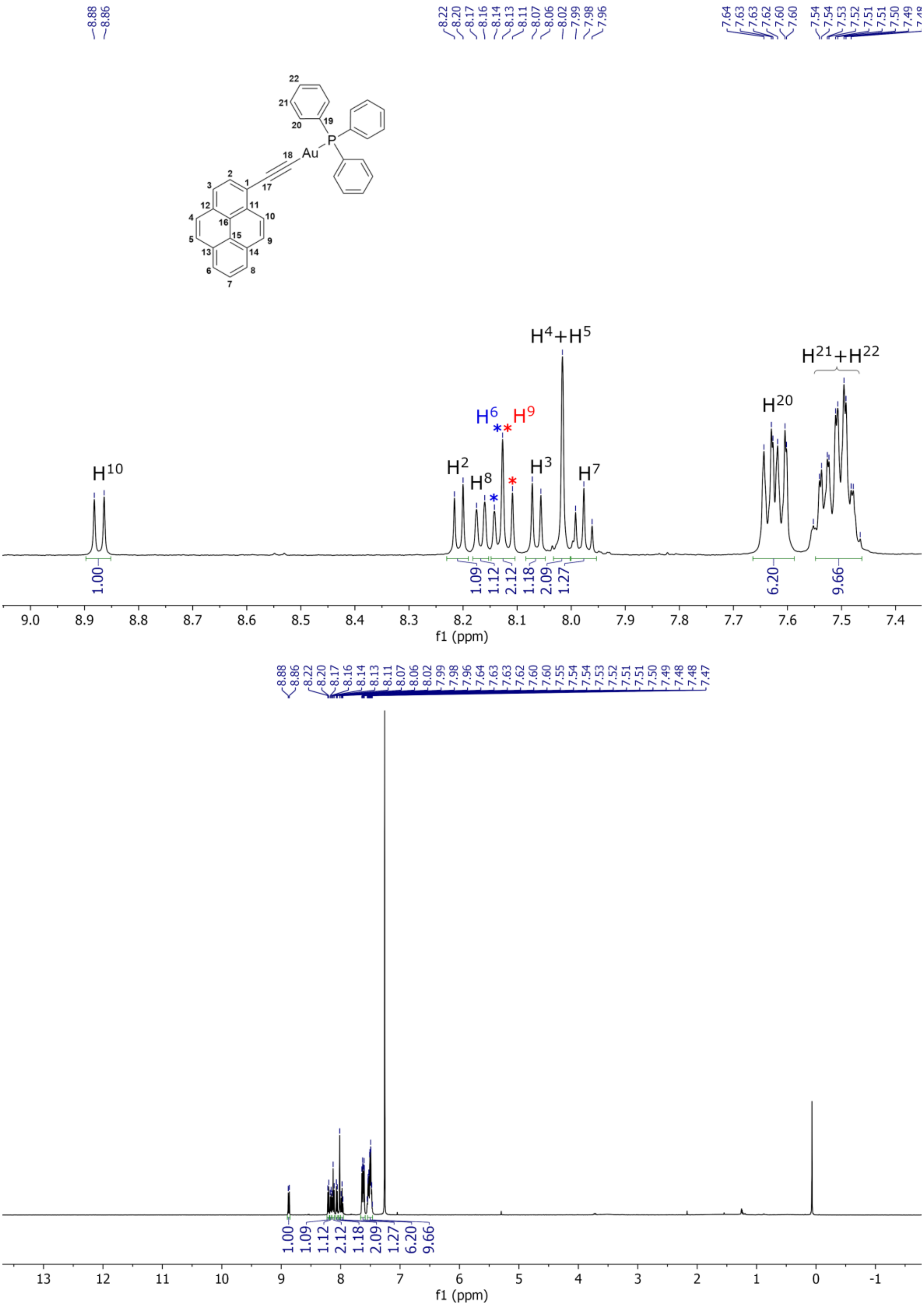

Figure S1. ^{1}H NMR spectrum of compound $PAuPPh_3$ (500 MHz, $CDCl_3$); selected regions (above) and full spectrum (below).

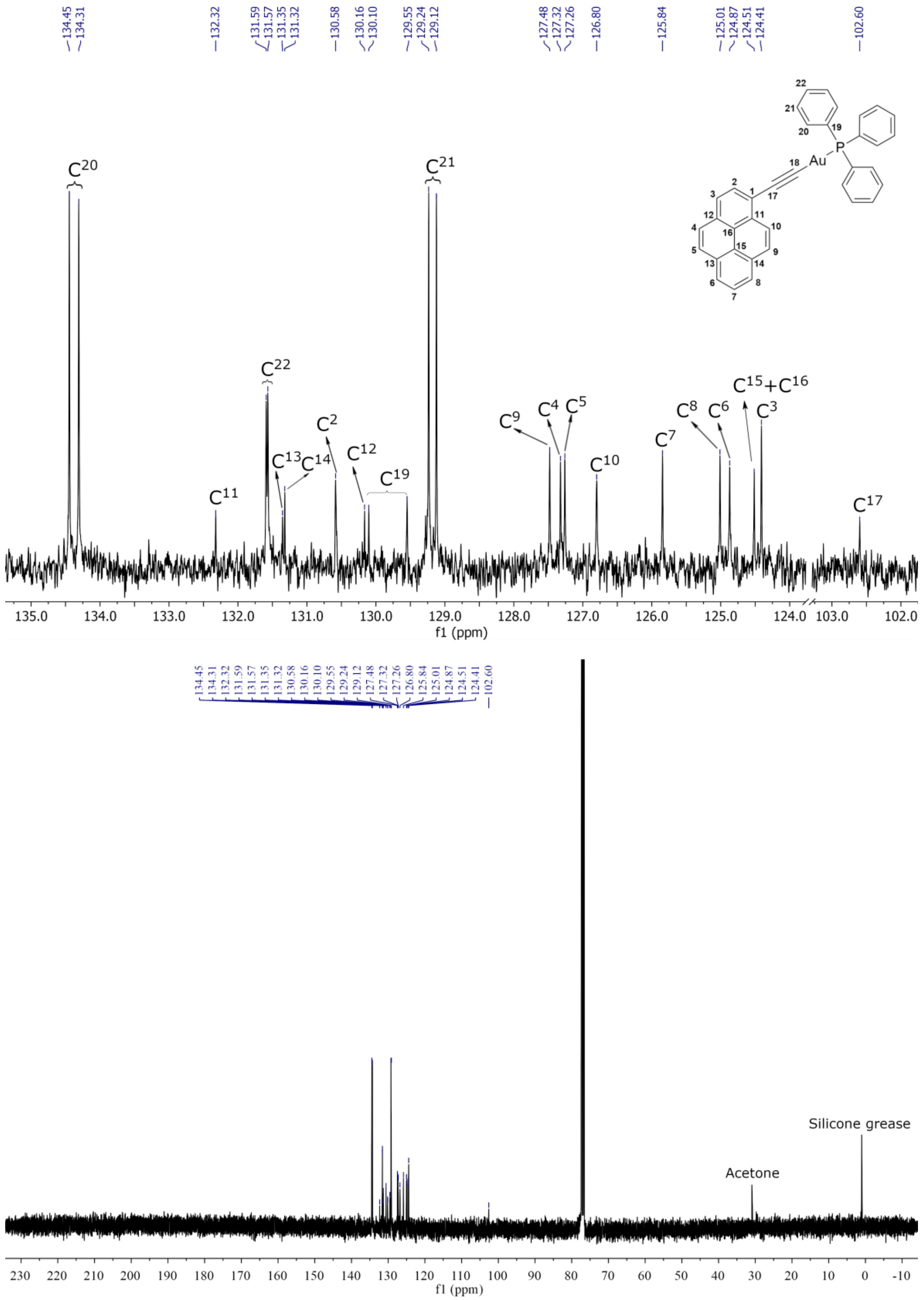

Figure S2. $^{13}C\{^1H\}$ NMR spectrum of compound PAuPPh₃ (101 MHz, $CDCl_3$); selected regions (above) and full spectrum (below).

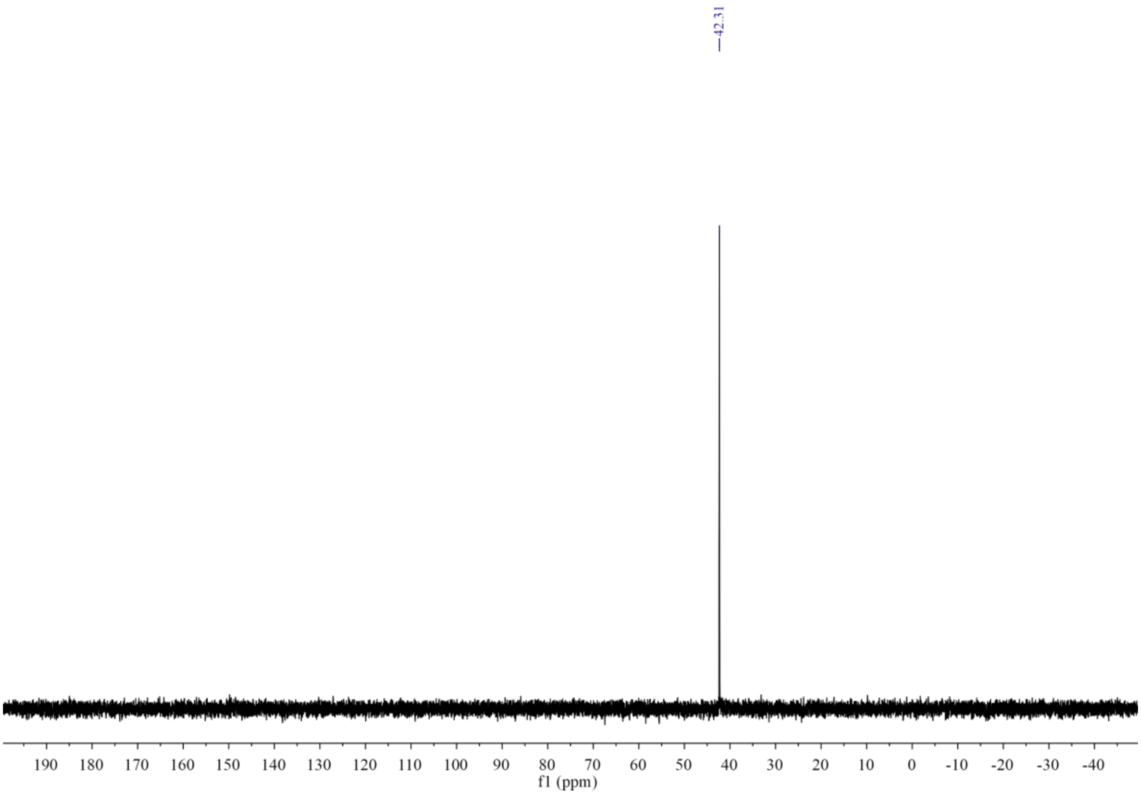

Figure S3. ^{31}P NMR spectrum of compound $PAuPPh_3$ (162 MHz, $CDCl_3$).

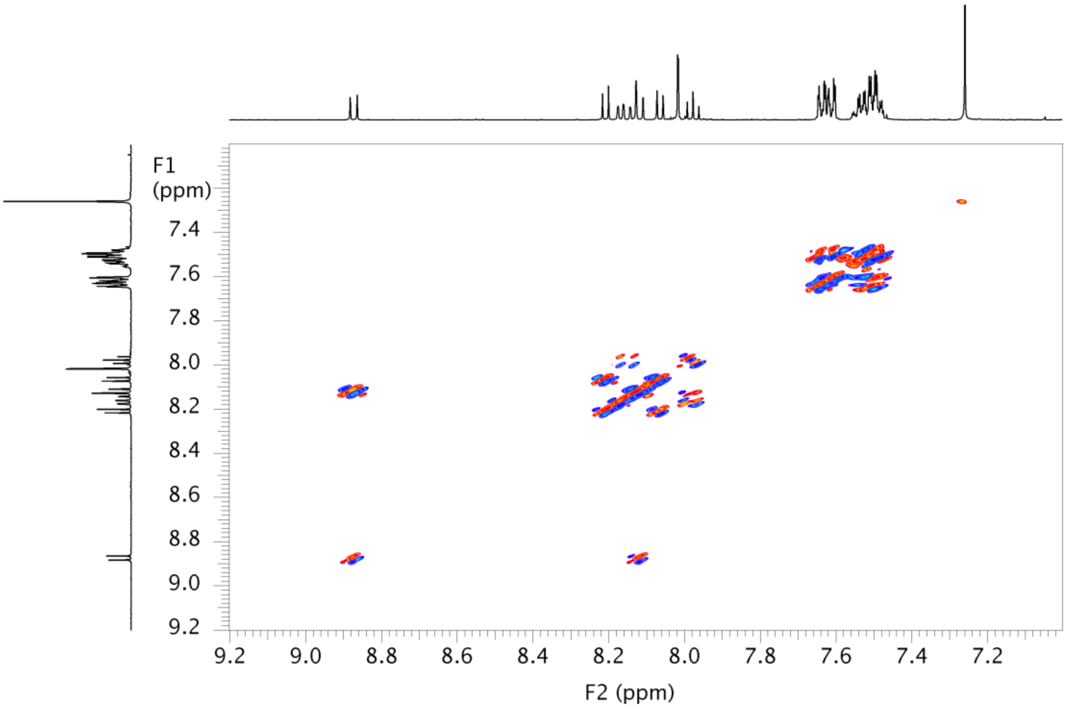

Figure S4. ^{1}H-^{1}H gDQFCOSY spectrum of compound $PAuPPh_3$ (500 MHz, $CDCl_3$).

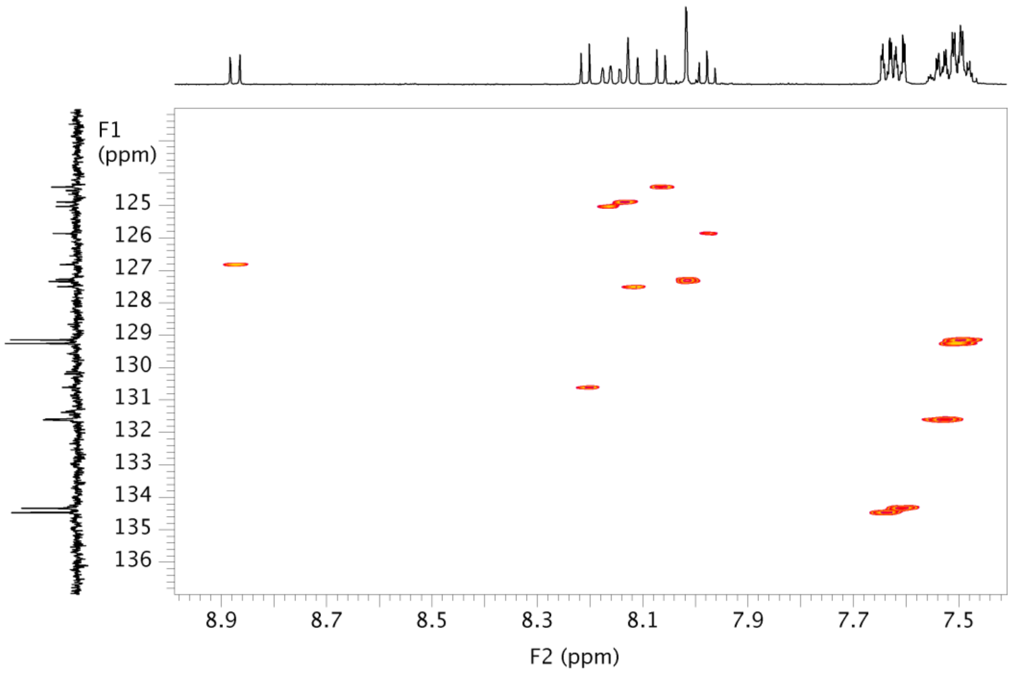

Figure S5. ^{1}H-^{13}C gHSQCAD spectrum of compound $PAuPPh_3$ (500 MHz, $CDCl_3$).

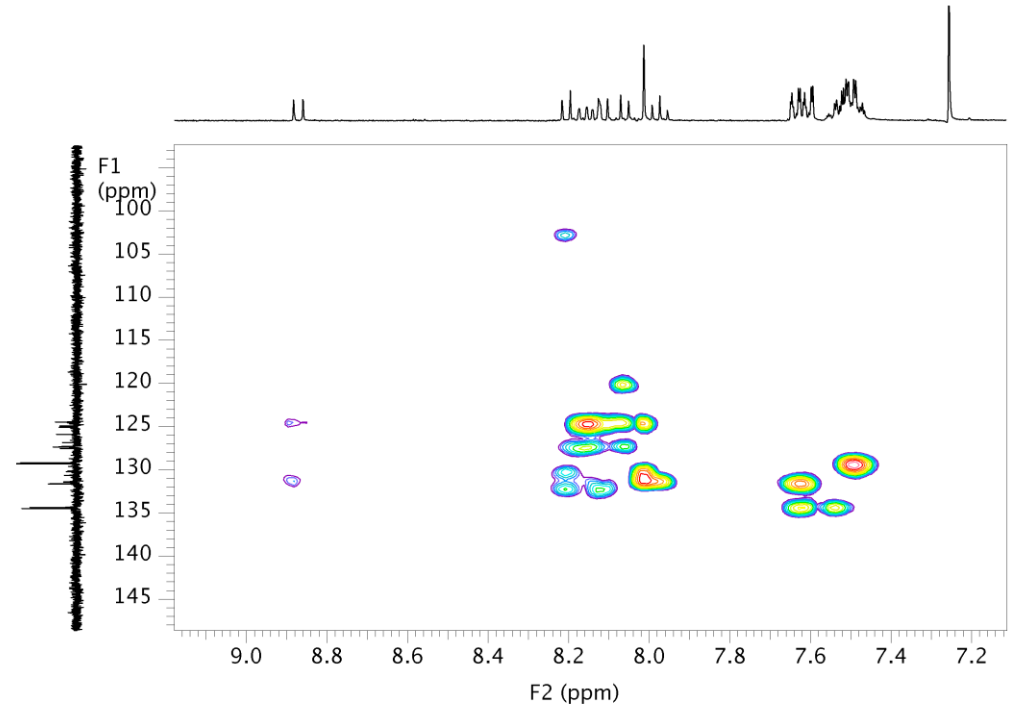

Figure S6. ^{1}H-^{13}C gHMBCAD spectrum of compound $PAuPPh_3$ (500 MHz, $CDCl_3$).

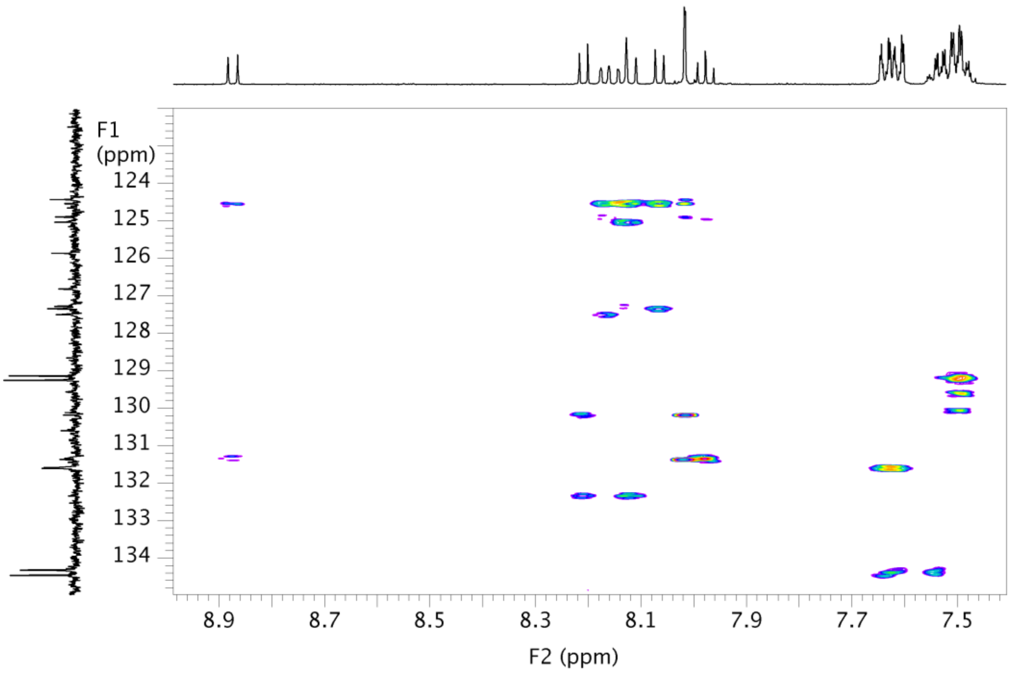

Figure S7. ^{1}H-^{13}C bsgHMBCAD spectrum of compound $PAuPPh_3$ (500 MHz, $CDCl_3$).

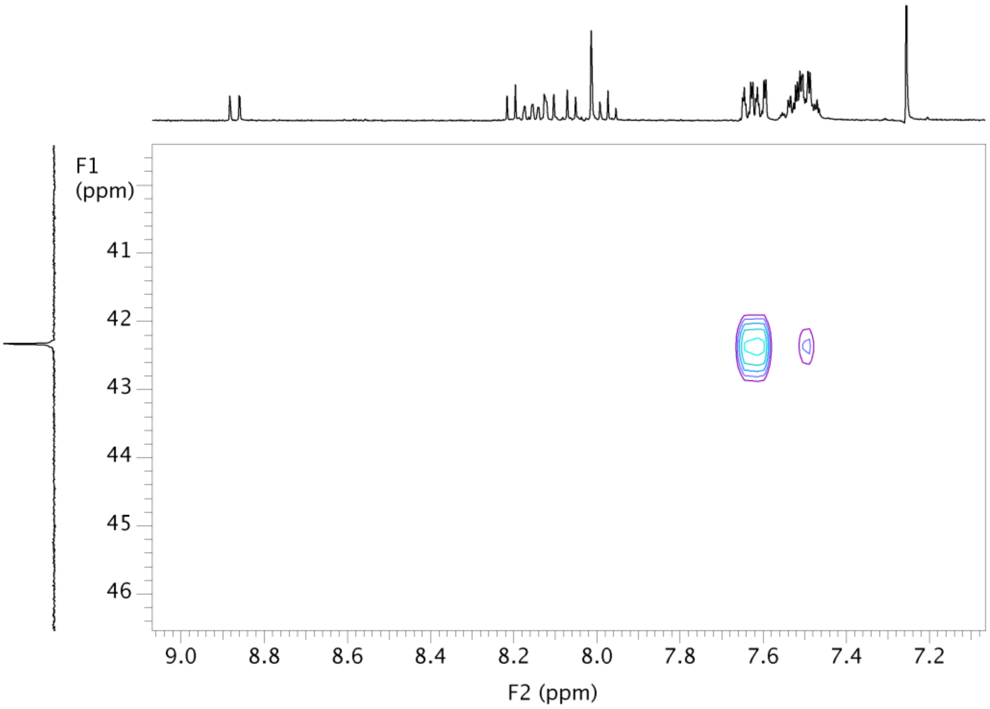

Figure S8. ^{1}H-^{31}P gHMBCAD spectrum of compound $PAuPPh_3$ (500 MHz, $CDCl_3$).

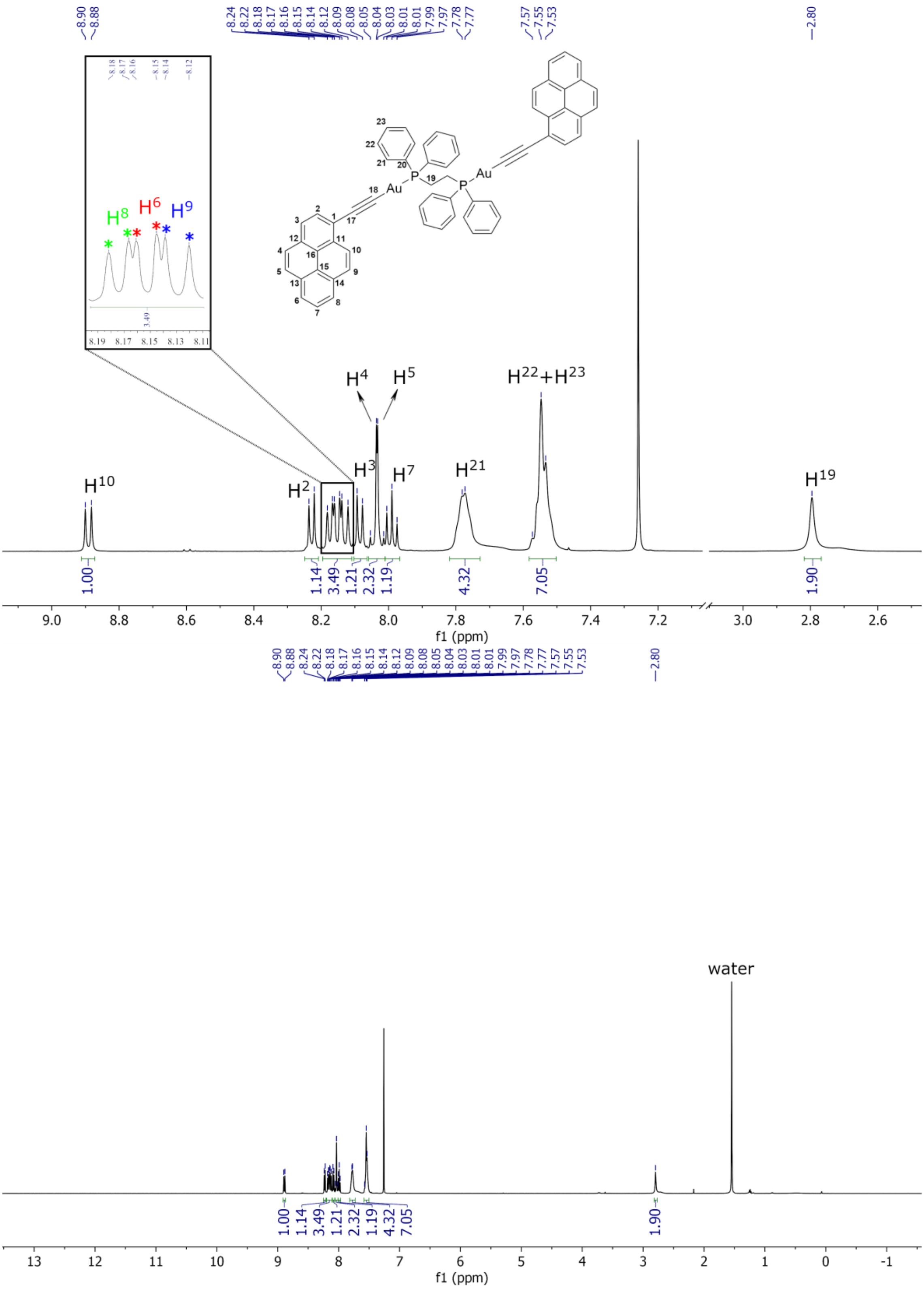

Figure S9. ^{1}H NMR spectrum of compound PAudppe (500 MHz, $CDCl_3$); selected regions (above) and full spectrum (below). Some signals are depicted in different colors for clarity purposes.

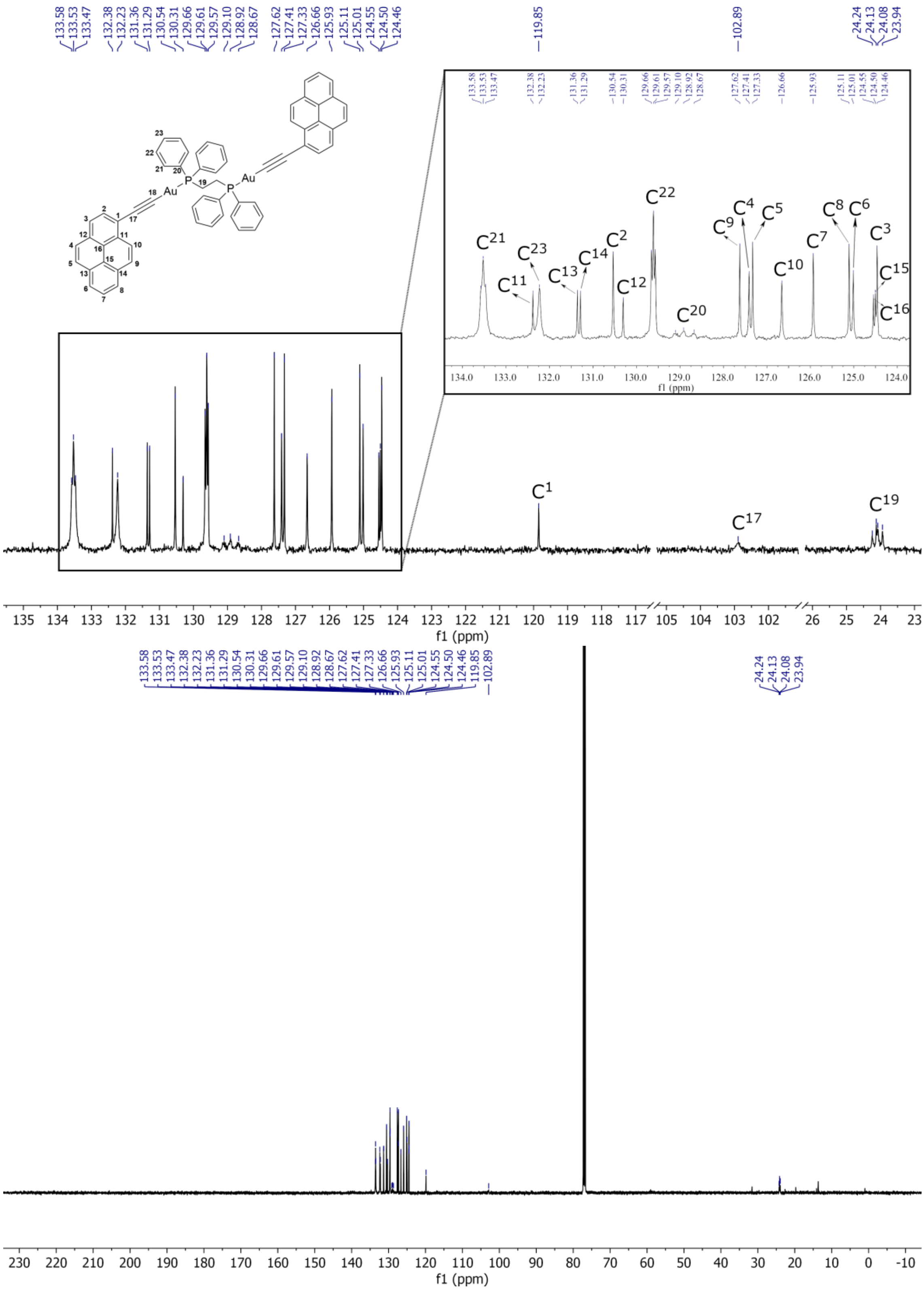

Figure S10. $^{13}C\{^{1}H\}$ NMR spectrum of compound PAudppe (126 MHz, $CDCl_3$); selected regions (above) and full spectrum (below).

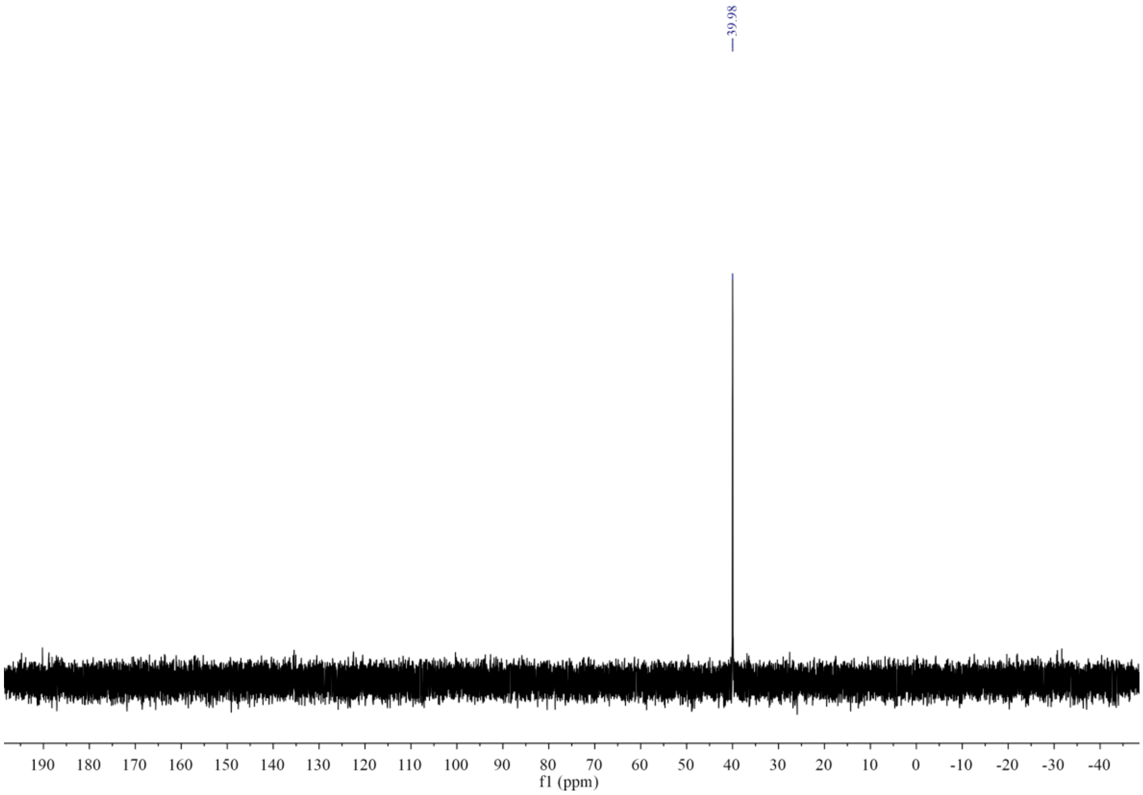

Figure S11. ^{31}P NMR spectrum of compound PAudppe (202 MHz, $CDCl_3$).

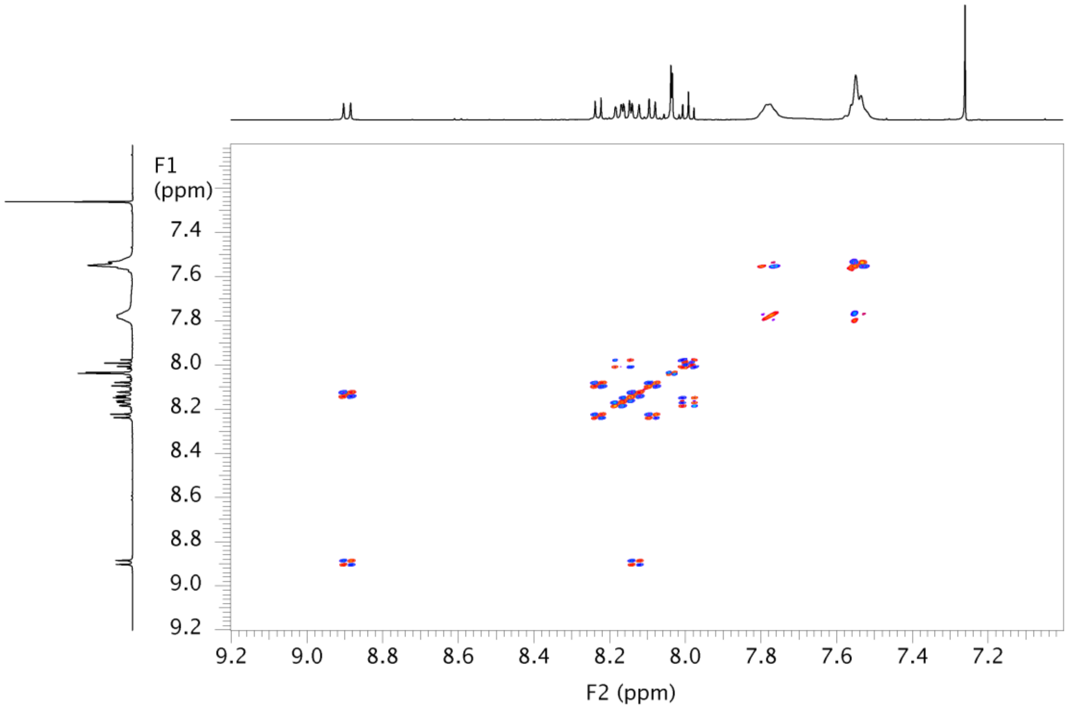

Figure S12. ^{1}H-^{1}H gDQFCOSY spectrum of compound PAudppe (500 MHz, $CDCl_3$).

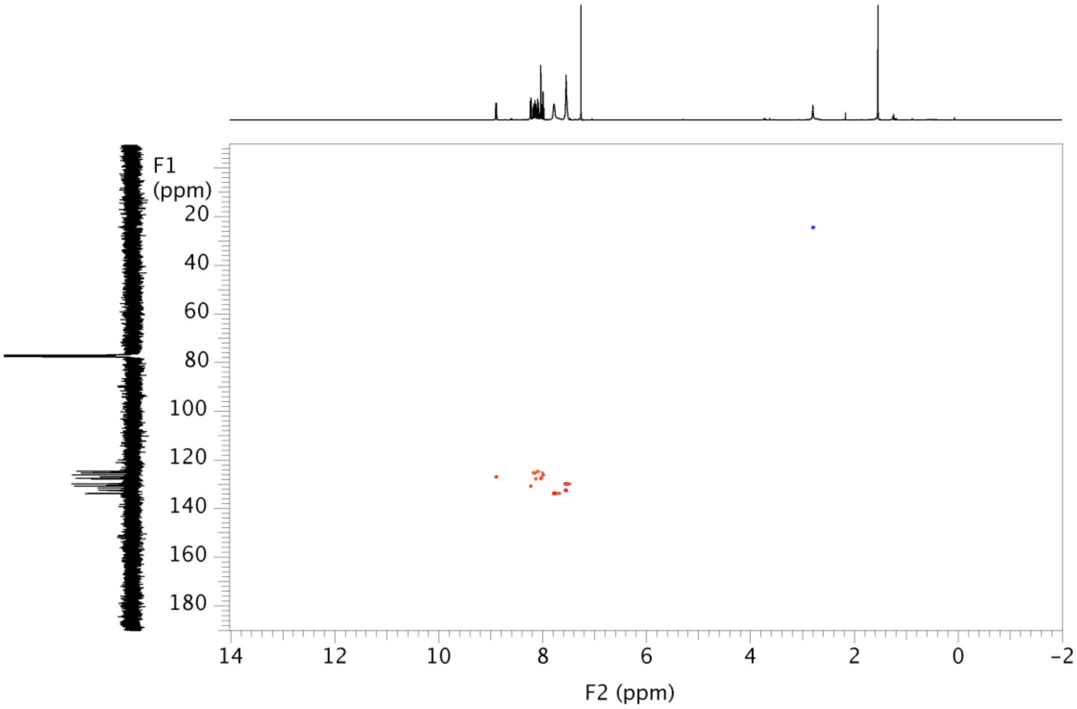

Figure S13. ^{1}H-^{13}C g2cHSQC spectrum of compound PAudppe (500 MHz, $CDCl_3$).

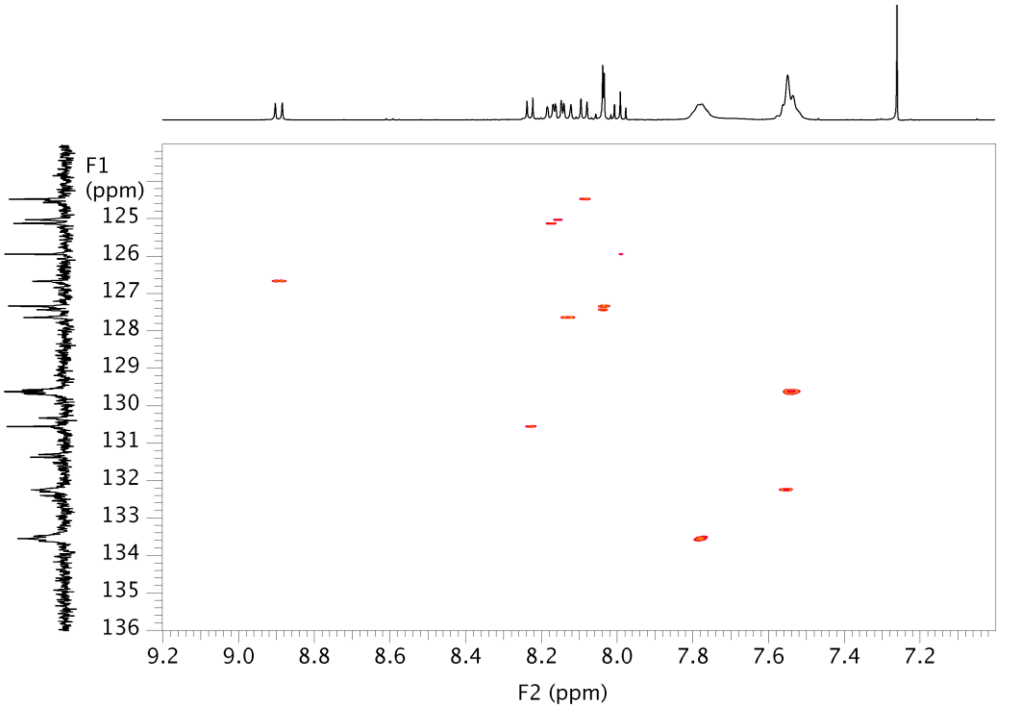

Figure S14. ^{1}H-^{13} bsgHSQCAD spectrum of compound PAudppe (500 MHz, $CDCl_3$).

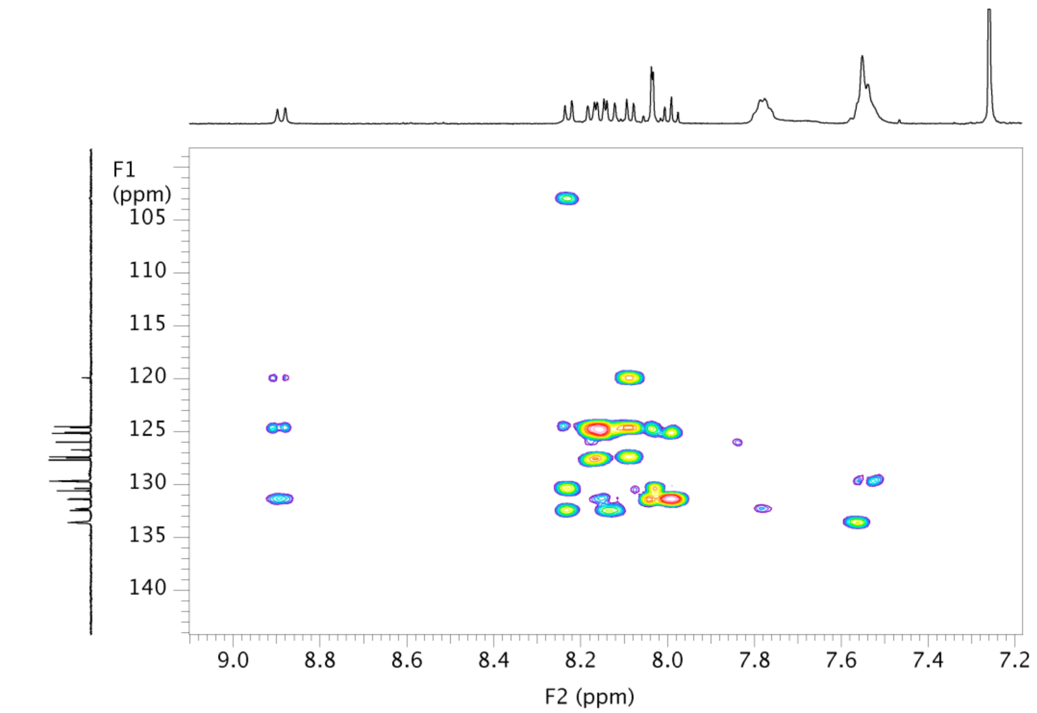

Figure S15. ^{1}H-^{13}C g2cHMBC spectrum of compound PAudppe (500 MHz, $CDCl_3$).

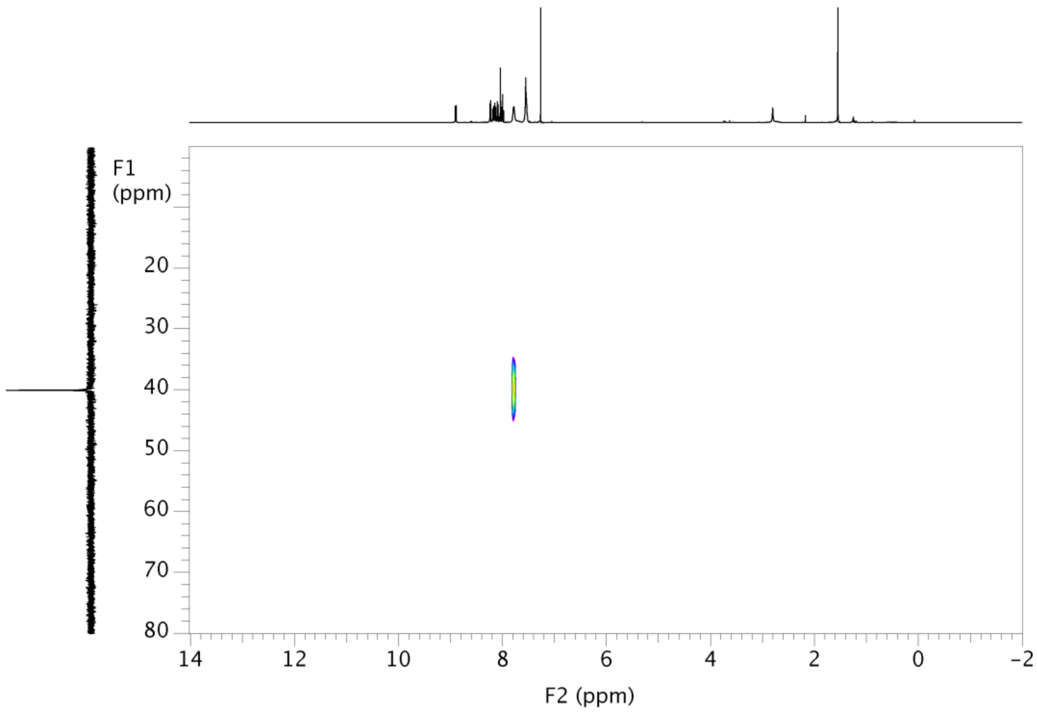

Figure S16. ^{1}H-^{31}P gHMBCAD spectrum of compound PAudppe (500 MHz, $CDCl_3$).

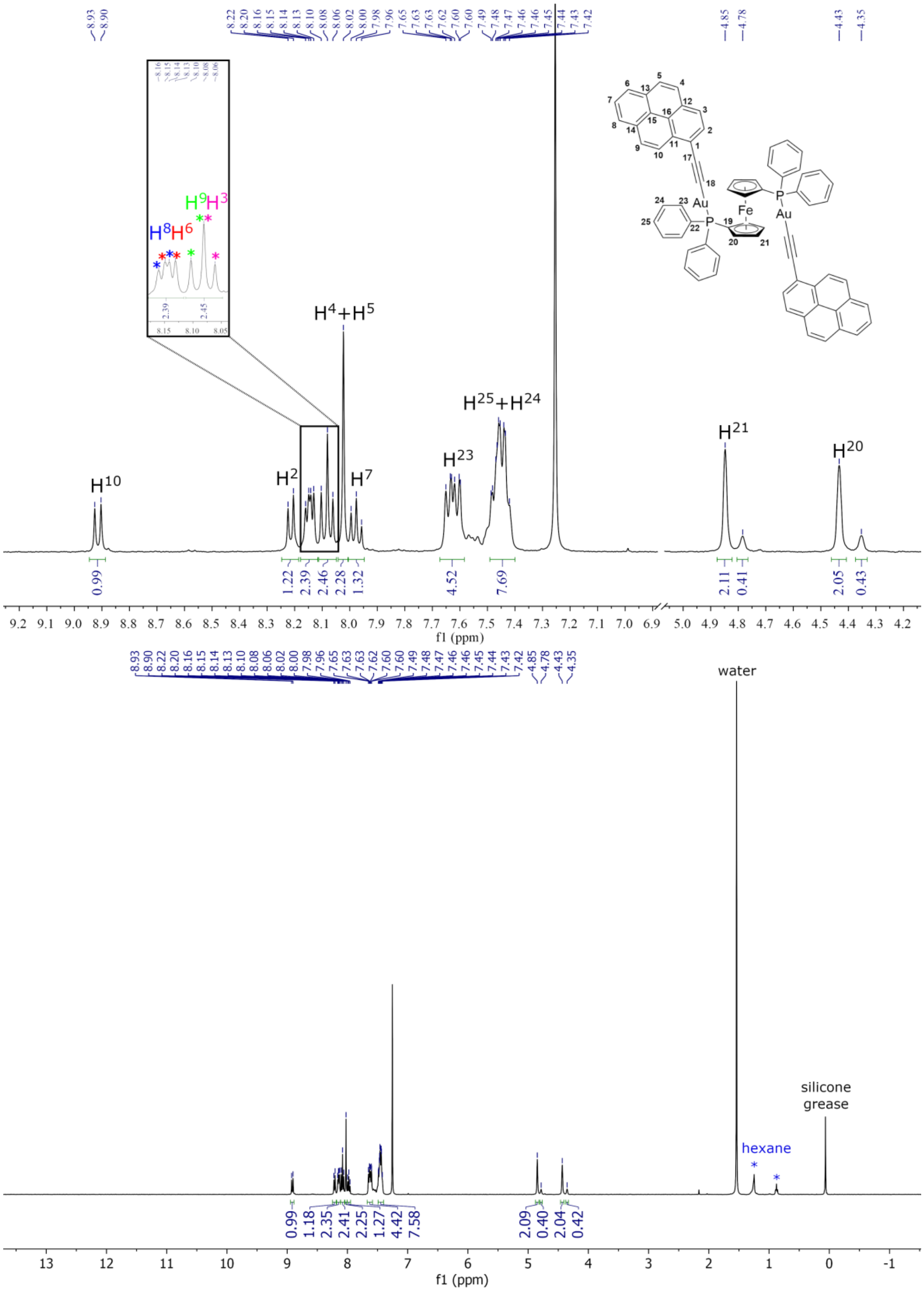

Figure S17. ^{1}H NMR spectrum of compound PAudppf (400 MHz, $CDCl_3$); selected regions (above) and full spectrum (below). Some signals are depicted in different colors for clarity purposes.

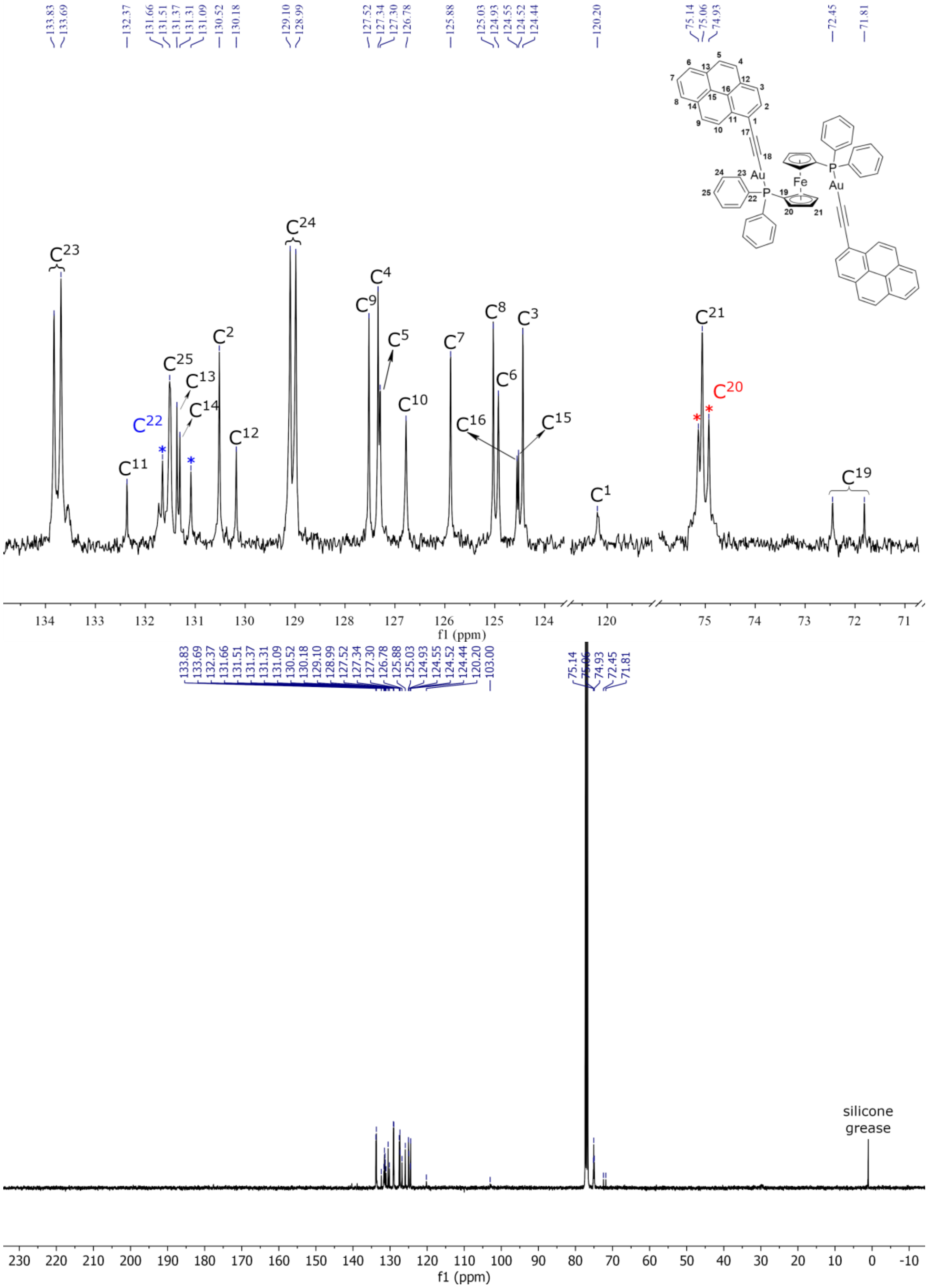

Figure S18. $^{13}C\{^{1}H\}$ NMR spectrum of compound PAudppf (101 MHz, $CDCl_3$); selected regions (above) and full spectrum (below). Some signals are depicted in different colors for clarity purposes.

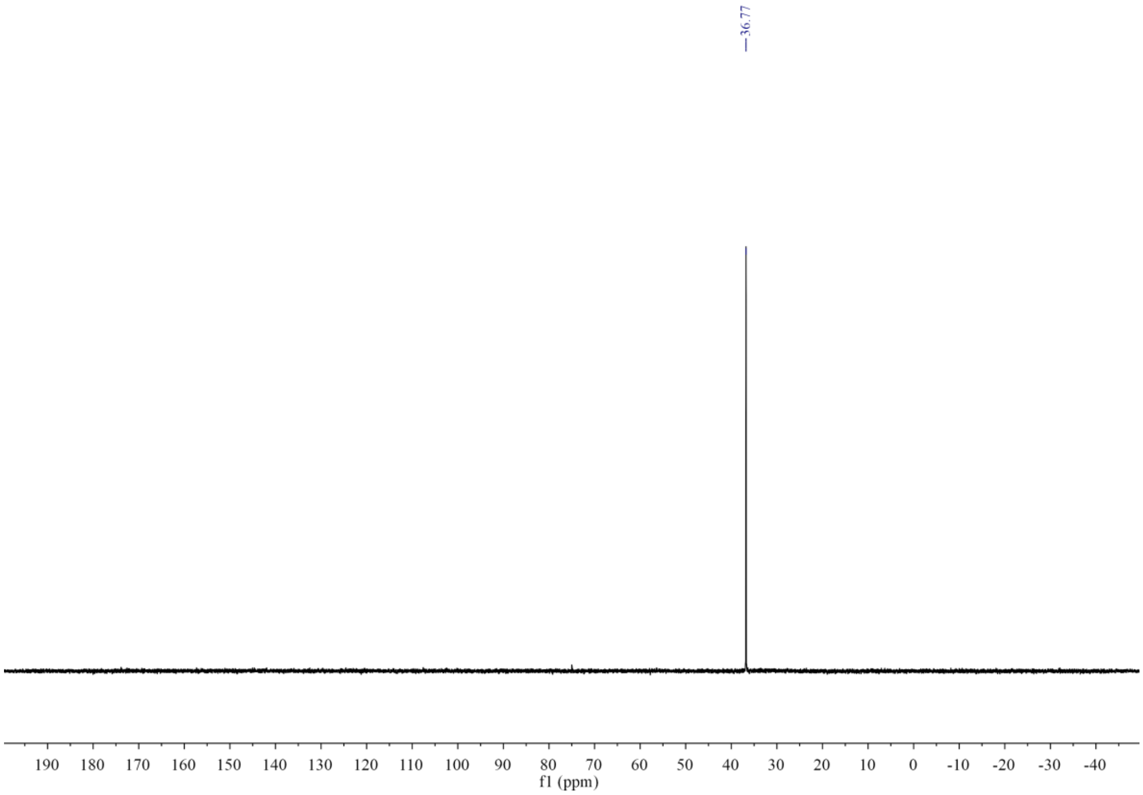

Figure S19. ^{31}P NMR spectrum of compound PAudppf (162 MHz, $CDCl_3$).

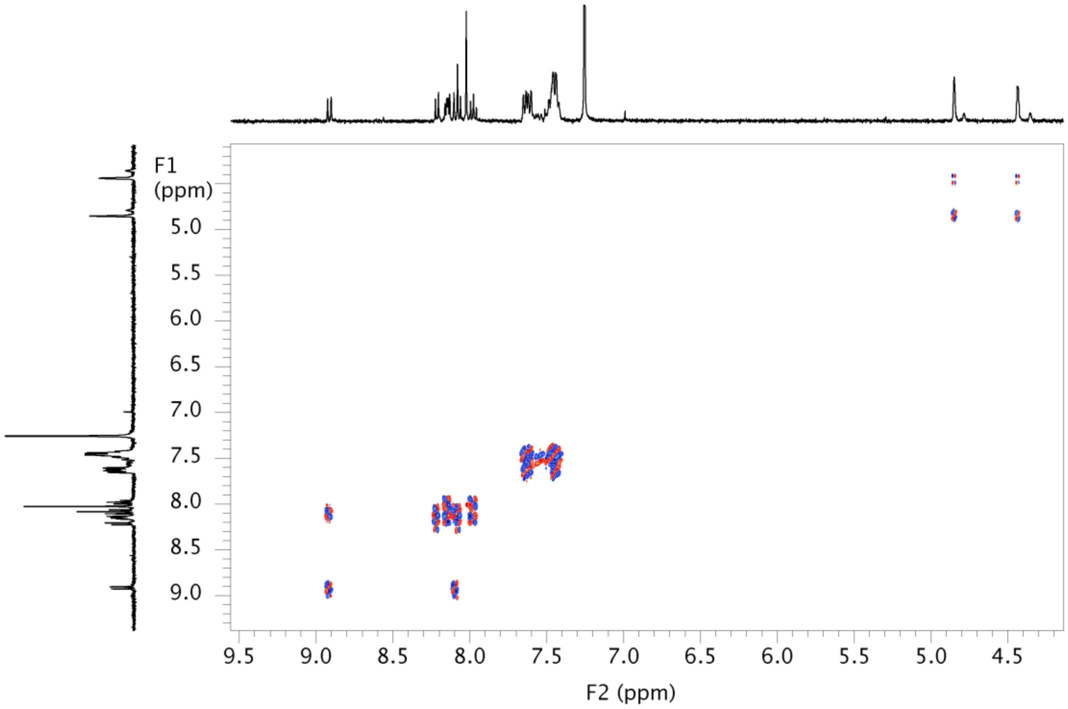

Figure S20. ^{1}H-^{1}H gDQFCOSY spectrum of compound PAudppf (500 MHz, $CDCl_3$).

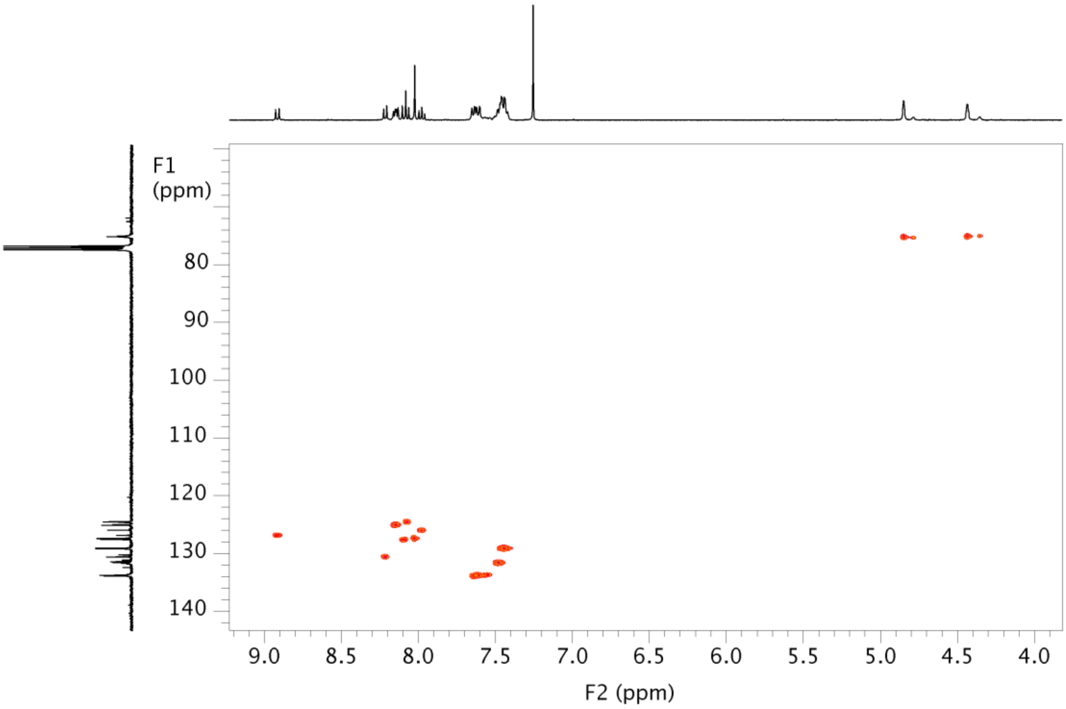

Figure S21. ^{1}H-^{13}C g2cHSQC spectrum of compound PAudppf (500 MHz, $CDCl_3$).

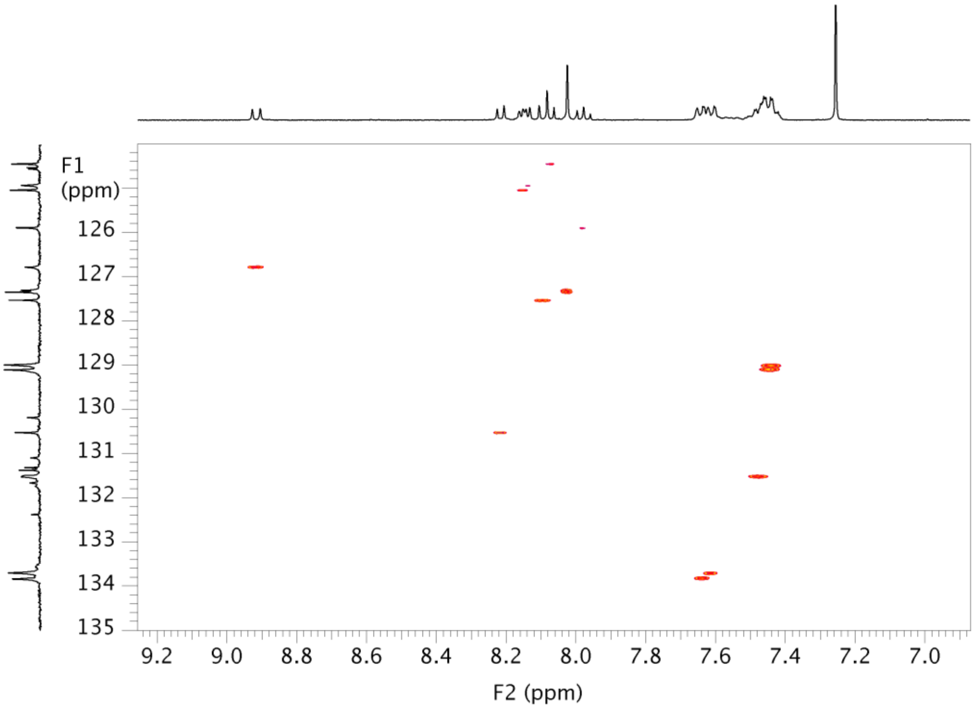

Figure S22. ^{1}H-^{13}C bsgHSQCAD spectrum of compound PAudppf (500 MHz, $CDCl_3$).

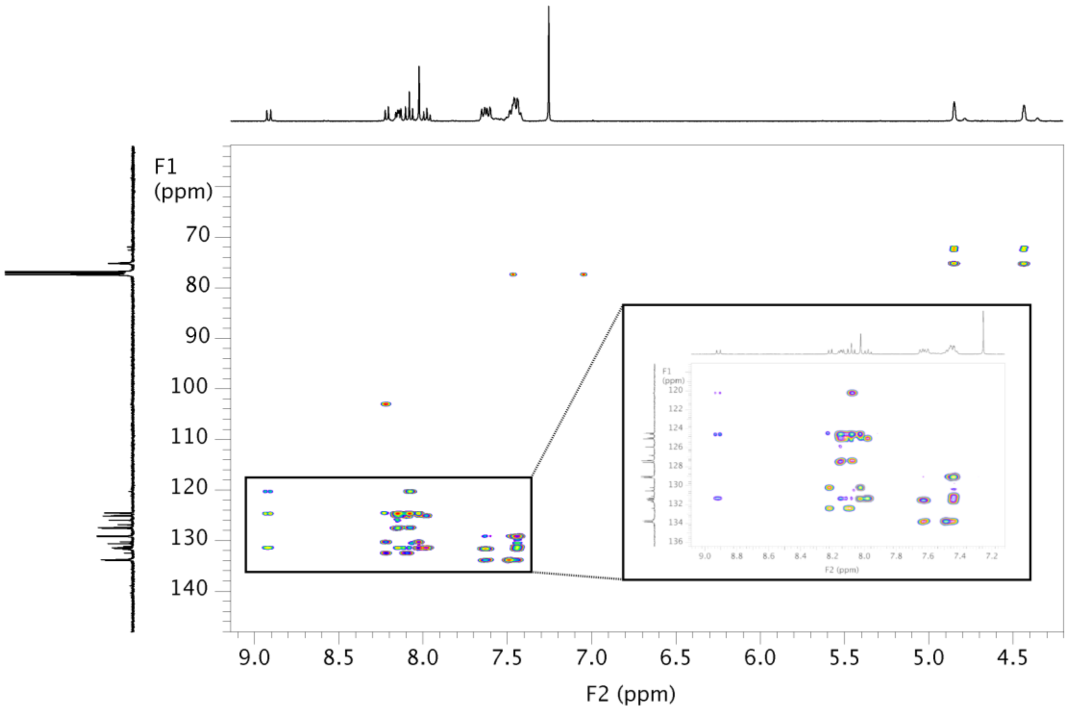

Figure S23. ^{1}H-^{13}C g2cHMBC spectrum of compound PAudppf (500 MHz, $CDCl_3$).

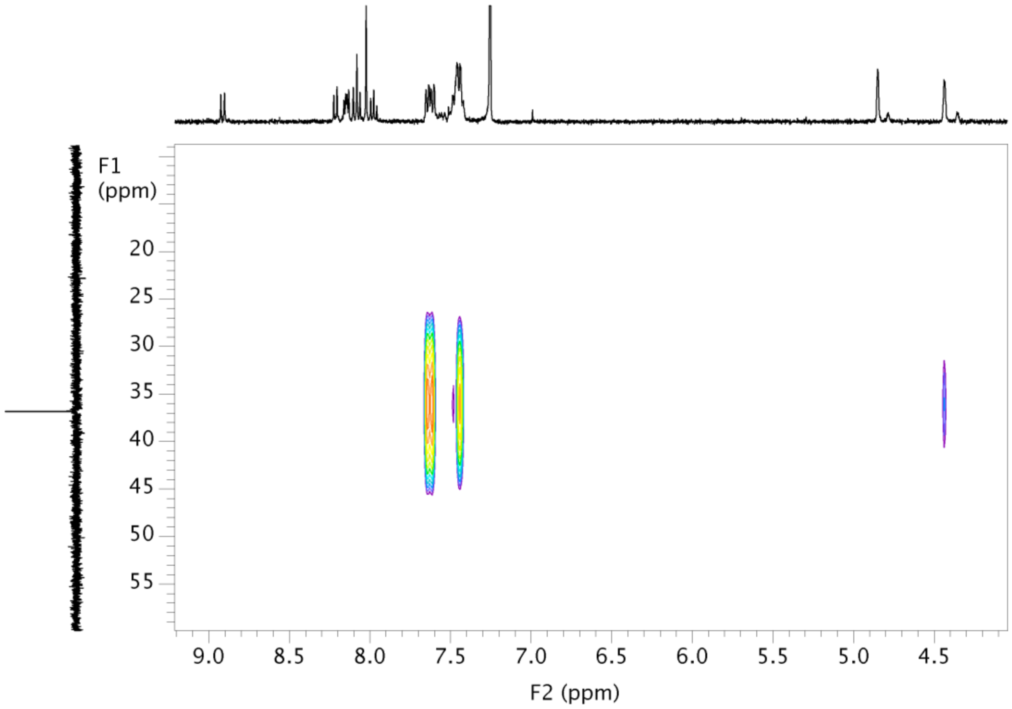

Figure S24. ^{1}H-^{31}P gHMBCAD spectrum of compound PAudppf (500 MHz, $CDCl_3$).

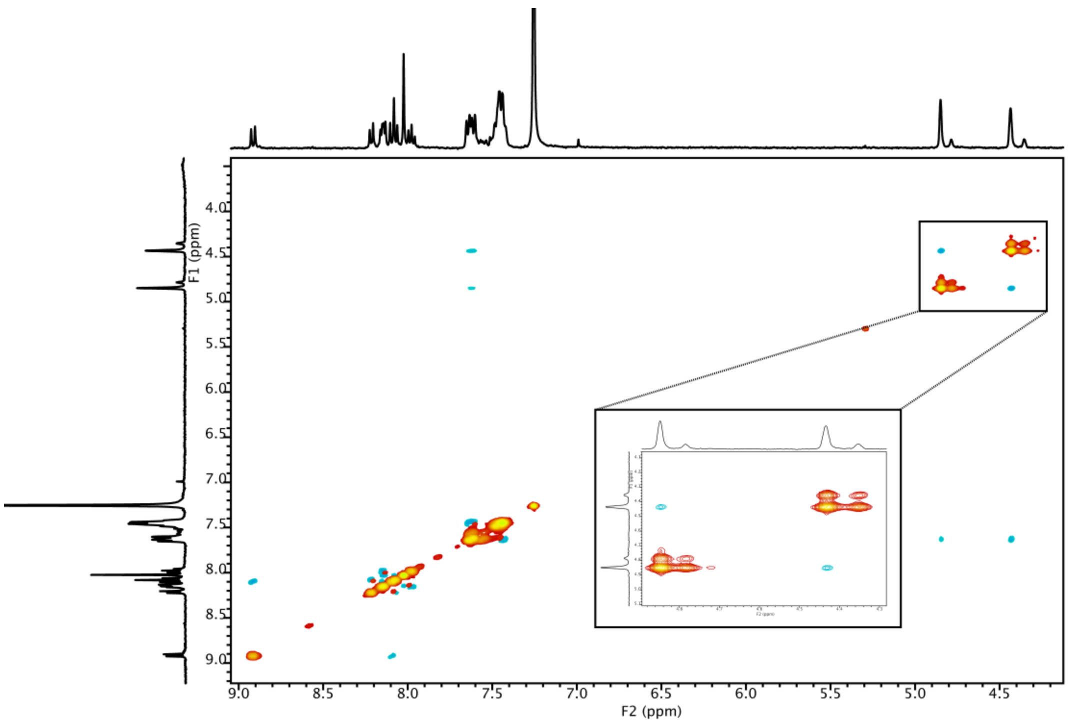

Figure S25. ^1H-^1H NOESY spectrum of compound PAudppf (500 MHz, $CDCl_3$). Inset: $_1H$-$_1H$ EXSY is appreciated between the cyclopentadyenil pairs (signals in red).

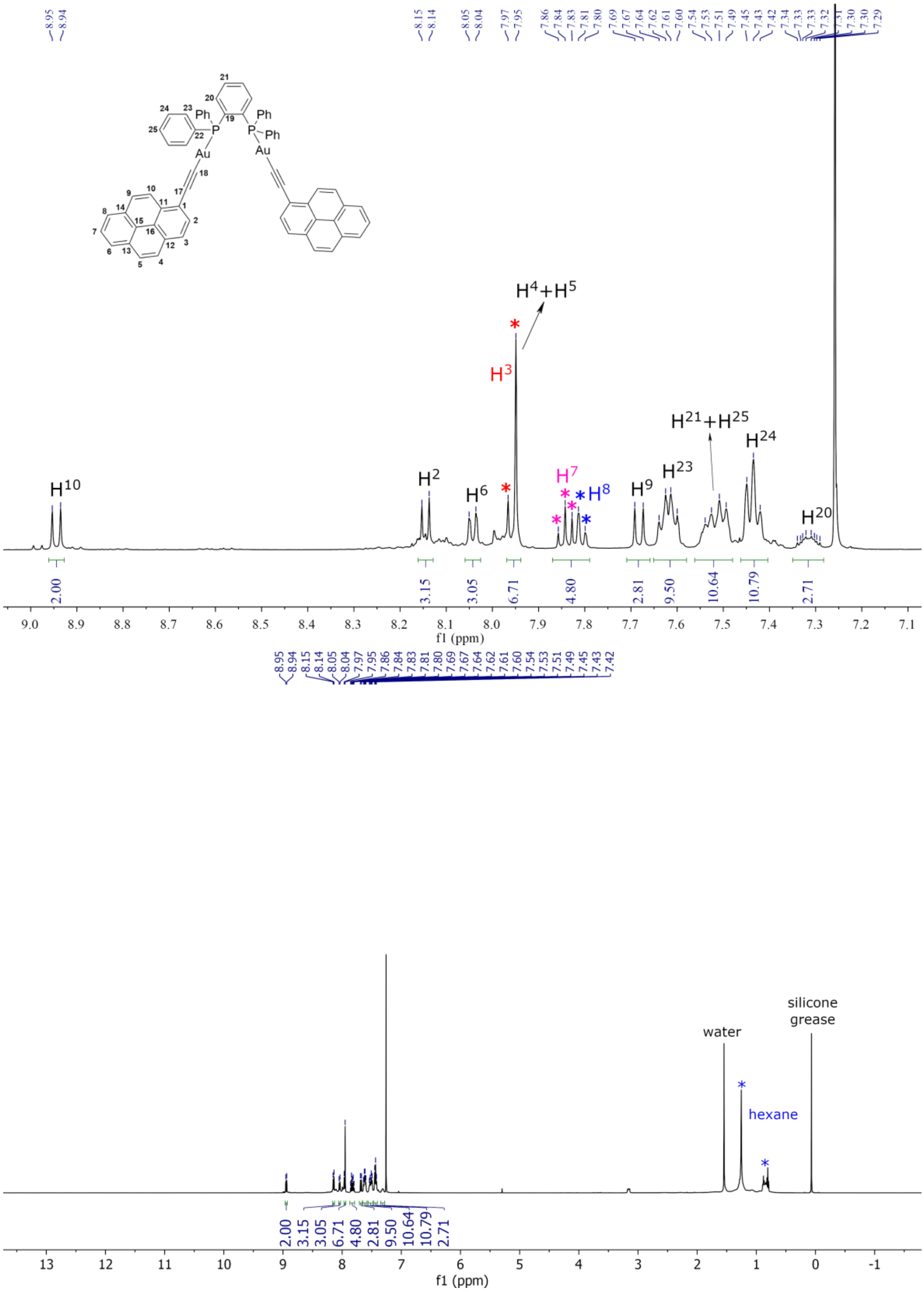

Figure S26. ^{1}H NMR spectrum of compound PAudppbenz (500 MHz, $CDCl_3$); selected regions (above) and full spectrum (below). Some signals are depicted in different colors for clarity purposes.

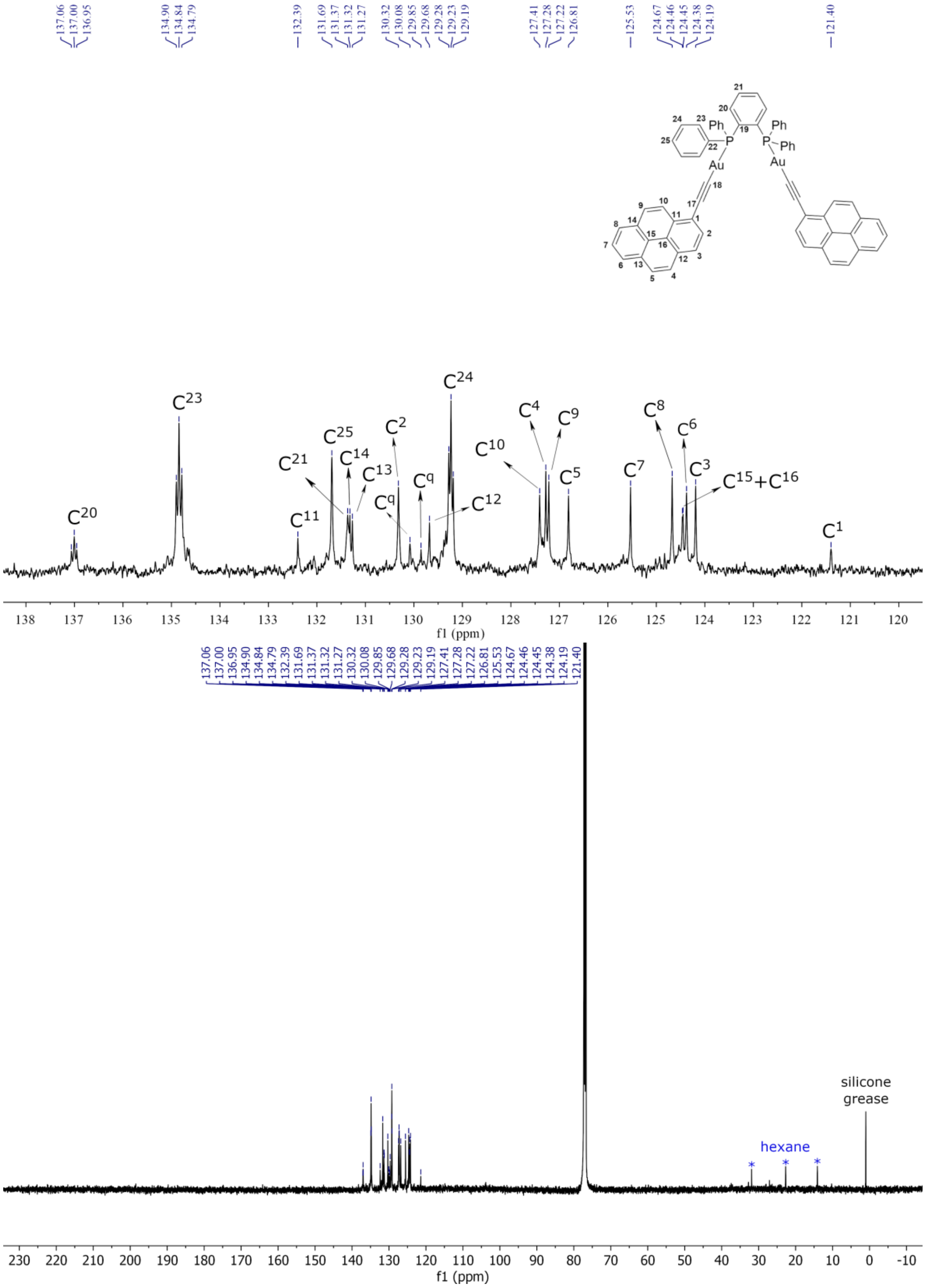

Figure S27. $^{13}C\{^{1}H\}$ NMR spectrum of compound PAudppbenz (126 MHz, $CDCl_3$); selected regions (above) and full spectrum (bellow). Some signals are depicted in different colors for clarity purposes.

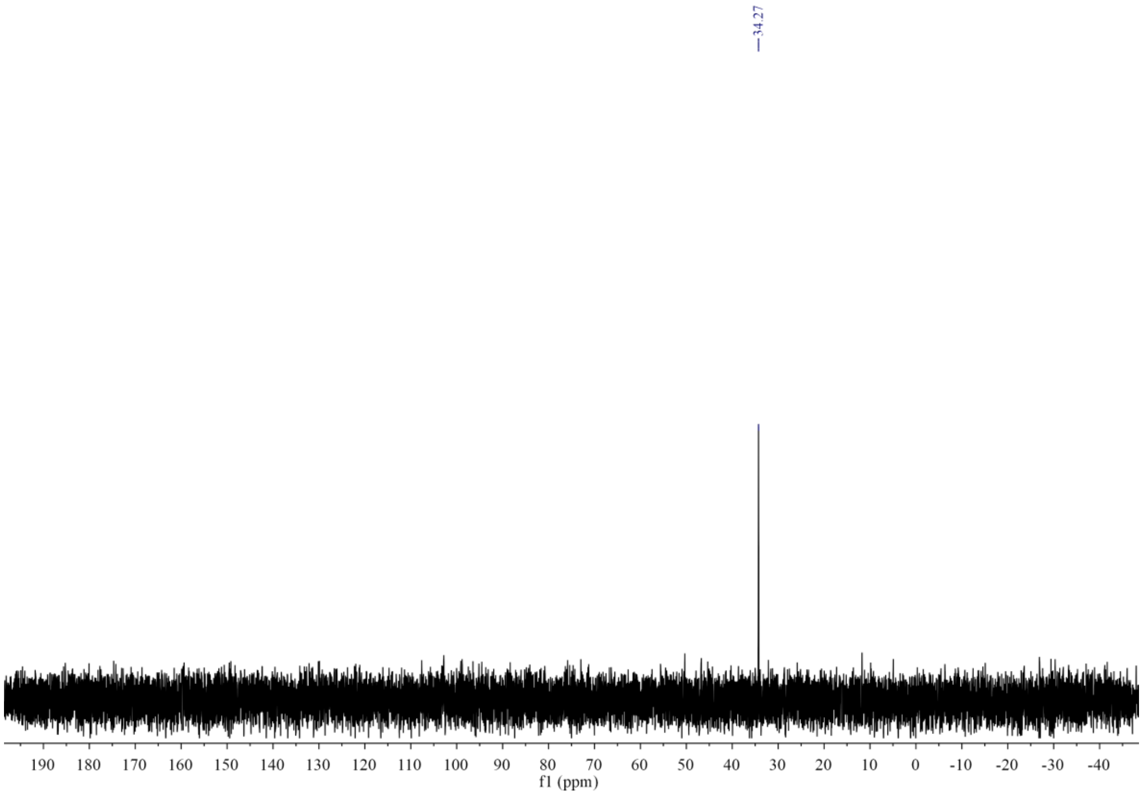

Figure S28. ^{31}P NMR spectrum of compound PAudppbenz (202 MHz, $CDCl_3$).

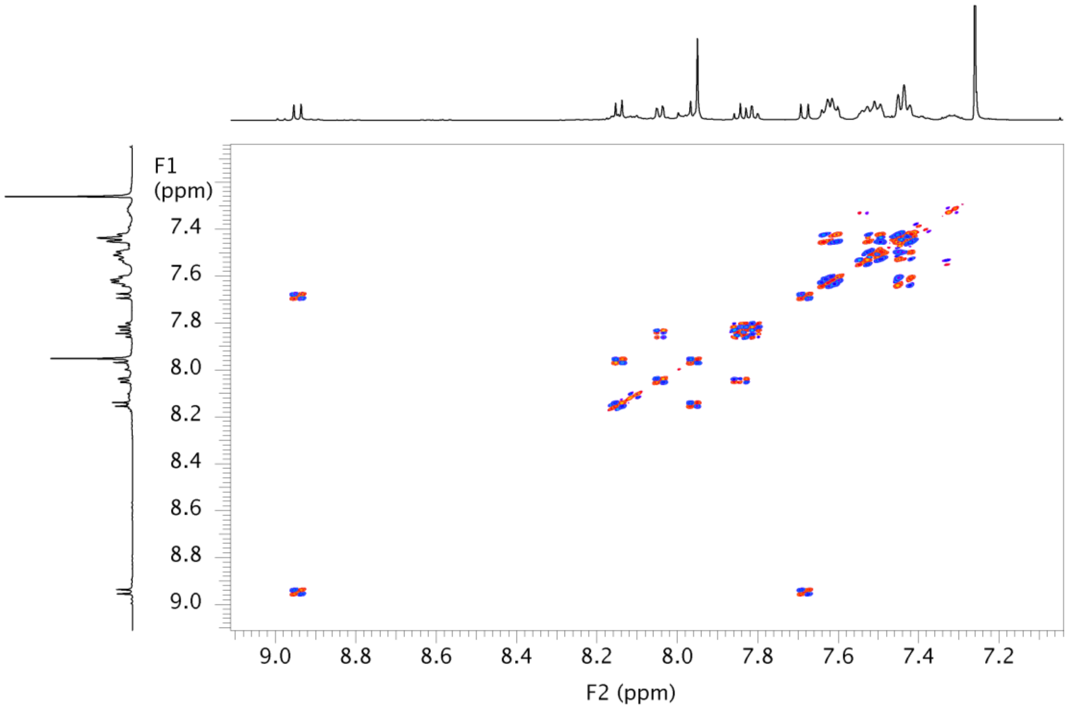

Figure S29. ^{1}H-^{1}H gDQFCOSY spectrum of compound PAudppbenz (500 MHz, $CDCl_3$).

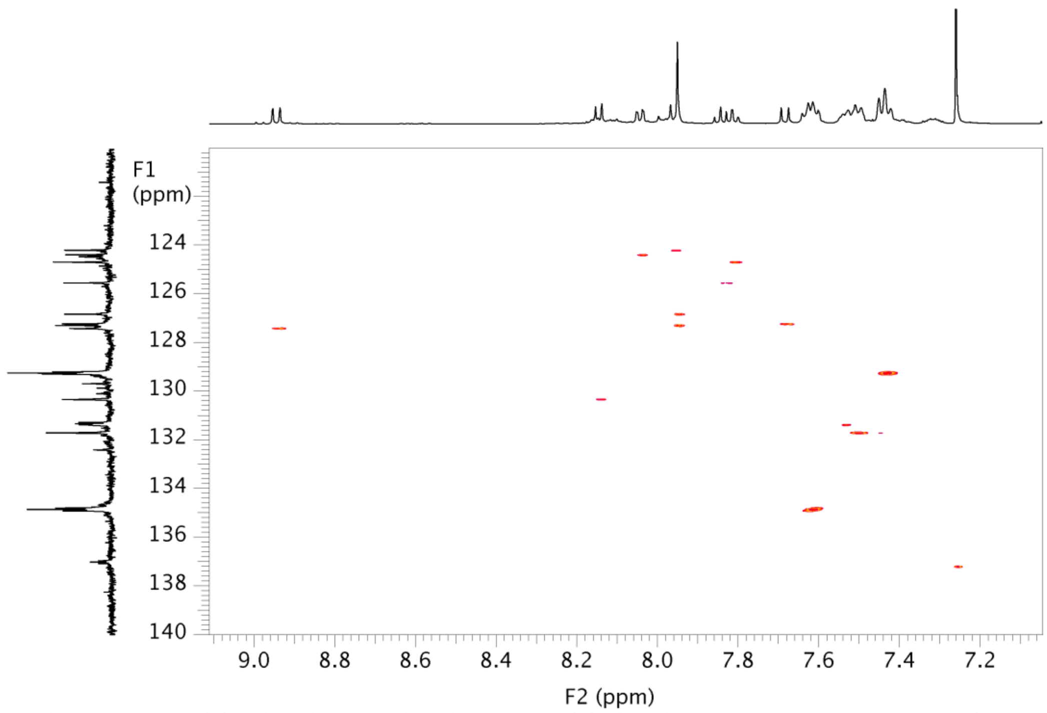

Figure S30. ^{1}H-^{13}C bsgHSQCAD spectrum of compound PAudppbenz (500 MHz, $CDCl_3$).

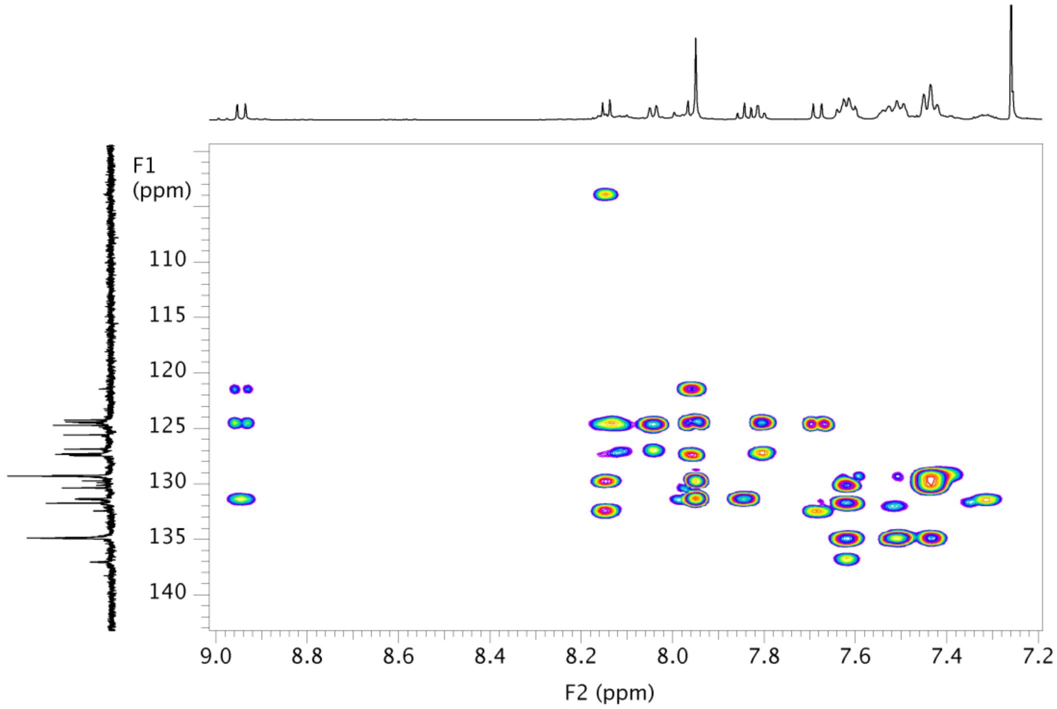

Figure S31. ^{1}H-^{13}C g2cHMBC spectrum of compound PAudppbenz (500 MHz, $CDCl_3$).

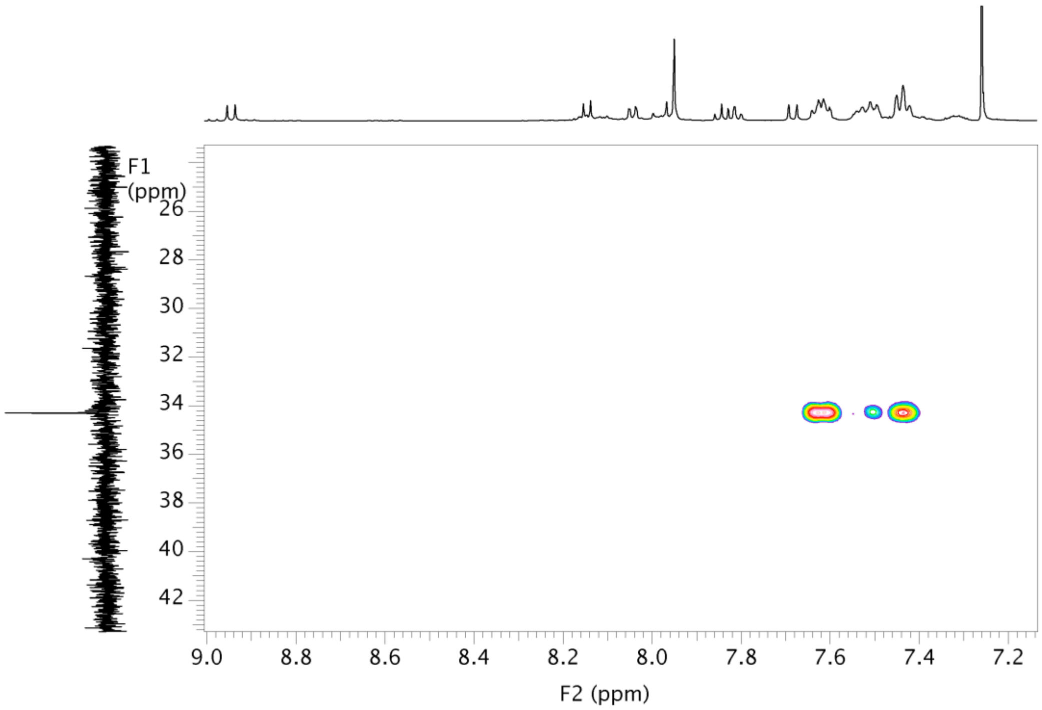

Figure S32. ^{1}H-^{31}P gHMBCAD spectrum of compound PAudppbenz (500 MHz, $CDCl_3$).

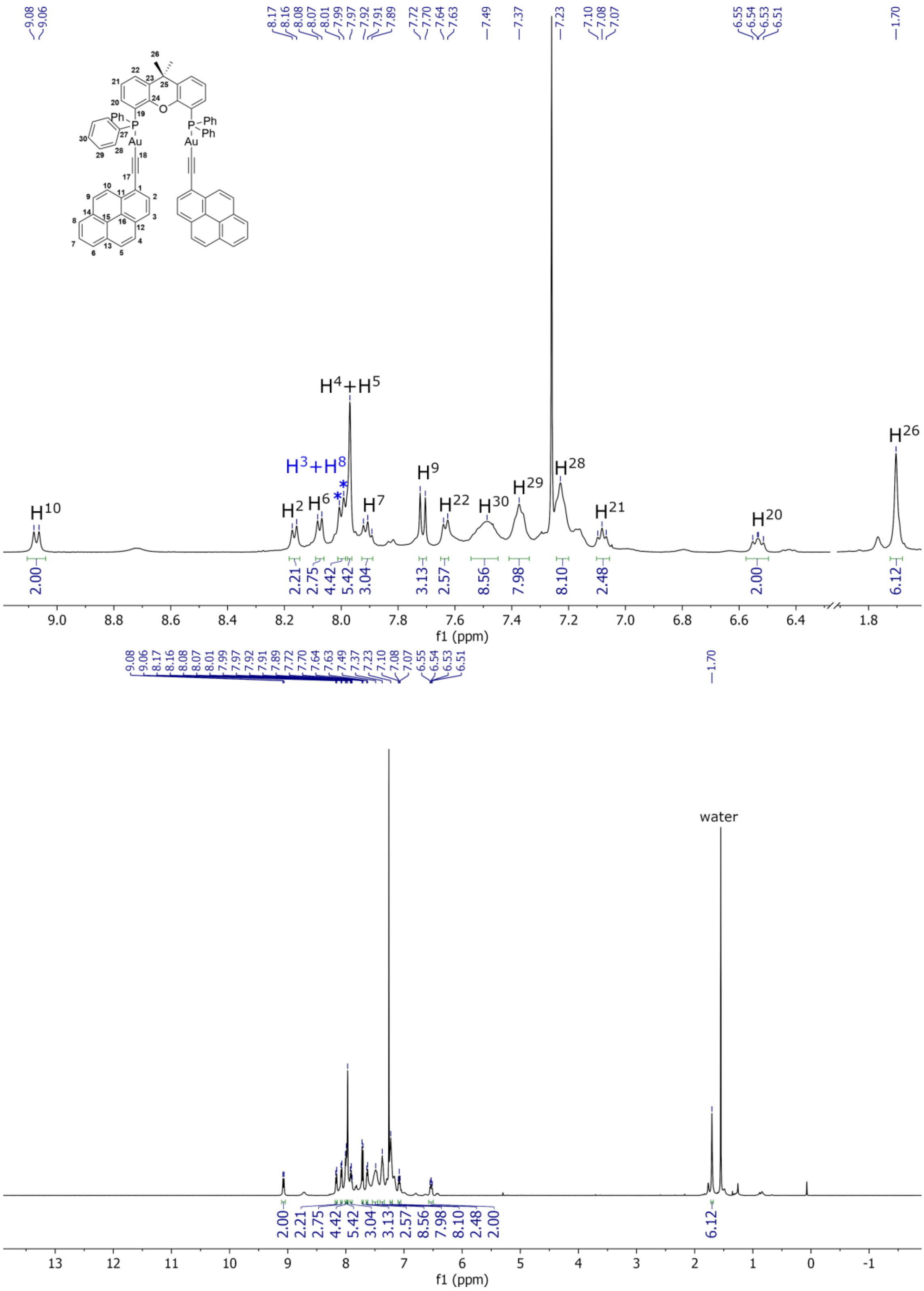

Figure S33. ^{1}H NMR spectrum of compound PAuxantphos (500 MHz, $CDCl_3$); selected regions (above) and full spectrum (below). Some signals are depicted in different colors for clarity purposes.

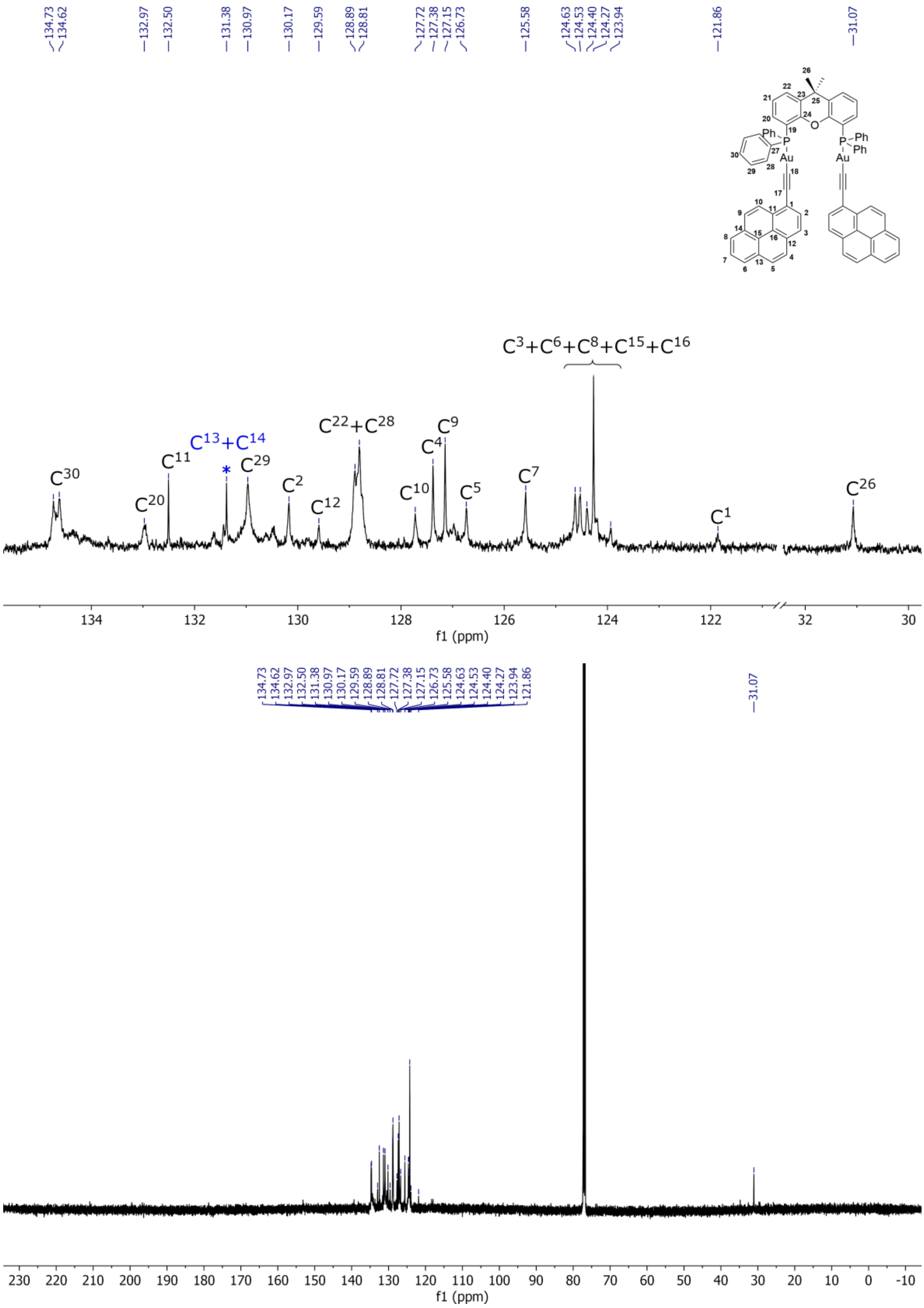

Figure S34. $^{13}C\{^{1}H\}$ NMR spectrum of compound PAuxantphos (126 MHz, $CDCl_3$); selected regions (above) and full spectrum (bellow). Some signals are depicted in different colors for clarity purposes.

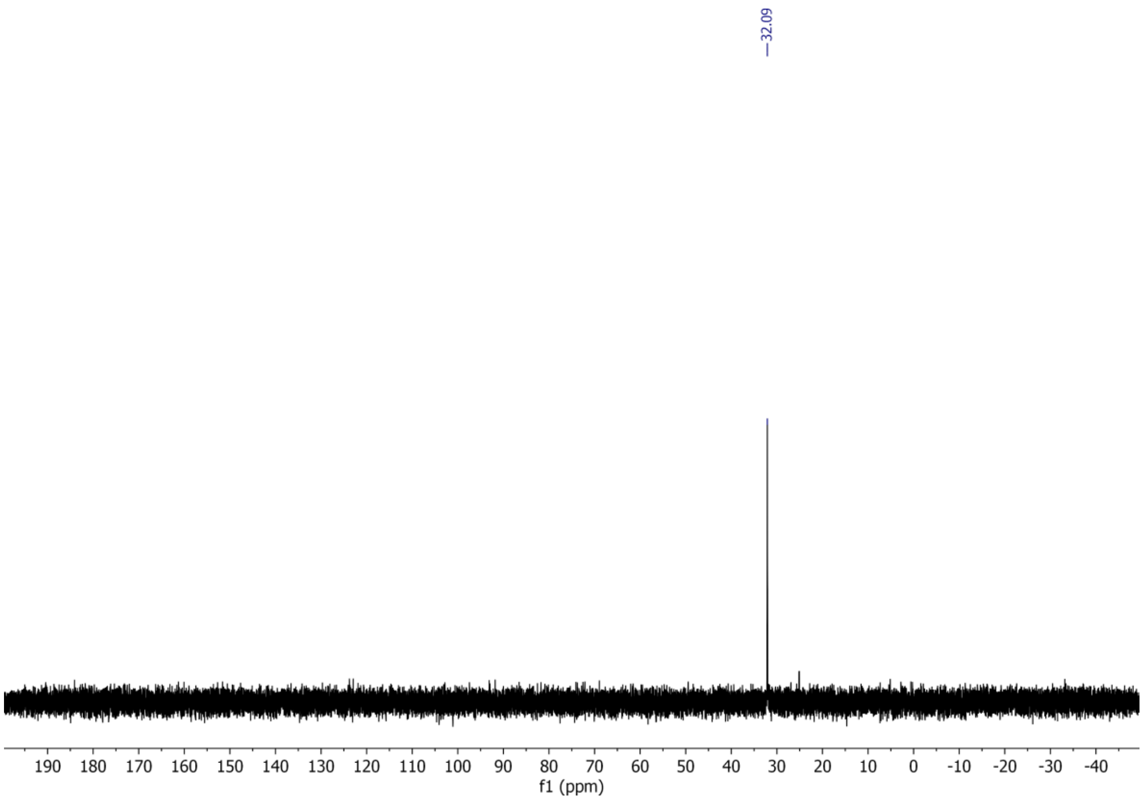

Figure S35. ^{31}P NMR spectrum of compound PAuxantphos (162 MHz, $CDCl_3$).

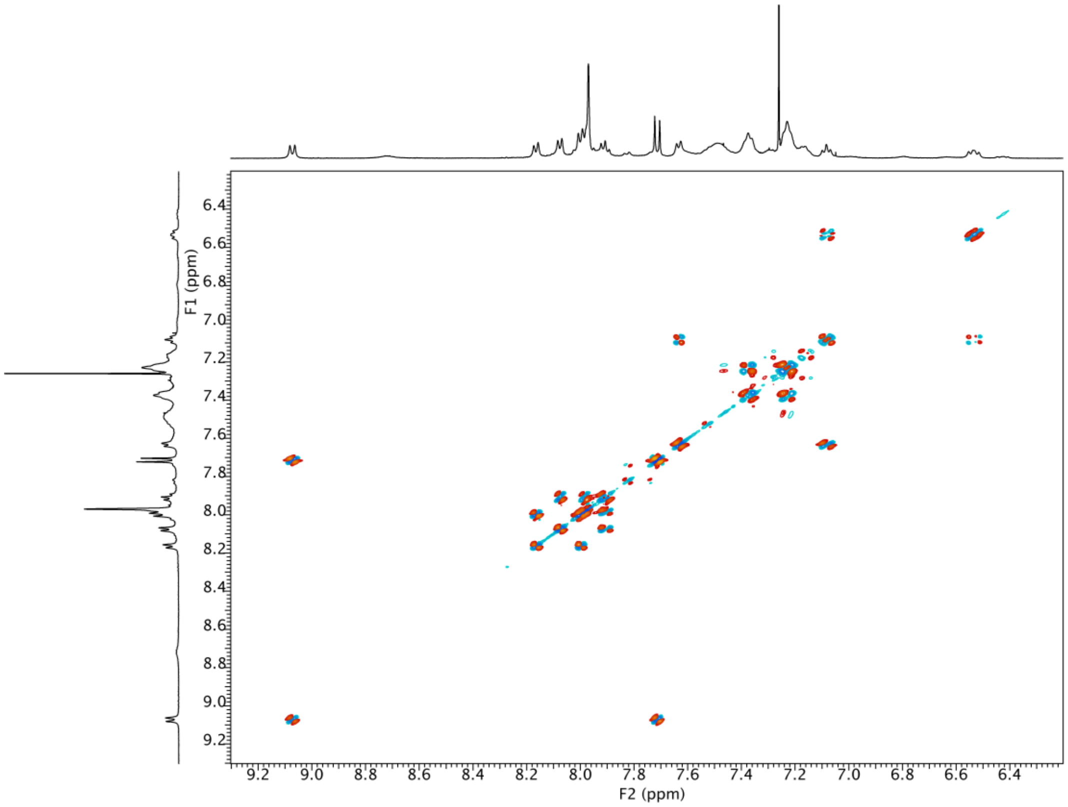

Figure S36. ^{1}H-^{1}H gDQFCOSY spectrum of compound PAuxantphos (500 MHz, $CDCl_3$).

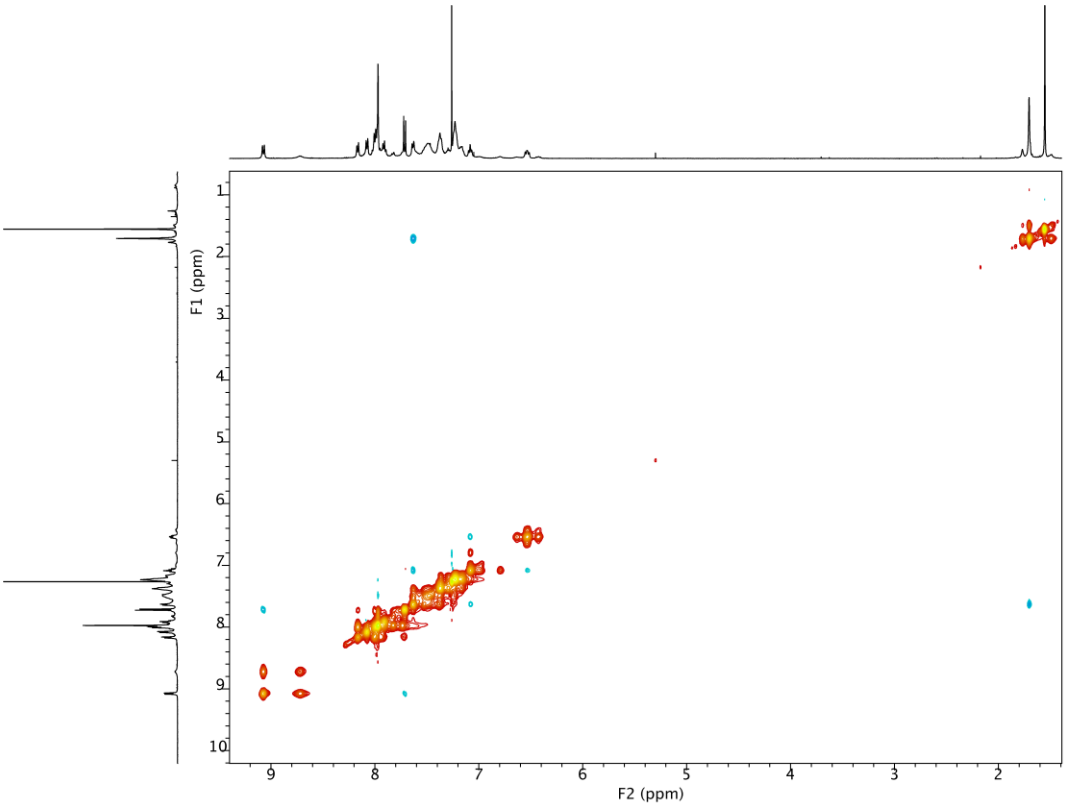

Figure S37. ^{1}H-^{1}H ROESYAD spectrum of compound PAuxantphos (500 MHz, $CDCl_3$).

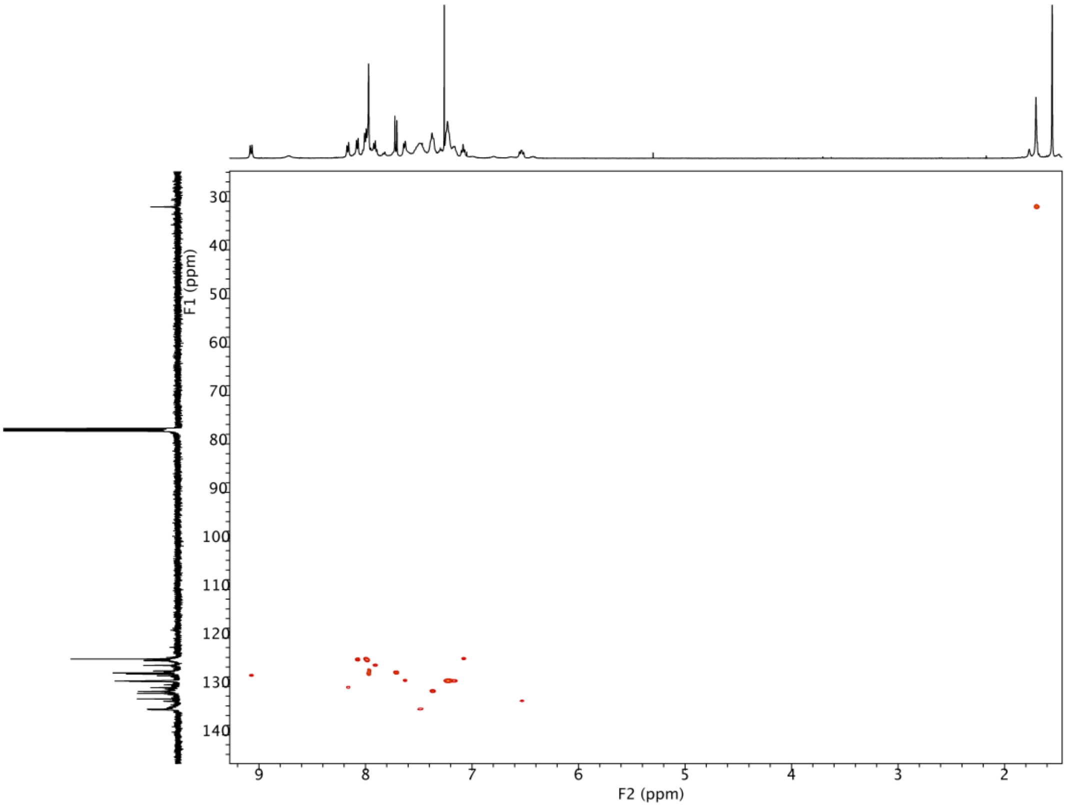

Figure S38. ^{1}H-^{13}C gc2hsqc spectrum of compound PAuxantphos (500 MHz, $CDCl_3$).

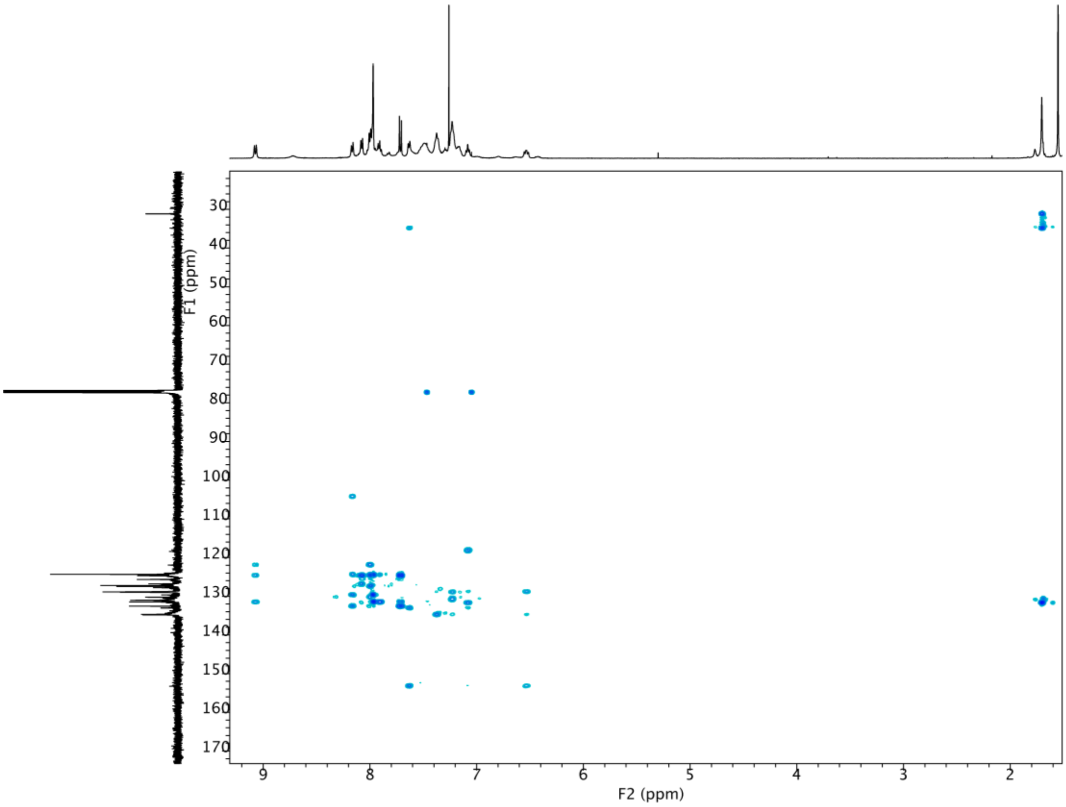

Figure S39. ^{1}H-^{13}C gc2hmbc spectrum of compound PAuxantphos (500 MHz, $CDCl_3$).

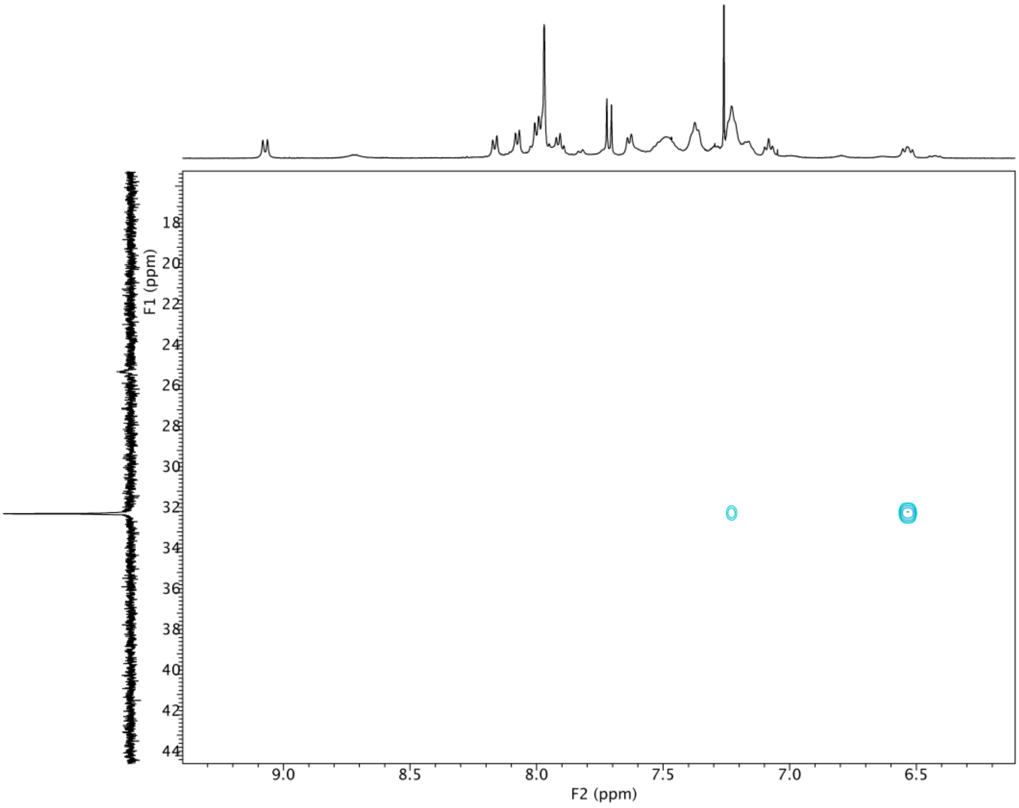

Figure S40. ^{1}H-^{31}P gHMBCAD spectrum of compound PAuxantphos (500 MHz, $CDCl_3$).

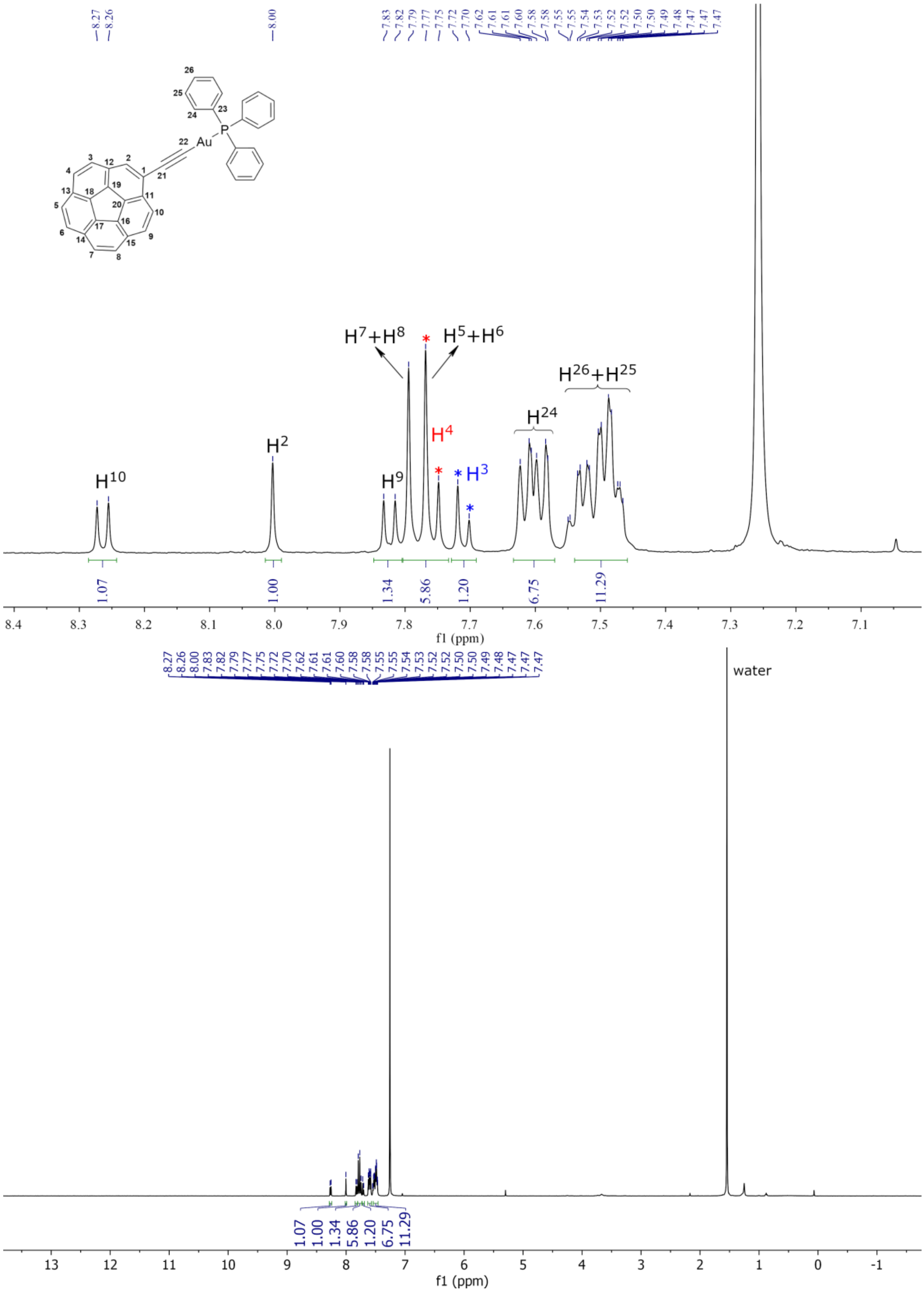

Figure S41. ^{1}H NMR spectrum of compound CAuPPh$_3$ (500 MHz, $CDCl_3$); selected regions (above) and full spectrum (below). Some signals are depicted in different colors for clarity purposes.

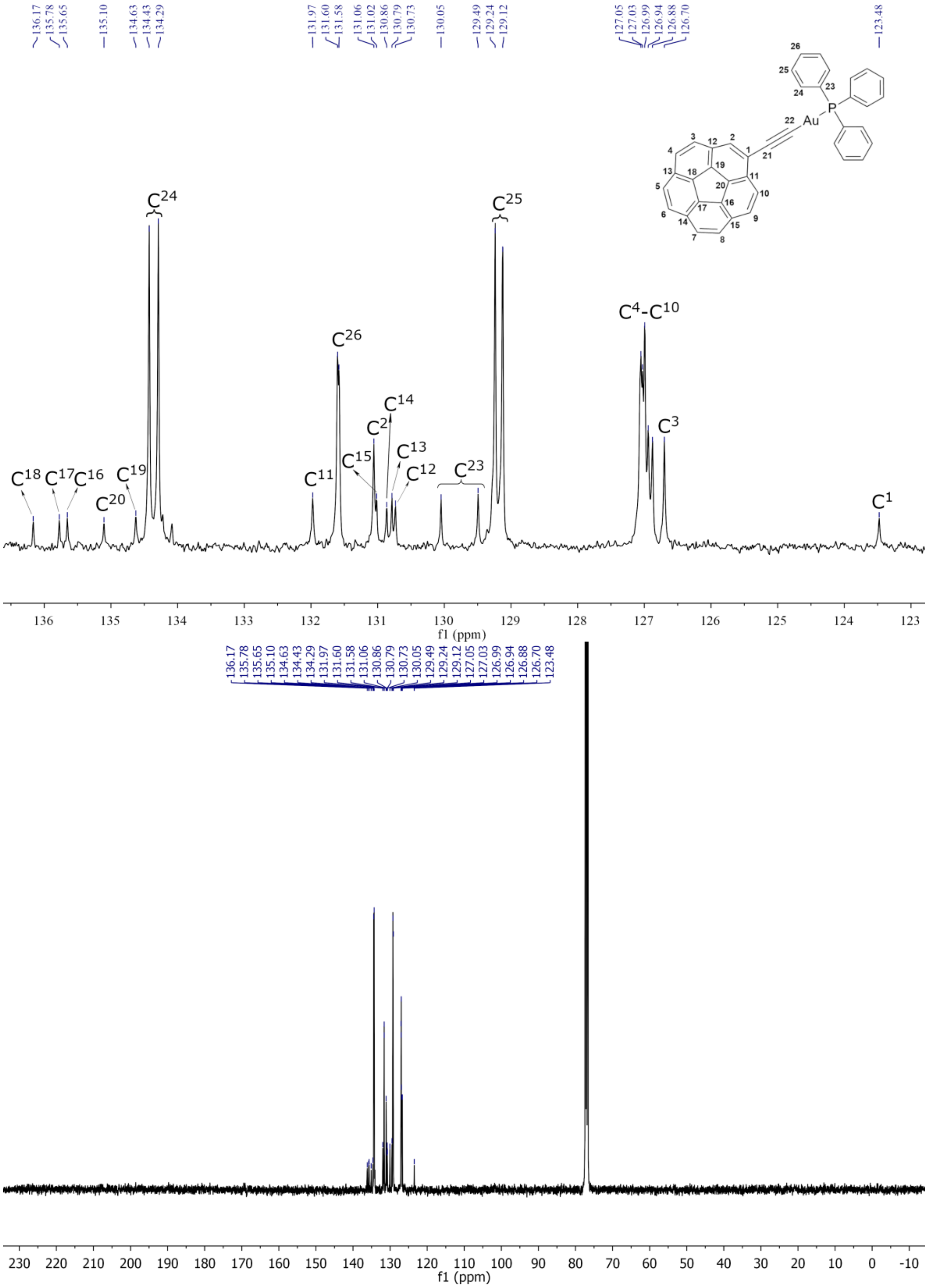

Figure S42. $^{13}C\{^{1}H\}$ NMR spectrum of compound CAuPPh$_3$ (101 MHz, CDCl$_3$); selected regions (above) and full spectrum (below).

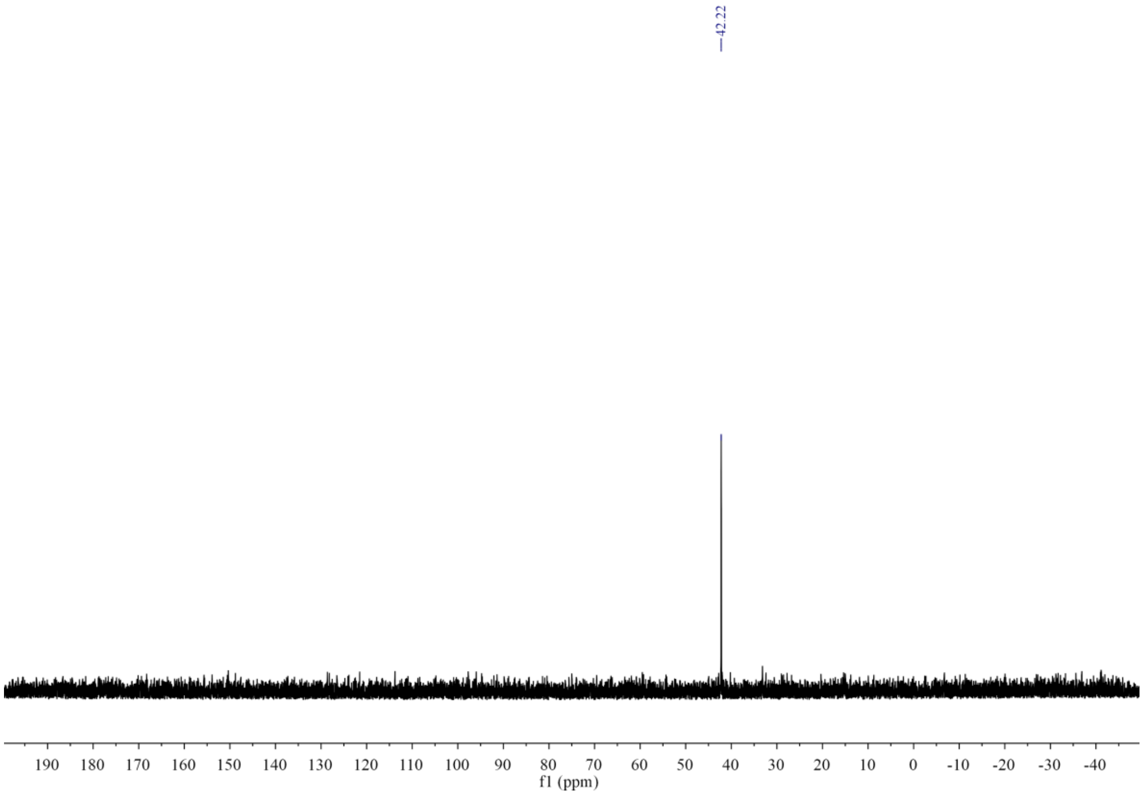

Figure S43. ^{31}P NMR spectrum of compound $CAuPPh_3$ (162 MHz, $CDCl_3$).

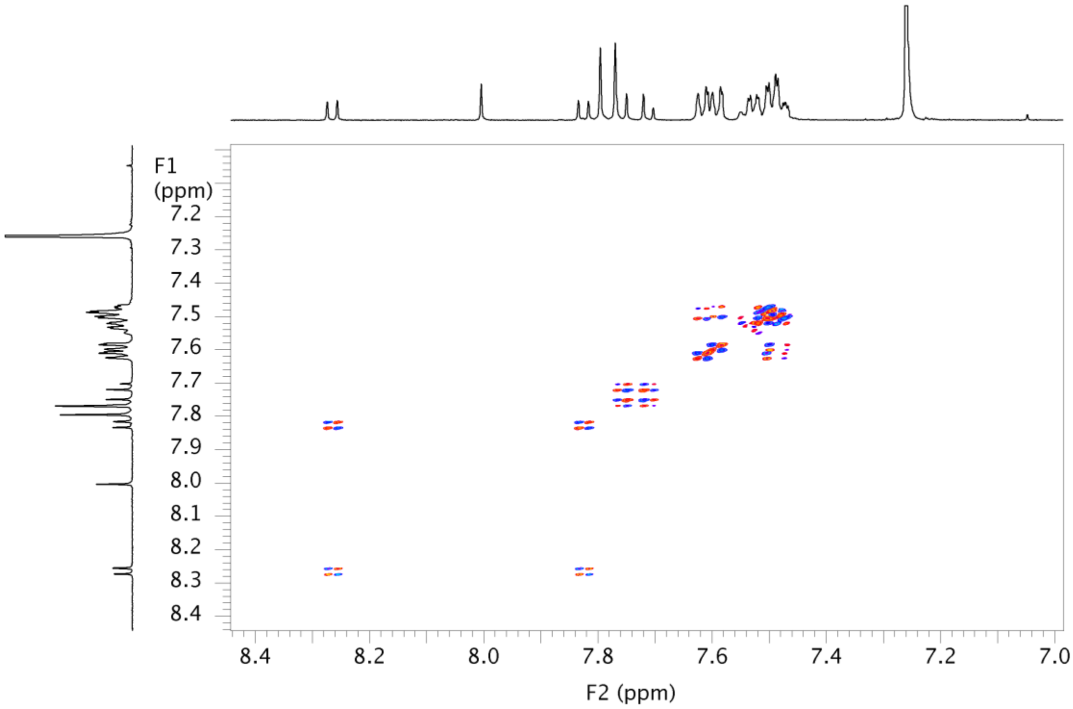

Figure S44. ^{1}H-^{1}H gDQFCOSY spectrum of compound $CAuPPh_3$ (500 MHz, $CDCl_3$).

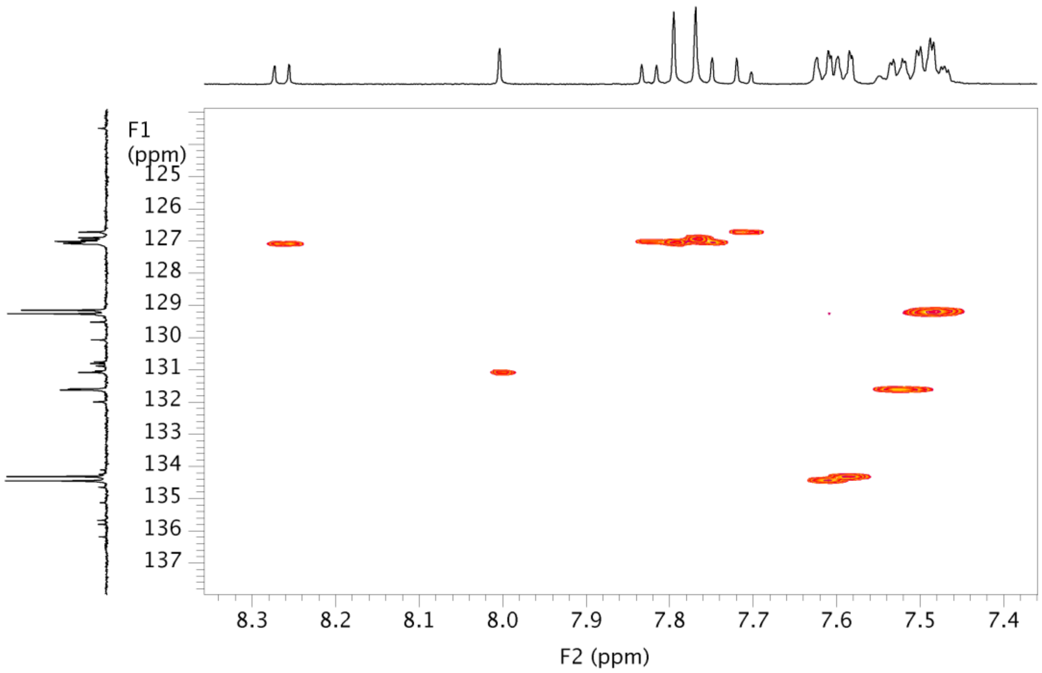

Figure S45. ^{1}H-^{13}C g2cHSQC spectrum of compound CAuPPh$_3$ (500 MHz, CDCl$_3$).

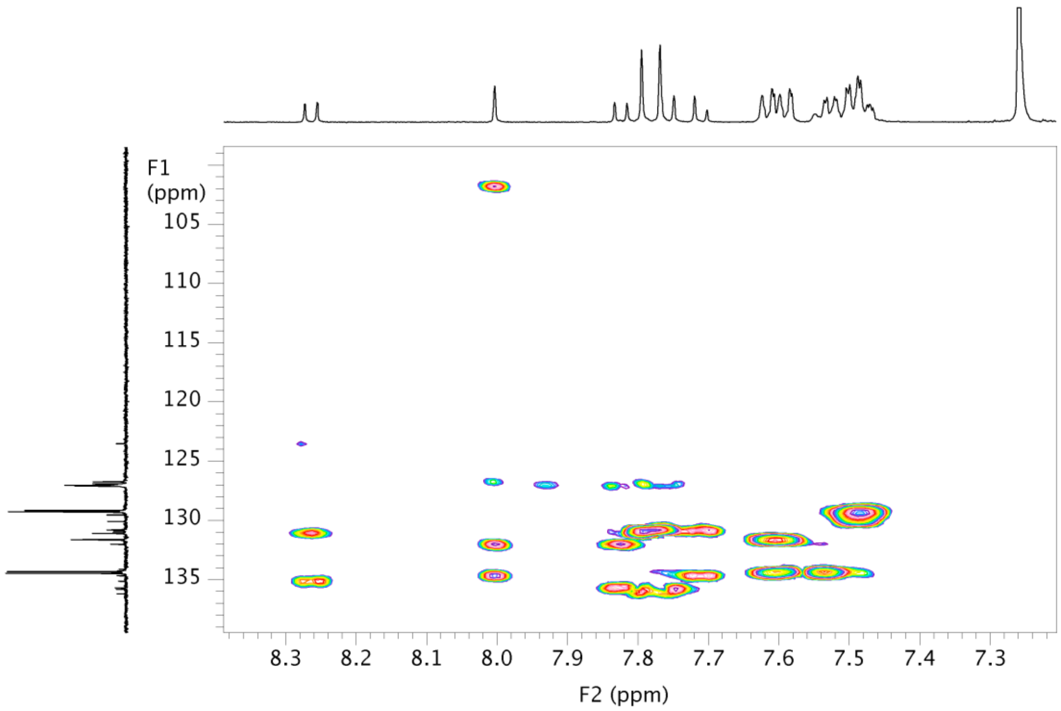

Figure S46. ^{1}H-^{13}C g2cHMBC spectrum of compound CAuPPh$_3$ (500 MHz, CDCl$_3$).

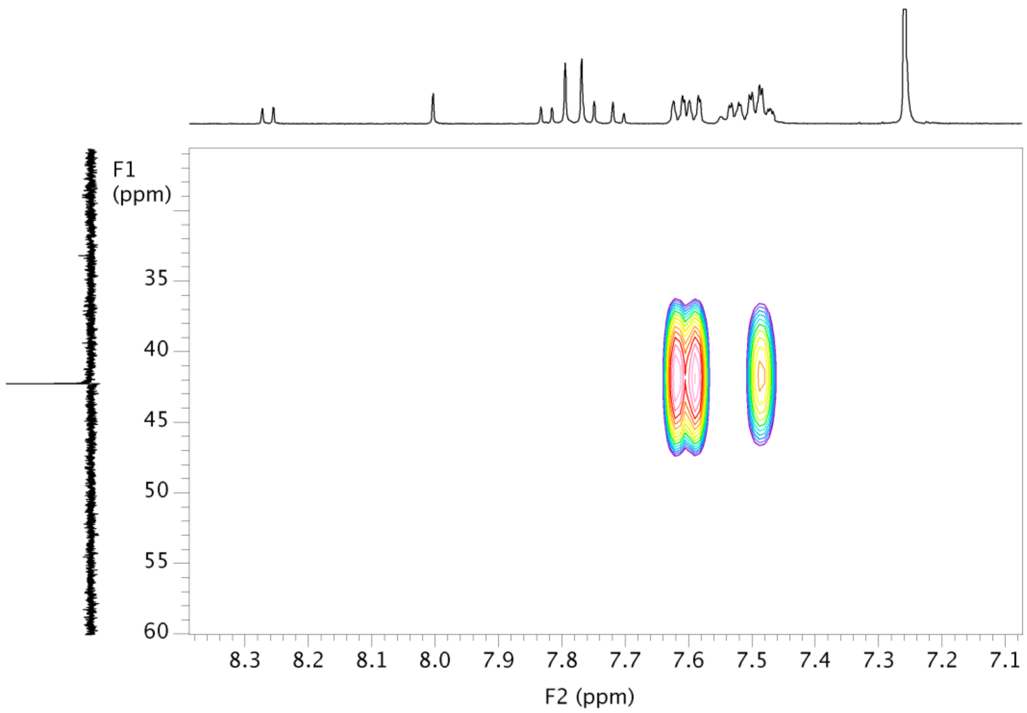

Figure S47. ^1H-^{31}P gHMBCAD spectrum of compound $CAuPPh_3$ (500 MHz, $CDCl_3$).

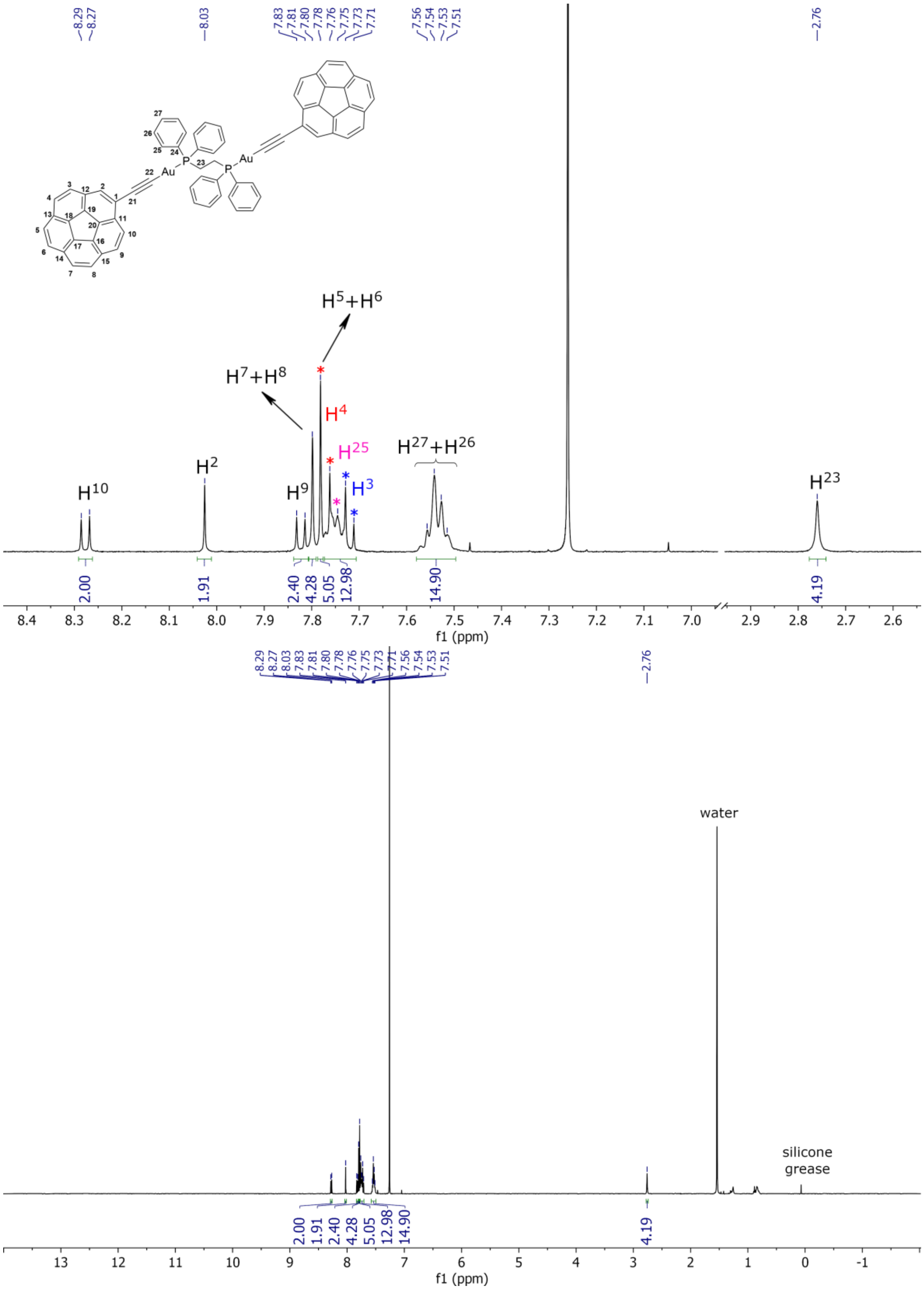

Figure S48. ^{1}H NMR spectrum of compound CAudppe (500 MHz, $CDCl_3$); selected regions (above) and full spectrum (below). Some signals are depicted in different colors for clarity purposes.

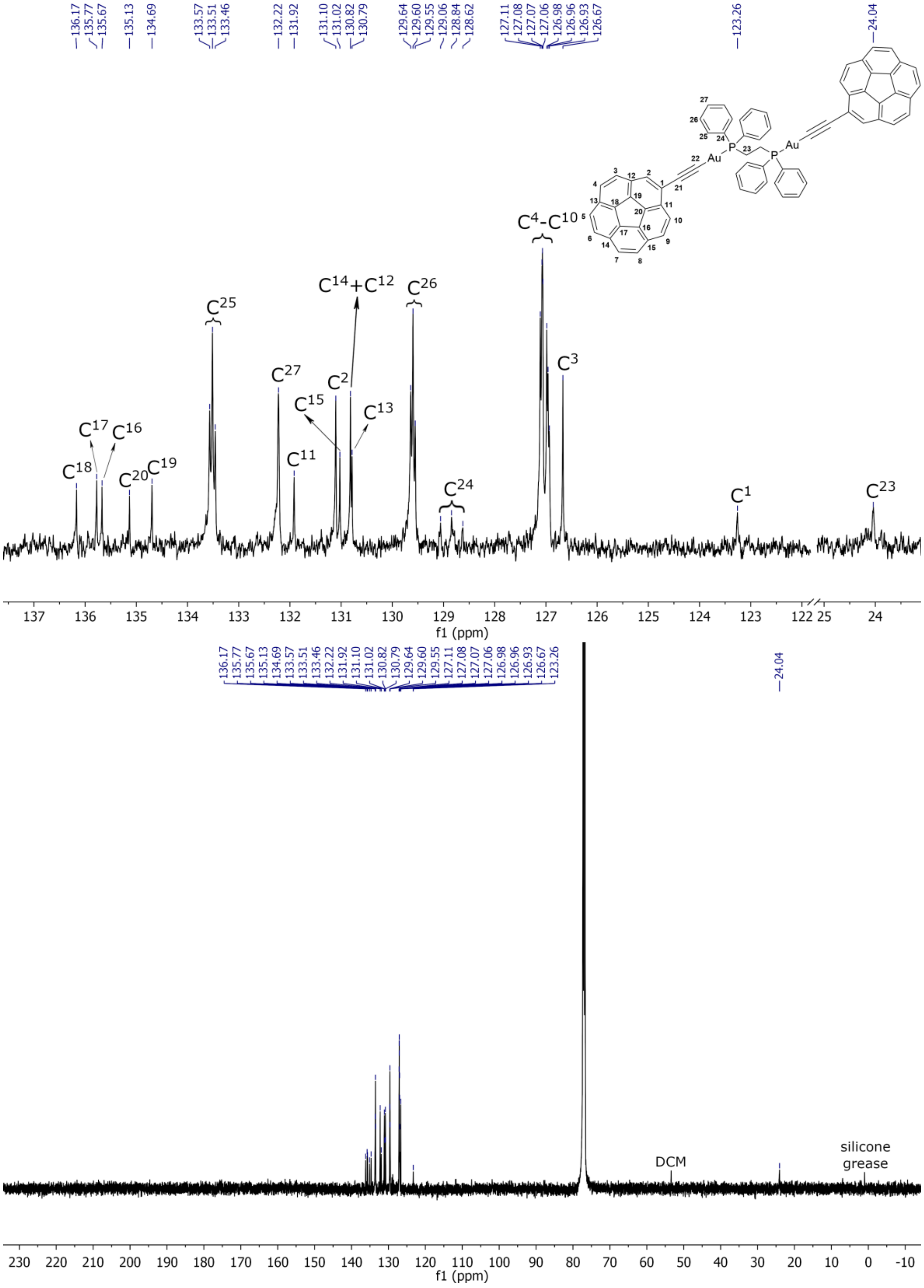

Figure S49. $^{13}C\{^1H\}$ NMR spectrum of compound CAudppe (126 MHz, $CDCl_3$); selected regions (above) and full spectrum (below).

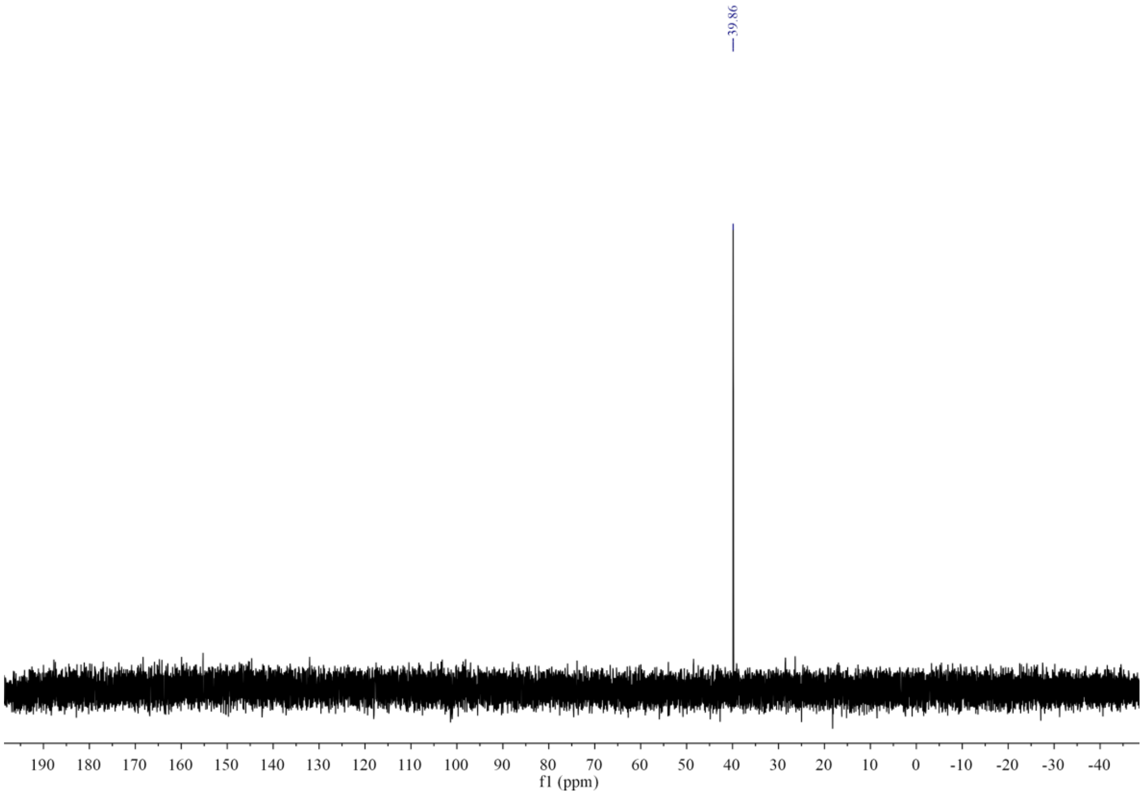

Figure S50. ^{31}P NMR spectrum of compound CAudppe (162 MHz, $CDCl_3$).

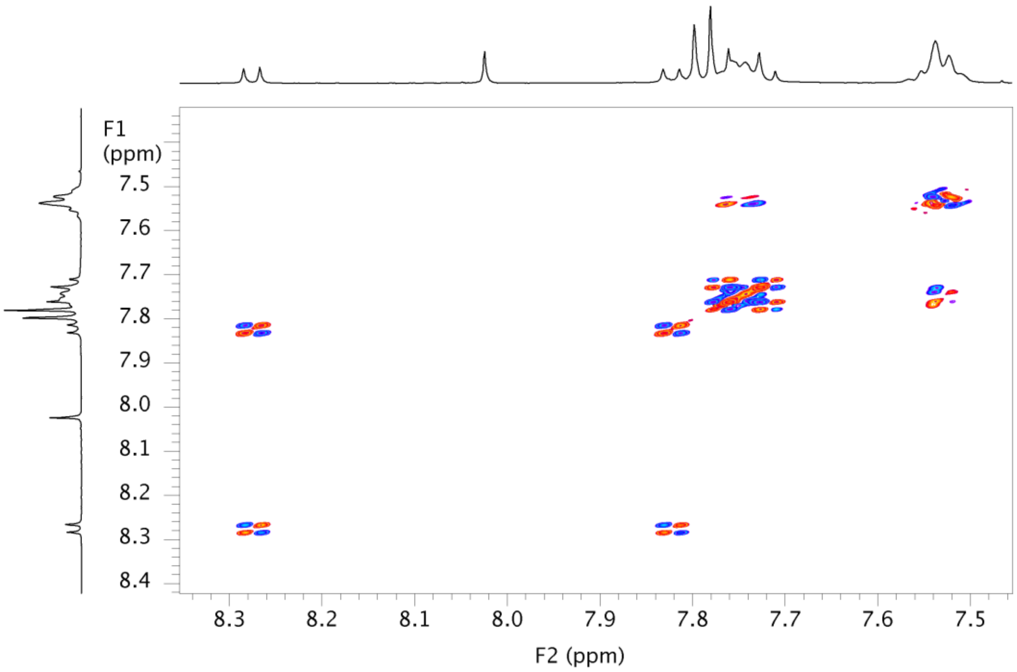

Figure S51. ^{1}H-^{1}H gDQFCOSY spectrum of compound CAudppe (500 MHz, $CDCl_3$).

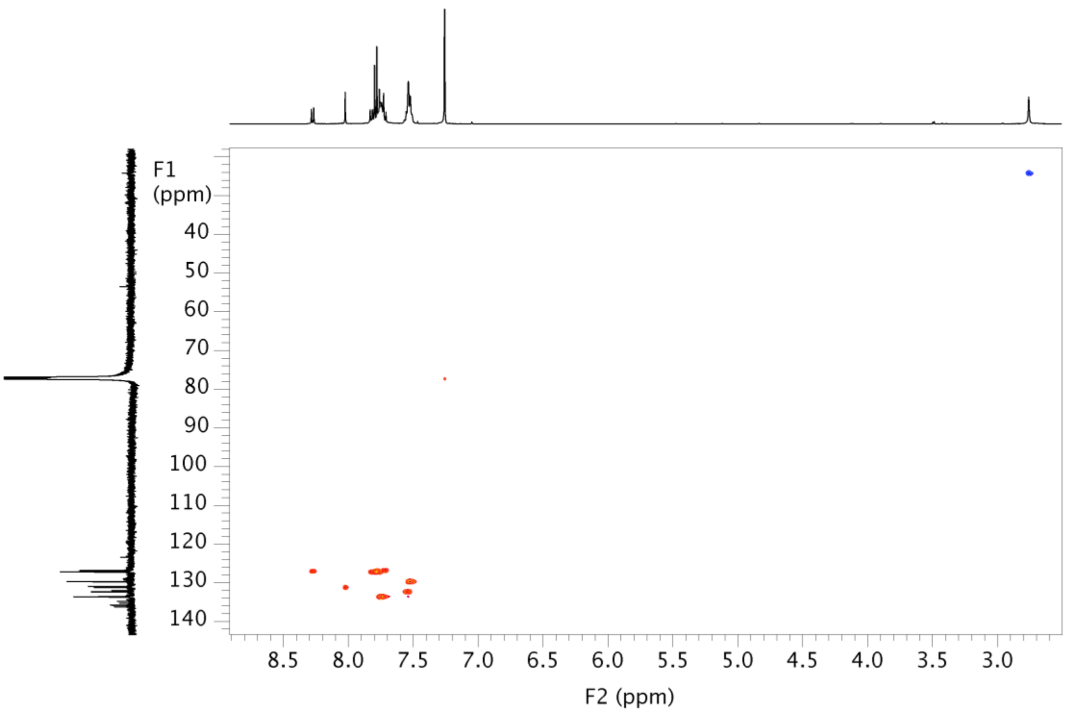

Figure S52. ^{1}H-^{13}C g2cHSQC spectrum of compound CAudppe (500 MHz, $CDCl_3$).

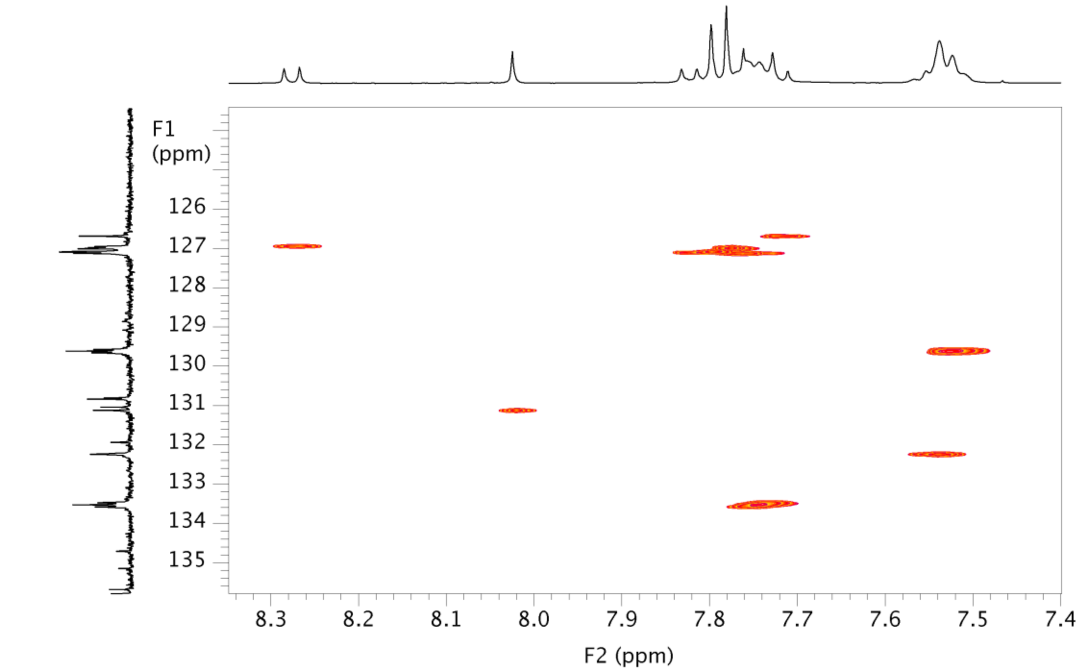

Figure S53. ^{1}H-^{13}C bsgHSQCAD spectrum of compound CAudppe (500 MHz, $CDCl_3$).

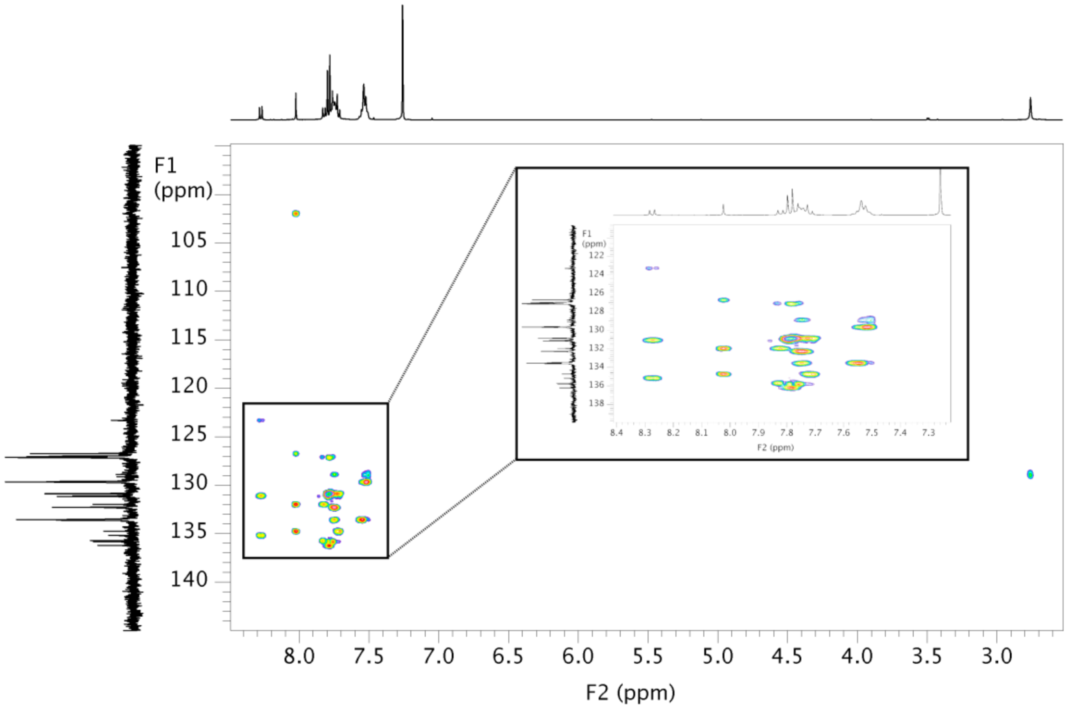

Figure S54. ^1H-^{13}C g2cHMBC spectrum of compound CAudppe (500 MHz, $CDCl_3$).

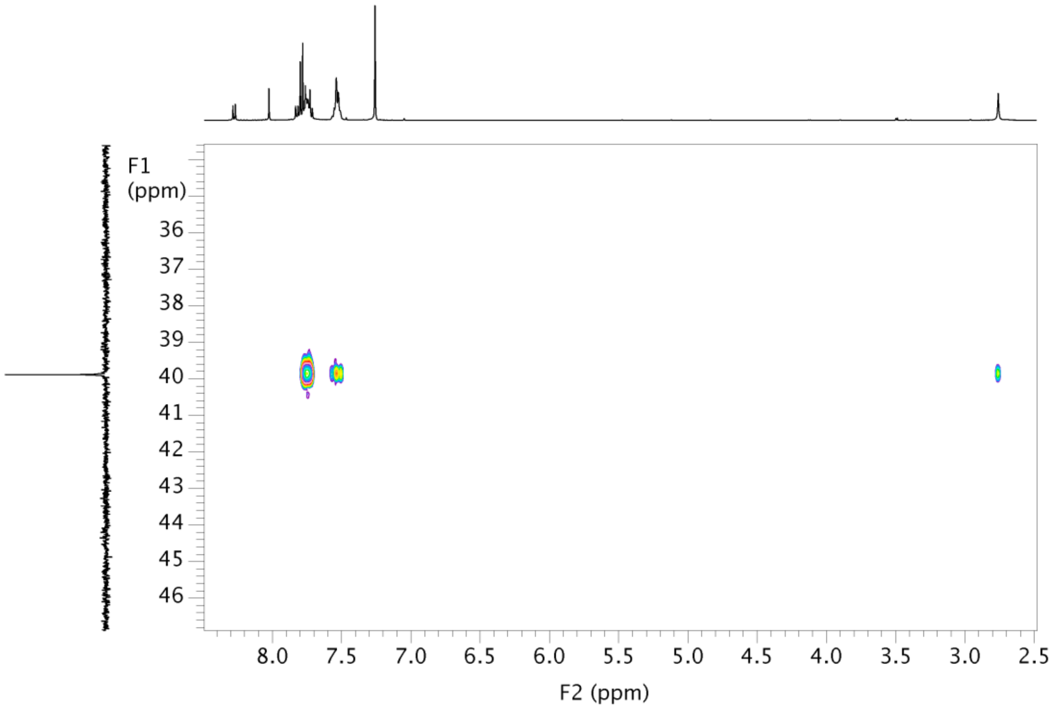

Figure S55. ^1H-^{31}P gHMBCAD spectrum of compound CAudppe (500 MHz, $CDCl_3$).

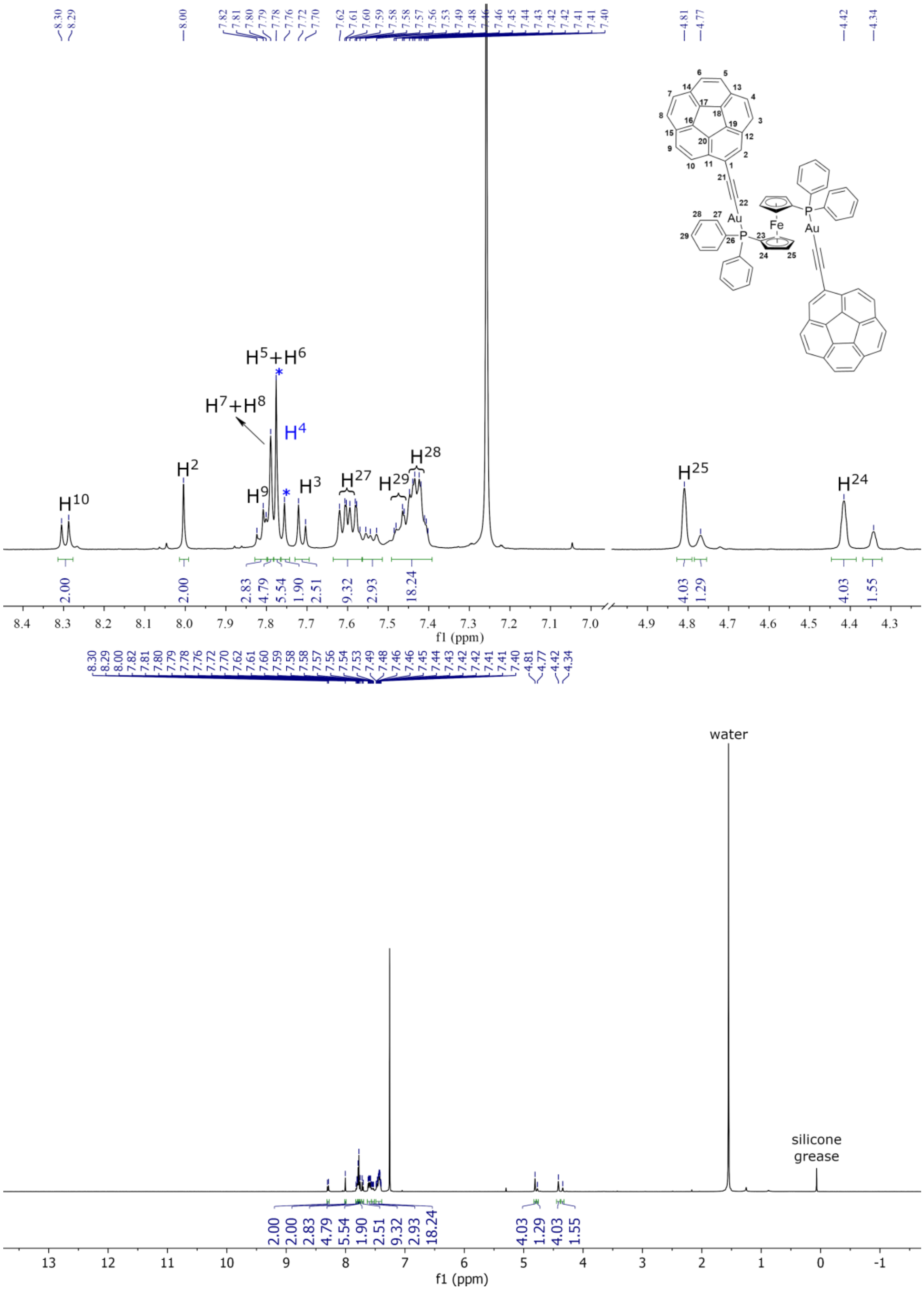

Figure S56. ^{1}H NMR spectrum of compound CAudppf (500 MHz, $CDCl_3$); selected regions (above) and full spectrum (below). Some signals are depicted in different colors for clarity purposes.

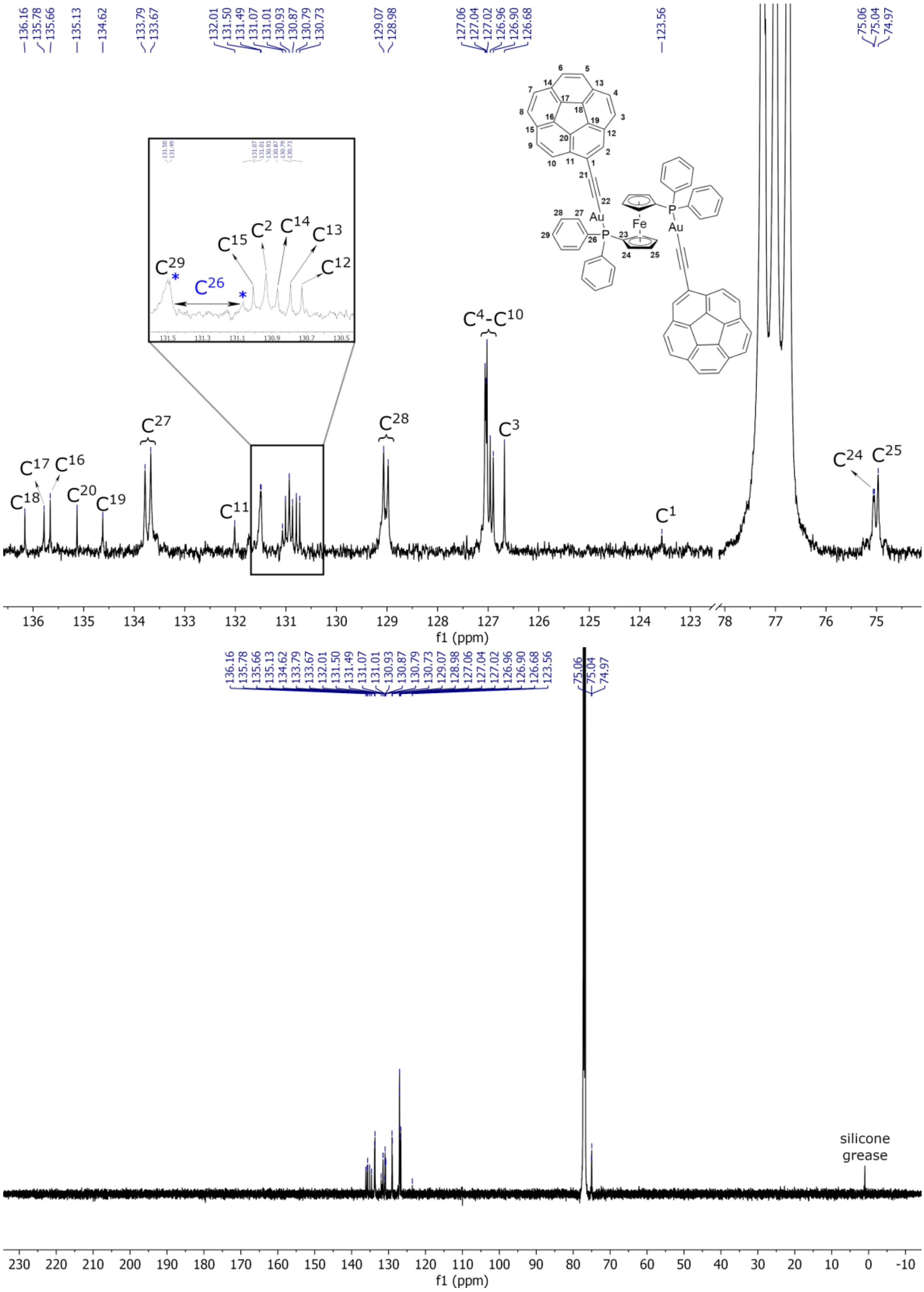

Figure S57. $^{13}C\{^1H\}$ NMR spectrum of compound CAudppf (101 MHz, $CDCl_3$); selected regions (above) and full spectrum (below). Some signals are depicted in different colors for clarity purposes.

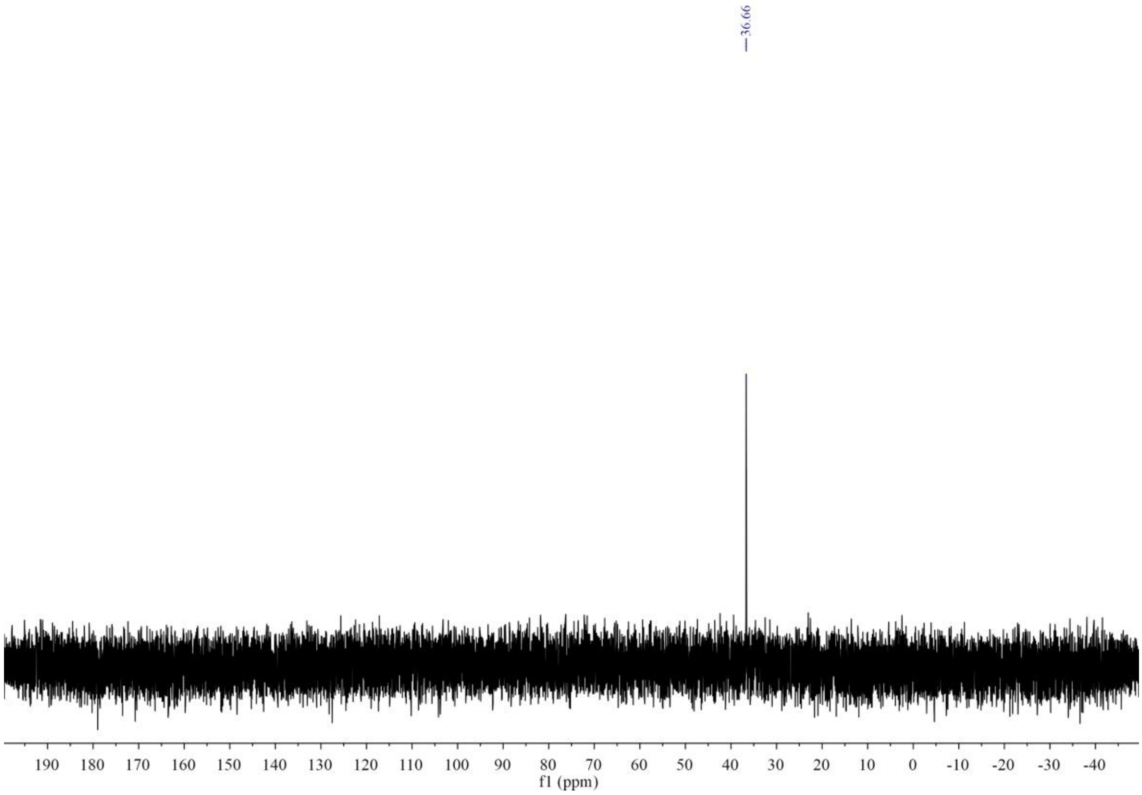

Figure S58. ^{31}P NMR spectrum of compound CAudppf (162 MHz, $CDCl_3$).

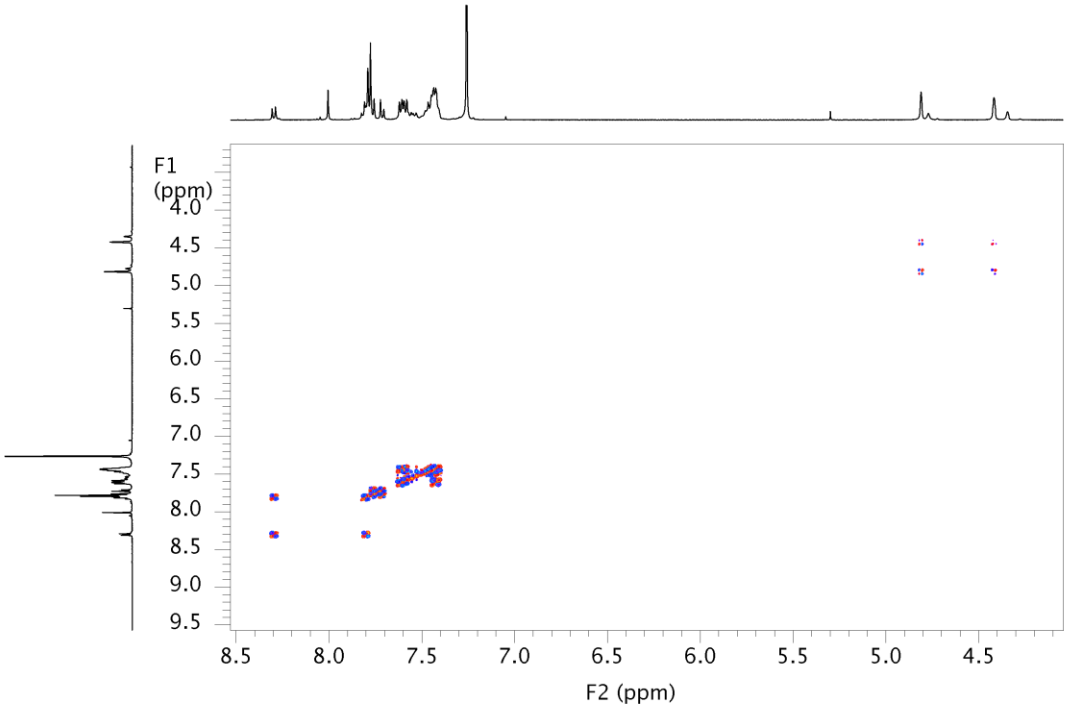

Figure S59. ^{1}H-^{1}H gDQFCOSY spectrum of compound CAudppf (500 MHz, $CDCl_3$).

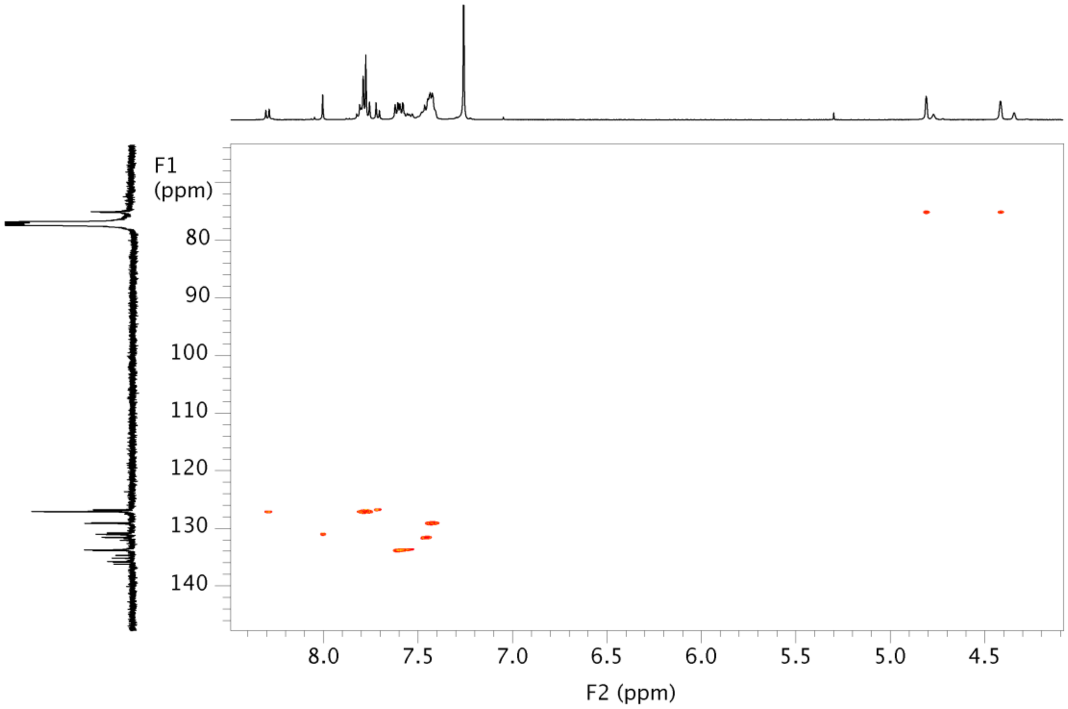

Figure S60. ^{1}H-^{13}C g2cHSQC spectrum of compound CAudppf (500 MHz, $CDCl_3$).

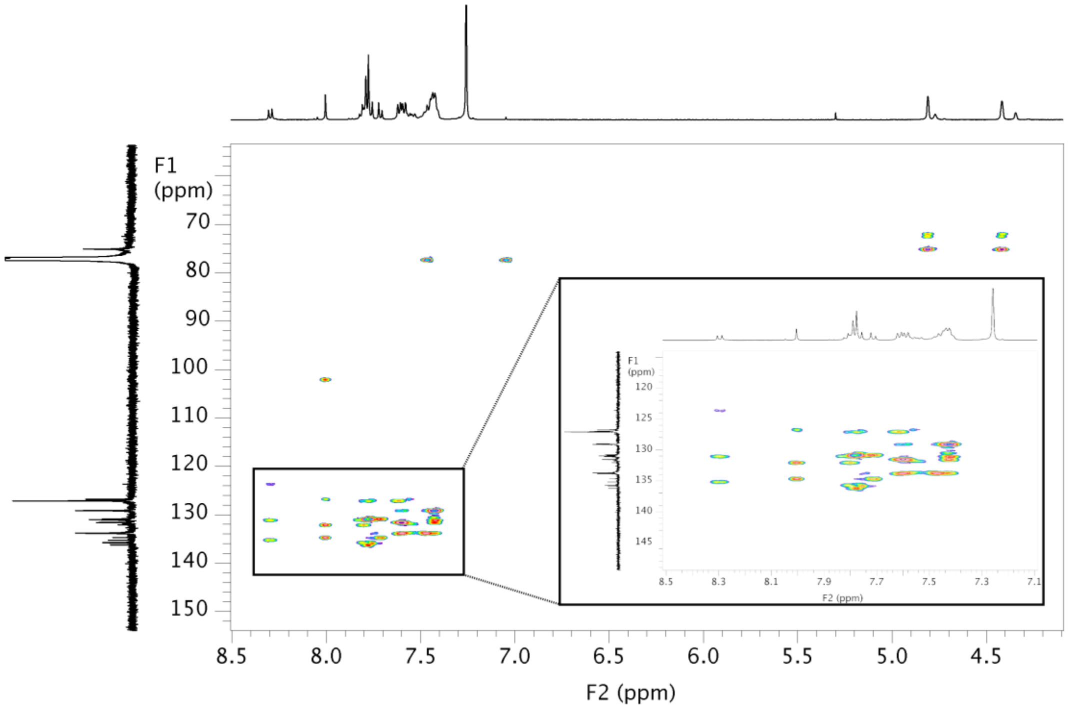

Figure S61. ^{1}H-^{13}C g2cHMBC spectrum of compound CAudppf (500 MHz, $CDCl_3$).

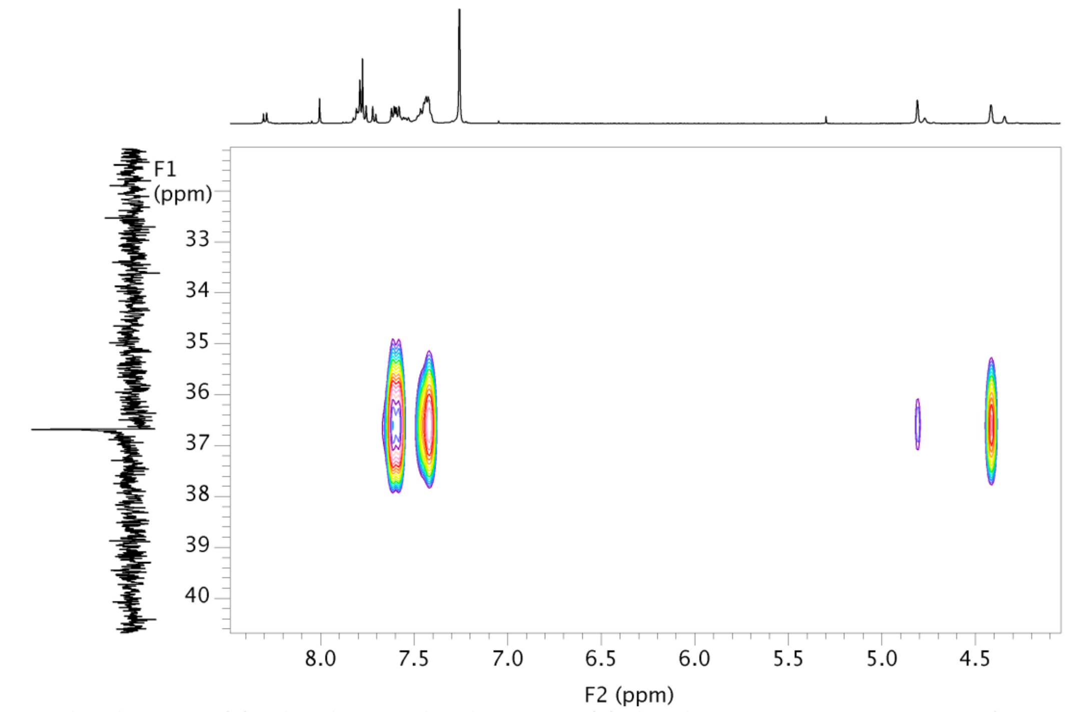

Figure S62. ^{1}H-^{31}P gHMBCAD spectrum of compound CAudppf (500 MHz, $CDCl_3$).

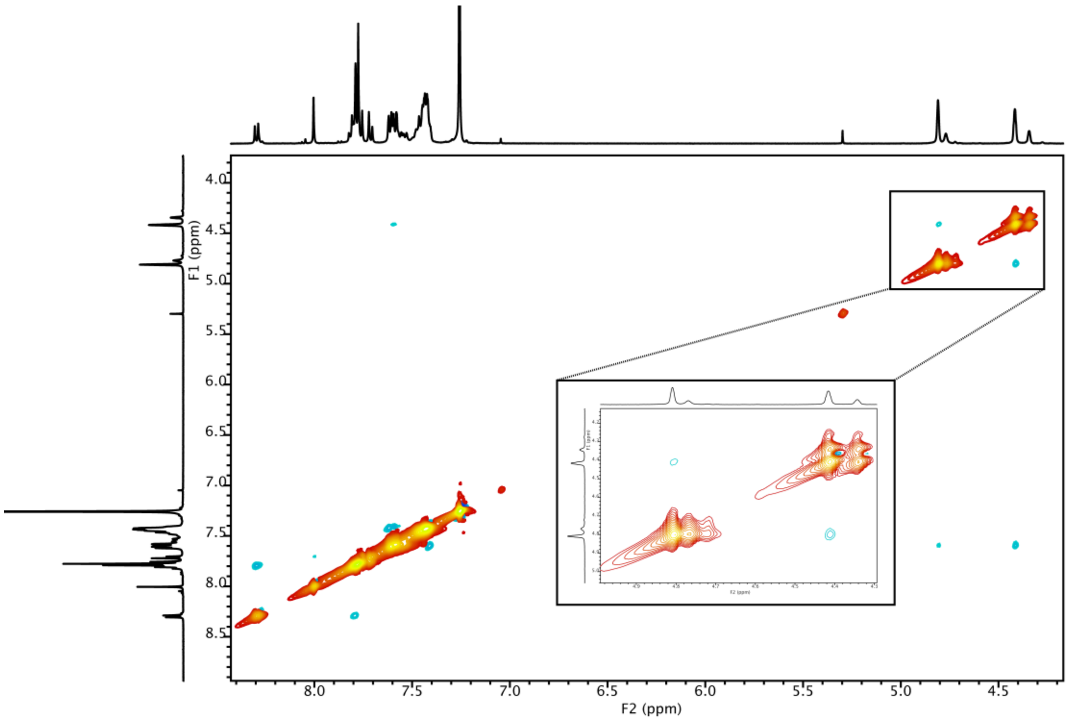

Figure S63. ^{1}H-^{1}H NOESY spectrum of compound CAudppf (500 MHz, $CDCl_3$). Inset: ^{1}H-^{1}H EXSY is appreciated between the cyclopentadyenil pairs (signals in red).

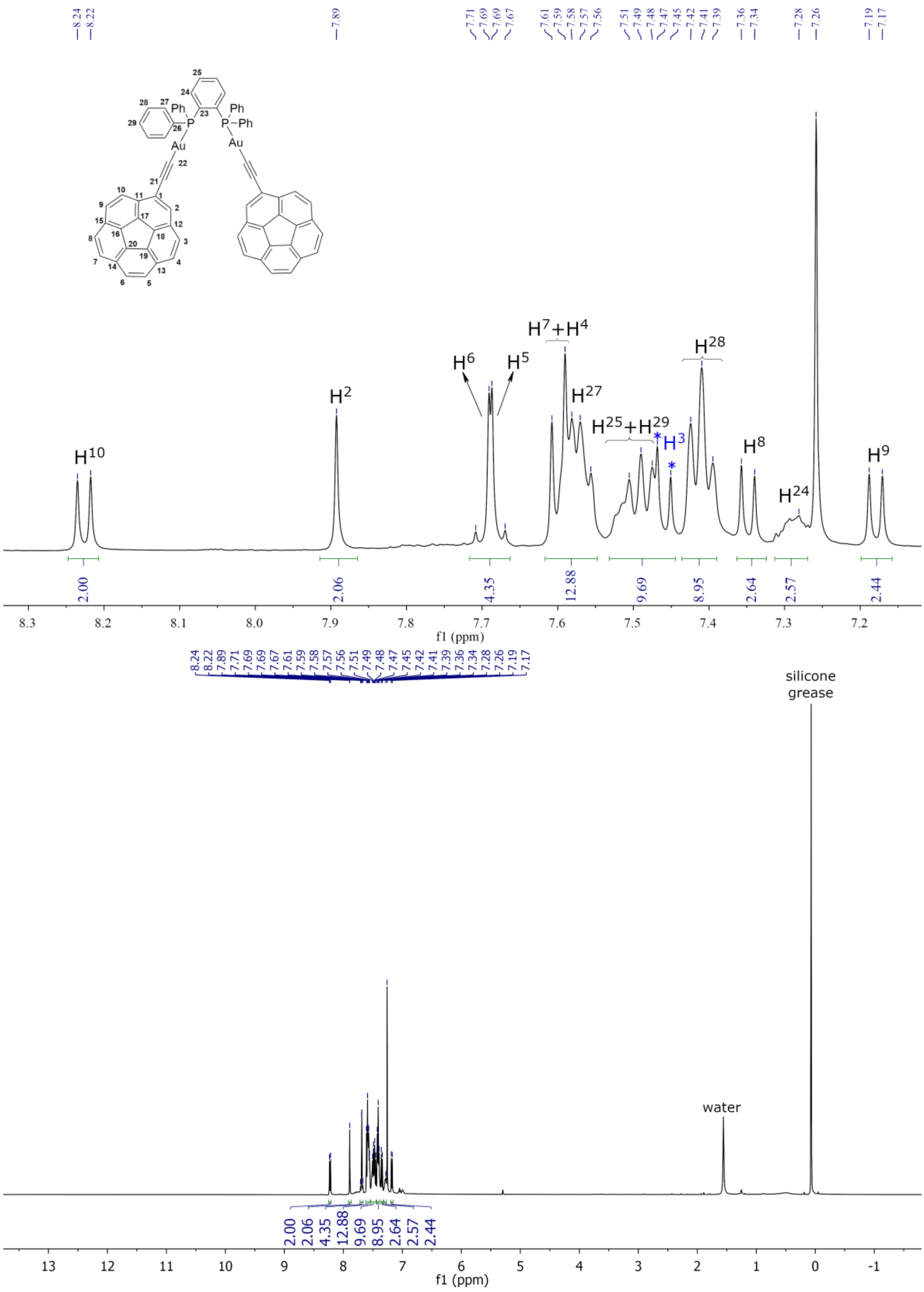

Figure S64. ^{1}H NMR spectrum of compound CAudppbenz (500 MHz, $CDCl_3$); selected regions (above) and full spectrum (below). Some signals are depicted in different colors for clarity purposes.

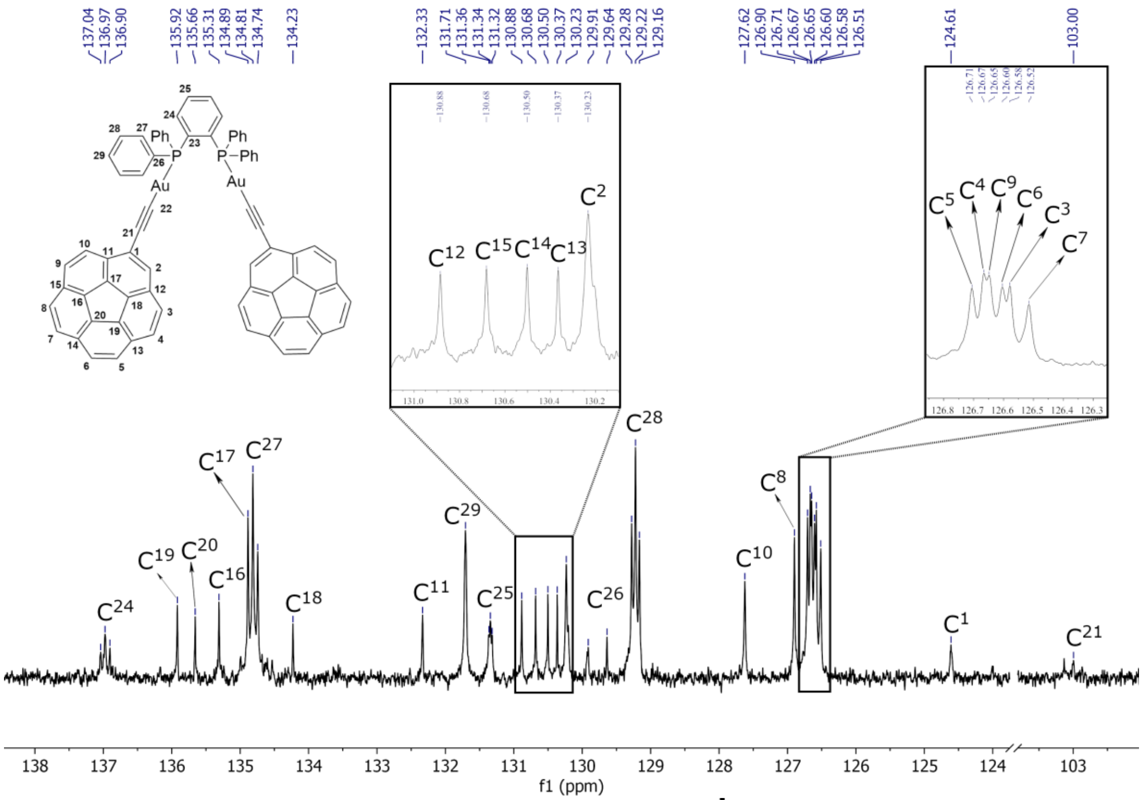

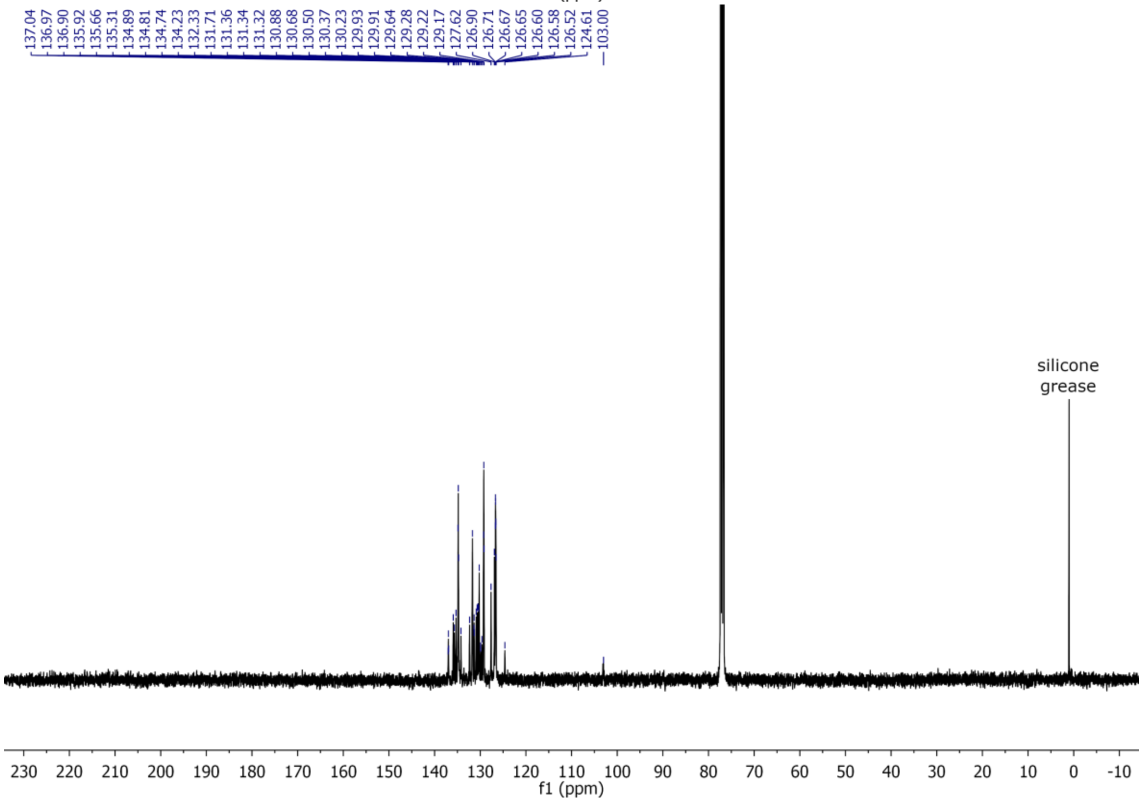

Figure S65. $^{13}C\{^{1}H\}$ NMR spectrum of compound CAudppbenz (101 MHz, $CDCl_3$); selected regions (above) and full spectrum (below).

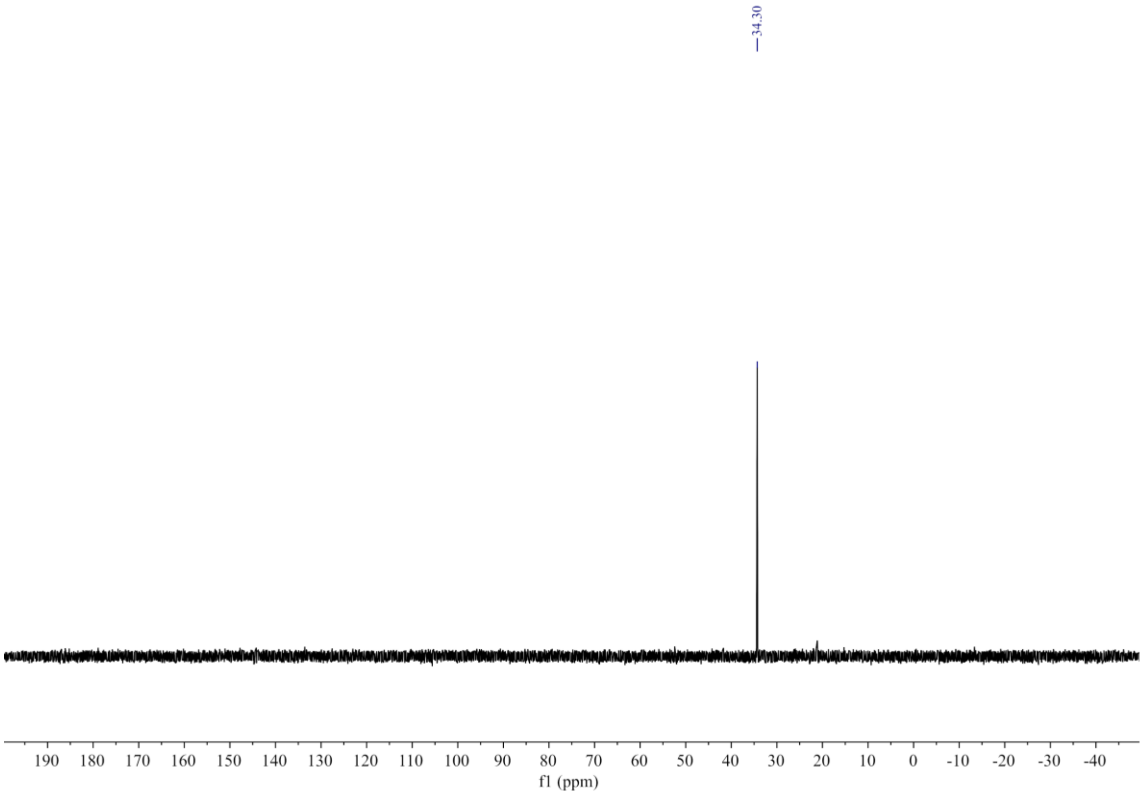

Figure S66. ^{31}P NMR spectrum of compound CAudppbenz (162 MHz, $CDCl_3$).

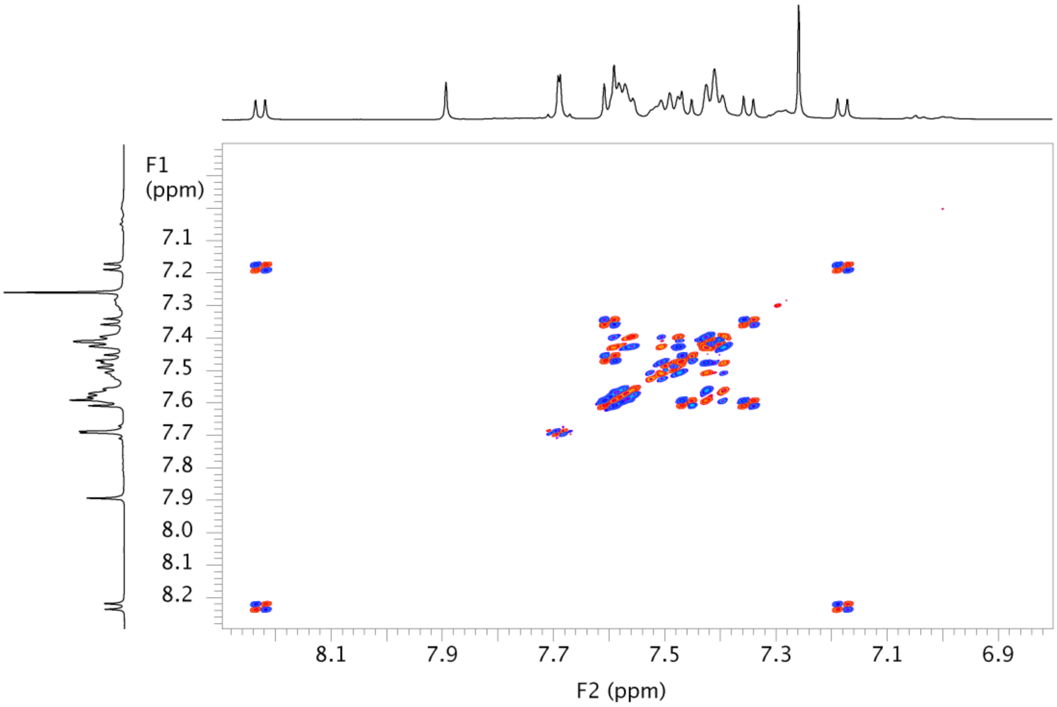

Figure S67. ^{1}H-^{1}H gDQFCOSY spectrum of compound CAudppbenz (500 MHz, $CDCl_3$).

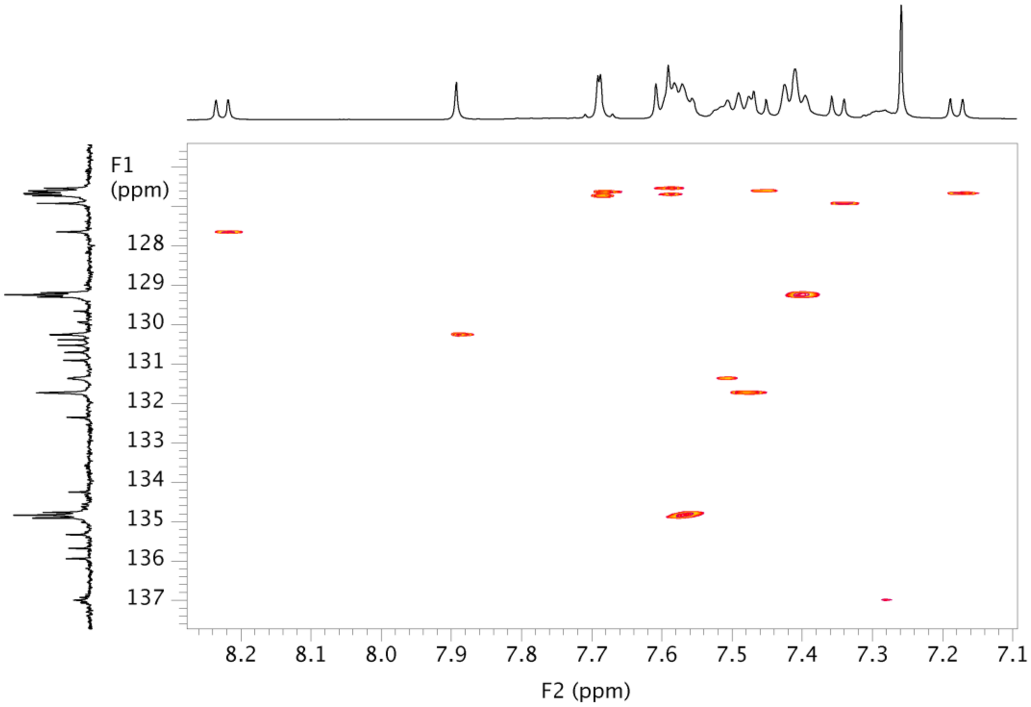

Figure S68. ^{1}H-^{13}C bsgHSQCAD spectrum of compound CAudppbenz (500 MHz, $CDCl_3$).

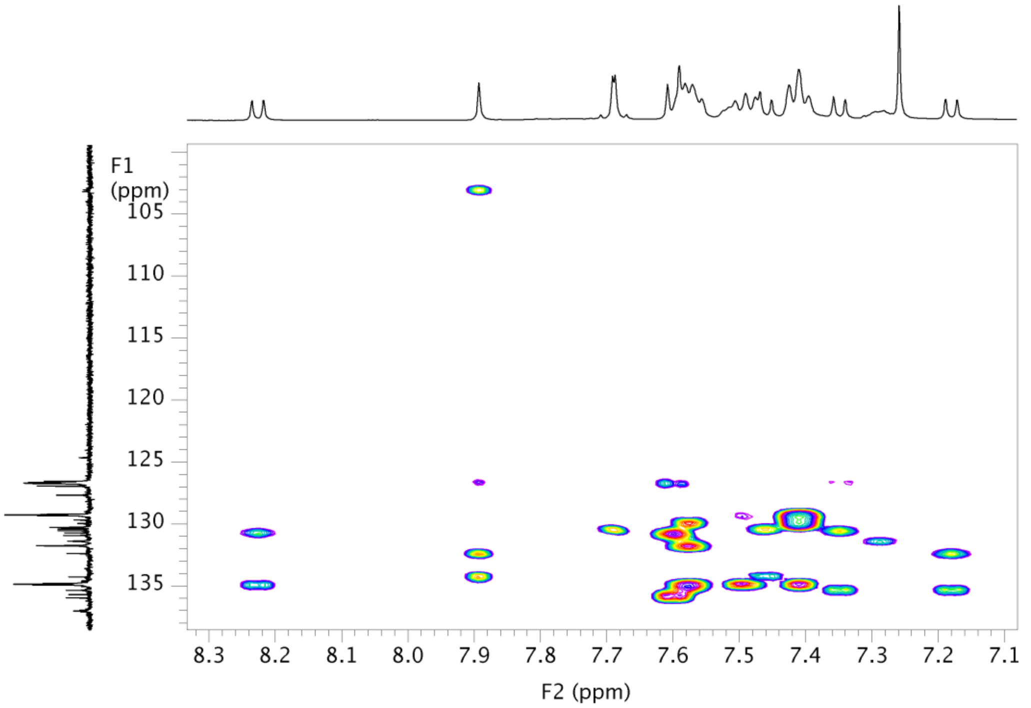

Figure S69. ^{1}H-^{13}C gc2hmbc spectrum of compound CAudppbenz (500 MHz, $CDCl_3$).

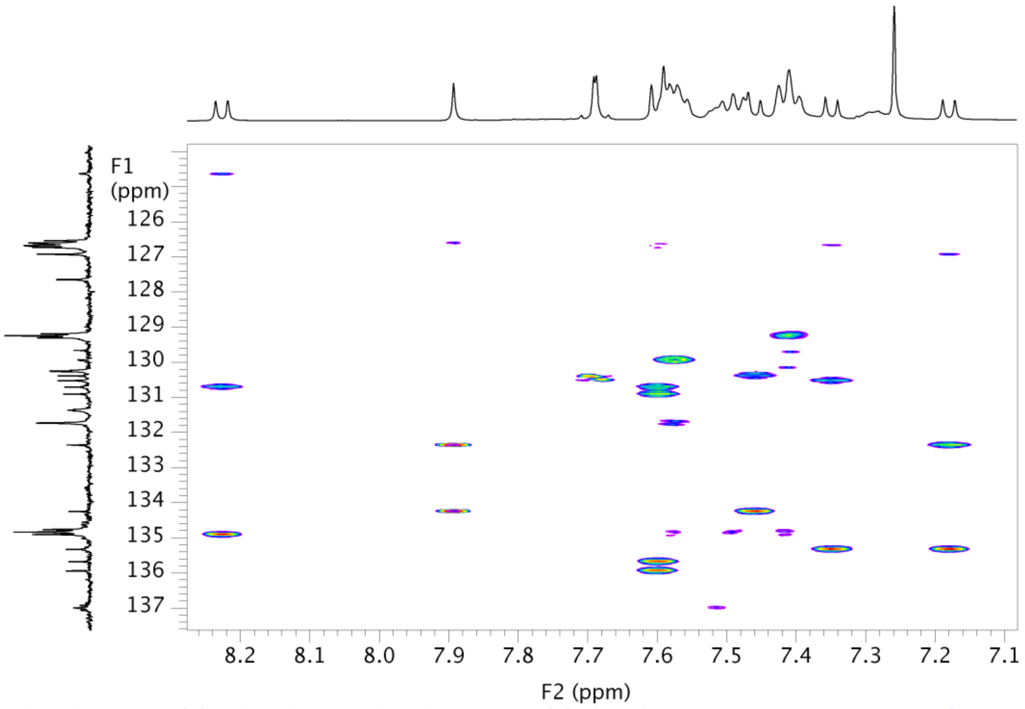

Figure S70. ^{1}H-^{13}C bsgHMBC spectrum of compound CAudppbenz (500 MHz, $CDCl_3$).

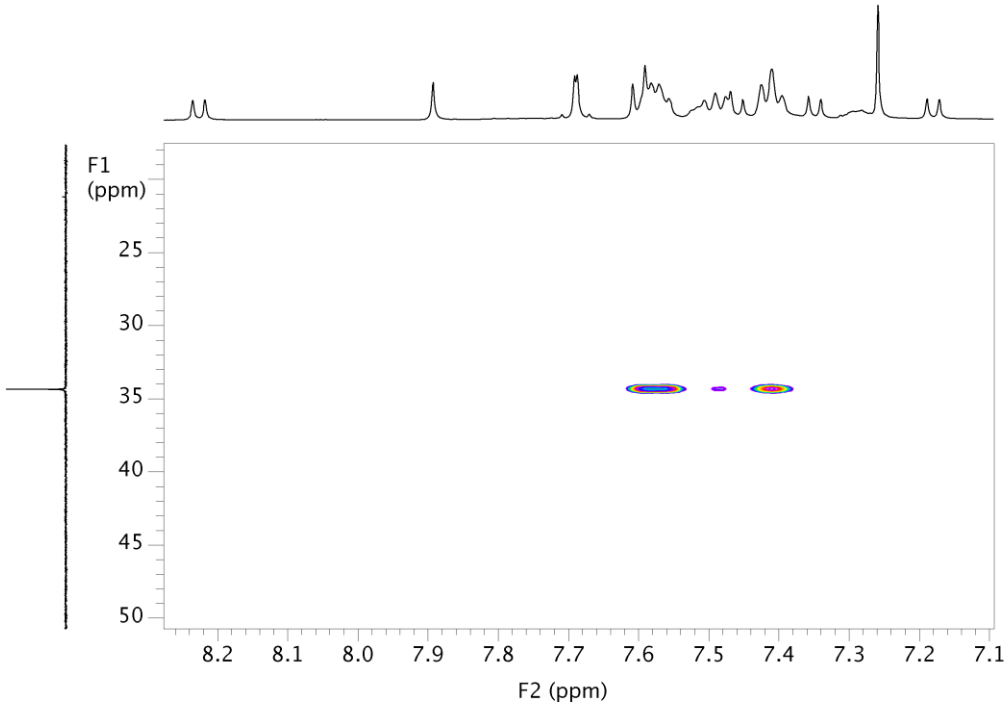

Figure S71. ^{1}H-^{31}P gHMBCAD spectrum of compound CAudppbenz (500 MHz, $CDCl_3$).

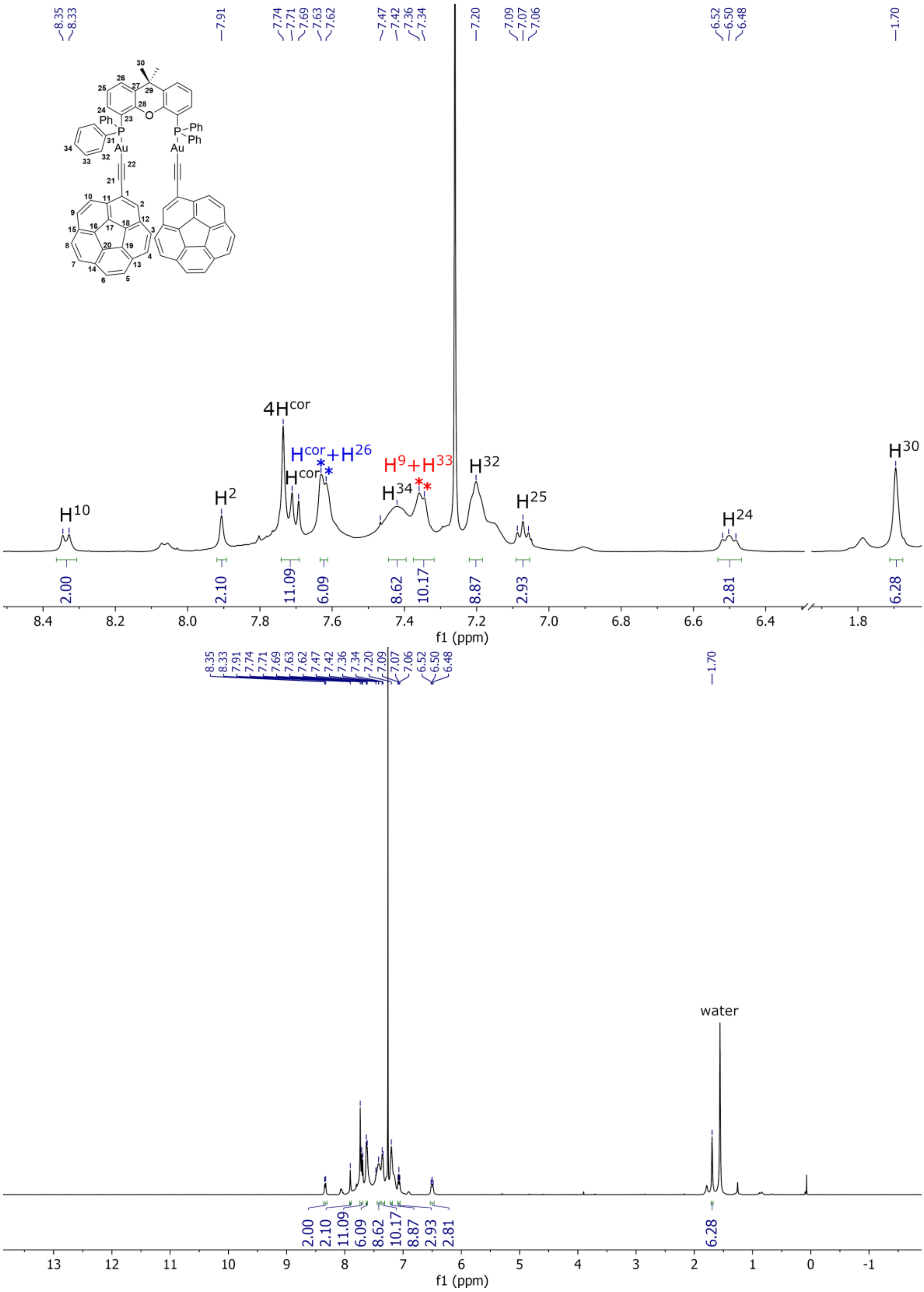

Figure S72. ^{1}H NMR spectrum of compound CAuxantphos (500 MHz, $CDCl_3$); selected regions (above) and full spectrum (below). Some signals are depicted in different colors for clarity purposes.

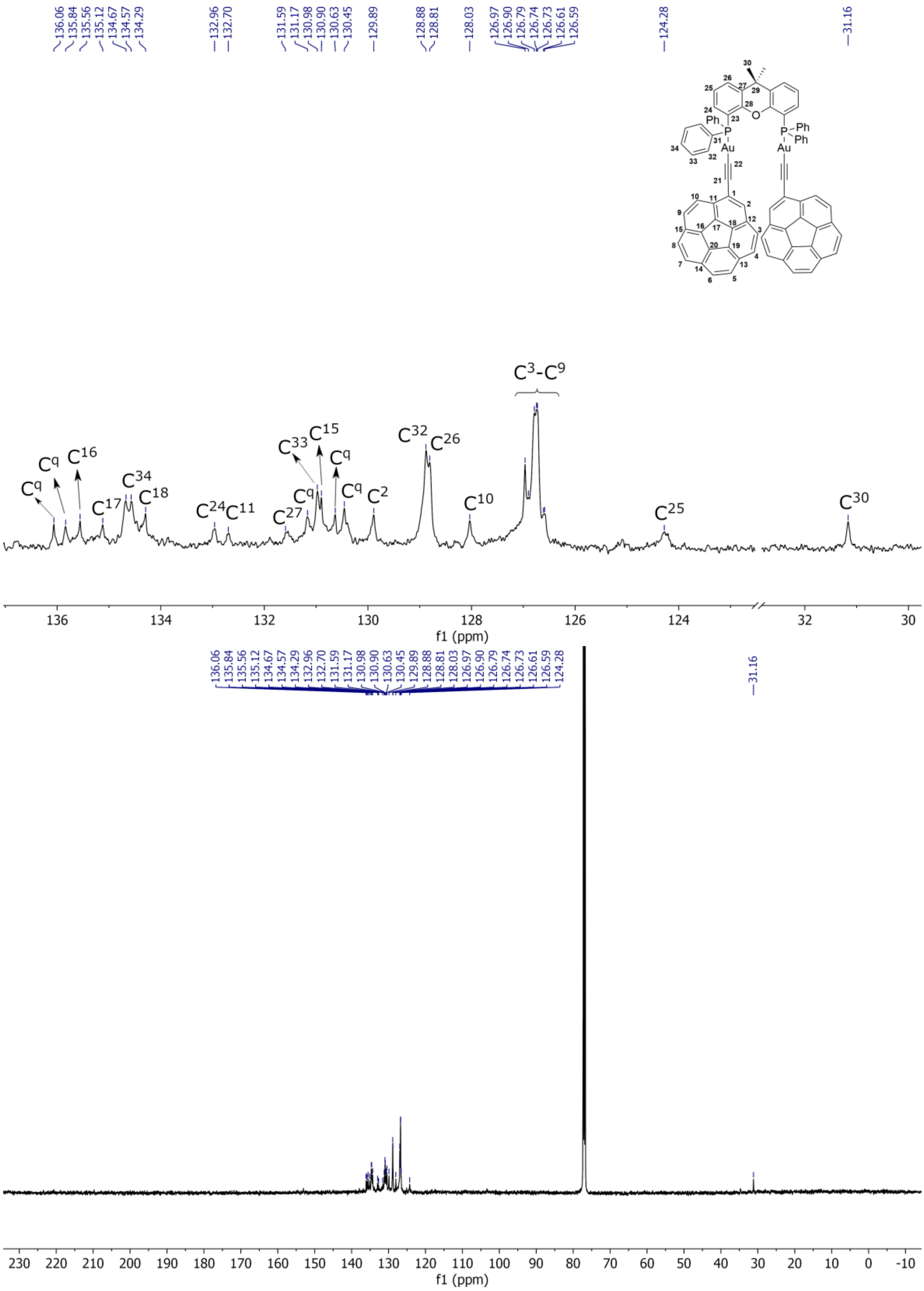

Figure S73. $^{13}C\{^1H\}$ NMR spectrum of compound CAuxantphos (126 MHz, $CDCl_3$); selected regions (above) and full spectrum (below).

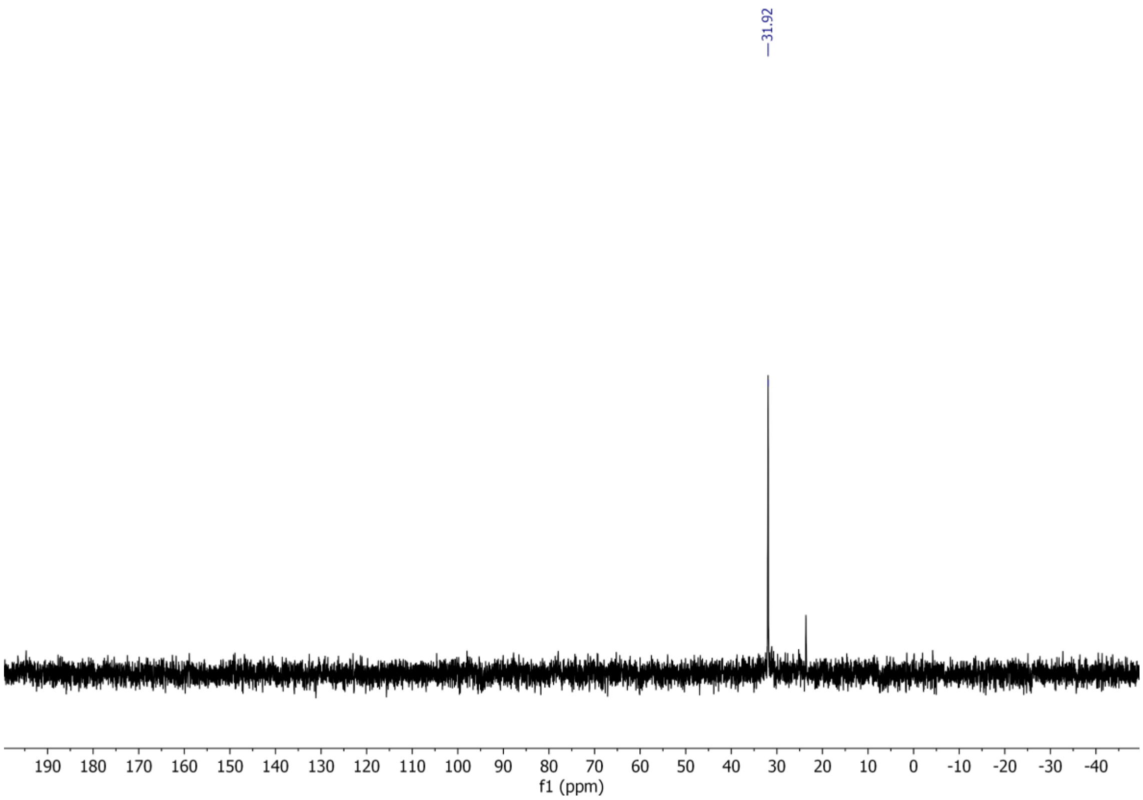

Figure S74. ^{31}P NMR spectrum of compound CAuxantphos (162 MHz, $CDCl_3$).

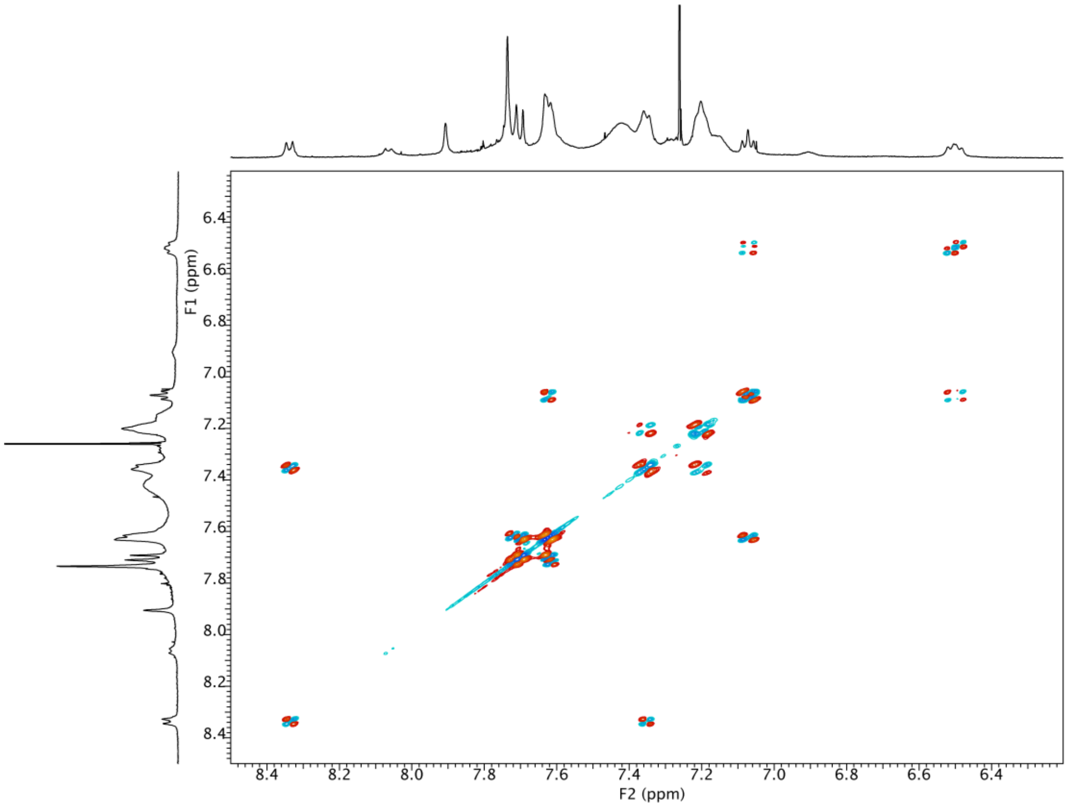

Figure S75. ^{1}H-^{1}H gDQFCOSY spectrum of compound CAuxantphos (500 MHz, $CDCl_3$).

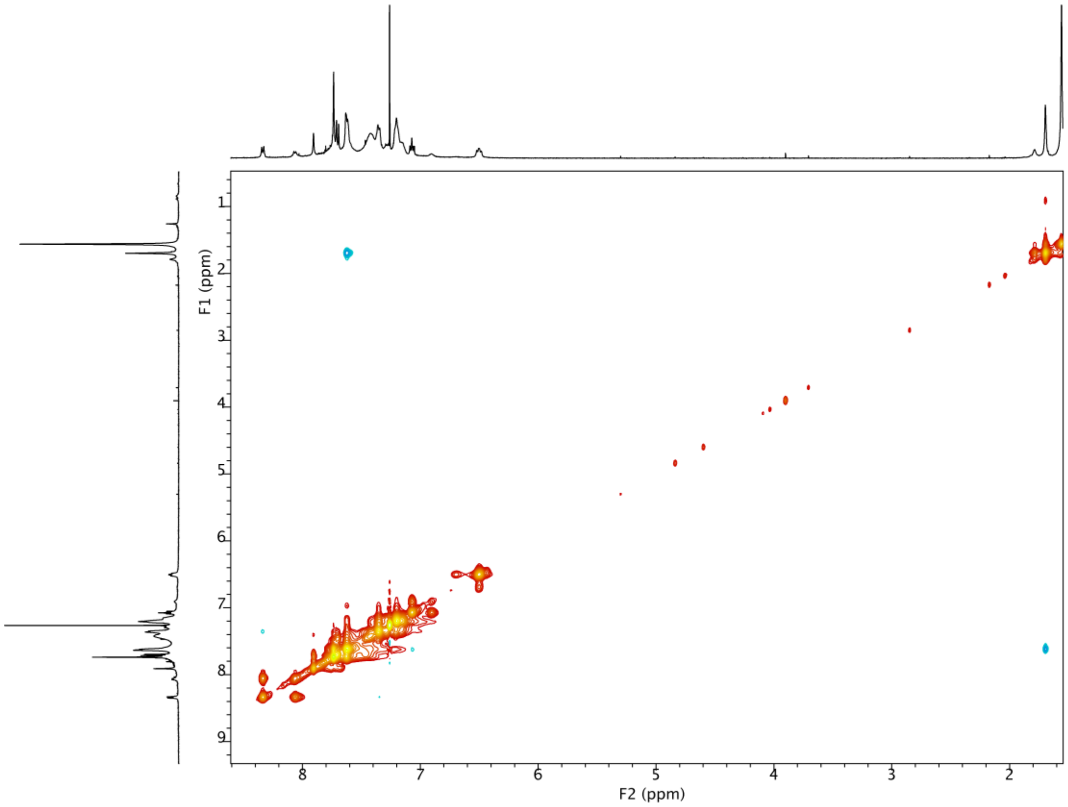

Figure S76. ^{1}H-^{1}H ROESYAD spectrum of compound CAuxantphos (500 MHz, $CDCl_3$).

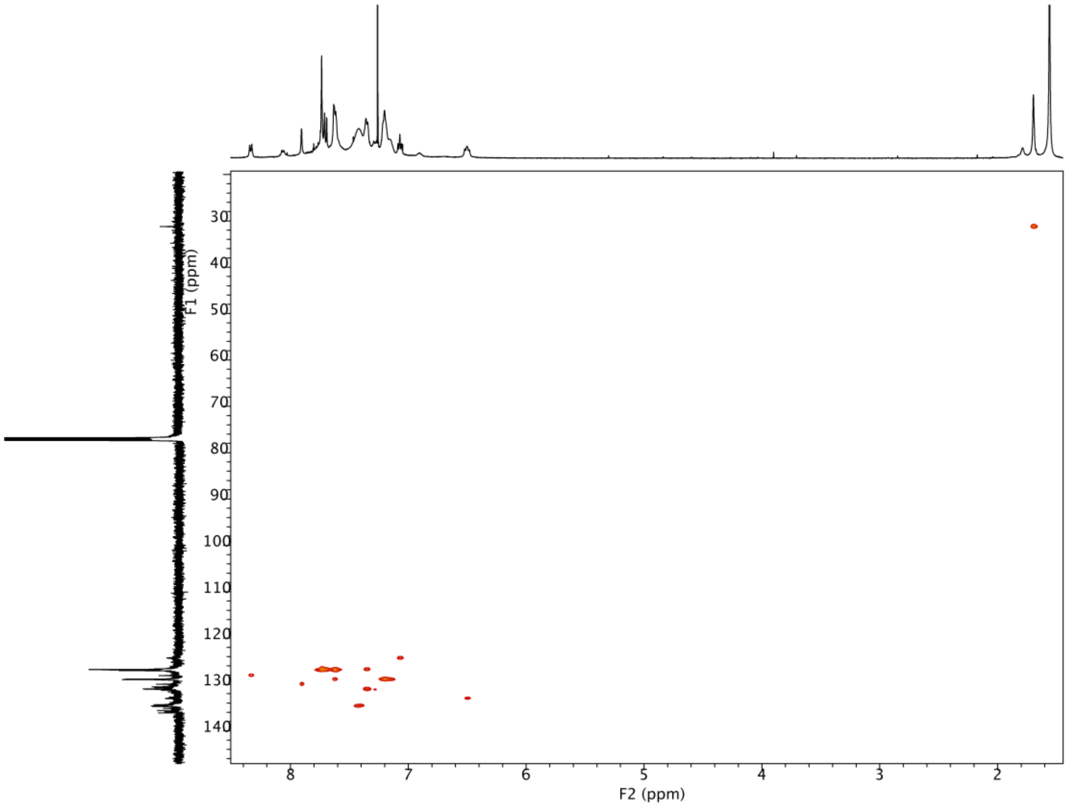

Figure S77. ^{1}H-^{13}C gc2hsqc spectrum of compound CAuxantphos (500 MHz, $CDCl_3$).

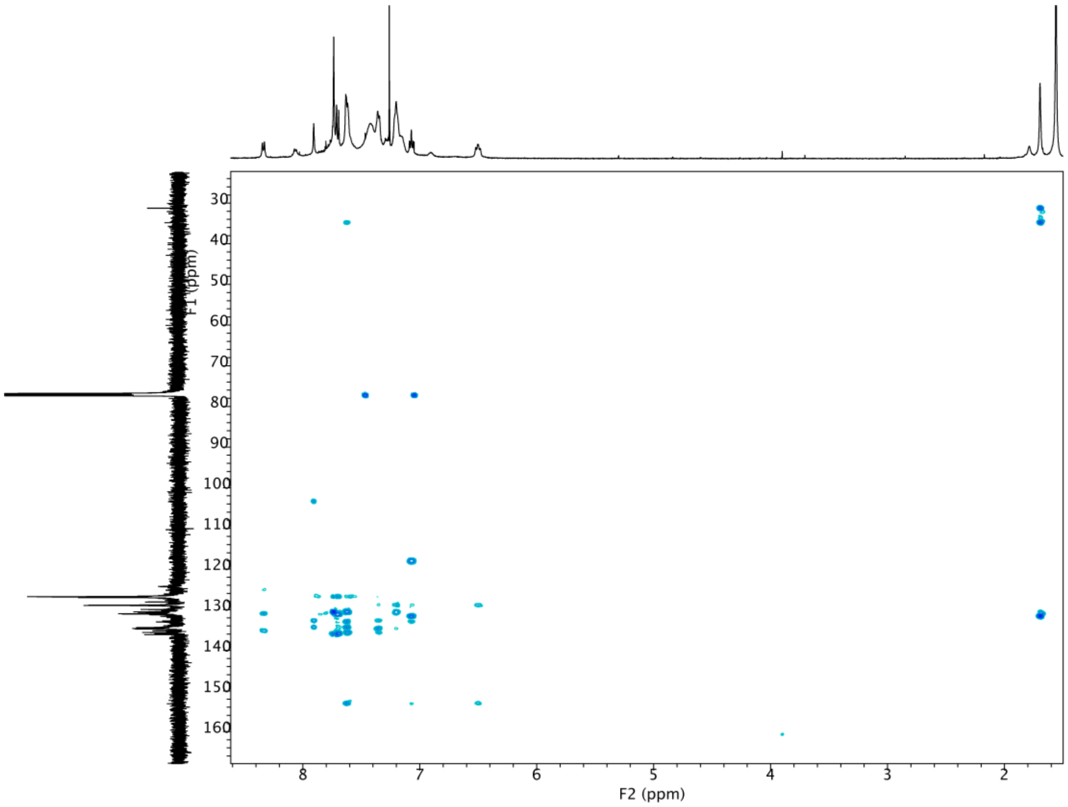

Figure S78. ^{1}H-^{13}C gc2hmbc spectrum of compound CAuxantphos (500 MHz, $CDCl_3$).

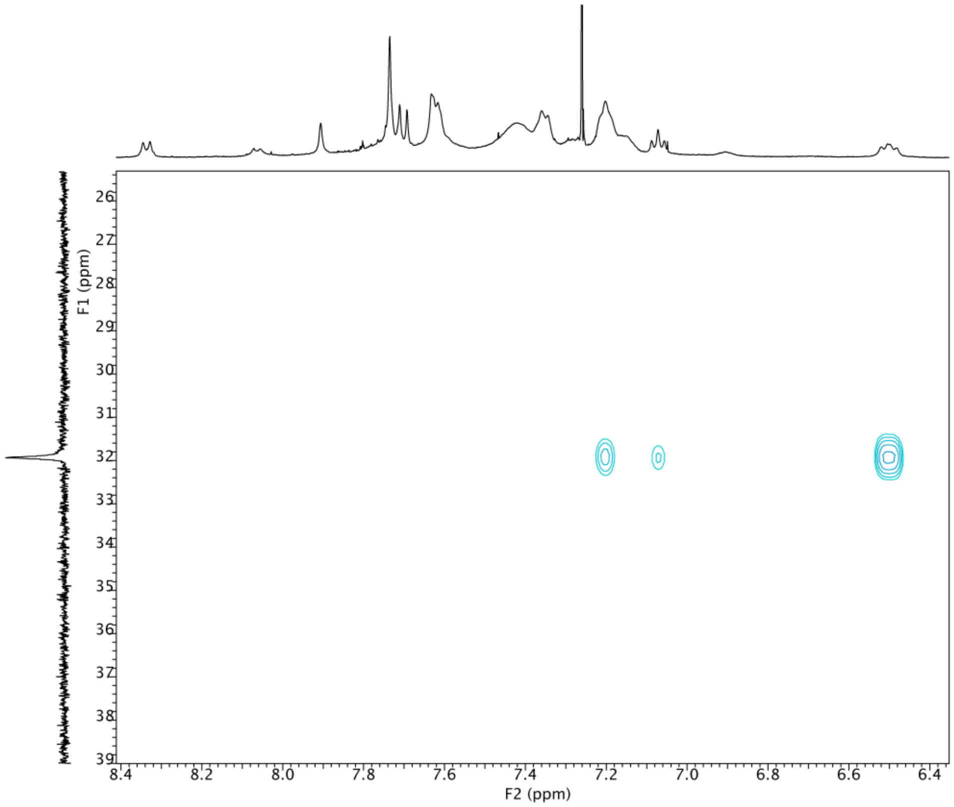

Figure S79. ^{1}H-^{31}P gHMBCAD spectrum of compound CAuxantphos (500 MHz, $CDCl_3$).

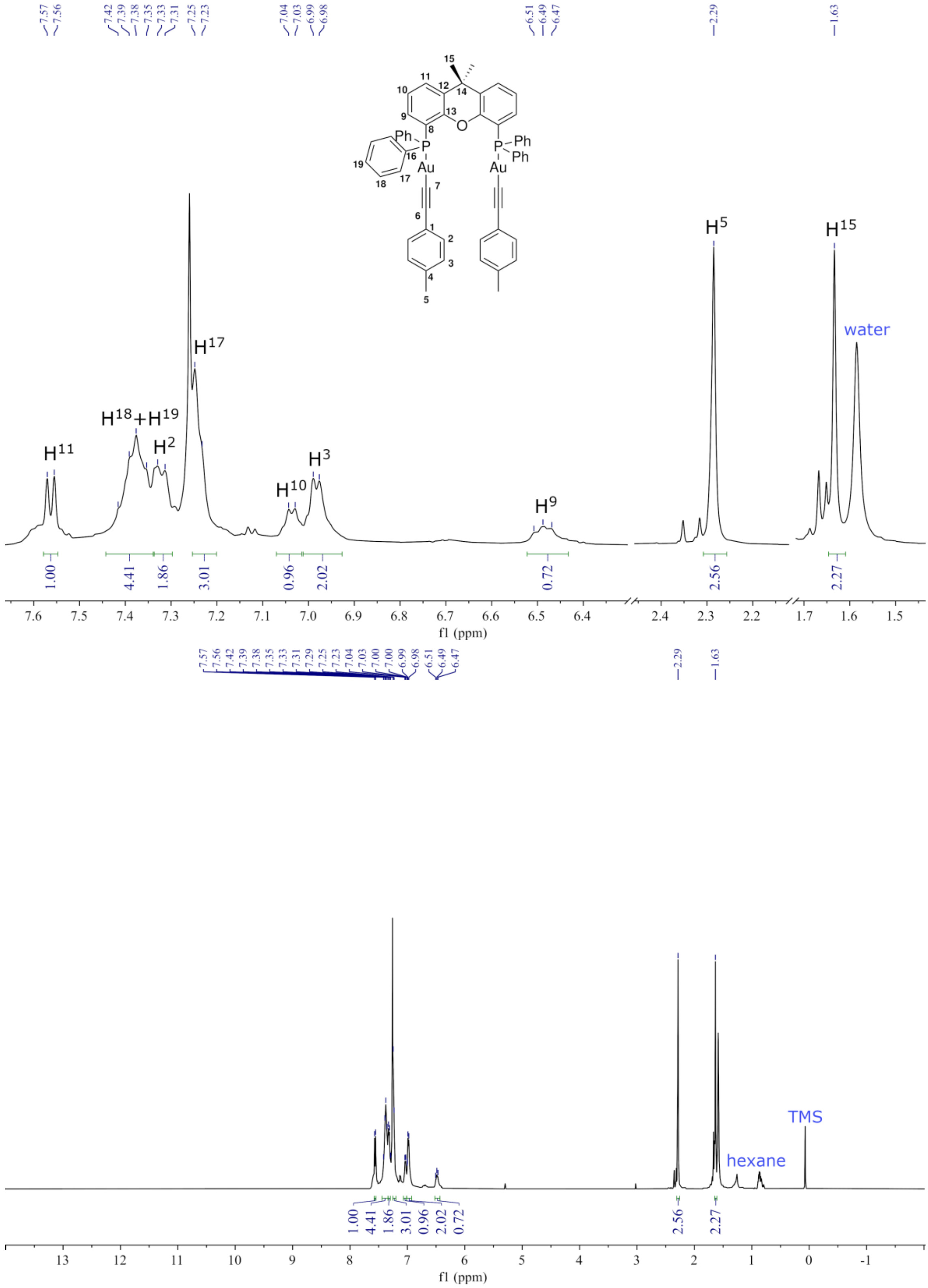

Figure S80. ^{1}H NMR spectrum of compound *p*-tolylAuxantphos (500 MHz, $CDCl_3$); selected regions (above) and full spectrum (below).

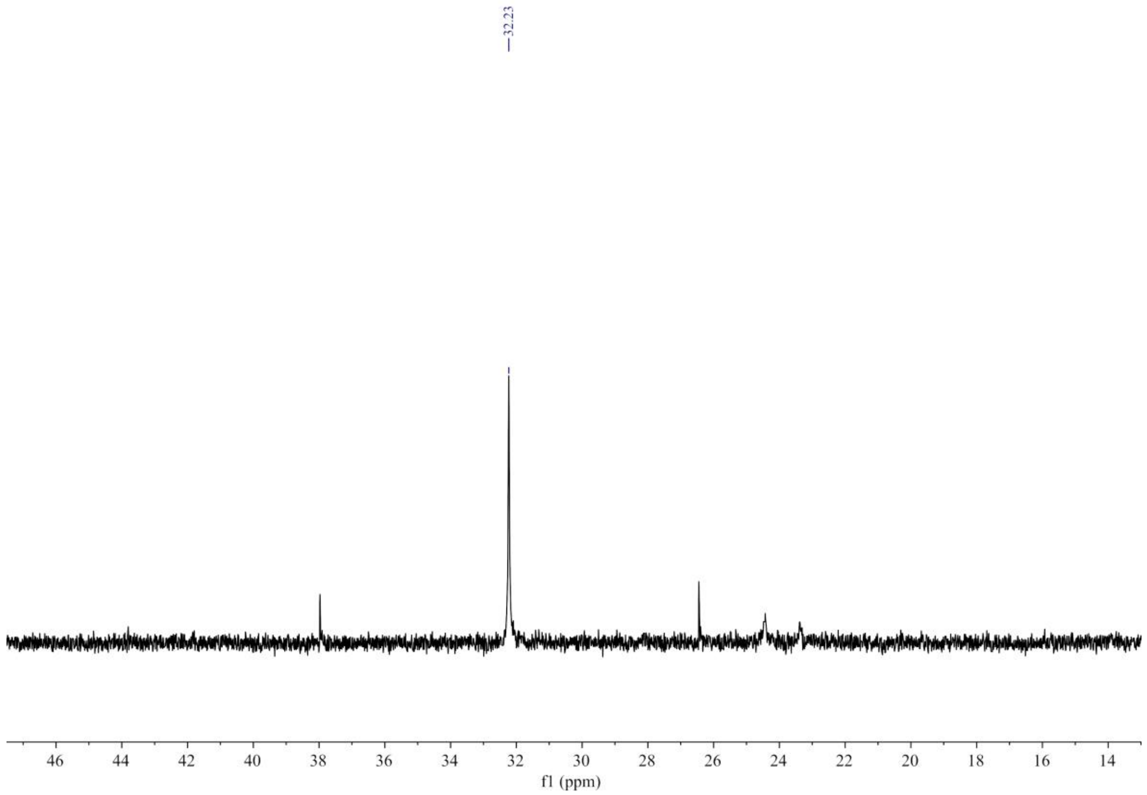

Figure S81. ^{31}P NMR spectrum of compound *p*-tolylAuxantphos (202 MHz, $CDCl_3$).

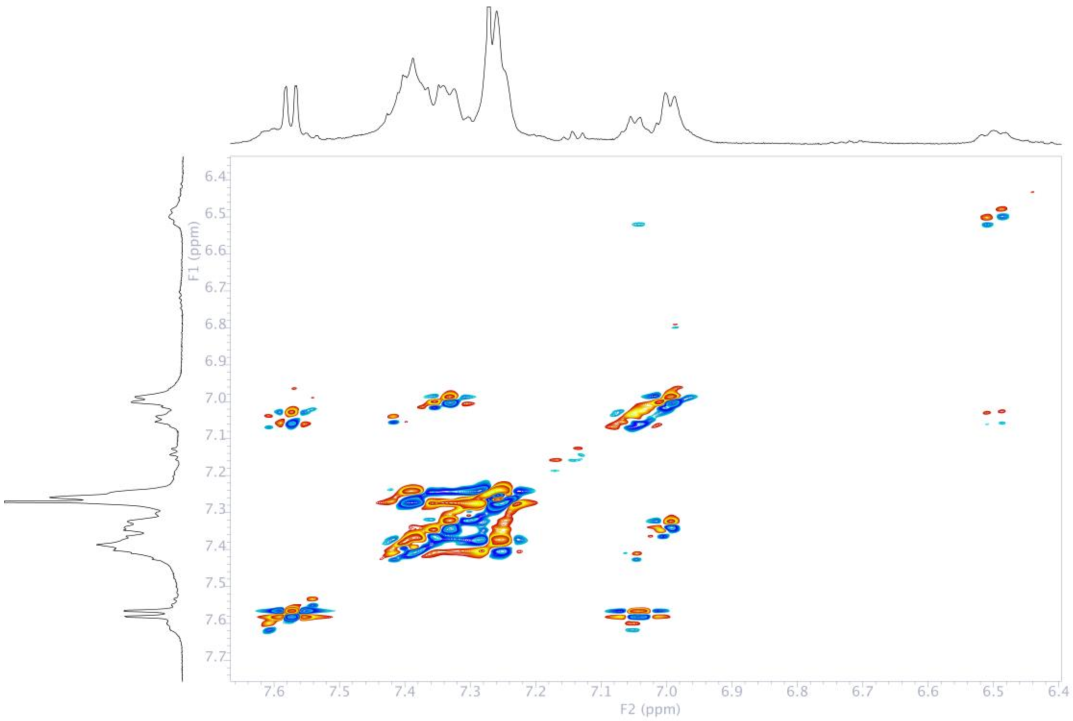

Figure S82. ^{1}H-^{1}H gDQFCOSY spectrum of compound *p*-tolylAuxantphos (500 MHz, $CDCl_3$).

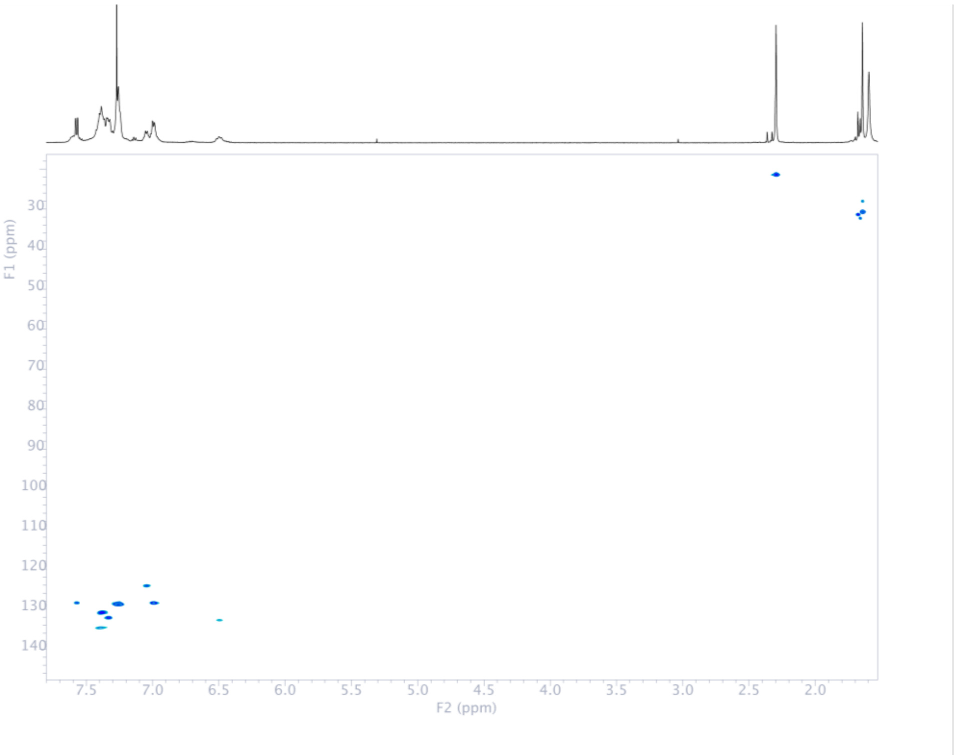

Figure S83. ^{1}H-^{13}C gc2hsqc spectrum of compound *p*-tolylAuxantphos (500 MHz, $CDCl_3$).

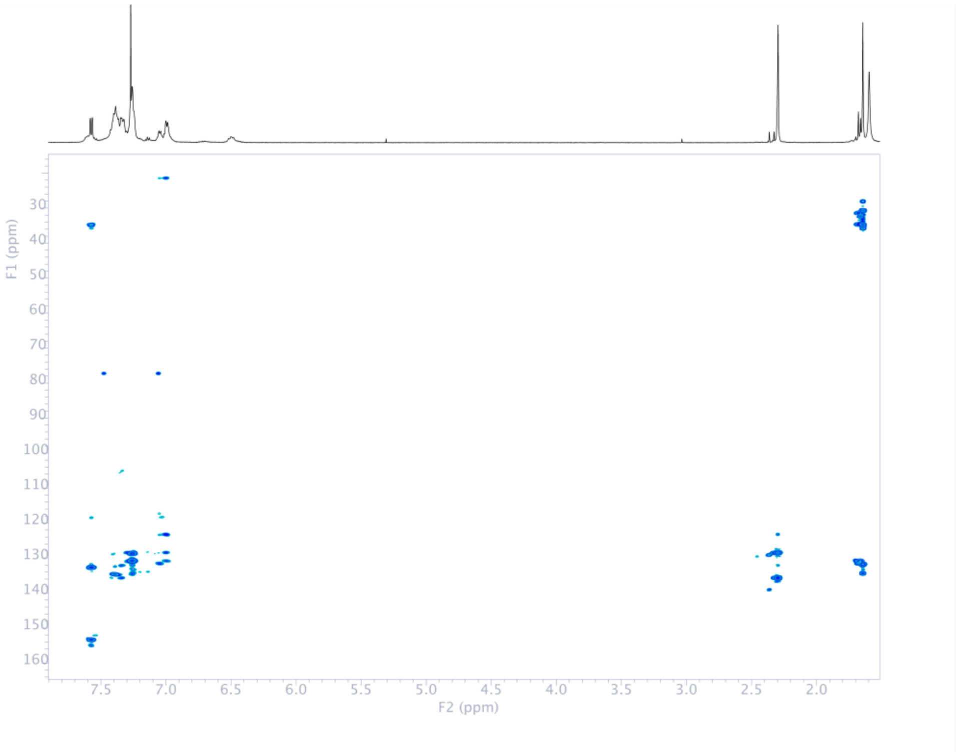

Figure S84. ^{1}H-^{13}C gc2hmbc spectrum of compound *p*-tolylAuxantphos (500 MHz, $CDCl_3$).

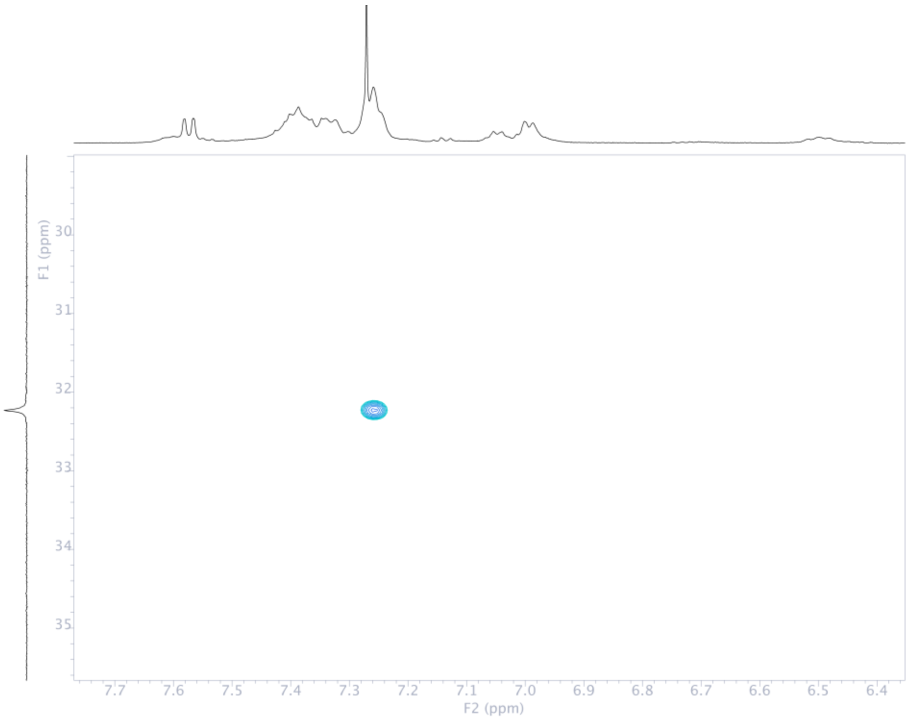

Figure S85. ^{1}H-^{31}P gHMBCAD spectrum of compound *p*-tolylAuxantphos (500 MHz, $CDCl_3$).

^{1}H-NMR VT Experiments

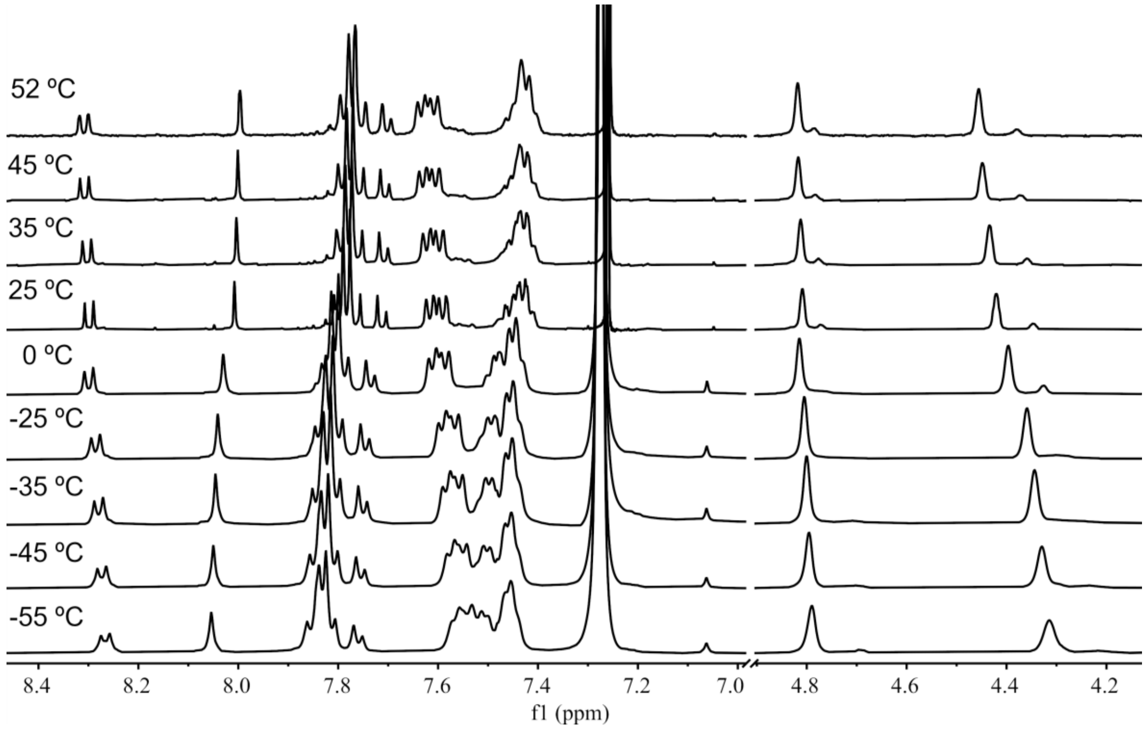

Figure S86. ^{1}H-NMR VT experiments (500 MHz, $CDCl_3$) of complex CAudppf.

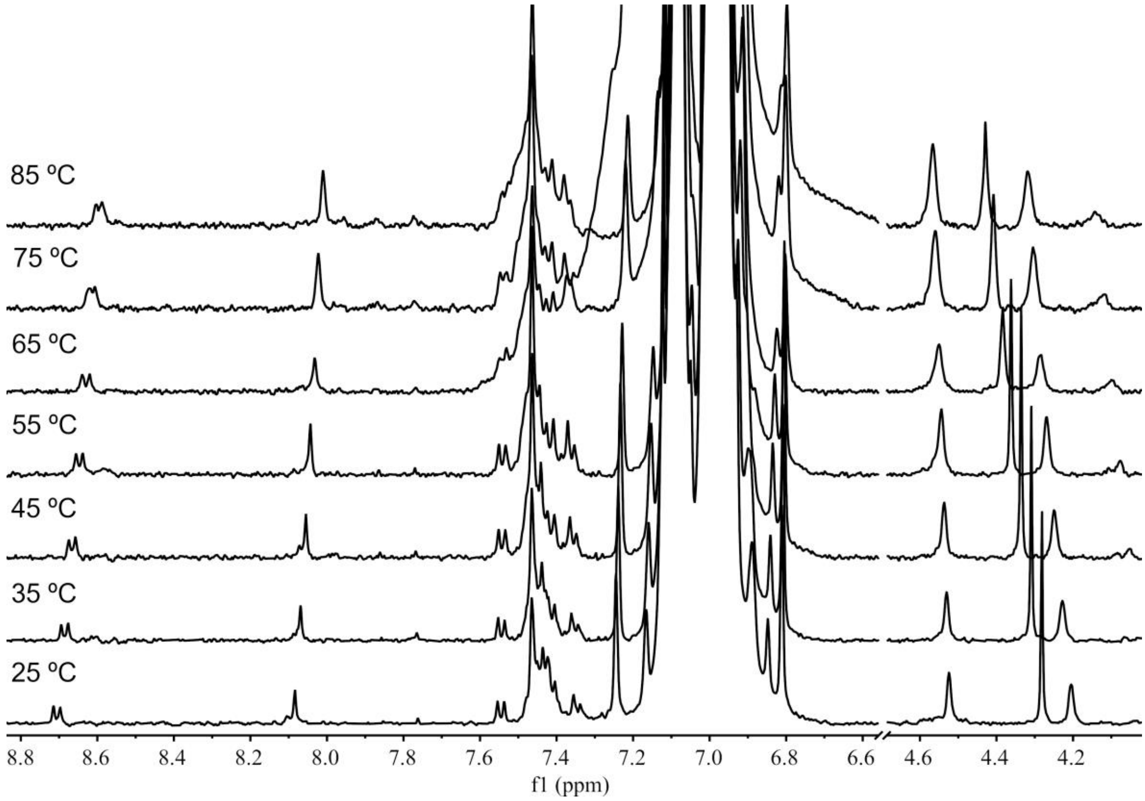

Figure S87. ^{1}H-NMR VT experiments (500 MHz, tol-d_8) of complex CAudppf.

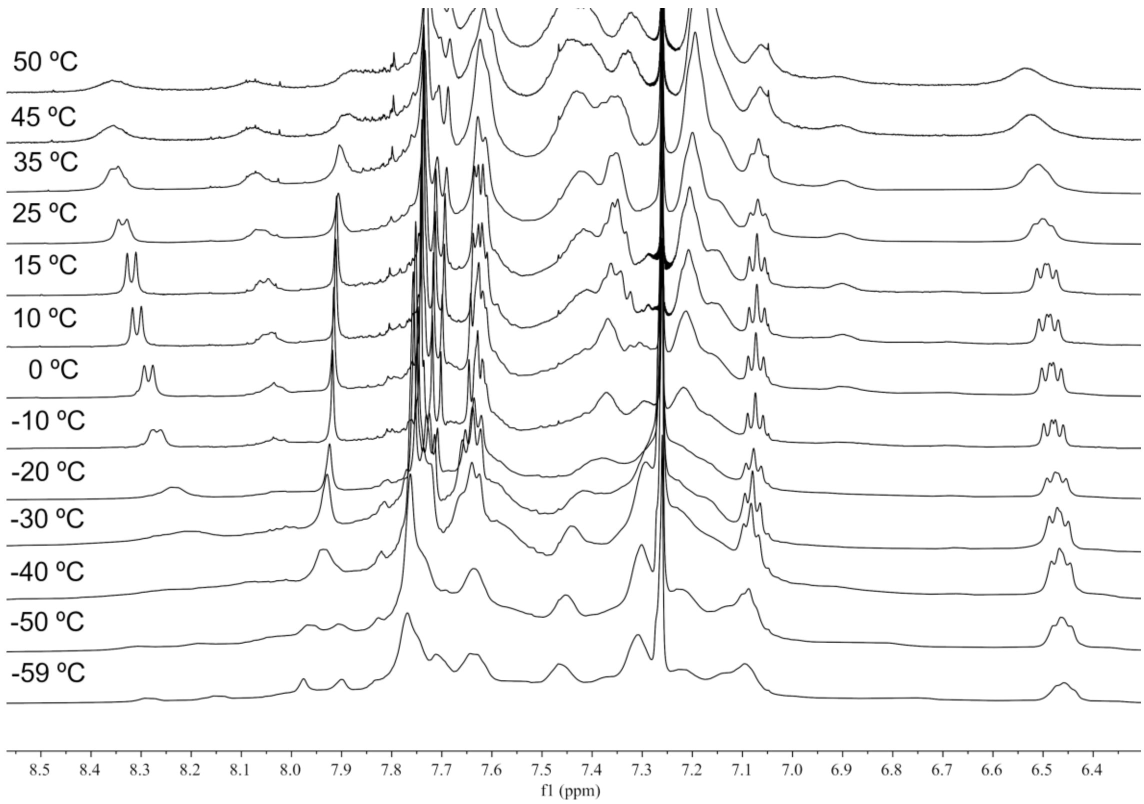

Figure S88. Full ^{1}H-NMR VT experiments (500 MHz, $CDCl_3$) of complex CAuxantphos.

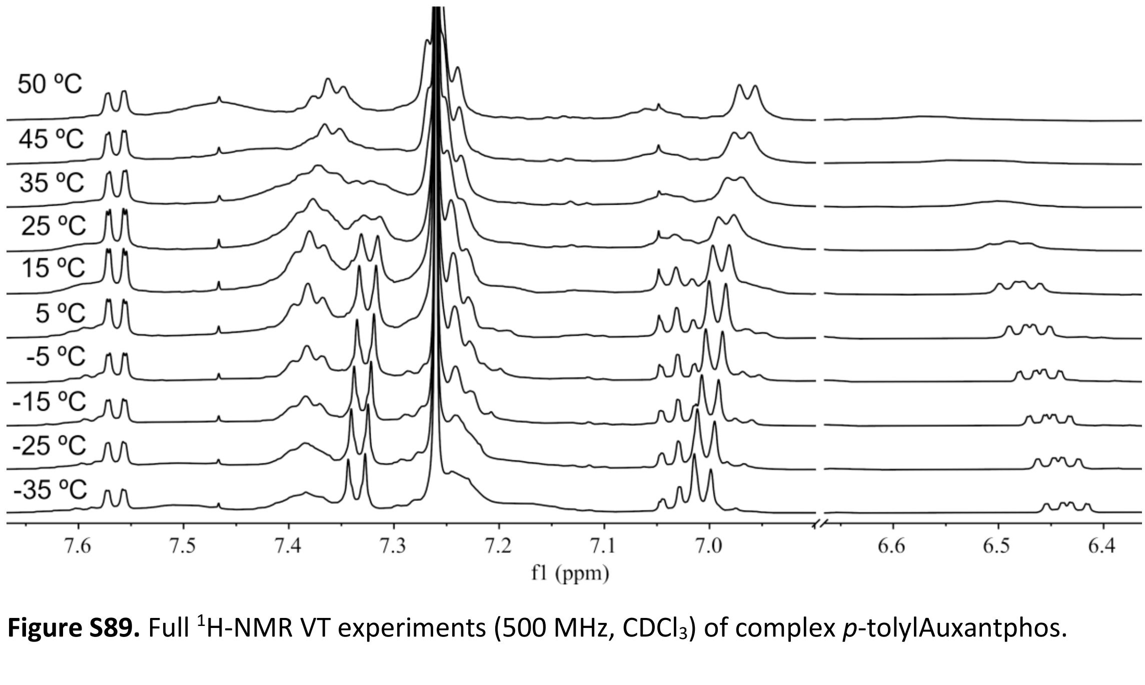

Figure S89. Full ^{1}H-NMR VT experiments (500 MHz, $CDCl_3$) of complex *p*-tolylAuxantphos.

High Resolution Mass Spectra

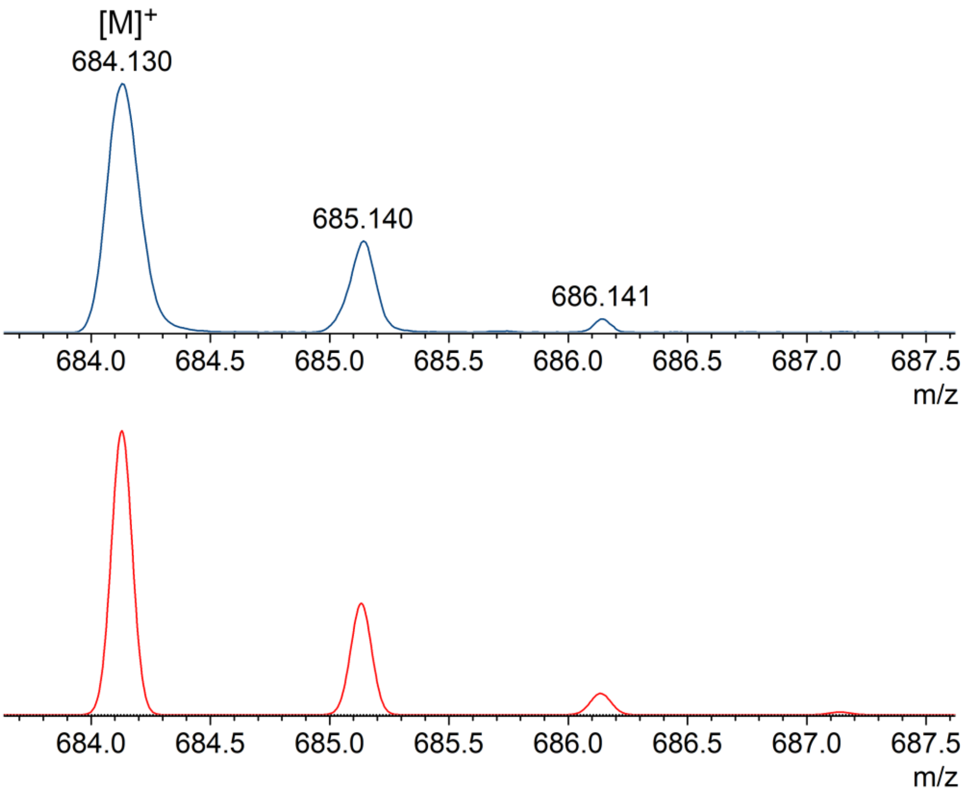

Figure S90. HRMS (MALDI-TOF) for compound PAuPPh$_3$ [M]$^+$. Calculated (red), measured (blue).

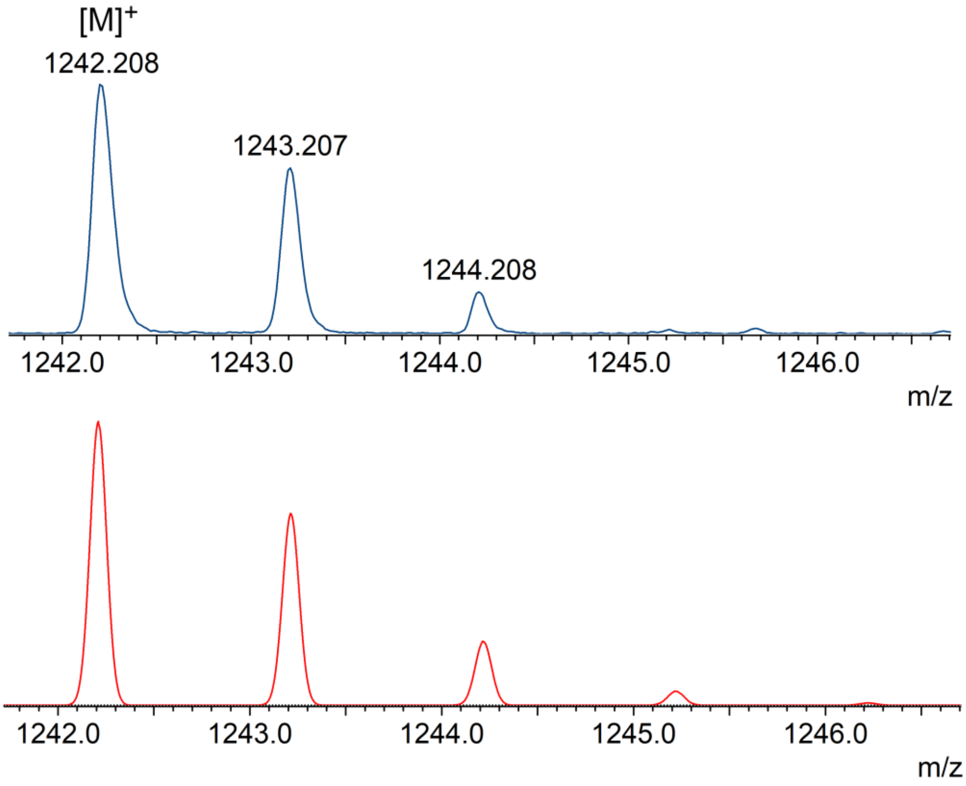

Figure S91. HRMS (MALDI-TOF) for compound PAudppe [M]$^+$. Calculated (red), measured (blue).

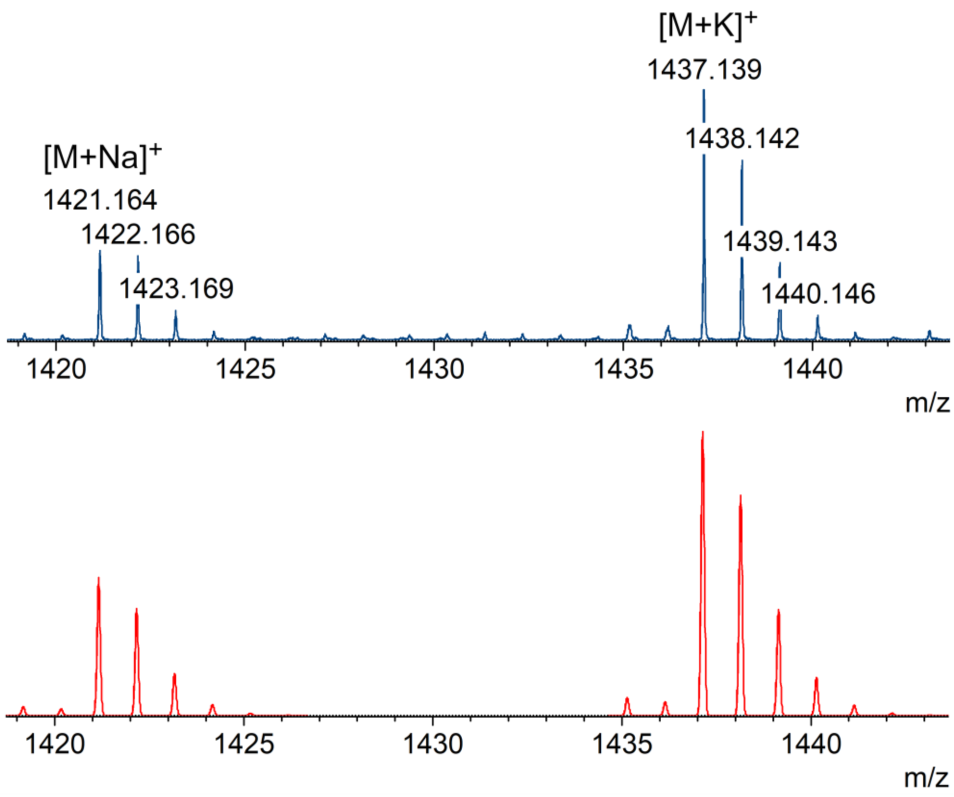

Figure S92. HRMS (ESI-TOF) for compound PAudppf [M+Na]$^{+}$ and [M+K]$^{+}$. Calculated (red), measured (blue).

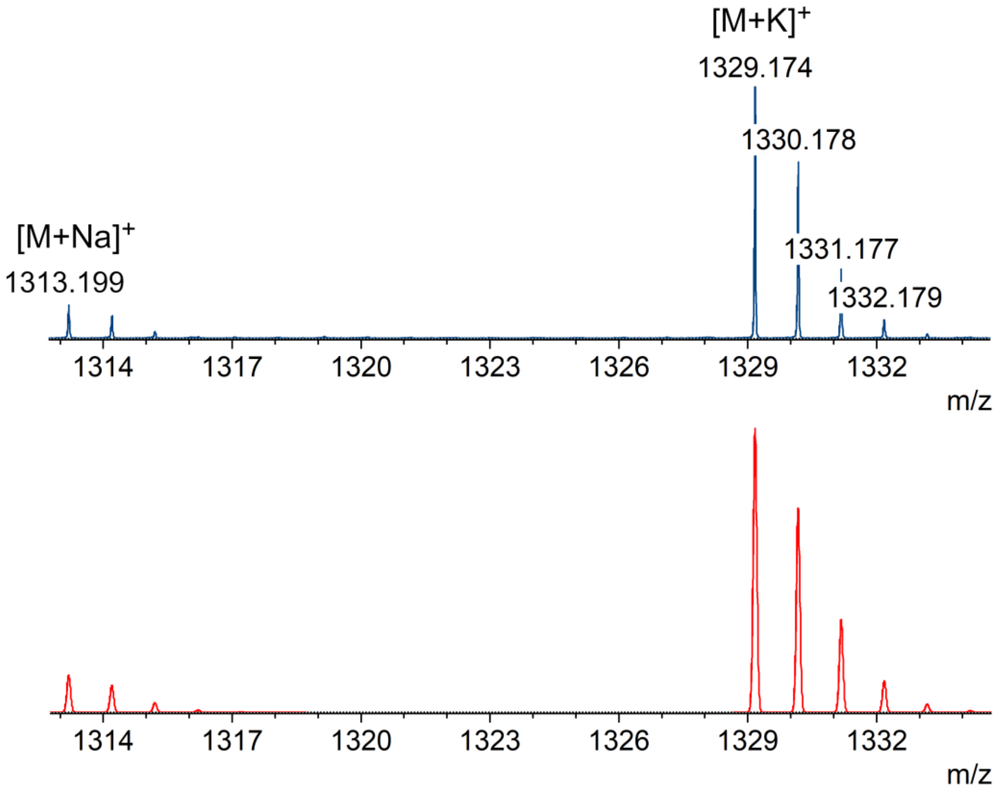

Figure S93. HRMS (ESI-TOF) for compound PAudppbenz [M+Na]$^{+}$ and [M+K]$^{+}$. Calculated (red), measured (blue).

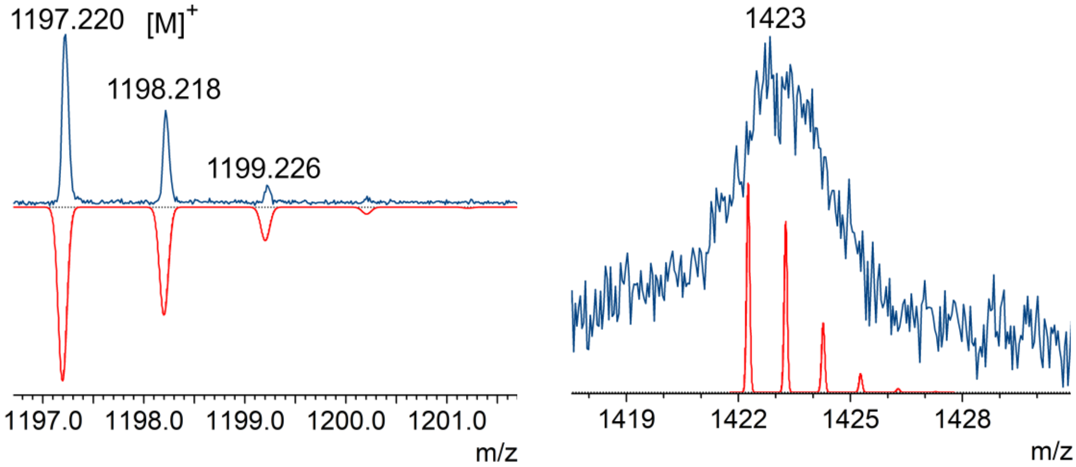

Figure S94. HRMS (ESI-TOF) for compound PAuxantphos – ethynylpyrene $[M]^+$ (left) and LRMS (MALDI-TOF) for compound PAuxantphos $[M]^+$ (right). Calculated (red), measured (blue).

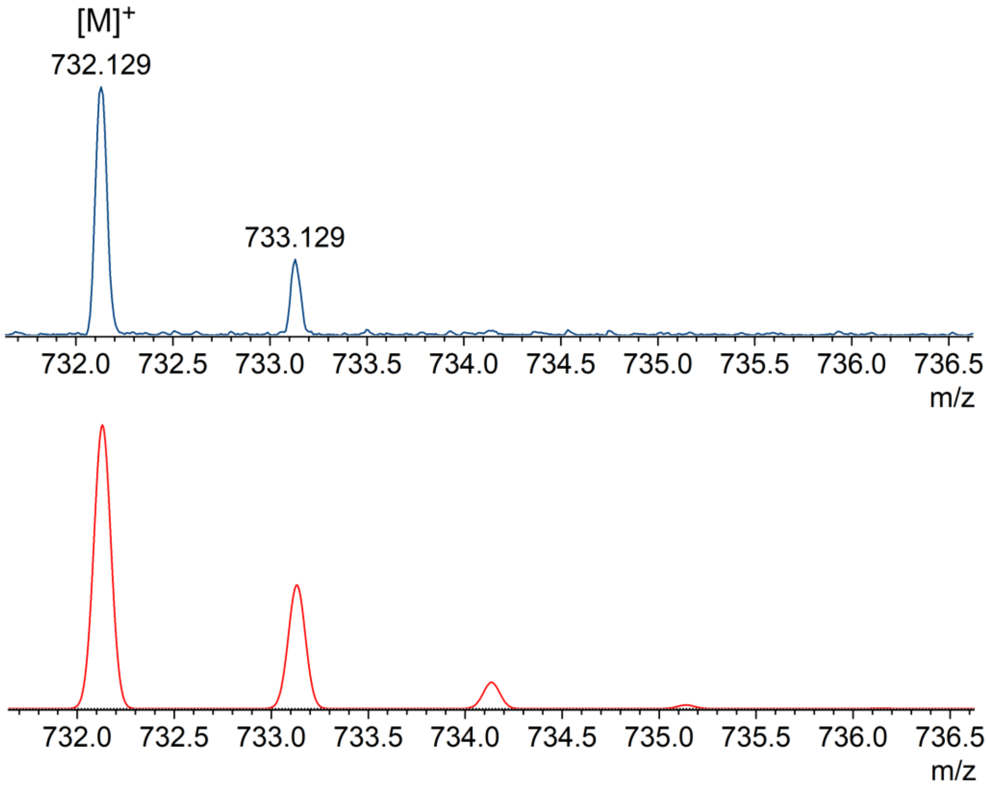

Figure S95. HRMS (MALDI-TOF) for compound $CAuPPh_3$ $[M]^+$. Calculated (red), measured (blue).

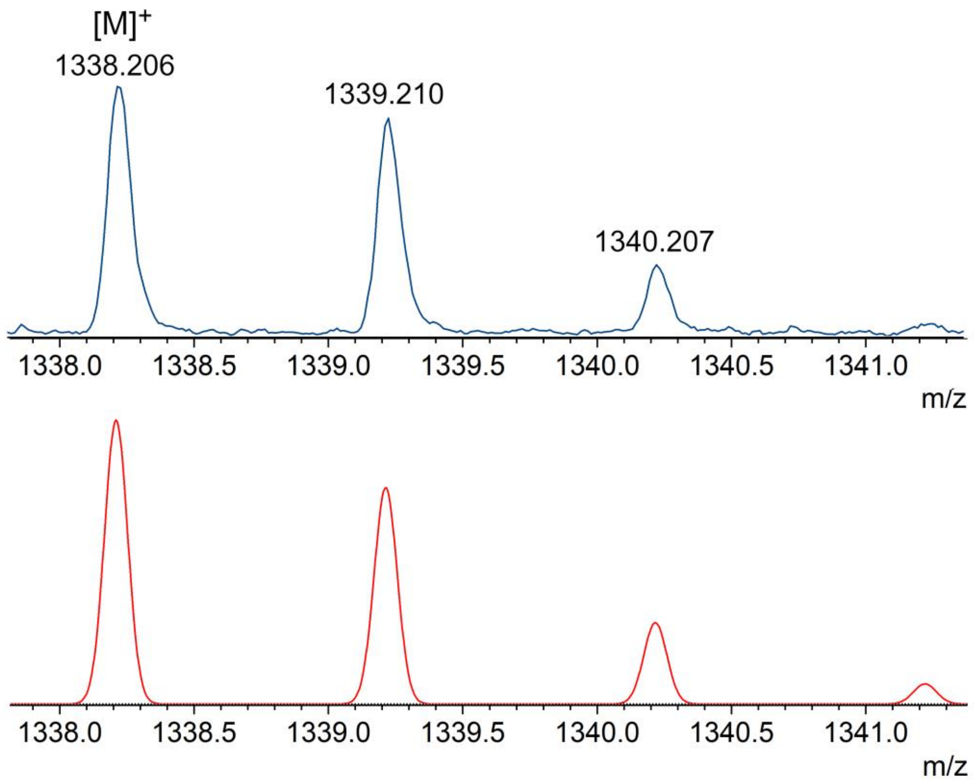

Figure S96. HRMS (MALDI-TOF) for compound CAudppe $[M]^{+}$. Calculated (red), measured (blue).

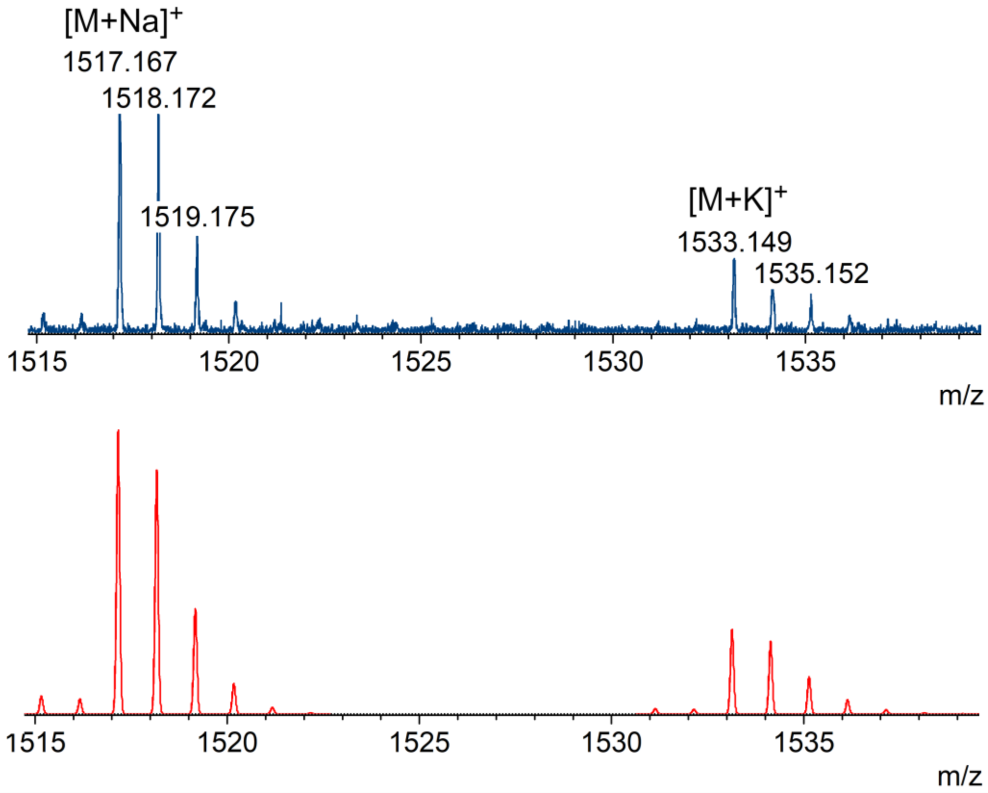

Figure S97. HRMS (ESI-QTOF) for compound CAudppf $[M+Na]^{+}$ and $[M+K]^{+}$. Calculated (red), measured (blue).

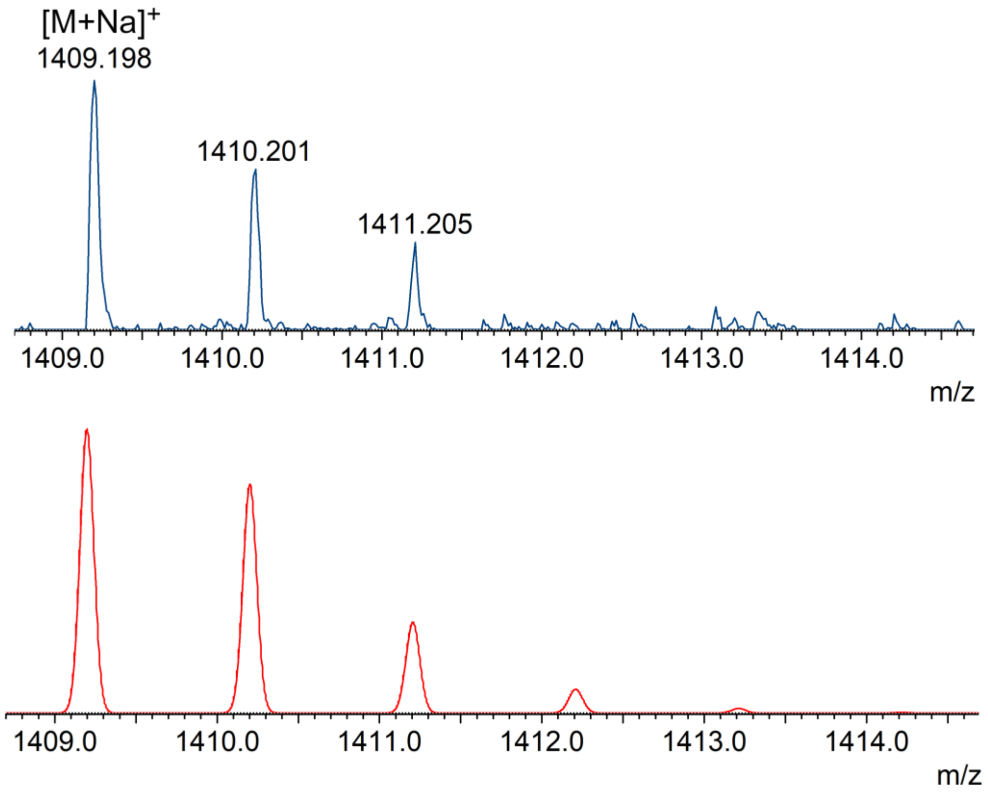

Figure S98. HRMS (ESI-QTOF) for compound CAudppbenz $[M+Na]^+$. Calculated (red), measured (blue).

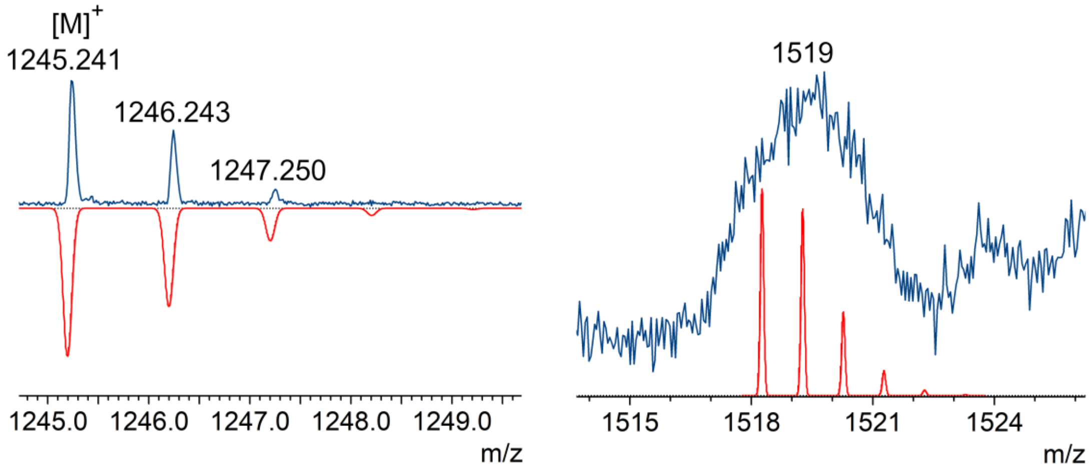

Figure S99. HRMS (ESI-TOF) for compound CAuxantphos – ethynylcorannulene $[M]^+$ (left) and LRMS (MALDI-TOF) for compound PAuxantphos [M]+ (right). Calculated (red), measured (blue).

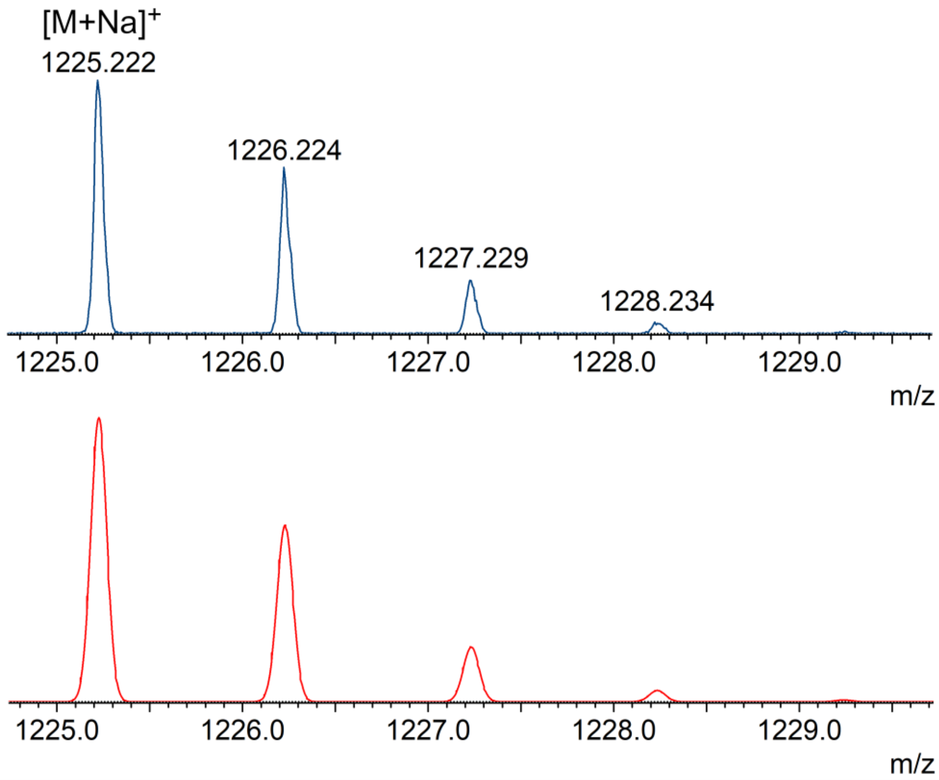

Figure S100. HRMS (ESI-QTOF) for compound *p*-tolylAuxantphos $[M+Na]^+$. Calculated (red), measured (blue).

UV-Vis and emission spectra

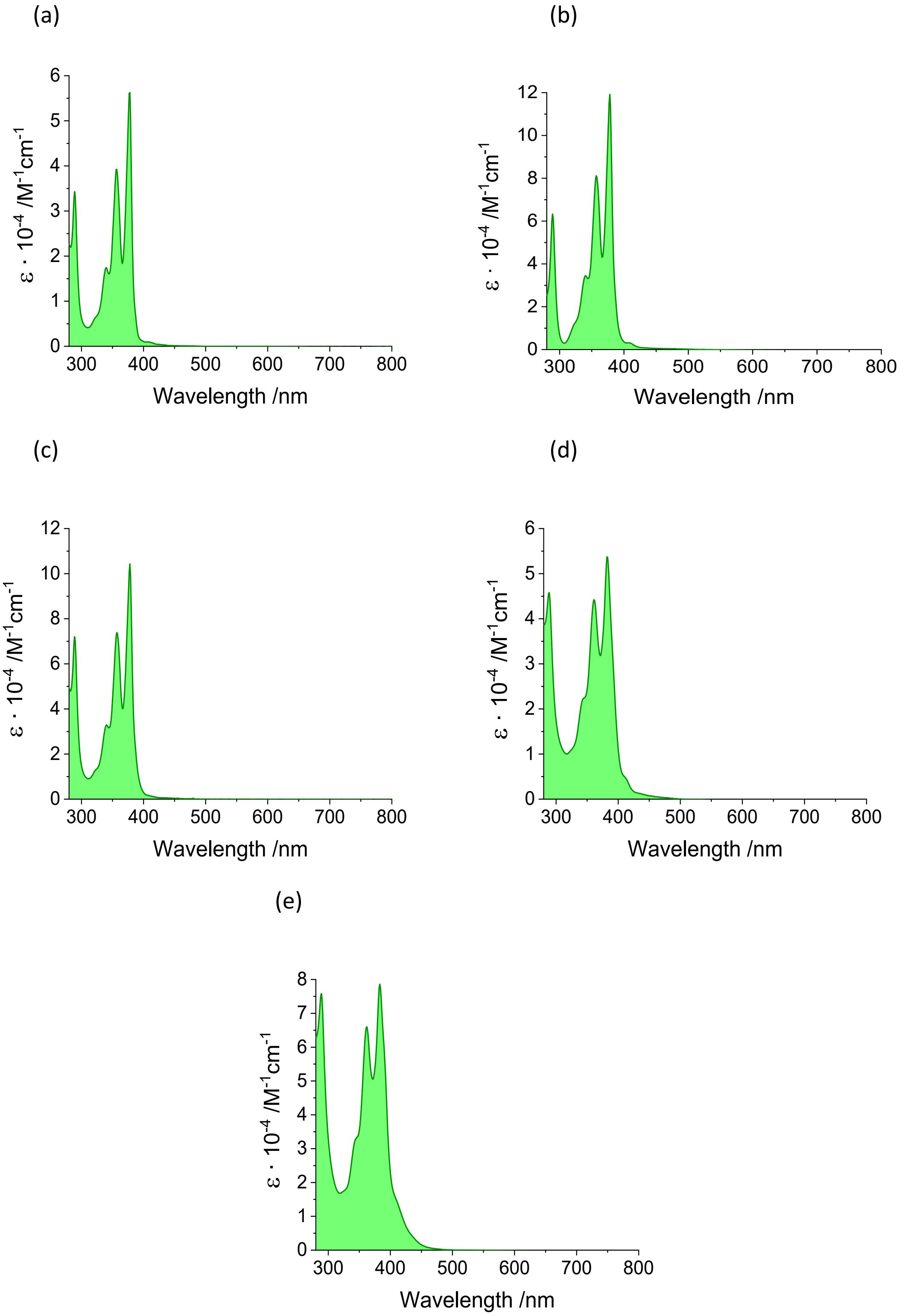

Figure S101. UV-Vis absorption spectra of complexes (a) $PAuPPh_3$ (10^{-5} M), (b) PAudppe (10^{-5} M), (c) PAudppf (10^{-5} M), (d) PAudppbenz (10^{-5} M) and (e) PAuxantphos (10^{-5} M) in DCM.

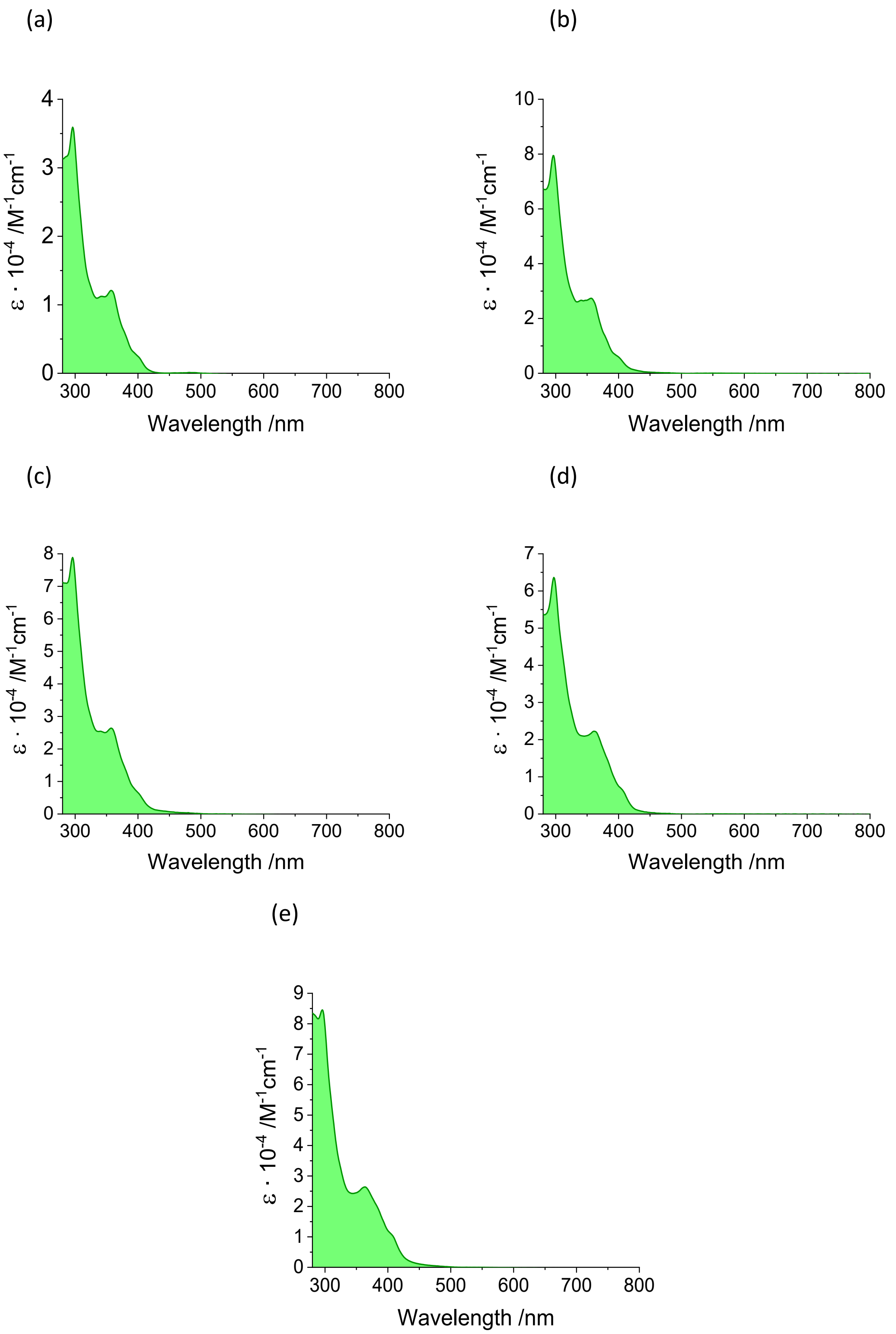

Figure S102. UV-Vis absorption spectra of complexes (a) $CAuPPh_3$ (10^{-5} M), (b) CAudppe (10^{-5} M), (c) CAudppf (10^{-5} M), (d) CAudppbenz (10^{-5} M) and (e) CAuxantphos (10^{-5} M) in DCM.

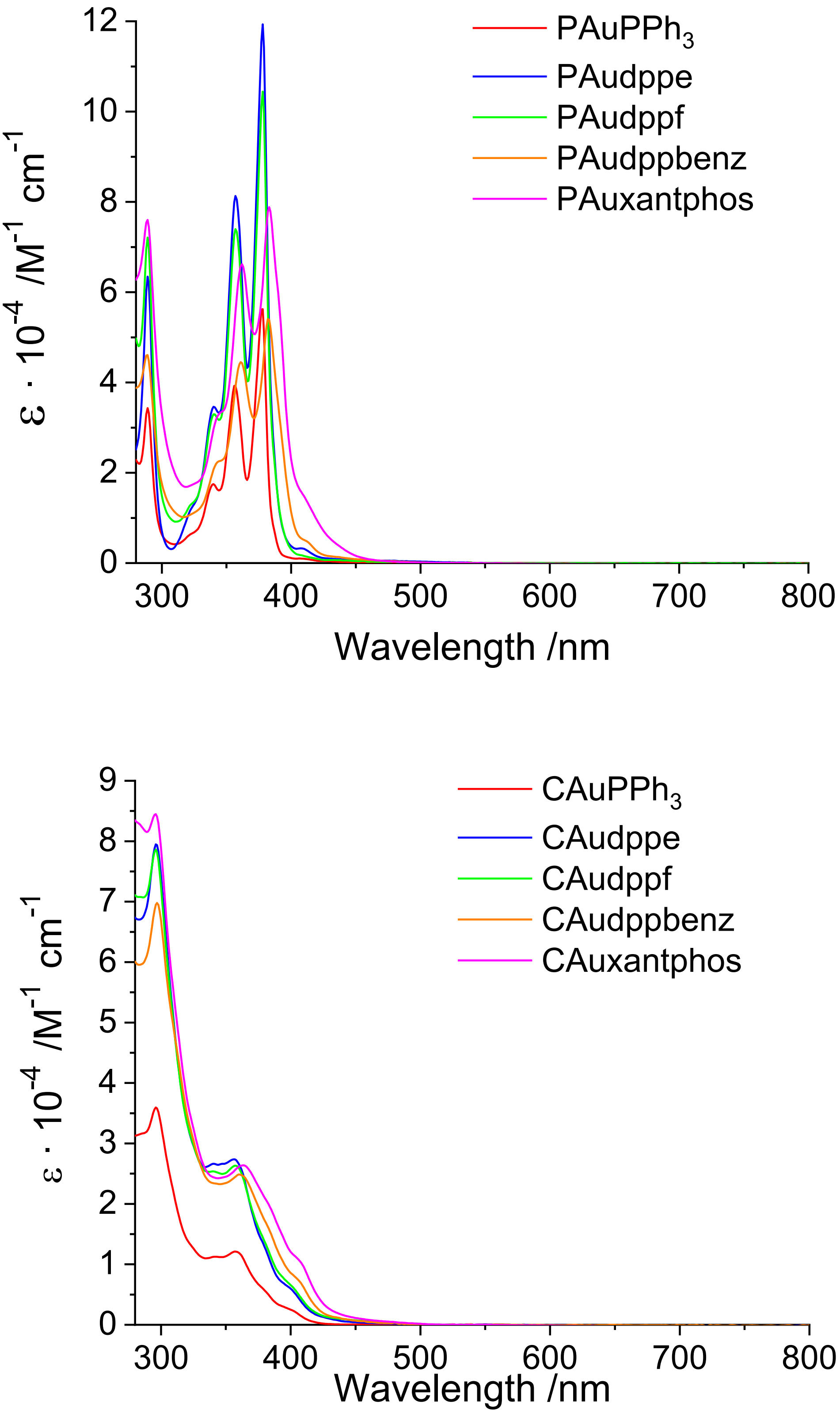

Figure S103. Absorption UV-Vis spectra recorded in 10^{-5} M CH_2Cl_2 solution of pyrene derivatives (above) and corannulene derivatives (below).

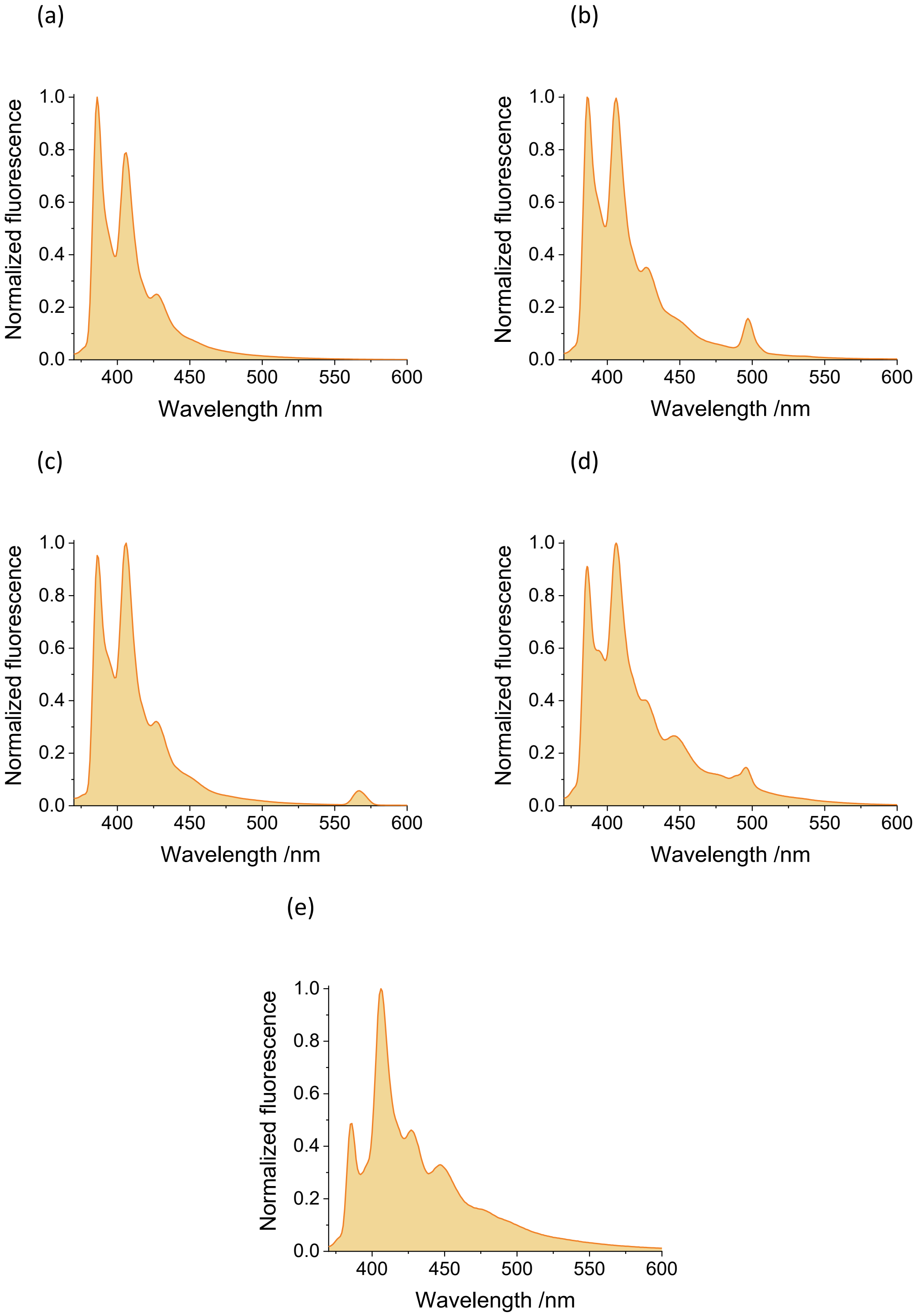

Figure S104. Normalized emission spectra of complexes (a) $PAuPPh_3$ (10^{-5} M) (λ_{ex} = 342 nm), (b) PAudppe (10^{-5} M) (λ_{ex} = 248 nm), (c) PAudppf (10^{-5} M) (λ_{ex} = 283 nm), (d) PAudppbenz (10^{-5} M) (λ_{ex} = 246 nm) and (e) PAuxantphos (10^{-5} M) (λ_{ex} = 341 nm) in DCM.

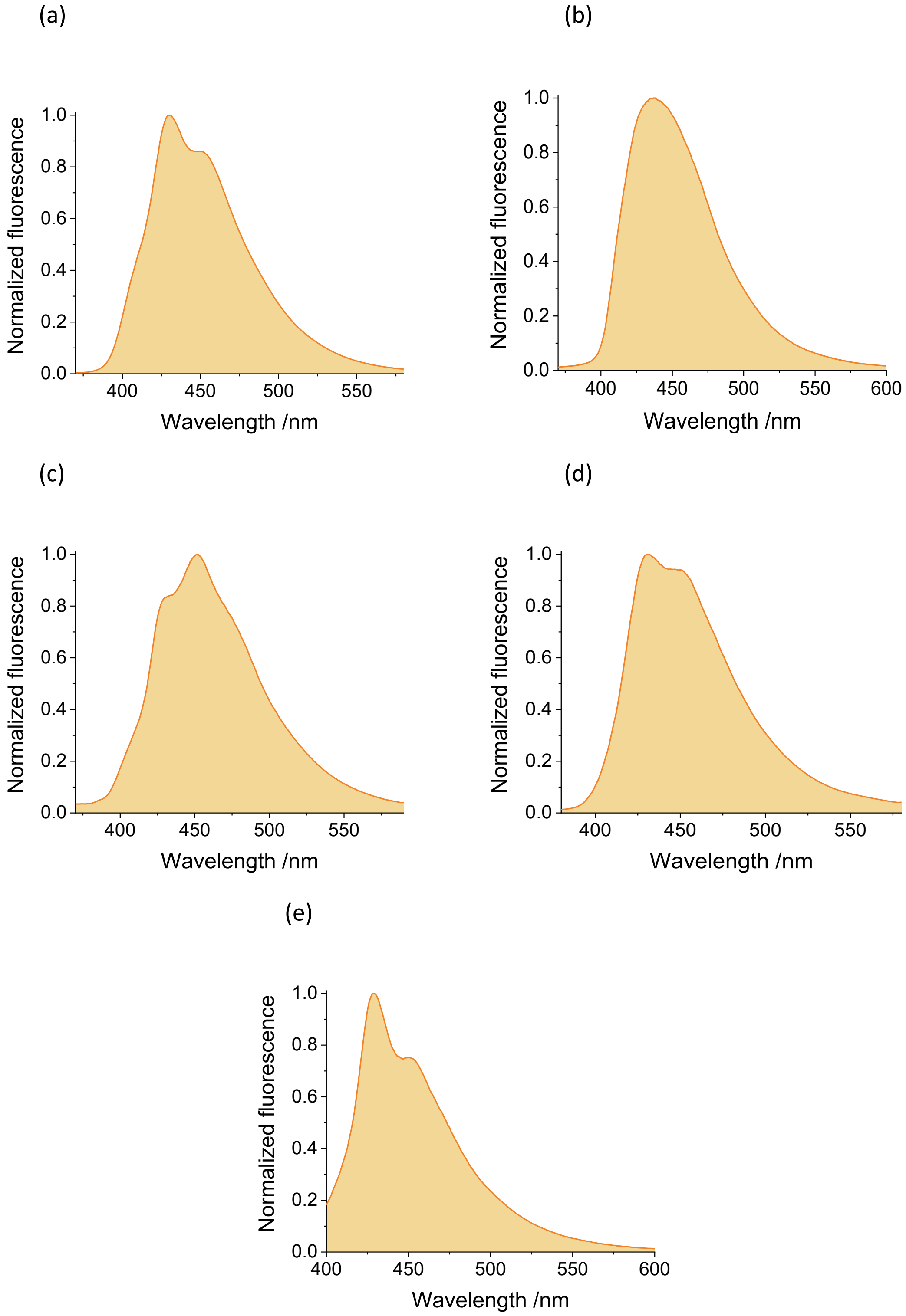

Figure S105. Normalized emission spectra of complexes (a) $CAuPPh_3$ (10^{-5} M) (λ_{ex} = 303 nm), (b) CAudppe (10^{-5} M) (λ_{ex} = 307 nm), (c) CAudppf (10^{-5} M) (λ_{ex} = 301 nm), (d) CAudppbenz (10^{-5} M) (λ_{ex} = 300 nm) and (e) CAuxantphos (10^{-5} M) (λ_{ex} = 369 nm) in DCM.

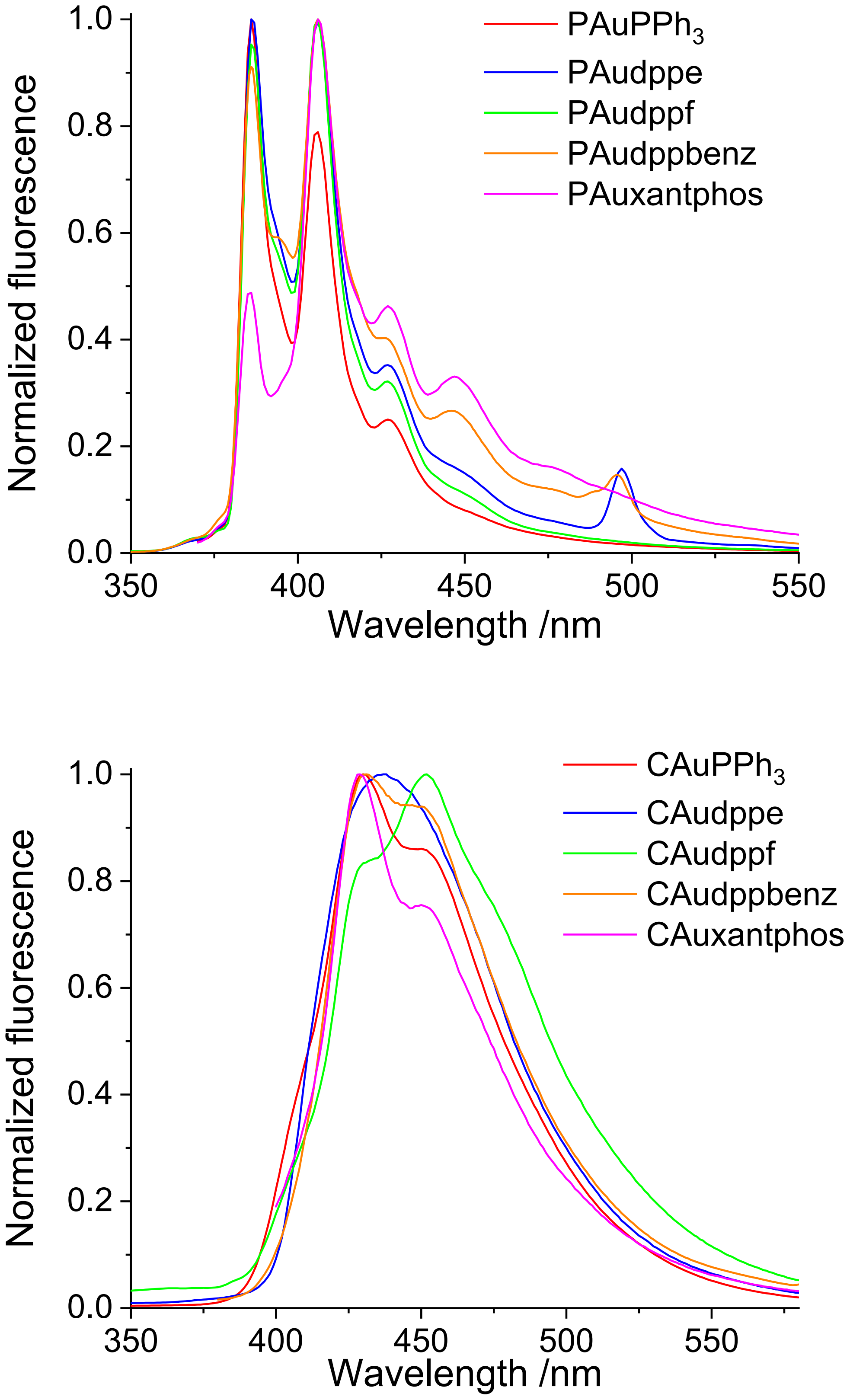

Figure S106. Emission spectra recorded in 10^{-5} M CH_2Cl_2 solution of pyrene derivatives ($\lambda_{ex} \approx 300$ nm) (above) and corannulene derivatives ($\lambda_{ex} \approx 300$ nm) (below).

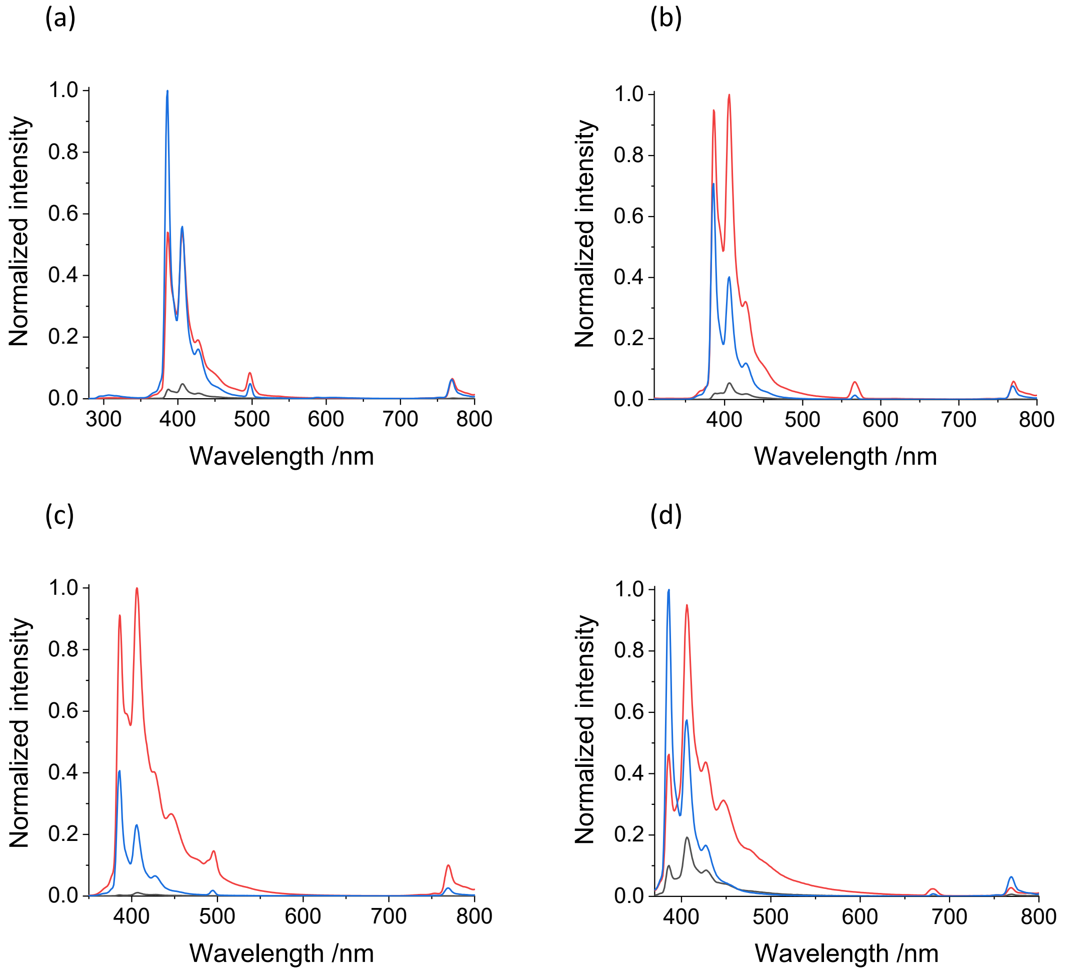

Figure S107. Fluorescence emission spectra in 5 · 10^{-5} M (black), 10^{-5} M (red) and 5 ·10^{-6} M (blue) CH_2Cl_2 solutions of (a) PAudppe (λ_{ex} = 248 nm), (b) PAudppf (λ_{ex} = 283 nm), (d) PAudppbenz (λ_{ex} = 246 nm) and (d) PAuxantphos (λ_{ex} = 341 nm). In some spectra, a harmonic is observed at twice the excitation wavelength.

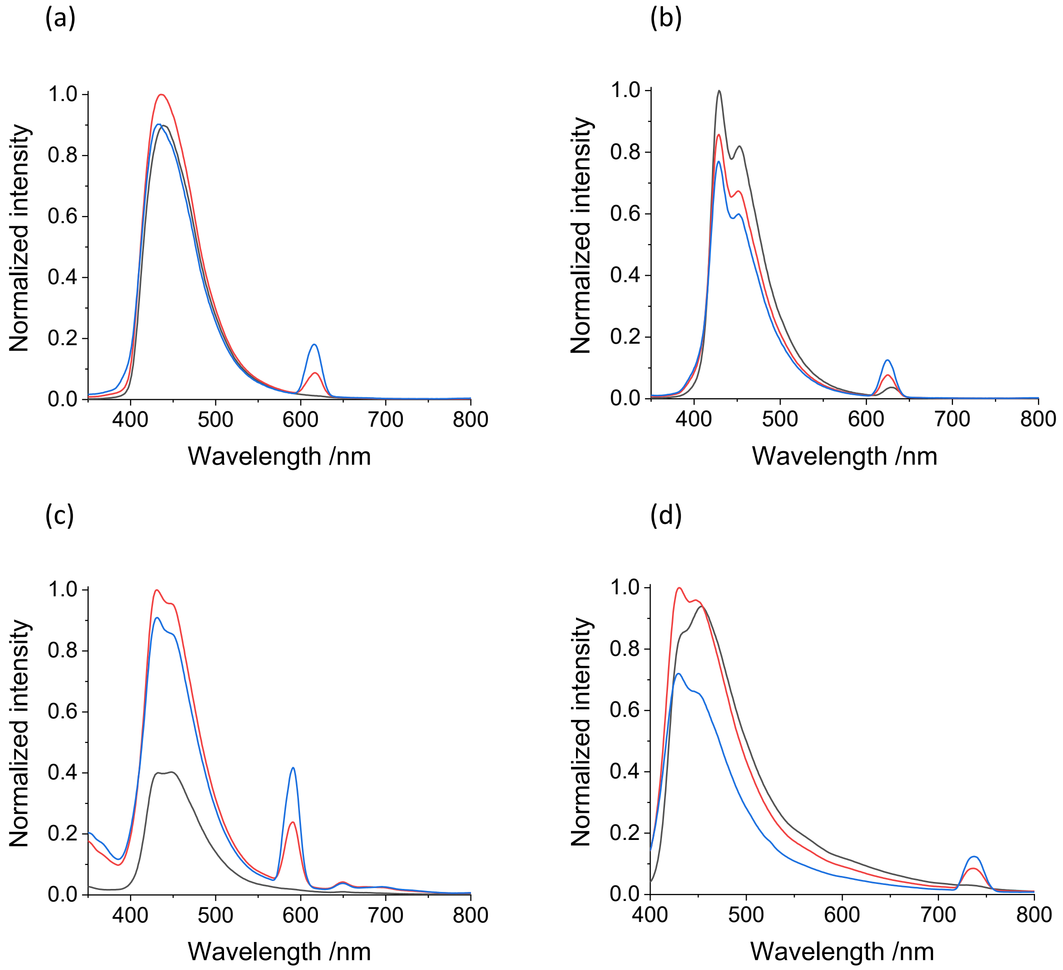

Figure S108. Fluorescence emission spectra in $5 \cdot 10^{-5}$ M (black), 10^{-5} M (red) and $5 \cdot 10^{-6}$ M (blue) CH_2Cl_2 solutions of (a) CAudppe (λ_{ex} = 307 nm), (b) CAudppf (λ_{ex} = 312 nm), (d) CAudppbenz (λ_{ex} = 295 nm) and (d) CAuxantphos (λ_{ex} = 369 nm). In some spectra, a harmonic is observed at twice the excitation wavelength.

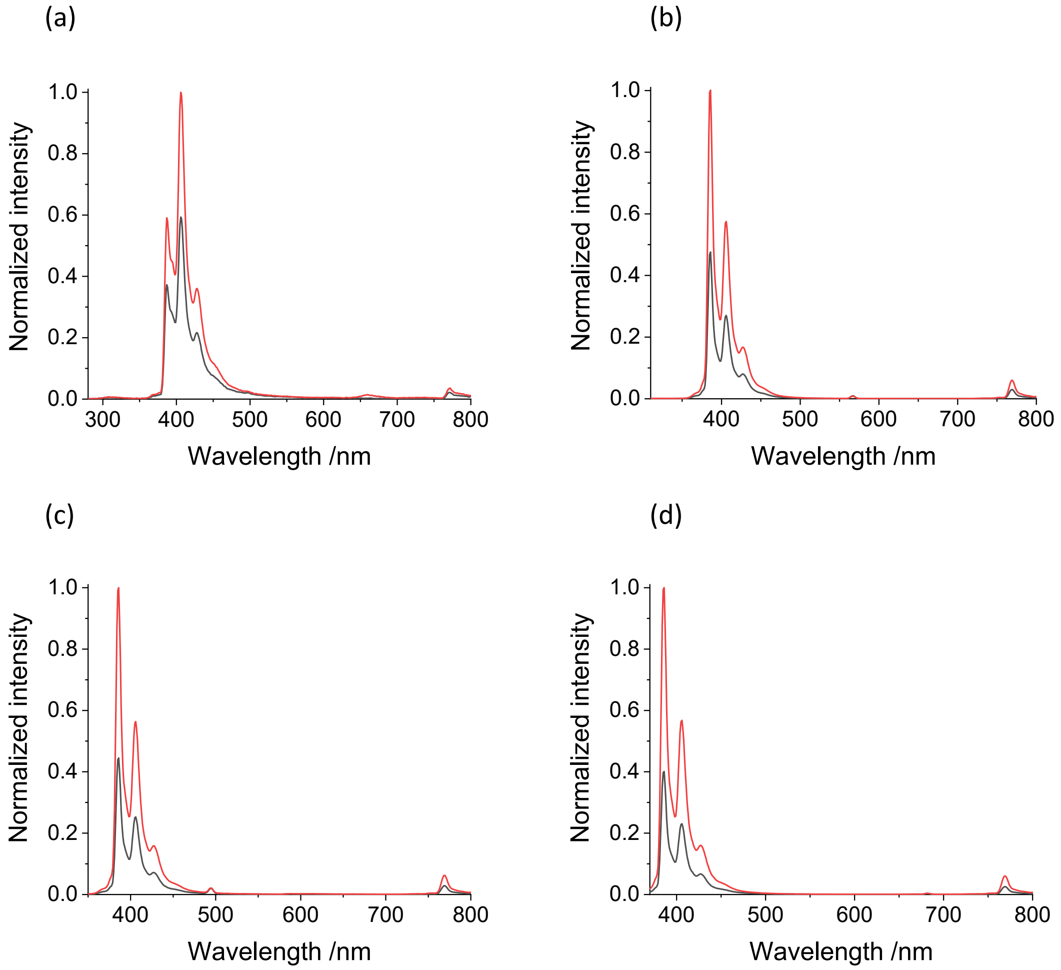

Figure S109. Fluorescence emission spectra in $5 \cdot 10^{-6}$ M CH_2Cl_2 solutions of (a) PAudppe (λ_{ex} = 248 nm), (b) PAudppf (λ_{ex} = 283 nm), (d) PAudppbenz (λ_{ex} = 246 nm) and (d) PAuxantphos (λ_{ex} = 341 nm) in deareated conditions (red) and atmospheric conditions (black). In some spectra, a harmonic is observed at twice the excitation wavelength.

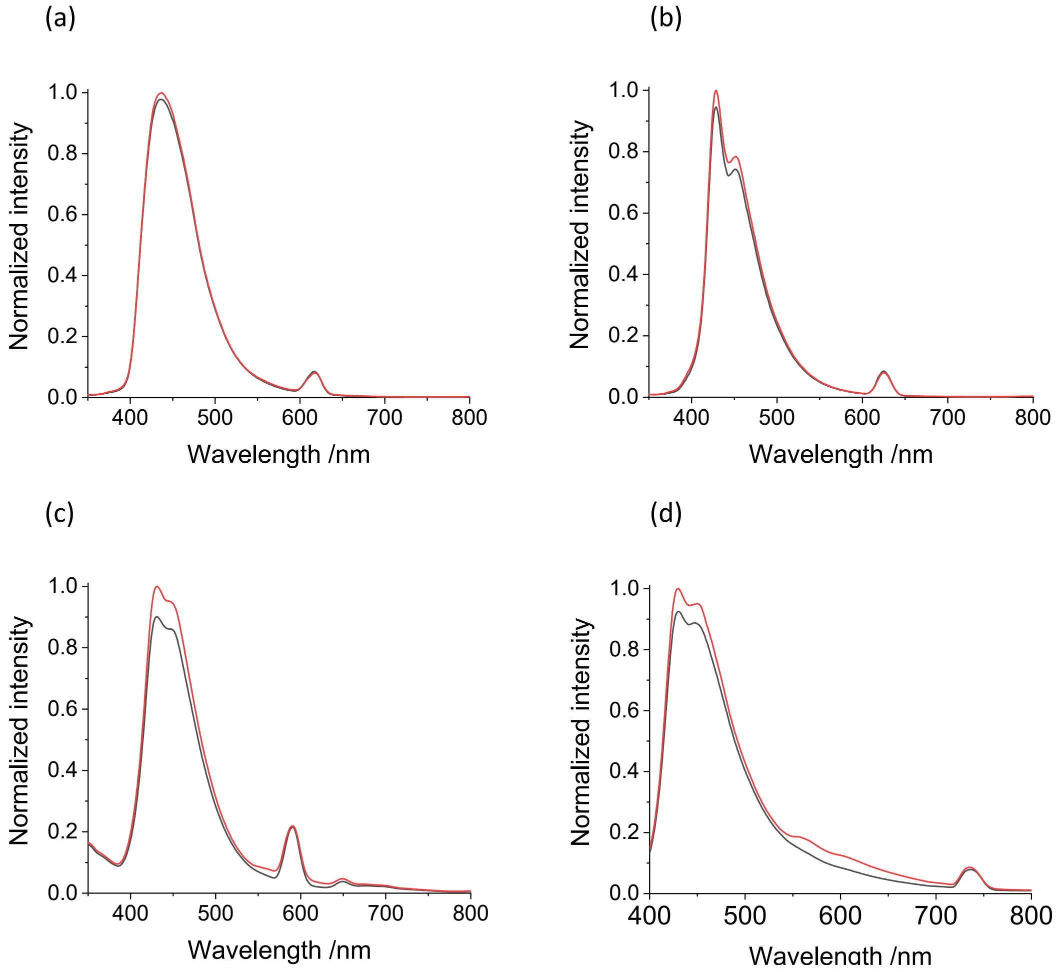

Figure S110. Fluorescence emission spectra in 10^{-5} M CH_2Cl_2 solutions of (a) CAudppe (λ_{ex} = 307 nm), (b) CAudppf (λ_{ex} = 312 nm), (d) CAudppbenz (λ_{ex} = 295 nm) and (d) CAuxantphos (λ_{ex} = 369 nm) in deareated conditions (red) and atmospheric conditions (black). In some spectra, a harmonic is observed at twice the excitation wavelength.

Electrochemical studies

(a)

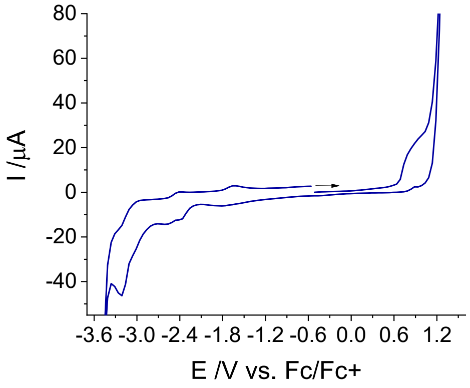

(b)

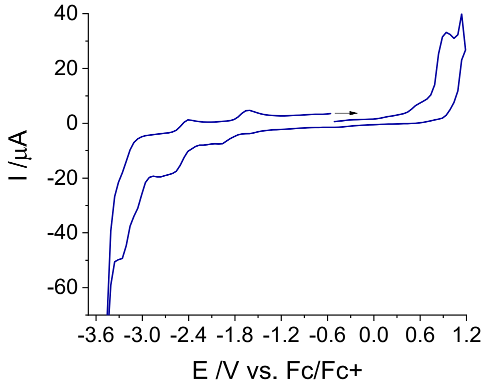

(c)

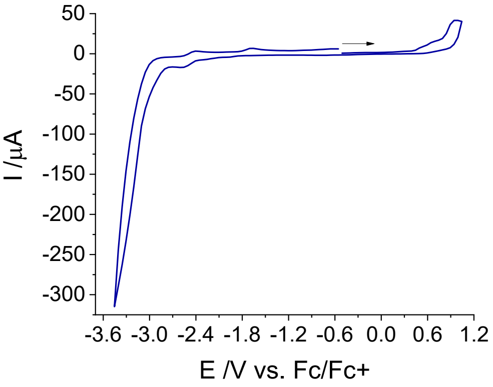

(d)

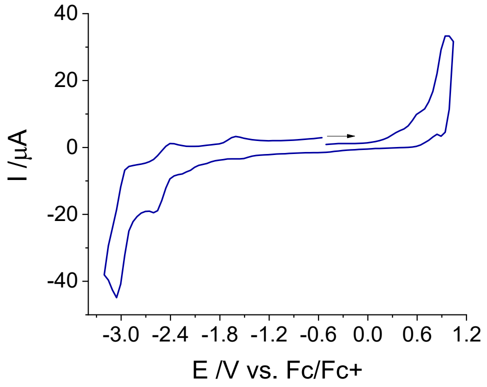

(e)

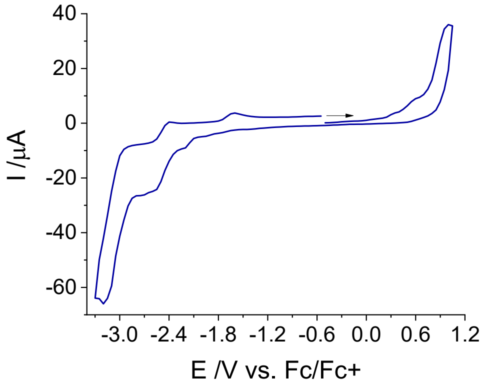

(f)

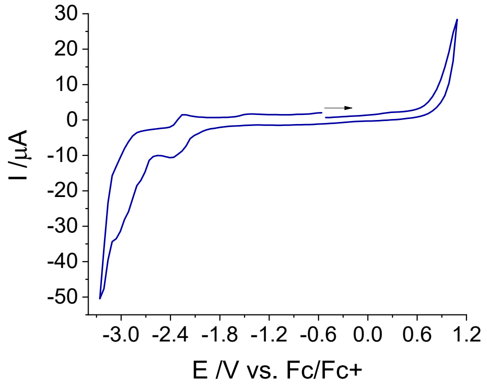

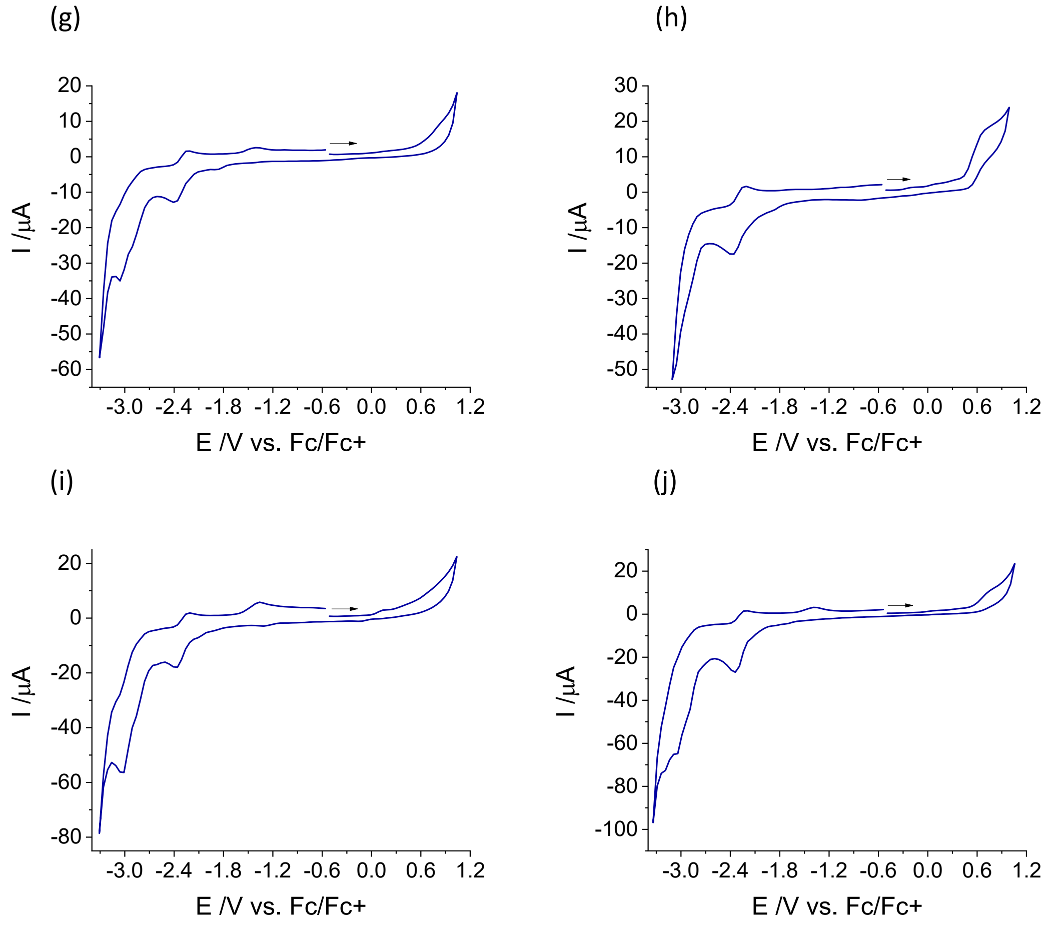

Figure S111. Cyclic voltammograms carried out at room temperature in a one-compartment cell equipped with a glassy carbon electrode, a platinum wire counter electrode and an Ag/AgCl wire as pseudo-reference electrode of complexes (a) PAuPPh$_3$, (b) PAudppe, (c) PAudppf, (d) PAudppbenz, (e) PAuxantphos, (f) CAuPPh$_3$, (g) CAudppe, (h) CAudppf, (i) CAudppbenz and (j) CAuxantphos in deareated DMF at a concentration of 1 mM containing a solution of NBu_4PF_6 (0.1 M). Scan rate of 100 mV · s^{-1}. Potentials are referenced against Fc/Fc$^+$ and plotted using IUPAC convention.

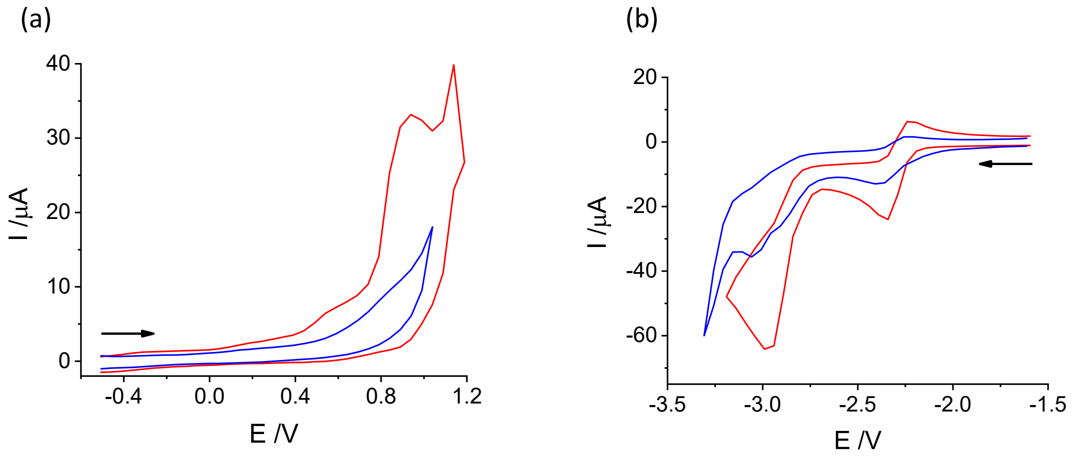

Figure S112. (a) Comparison of oxidative scans of complexes PAudppe (red) and CAudppe (blue). (b) Comparison of reductive scans of corannulene (red) and CAudppe (blue). Measurements performed in DMF at room temperature using tetrabutylammonium hexafluorophosphate (0.1M) as the supporting electrolyte. Potential values are referenced against Fc/Fc$^+$ couple.

(a)

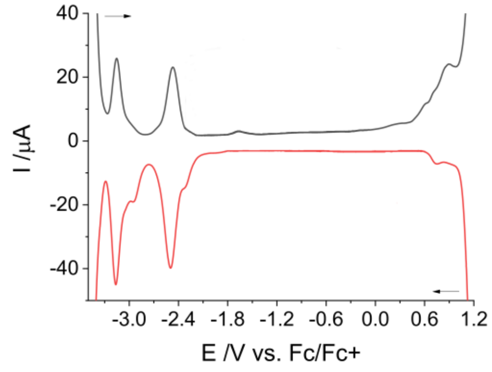
I /µA
E /V vs. Fc/Fc+

(b)

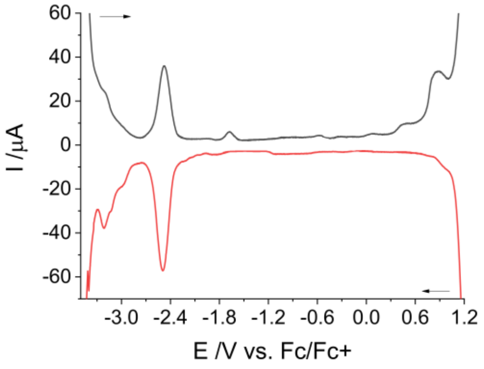
I /µA
E /V vs. Fc/Fc+

(c)

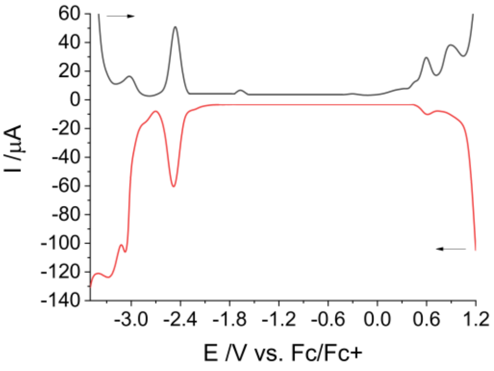
I /µA
E /V vs. Fc/Fc+

(d)

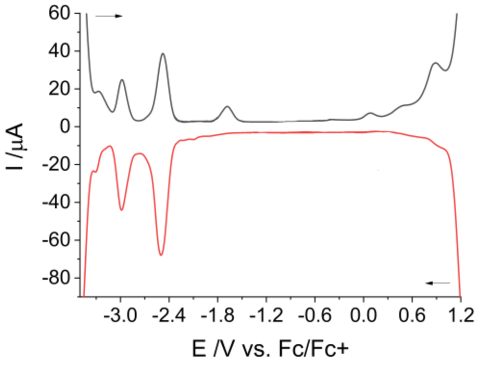
I /µA
E /V vs. Fc/Fc+

(e)

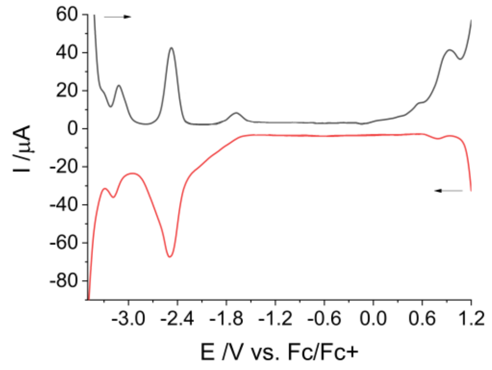
I /µA
E /V vs. Fc/Fc+

(f)

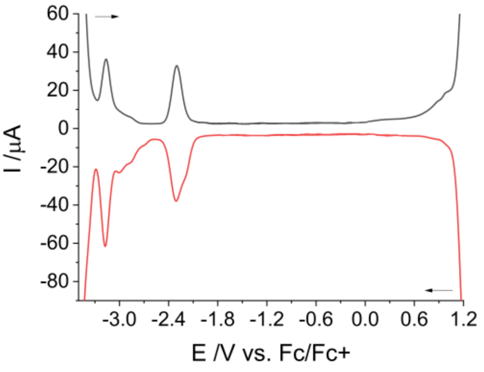
I /µA
E /V vs. Fc/Fc+

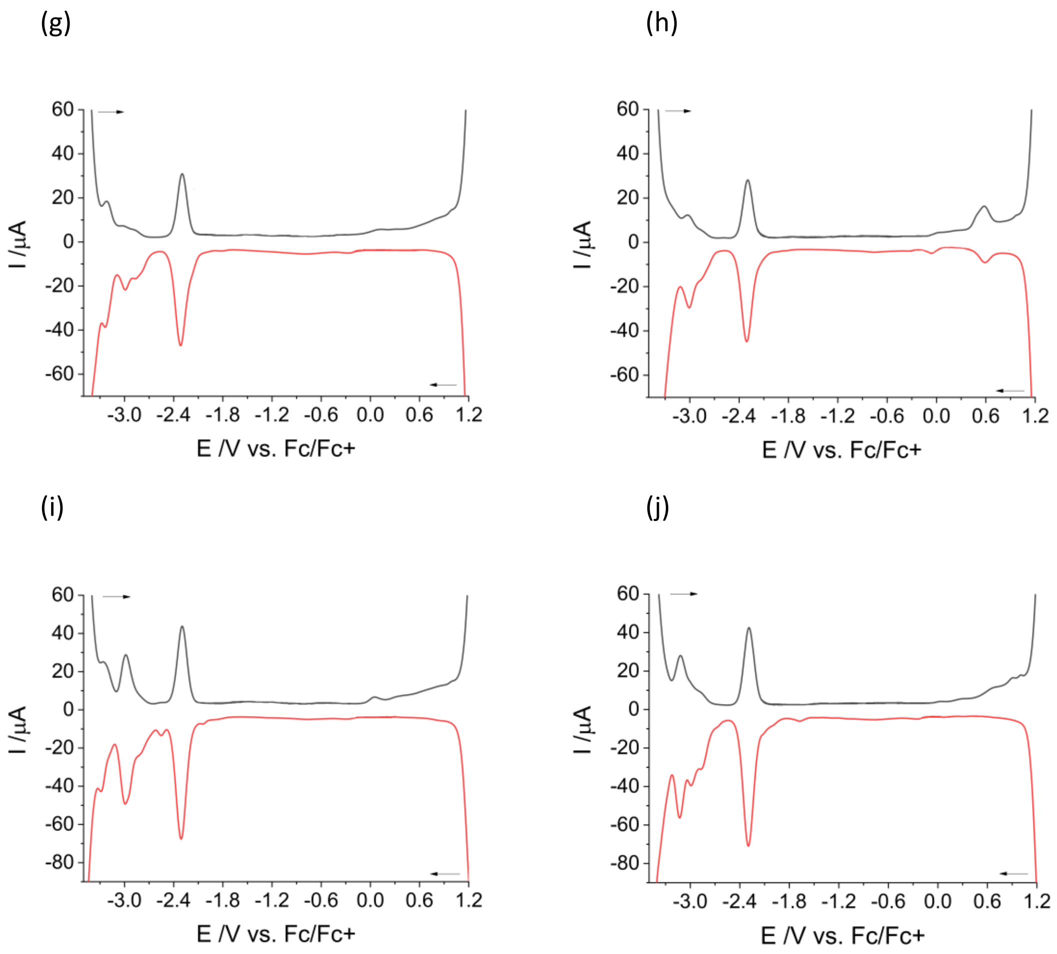

Figure S113. Square-wave voltammograms of compounds (a) $PAuPPh_3$, (b) PAudppe, (c) PAudppf, (d) PAudppbenz, (e) PAuxantphos, (f) $CAuPPh_3$, (g) CAudppe, (h) CAudppf, (i) CAudppbenz and (j) CAuxantphos in deareated DMF at a concentration of 1 mM containing a solution of NBu_4PF_6 (0.1 M). Oxidative scan (black) and reductive scan (red). Potentials are referenced against Fc/Fc^+ and plotted using IUPAC convention.

Table S1. Oxidation and reduction peaks potentials at a concentration of 1 mM containing a solution of NBu_4PF_6 (0.1 M). Potentials are referenced against Fc/Fc^+. All peaks were measured by square-wave voltammetry (SWV).

**Compound**	**E_{ox1} /V**	**E_{ox2} /V**	**E_{red1} /V**	**E_{red2} /V**	**E_{red3} /V**	**E_{red4} /V**
**$PAuPPh_3$**	0.89	-	-2.50	-3.16	-	-
**PAudppe**	0.90	-	-2.49	-3.21	-	-
**PAudppf**	0.60	0.89	-2.48	-3.08	-3.27	-
**PAudppbenz**	0.90	-	-2.50	-2.99	-	-
**PAuxantphos**	0.94	-	-2.50	-3.19	-	-
**$CAuPPh_3$**	-	-	-2.31	-3.17	-	-
**CAudppe**	-	-	-2.32	-2.87	-2.99	-3.24
**CAudppf**	0.58	-	-2.31	-3.01	-	-
**CAudppbenz**	-	-	-2.31	-2.99	-3.29	-
**CAuxantphos**	-	-	-2.30	-2.87	-2.99	-3.13

Reductive scans revealed reduction peaks corresponding to the PAH acetylide ligands. For comparison, the voltammogram of pristine corannulene was recorded under identical

conditions (**¡Error! No se encuentra el origen de la referencia.**b). The reductive scans of corannulene displayed two redox events before solvent reduction occurred. The first event was a reversible one-electron reduction, at -2.29 V (*vs*. Fc/Fc^+), while the second was an irreversible one-electron reduction, at -2.88 V (*vs*. Fc/Fc^+).[¡Error! No se encuentra el origen de la referencia.] In the complexes, the potential of the first reversible reduction remained unaffected by coordination. However, the second reduction exhibited an average cathodic shift of 23 mV relative to pristine corannulene, suggesting an electronic influence from the metal center.

X-Ray crystallography structures

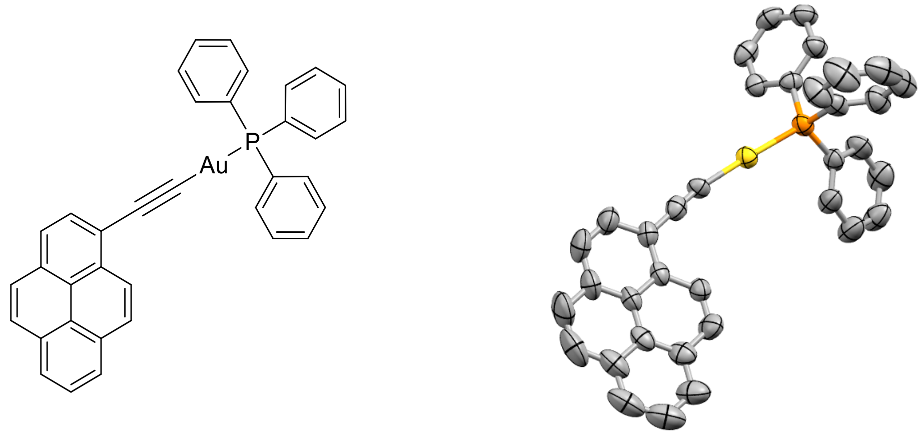

Figure S114. X-Ray diffraction structure of compound PAuPPh$_3$ (50% probability level). Hydrogen atoms have been removed for clarity.

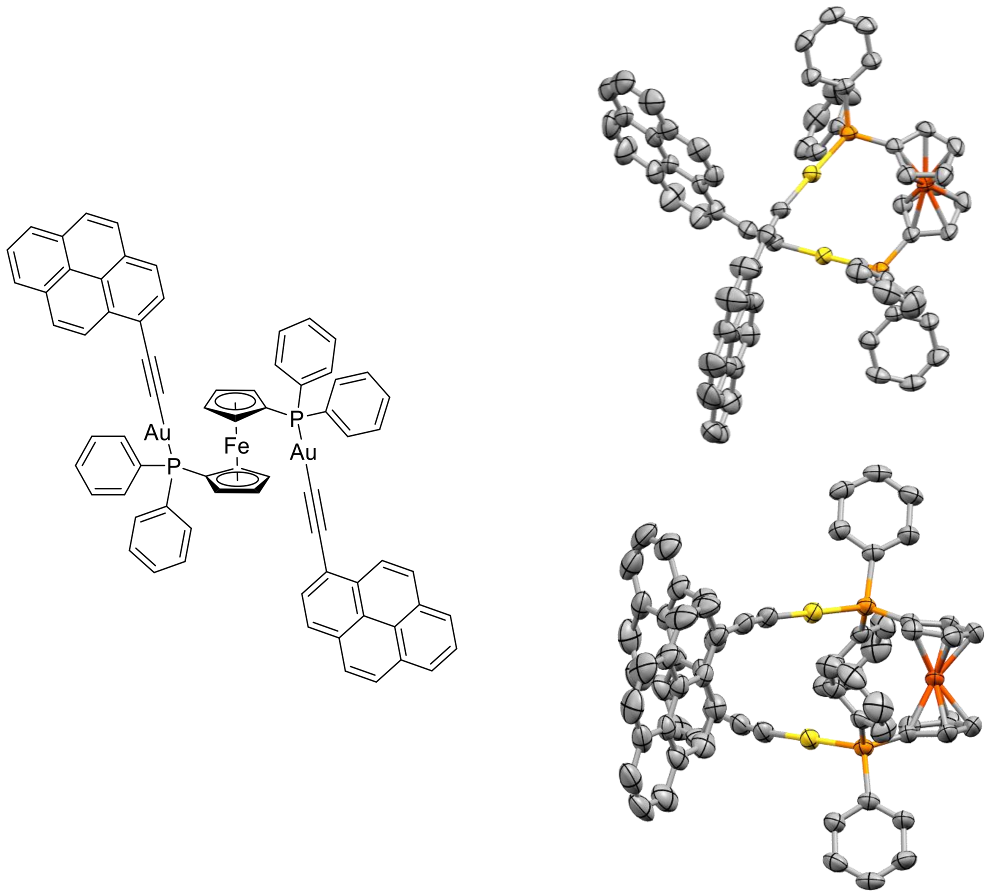

Figure S115. X-Ray diffraction structure of compound PAudppf (50% probability level). Hydrogen atoms have been removed for clarity.

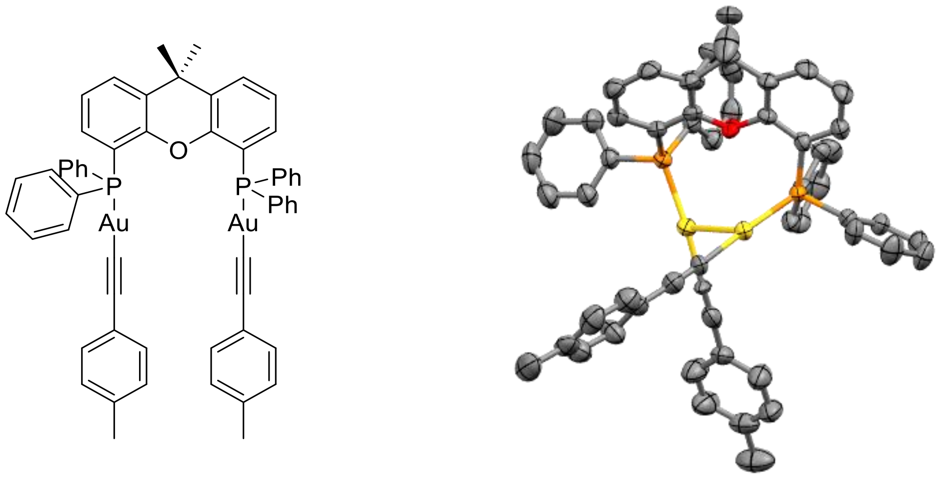

Figure S116. X-Ray diffraction structure of compound *p*-tolylAuxantphos (50% probability level). Hydrogen atoms have been removed for clarity.

Table S2. Crystallographic data for compounds $PAuPPh_3$, PAudppf and *p*-tolylAuxantphos.

**Compound**	**$PAuPPh_3$**	**PAudppf**	***p*-tolylAuxantphos**
CCDC	2488608	2488609	2488610
Empirical formula	$C_{36}H_{24}PAu$	$C_{70}H_{46}P_2FeAu_2$	$C_{57}H_{46}OP_2Au_2$
Formula weight	684.49	1398.79	1202.81
Temperature/K	298	298	298
Crystal system	monoclinic	monoclinic	monoclinic
Space group	C2/c	C2/c	$P2_1/n$
a/Å	20.7264(5)	21.3851(10)	14.1269(4)
b/Å	11.9923(2)	11.6201(4)	19.3899(5)
c/Å	23.9927(5)	22.9844(8)	17.0833(5)
α/°	90	90	90
β/°	111.907(3)	110.797(5)	95.921(3)
γ/°	90	90	90
Volume/$Å^3$	5532.9(2)	5339.4(4)	4654.5(2)
Z	8	4	4
$\rho_{calc}g/cm^3$	1.643	1.740	1.716
μ/mm^{-1}	5.399	5.854	6.406
F(000)	2672.0	2720.0	2336.0
Crystal size/mm^3	0.435 × 0.38 × 0.292	0.295 × 0.227 × 0.132	0.259 × 0.111 × 0.084
Radiation	Mo Kα (λ = 0.71073)	Mo Kα (λ = 0.71073)	Mo Kα (λ = 0.71073)
2Θ range for data collection/°	5.934 to 59.096	6.026 to 59.394	4.472 to 59.344
Index ranges	-28 ≤ h ≤ 28, -14 ≤ k ≤ 16, -30 ≤ l ≤ 28	-26 ≤ h ≤ 26, -15 ≤ k ≤ 16, -21 ≤ l ≤ 30	-19 ≤ h ≤ 19, -24 ≤ k ≤ 25, -22 ≤ l ≤ 21
Reflections collected	24301	14362	103327
Independent reflections	6833 [R_{int} = 0.0358, R_{sigma} = 0.0338]	6362 [R_{int} = 0.0244, R_{sigma} = 0.0418]	12223 [R_{int} = 0.0779, R_{sigma} = 0.0557]
Data/restraints/parameters	6833/0/343	6362/0/339	12223/0/563
Goodness-of-fit on F^2	1.058	1.031	1.059
Final R indexes [I>=2σ (I)]	R_1 = 0.0333, wR_2 = 0.0710	R_1 = 0.0312, wR_2 = 0.0682	R_1 = 0.0386, wR_2 = 0.0722
Final R indexes [all data]	R_1 = 0.0503, wR_2 = 0.0784	R_1 = 0.0536, wR_2 = 0.0742	R_1 = 0.0898, wR_2 = 0.0903
Largest diff. peak/hole / e $Å^{-3}$	0.75/-0.55	0.85/-0.89	1.14/-1.19

^{1}H NMR Titration Experiments

To estimate the association constants (K_a) of the different corannulene complexes prepared with fullerenes C_{60} and C_{70}, the following method was employed: a solution of each compound (5×10^{-5}M) in deuterated toluene (toluene-d_8) was prepared, and a known volume (0.5 mL) was transferred to an NMR tube covered with a septum. The titration was carried out by adding known portions of a stock solution of the fullerene (5×10^{-4}M) in tol-d_8 to cover a wide range of equivalents. A ^{1}H-NMR experiment was conducted at room temperature (298 K) after each addition. Once all data had been obtained, the changes in chemical shifts ($\Delta\delta$) of selected protons were plotted as a function of the molar fraction of the guest, and the resulting curve was fitted by a nonlinear method using the global analysis approach according to the following equations assuming the different equilibria.[5]

A 1:1 equilibrium:

$$K_a = \frac{[\mathrm{HG}]}{[\mathrm{H}][\mathrm{G}]} \qquad \text{eq. 1}$$

The change in the chemical shift upon NMR titration is expressed as Equation 2.

$$\Delta\delta = \Delta\delta_{max}\left(\frac{[\mathrm{HG}]}{[\mathrm{H}]_0}\right) \qquad \text{eq. 2}$$

Where [HG] is the concentration of the supramolecular adduct, and is calculated using Equation 3:

$$[\mathrm{HG}] = \frac{1}{2}\left([\mathrm{G}]_0 + [\mathrm{H}]_0 + \frac{1}{K_a}\right) - \sqrt{\left([\mathrm{G}]_0 + [\mathrm{H}]_0 + \frac{1}{K_a}\right)^2 + 4[\mathrm{G}]_0[\mathrm{H}]_0} \qquad \text{eq. 3}$$

Where:

$[\mathrm{G}]_0$ is the total concentration of the guest (C_{60} or C_{70}).

$[\mathrm{H}]_0$ is the total concentration of the host ($CAuPPh_3$, CAudppe, CAudppf, CAudppbenz or CAuxantphos).

$\Delta\delta_{max}$ is $\Delta\delta$ at maximum complexation (100% supramolecular complex formation).

K_a is the estimated association constant for 1:1 equilibrium.

A 2:1 equilibrium:

$$K_1 = \frac{[\mathrm{HG}]}{[\mathrm{H}][\mathrm{G}]} \qquad \text{eq. 4}$$

$$K_2 = \frac{[\mathrm{H_2G}]}{[\mathrm{HG}][\mathrm{G}]} \qquad \text{eq. 5}$$

$$\beta = K_1K_2 = \frac{[\mathrm{H_2G}]}{[\mathrm{H}]^2[\mathrm{G}]} \qquad \text{eq. 6}$$

The change in the chemical shift upon NMR titration is expressed as Equation 7.

$$\Delta\delta = \frac{\delta_{\Delta\mathrm{HG}}K_1[\mathrm{G}]_0[\mathrm{H}] + 2\delta_{\Delta\mathrm{HG}_2}K_1K_2[\mathrm{G}]_0[\mathrm{H}]^2}{[\mathrm{H}]_0(1 + K_1[\mathrm{H}] + K_1K_2[\mathrm{H}]^2)} \qquad \text{eq. 7}$$

Where [H] is the concentration of free host, and is calculated using the cubic Equation 8:

$$[\mathrm{H}]^3(\mathrm{A}) + [\mathrm{H}]^2(\mathrm{B}) + [\mathrm{H}](\mathrm{C}) - [\mathrm{G}]_0 = 0 \qquad \text{eq. 8}$$

With:

$$\mathrm{A} = (K_1K_2)$$

$$\mathrm{B} = \{K_1(2K_2[\mathrm{G}]_0 - K_2[\mathrm{H}]_0 + 1)\}$$

$$\mathrm{C} = \{K_1([\mathrm{G}]_0 - [\mathrm{H}]_0) + 1\}$$

Where:

$[G]_0$ is the total concentration of the guest (C_{70}).

$[H]_0$ is the total concentration of the host ($CAuPPh_3$).

$\Delta\delta_{\Delta HG}$ is $\Delta\delta$ at maximum complexation of the first equilibrium [HG].

$\Delta\delta_{\Delta H2G}$ is $\Delta\delta$ at maximum complexation of the second equilibrium $[H_2G]$.

K_1 is the estimated association constant for the first equilibrium.

K_2 is the estimated association constant for the second equilibrium.

In each case, the $\Delta\delta_{max}$ and K_a for a 1:1 equilibrium and $\Delta\delta_{\Delta HG}$, $\Delta\delta_{\Delta H2G}$, K_1 and K_2 for a 2:1 equilibrium were extracted using the non-linear fitting tool provided at the open access web portal supramolecular.org. Links to all the fittings of the data are provided below. The most strongly affected chemical shifts were those belonging to the protons of the corannulene substituent, and the protons H^2 and H^{10}, which are the protons closest to the substituted carbon, were usually selected. They were also selected because they appear in a clean region of the spectrum, and their signals can be reliably followed.

$CAuPPh_3$ *vs.* C_{60}

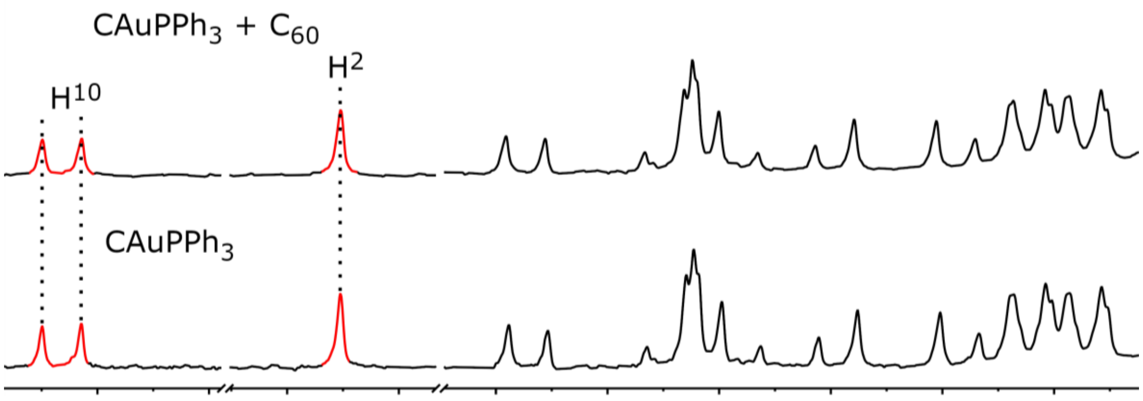

Figure S117. ^{1}H NMR spectra of complex $CAuPPh_3$ (down) and its mixture with excess amount of C_{60} (up) in toluene-d_8 at 298 K. No change in the chemical shifts was observed upon C_{60} addition.

$CAuPPh_3$ *vs.* C_{70}

1:1

http://app.supramolecular.org/bindfit/view/a0d050f5-cfc7-4fed-a96f-67d380fc3d24

2:1 full

http://app.supramolecular.org/bindfit/view/89c0bab7-6f1d-4751-ad43-a32ca3e22219

2:1 additive

http://app.supramolecular.org/bindfit/view/7a469b97-539f-4a55-8895-422dfac2ae71

2:1 non-cooperative

http://app.supramolecular.org/bindfit/view/8a32612f-3420-4205-980d-900482c0a8d0

2:1 statistical

http://app.supramolecular.org/bindfit/view/02c319fc-36f4-45f8-a361-7bde496b04c6

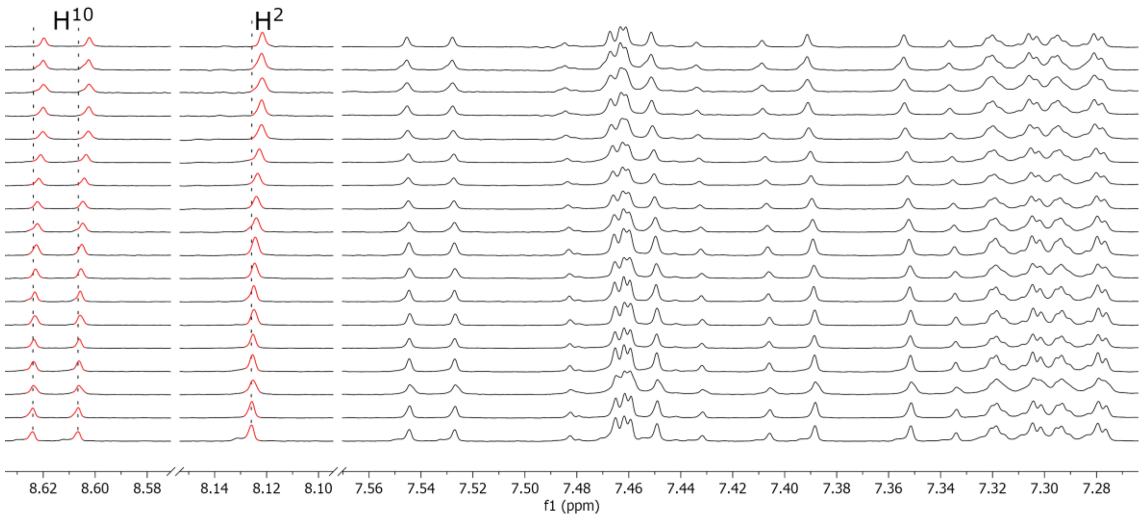

Figure S118. Stacked ^{1}H-NMR spectra for the titration of $CAuPPh_3$ with variable concentrations of C_{70} in toluene-d_8 at 298 K. The most significant chemical shifts of the corannulene unit (H^{10}, H^2) have been labelled.

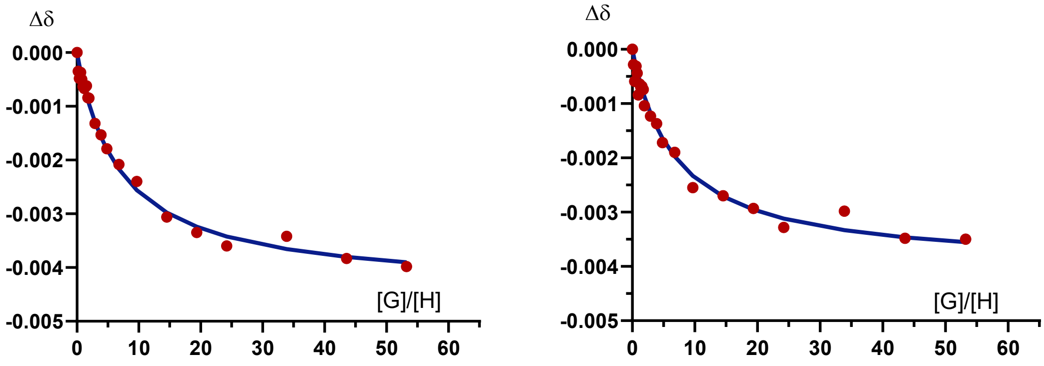

Figure S119. Non-linear regressions (1:1 equilibrium) for selected protons (left plot: H^{10}, right plot: H^2) for the titration of complex $CAuPPh_3$ with C_{70}.

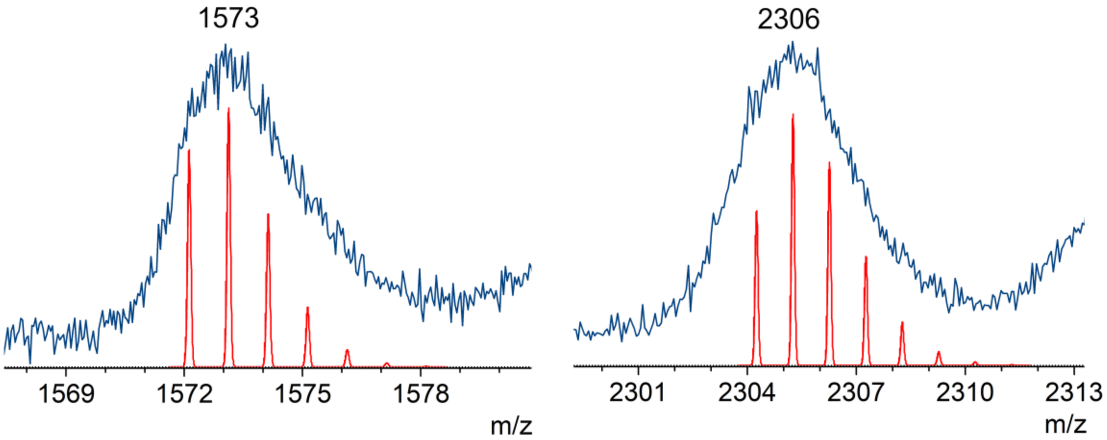

Figure S120. Detection of adducts C_{70}@CAuPPh$_3$ (left) and C_{70}@(CAuPPh$_3$)$_2$ (right) in LRMS (MALDI-TOF, linear detection, positive mode). Calculated (red), measured (blue).

Table S3. Association constants (M^{-1}) and ΔG_a (kJ/mol) values of host CAuPPh$_3$ with C_{70} according to different binding models.

Model	K_1	K_2	ΔG_1	ΔG_2	Cov_{fit} ratio
**1:1**	4.41 ± 0.10 · 10^2	-	-15.09 ± 0.06	-	1.00
**2:1 full**	6.98 ± 0.33 · 10^2	3.20 ± 1.46 · 10^3	-16.23 ± 0.16	-20.0 ± 1.13	1.09
**2:1 additive**	3.05 ± 0.13 · 10^2	2.26 ± 2.81 · 10^2	-14.17 ± 0.11	-13.43 ± 3.08	1.00
**2:1 non-coop**	4.42 ± 0.10 · 10^2	1.10 ± 0.02 · 10^2	-15.10 ± 0.06	-11.66 ± 0.06	1.09
**2:1 statistical**	3.82 ± 0.08 · 10^2	95.4 ± 2.1	-14.73 ± 0.05	-11.29 ± 0.05	1.00

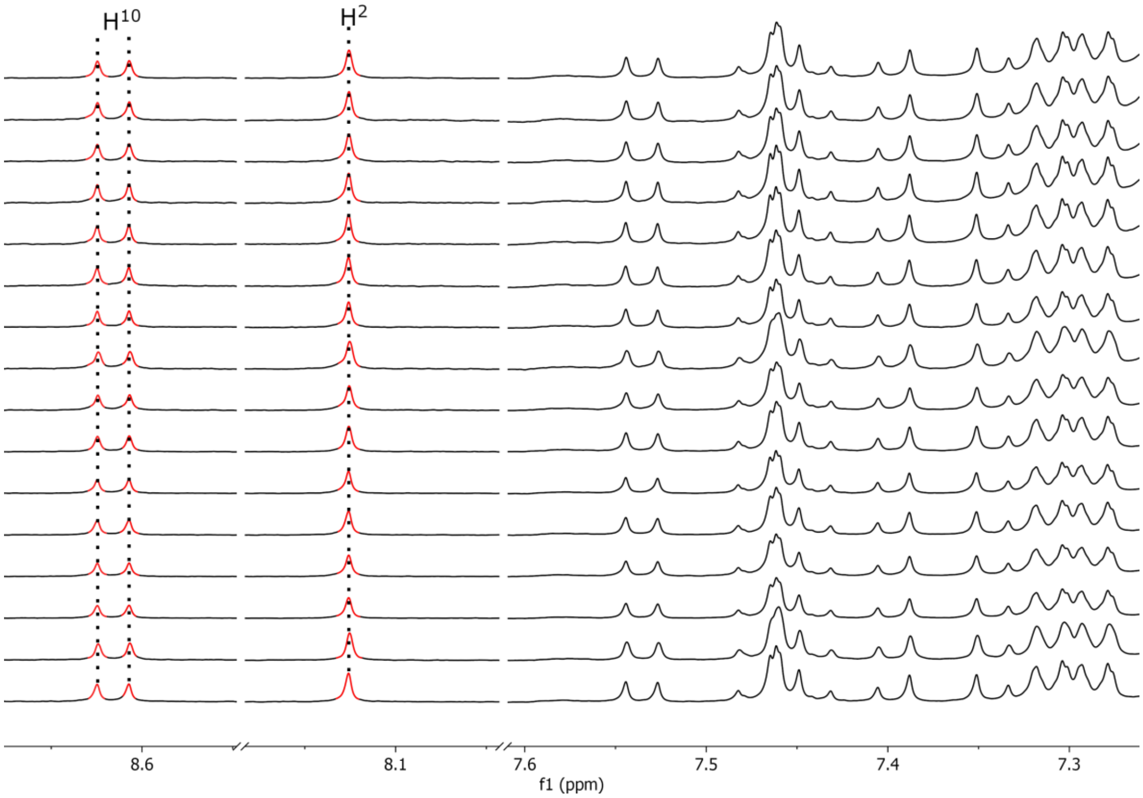

Figure S121. Stacked ^{1}H-NMR spectra for the dilution experiments of $CAuPPh_3$ in toluene-d_8 at 298 K. The most significant signals of the corannulene unit (H^{10}, H^2) have been labelled.

CAudppe *vs.* C_{60}

1:1

http://app.supramolecular.org/bindfit/view/1b1fe1e8-e8ab-4c85-90c9-828255e9571d

CAudppe *vs.* C_{70}

1:1

http://app.supramolecular.org/bindfit/view/fe23b9b2-87d7-4109-af1d-e173e09432cb

Table S4. Association constants (M^{-1}) and and ΔG_a (kJ/mol) of host CAudppe with fullerenes according to a 1:1 model.

Guest	K_a	ΔG_a
C_{60}	$6.85 \pm 0.18 \cdot 10^2$	-16.18 ± 0.07
C_{70}	$7.72 \pm 0.31 \cdot 10^2$	-16.47 ± 0.10

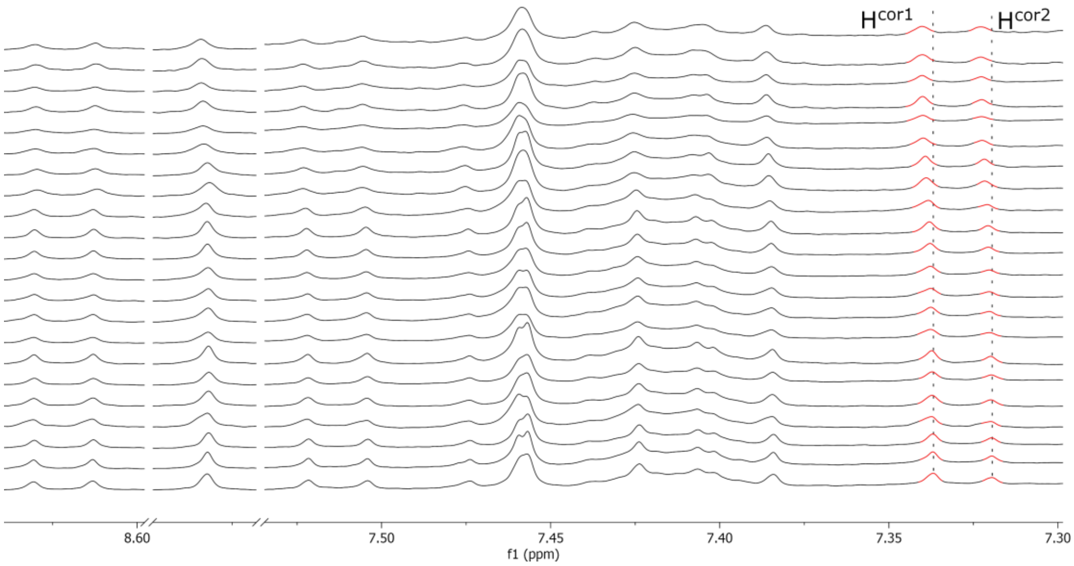

Figure S122. Stacked ^{1}H-NMR spectra for the titration of CAudppe with variable concentrations of C_{60} in toluene-d_8 at 298 K. The most significant chemical shifts of the corannulene unit (H^{cor1}, H^{cor2}) have been labelled.

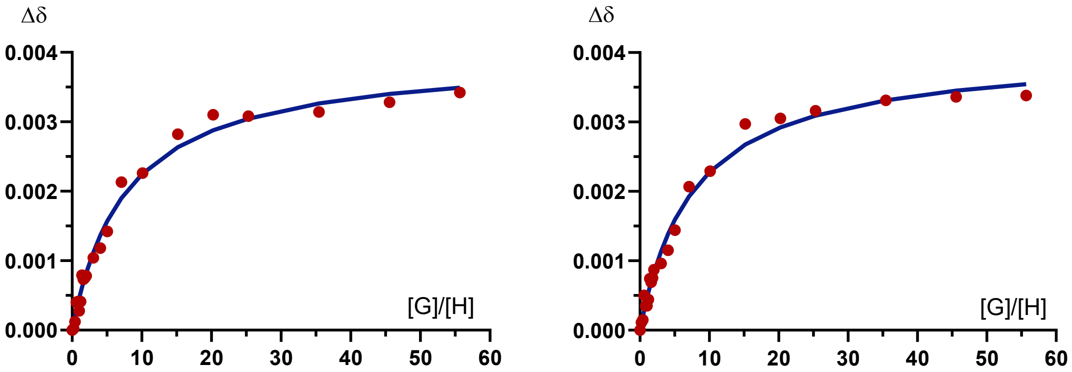

Figure S123. Non-linear regressions for selected protons (left plot: H^{cor1}, right plot: H^{cor2}) for the titration of complex CAudppe with C_{60}.

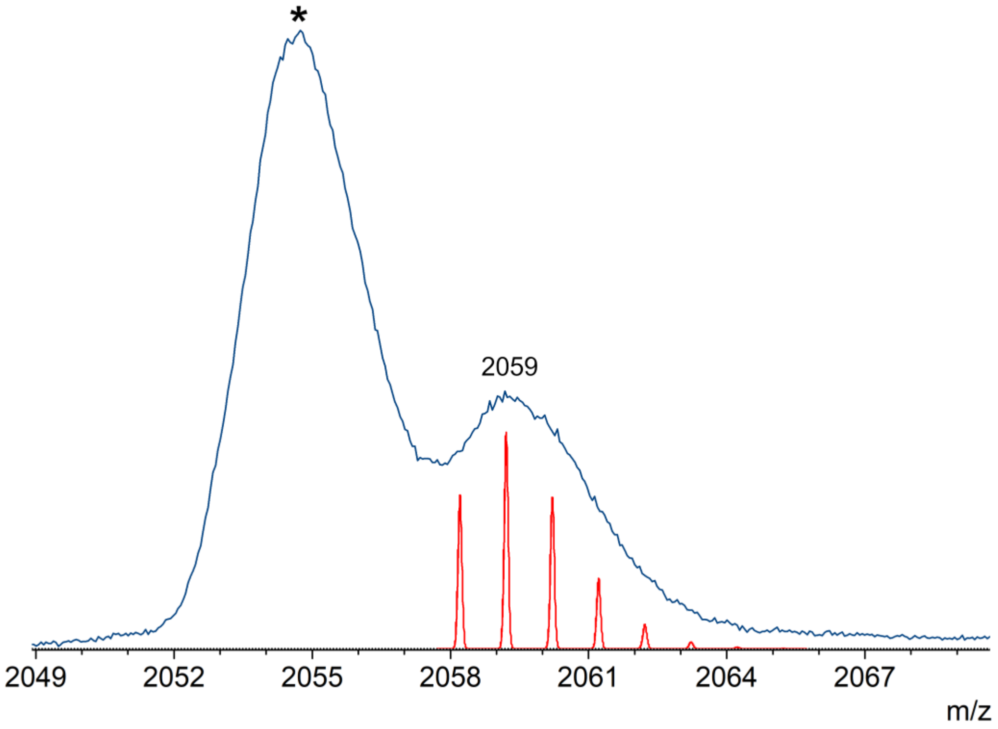

Figure S124. Detection of adduct C_{60}@CAudppe in LRMS (MALDI-TOF, linear detection, positive mode). Calculated (red), measured (blue). *Peaks could not be assigned.

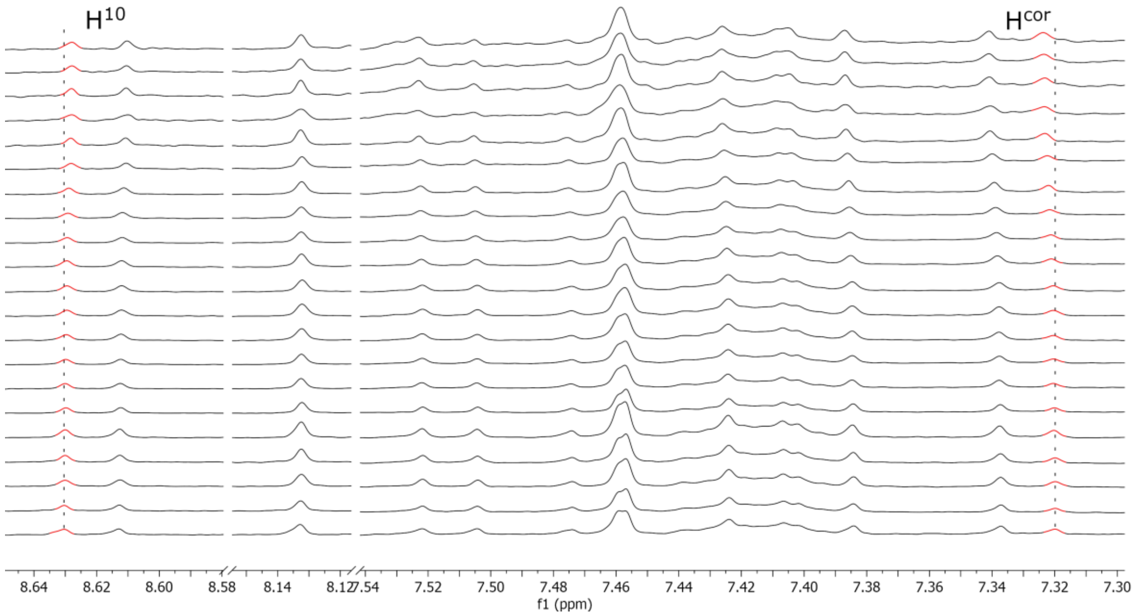

Figure S125. Stacked ^{1}H-NMR spectra for the titration of CAudppe with variable concentrations of C_{70} in toluene-d_8 at 298 K. The most significant chemical shifts of the corannulene unit (H^{10}, H^{cor}) have been labelled.

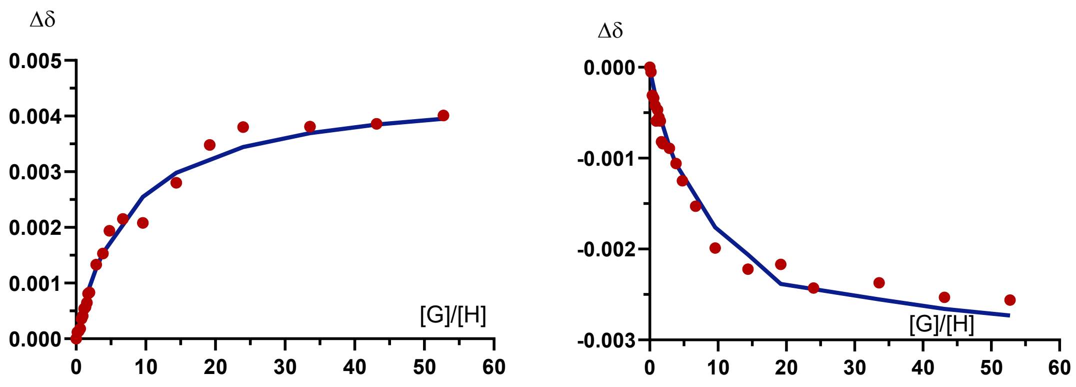

Figure S126. Non-linear regressions for selected protons (left plot: H^{10}, right plot: H^{cor}) for the titration of complex CAudppe with C_{70}.

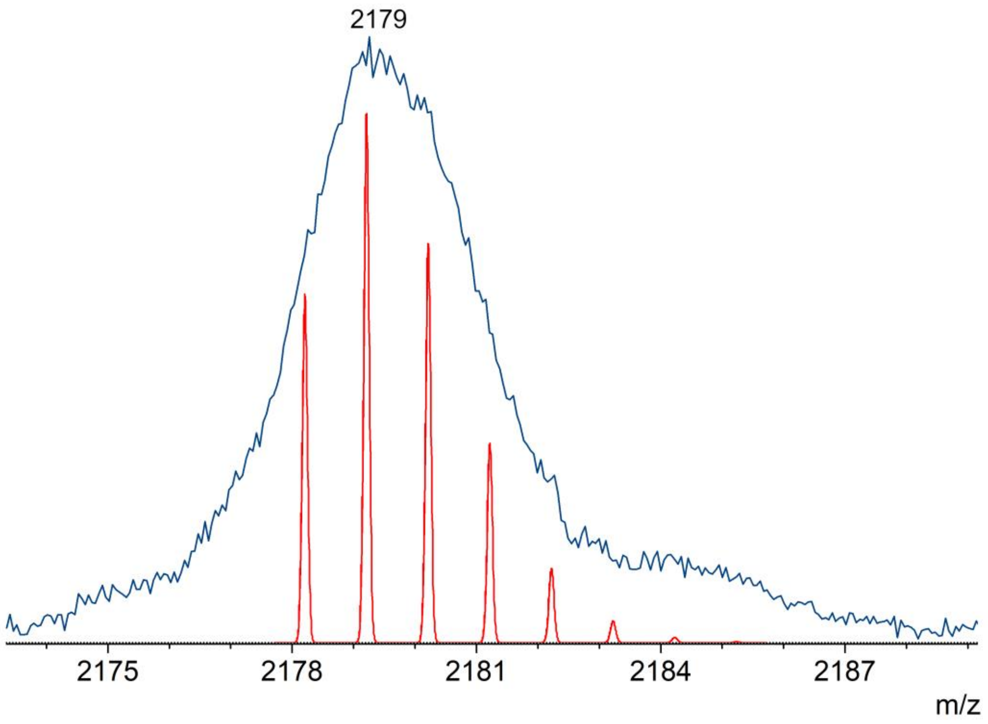

Figure S127. Detection of adduct C_{70}@CAudppe in LRMS (MALDI-TOF, linear detection, positive mode). Calculated (red), measured (blue).

CAudppf *vs.* C_{60}

1:1

http://app.supramolecular.org/bindfit/view/cbfdcc35-efab-4f5c-a5e3-dab887f8bf9b

CAudppf *vs.* C_{70}

1:1

http://app.supramolecular.org/bindfit/view/e335a5d4-9424-4f39-a269-21b3581c7887

Table S5. Association constants (M^{-1}) and and ΔG_a (kJ/mol) of host CAudppf with fullerenes according to a 1:1 model.

Guest	K_a	ΔG_a
C_{60}	$9.64 \pm 0.23 \cdot 10^2$	-17.02 ± 0.06
C_{70}	$9.44 \pm 0.19 \cdot 10^2$	-16.97 ± 0.05

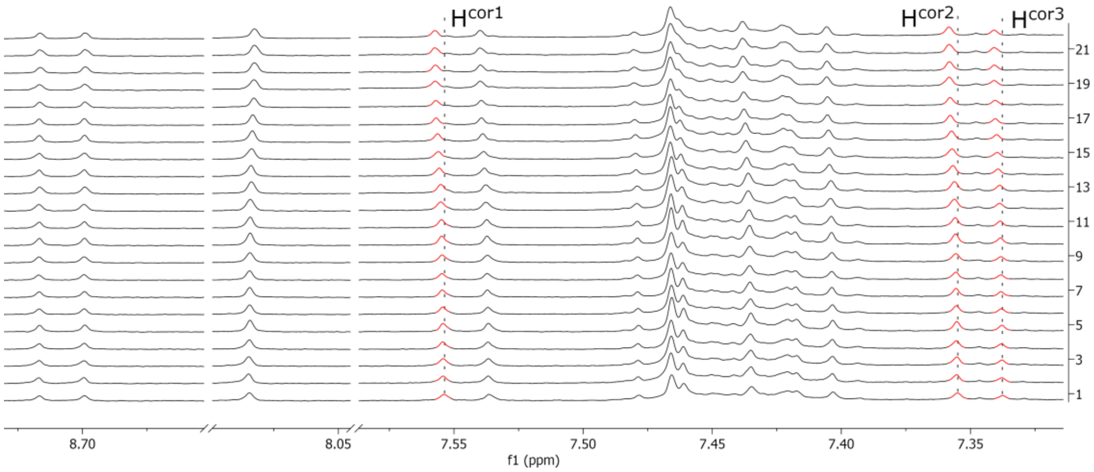

Figure S128. Stacked ^{1}H-NMR spectra for the titration of CAudppf with variable concentrations of C_{60} in toluene-d_8 at 298 K. The most significant chemical shifts of the corannulene unit (H^{cor1}, H^{cor2}) have been labelled.

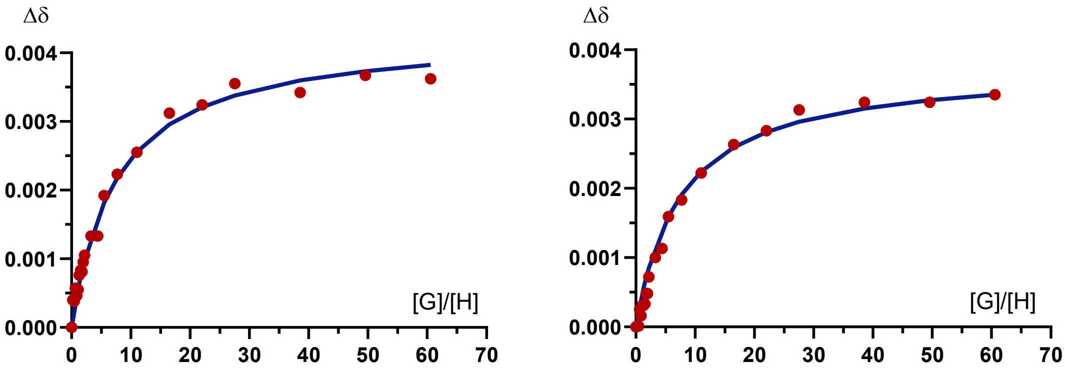

Figure S129. Non-linear regressions for selected protons (left plot: H^{cor1}, right plot: H^{cor2}) for the titration of complex CAudppf with C_{60}.

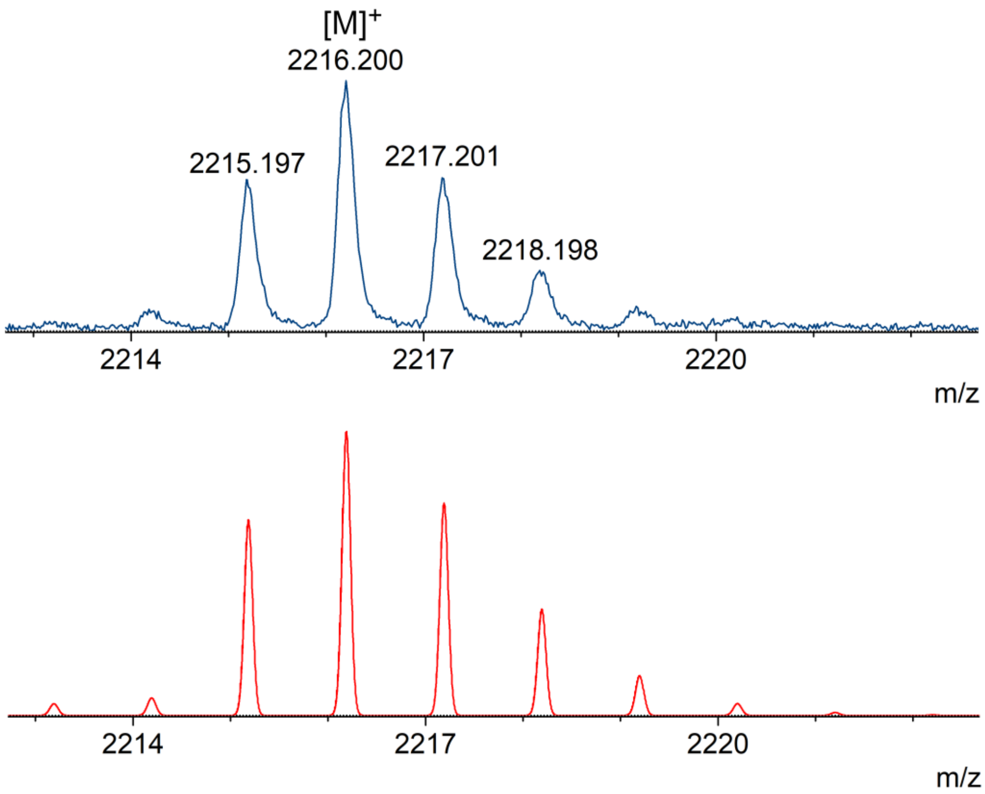

Figure S130. Detection of adduct C_{60}@CAudppf in HRMS (MALDI-TOF, linear detection, positive mode). Calculated (red), measured (blue). HRMS (MALDI-TOF): m/z = 2216.2000 $[M]^+$, calculated 2216.1872 for $C_{138}H_{47}Au_2FeP_2$.

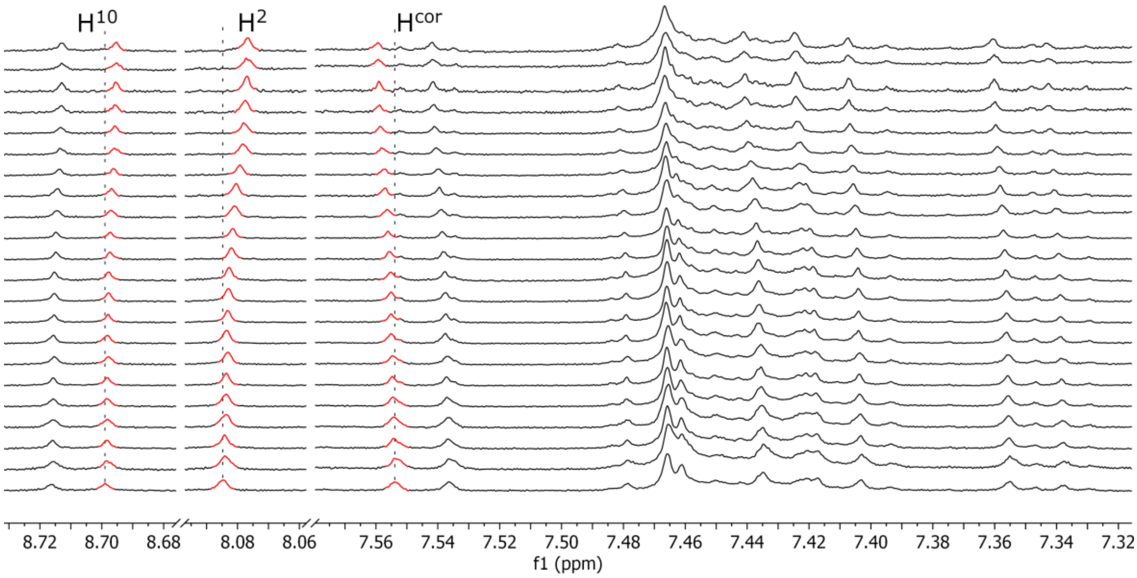

Figure S131. Stacked ^1H-NMR spectra for the titration of CAudppf with variable concentrations of C_{70} in toluene-d_8 at 298 K. The most significant chemical shifts of the corannulene unit (H^{10}, H^2, H^{cor}) have been labelled.

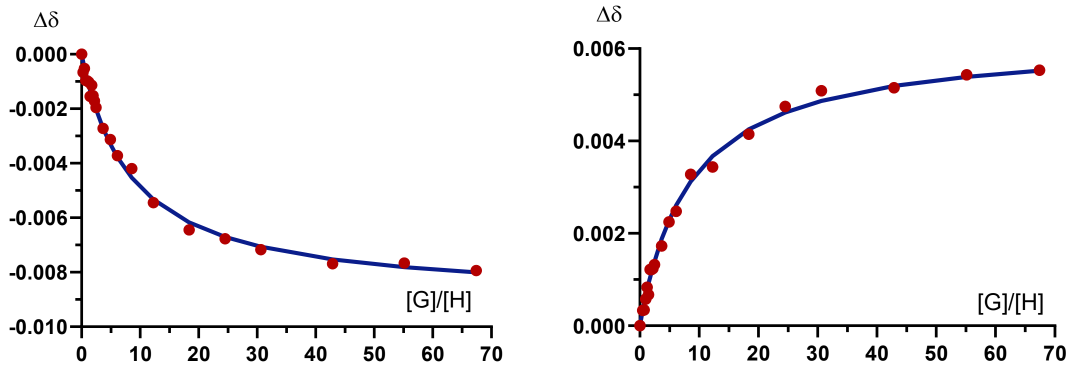

Figure S132. Non-linear regressions for selected protons (left plot: H^2, right plot: H^{cor}) for the titration of complex CAudppf with C_{70}.

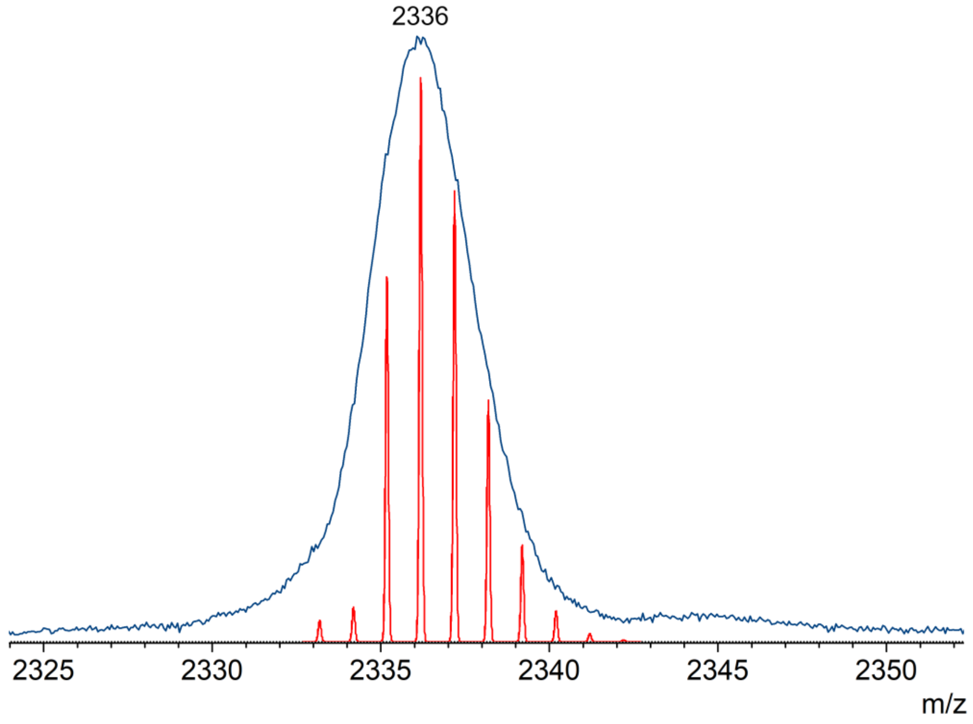

Figure S133. Detection of adduct C_{70}@CAudppf in LRMS (MALDI-TOF, linear detection, positive mode). Calculated (red), measured (blue).

CAudppbenz *vs*. C_{60}

1:1

http://app.supramolecular.org/bindfit/view/2681de44-aa12-41e8-b255-82c62cea2885

CAudppbenz *vs*. C_{70}

1:1

http://app.supramolecular.org/bindfit/view/99381b5d-636a-48b8-a02d-5326da312b94

Table S6. Association constants (M^{-1}) and and ΔG_a (kJ/mol) of host CAudppbenz with fullerenes according to a 1:1 model.

Guest	K_a	ΔG_a
C_{60}	$2.89 \pm 0.04 \cdot 10^2$	-14.04 ± 0.03
C_{70}	$5.93 \pm 0.05 \cdot 10^2$	-15.82 ± 0.02

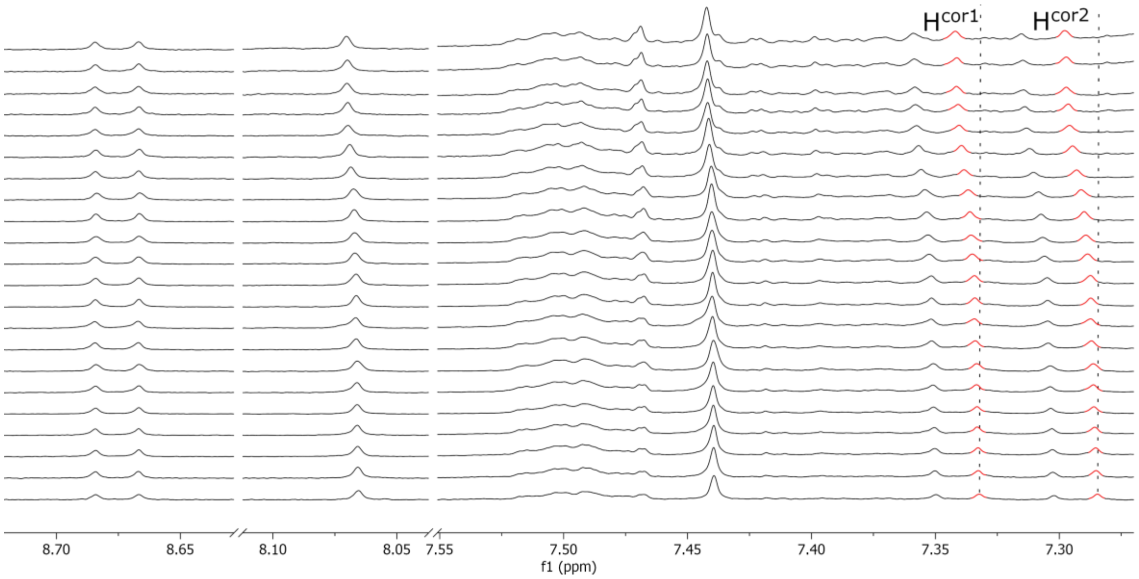

Figure S134. Stacked ^{1}H-NMR spectra for the titration of CAudppbenz with variable concentrations of C_{60} in toluene-d_8 at 298 K. The most significant chemical shifts of the corannulene unit (H^{cor1}, H^{cor2}) have been labelled.

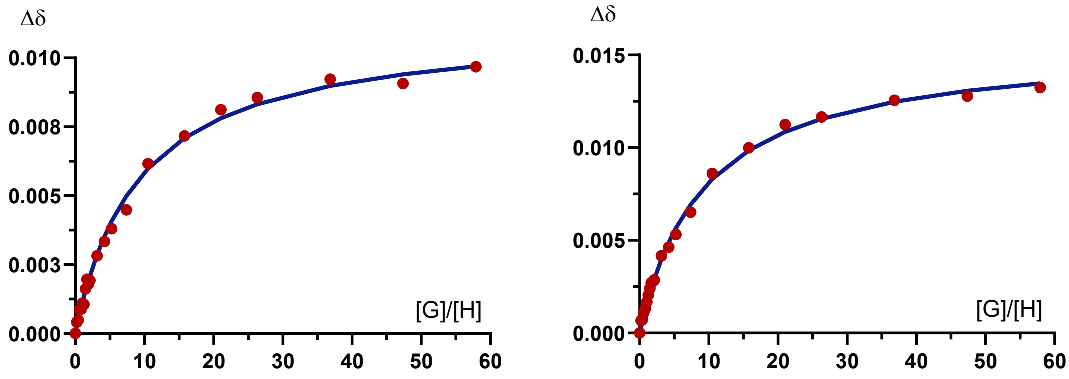

Figure S135. Non-linear regressions for selected protons (left plot: H^{cor1}, right plot: H^{cor2}) for the titration of complex CAudppbenz with C_{60}.

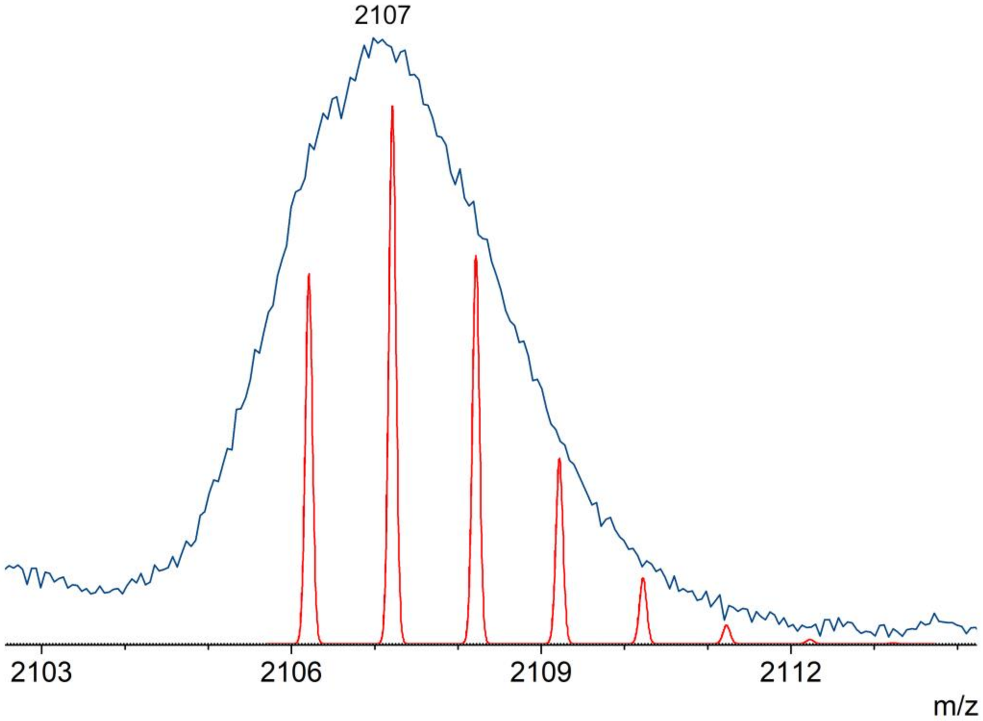

Figure S136. Detection of adduct C_{60}@CAudppbenz in LRMS (MALDI-TOF, linear detection, positive mode). Calculated (red), measured (blue).

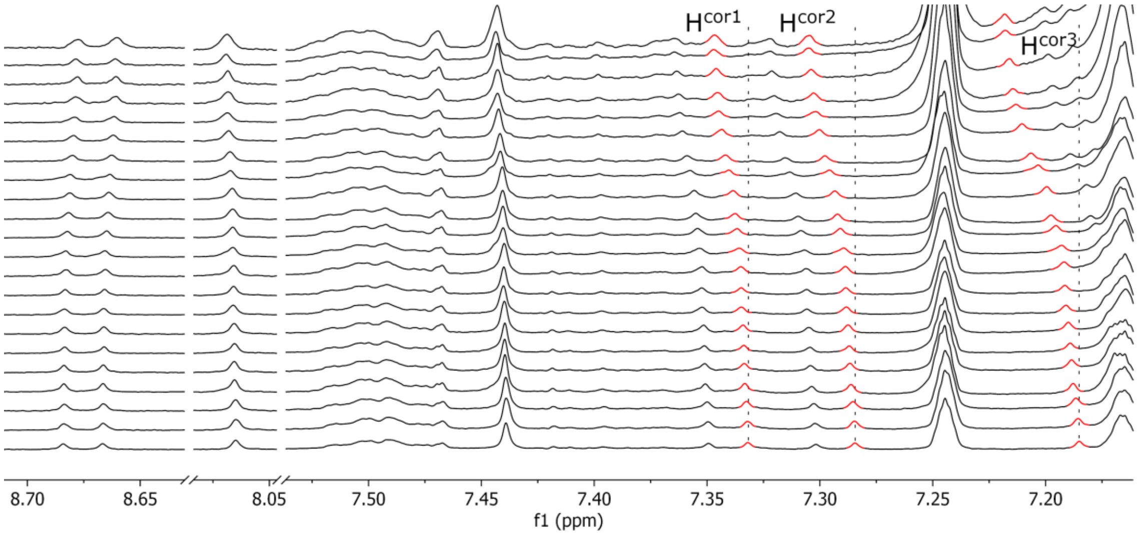

Figure S137. Stacked ^{1}H-NMR spectra for the titration of CAudppbenz with variable concentrations of C_{70} in toluene-d_8 at 298 K. The most significant chemical shifts of the corannulene unit (H^{cor1}, H^{cor2}, H^{cor3}) have been labelled.

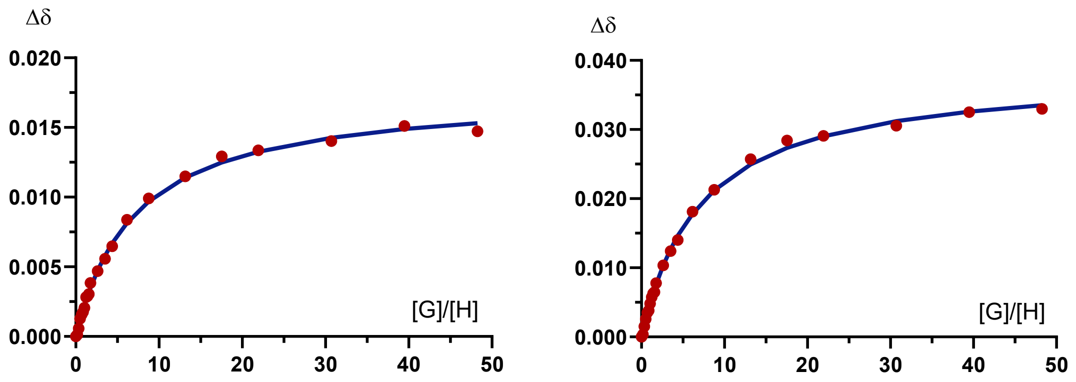

Figure S138. Non-linear regressions for selected protons (left plot: H^{cor1}, right plot: H^{cor3}) for the titration of complex CAudppbenz with C_{70}.

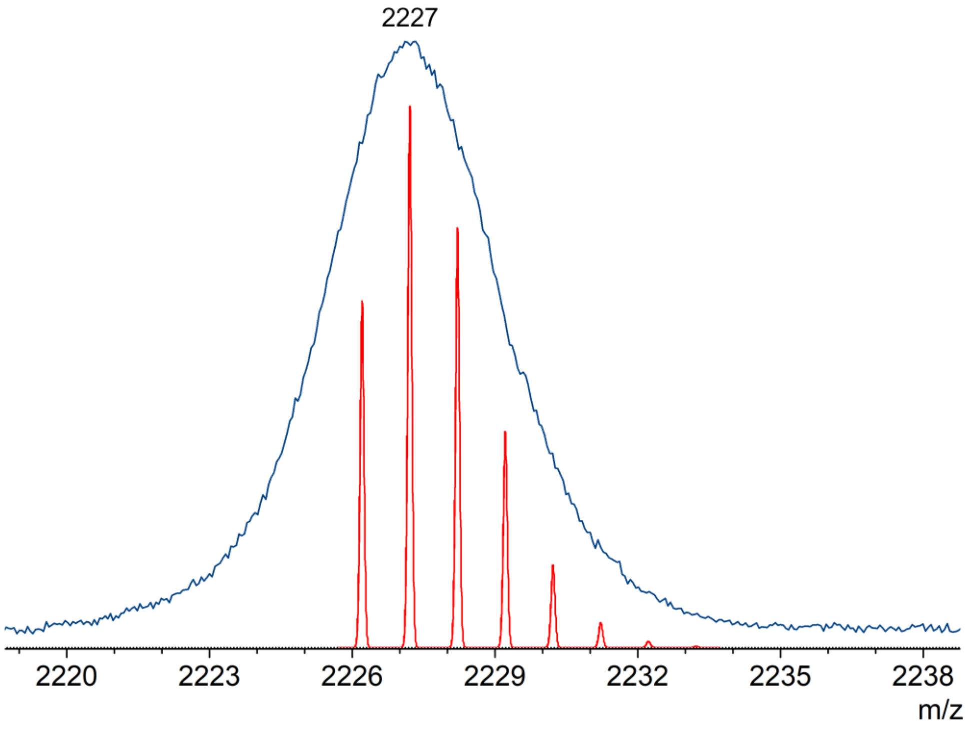

Figure S139. Detection of adduct C_{70}@CAudppbenz in LRMS (MALDI-TOF, linear detection, positive mode). Calculated (red), measured (blue).

CAuxantphos *vs.* C_{60}

1:1

http://app.supramolecular.org/bindfit/view/f314eb5c-200d-40d9-a743-708b5824dfaf

CAuxantphos *vs.* C_{70}

1:1

http://app.supramolecular.org/bindfit/view/803e855e-9d94-4b08-a509-d1f9838e919d

Table S7. Association constants (M^{-1}) and and ΔG_a (kJ/mol) of host CAuxantphos with fullerenes according to a 1:1 model.

Guest	K_a	ΔG_a
C_{60}	$1.18 \pm 0.01 \cdot 10^2$	-11.82 ± 0.03
C_{70}	$8.23 \pm 0.17 \cdot 10^2$	-16.63 ± 0.05

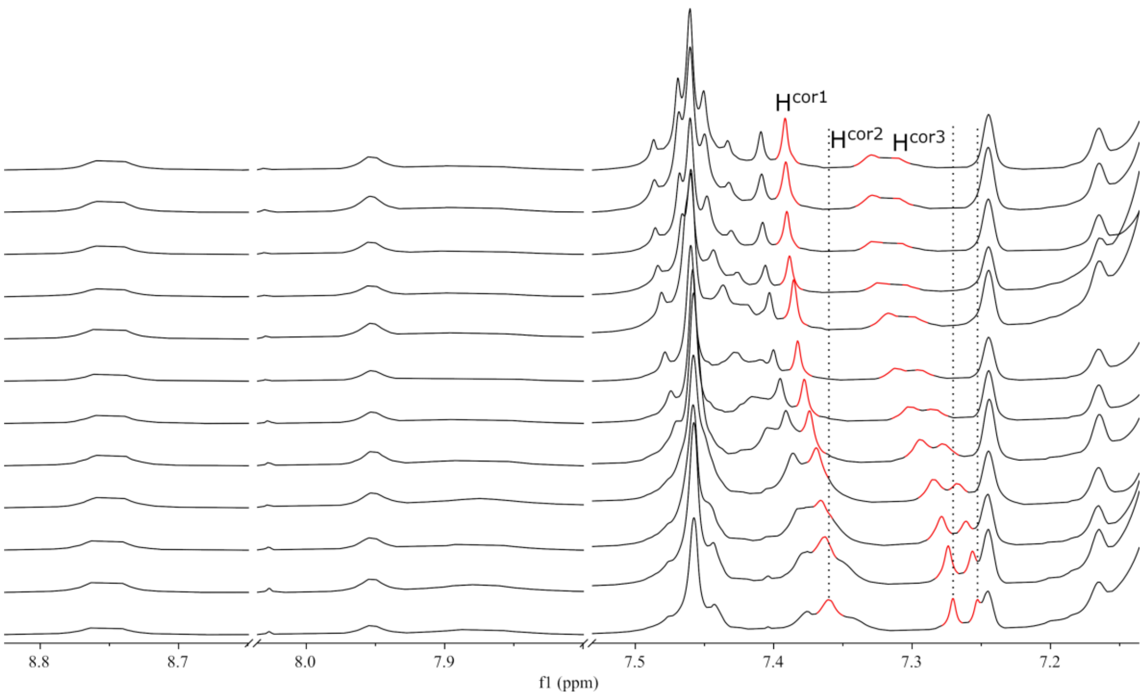

Figure S140. Stacked ^{1}H-NMR spectra for the titration of CAuxantphos with variable concentrations of C_{60} in toluene-d_8 at 298 K. The most significant chemical shifts of the corannulene unit (H^{cor1}, H^{cor2}, H^{cor3}) have been labelled.

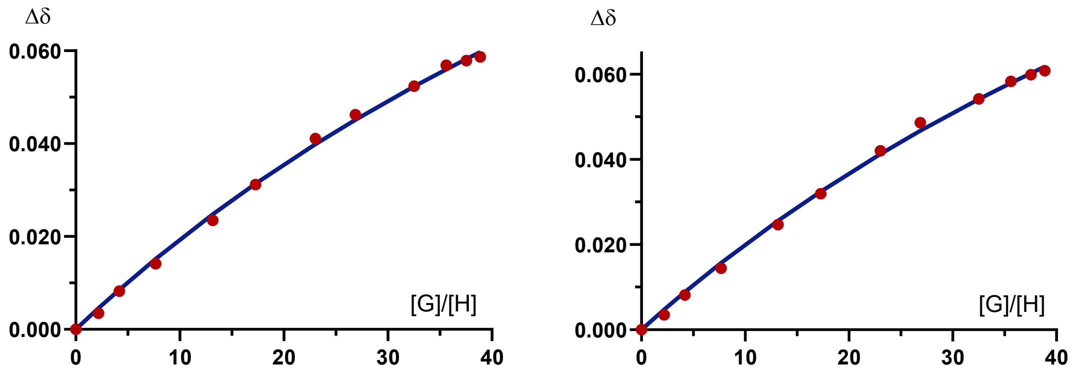

Figure S141. Non-linear regressions for selected protons (left plot: H^{cor2}, right plot: H^{cor3}) for the titration of complex CAuxantphos with C_{60}.

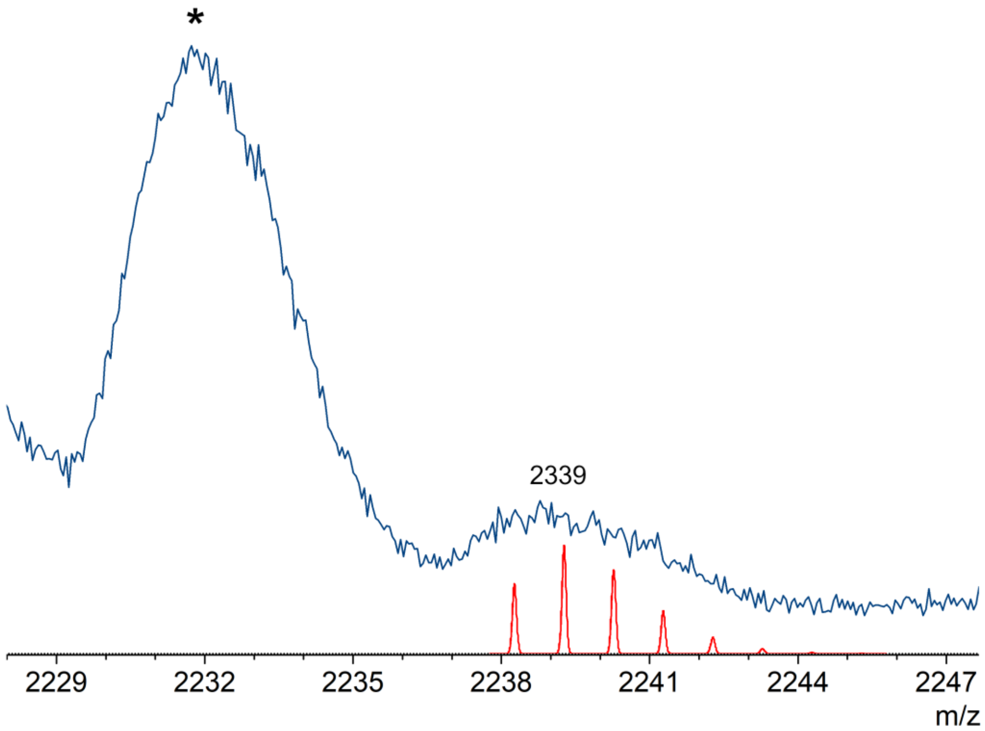

Figure S142. Detection of adduct C_{60}@CAuxantphos in LRMS (MALDI-TOF, linear detection, positive mode). Calculated (red), measured (blue). *Peak could not be assigned.

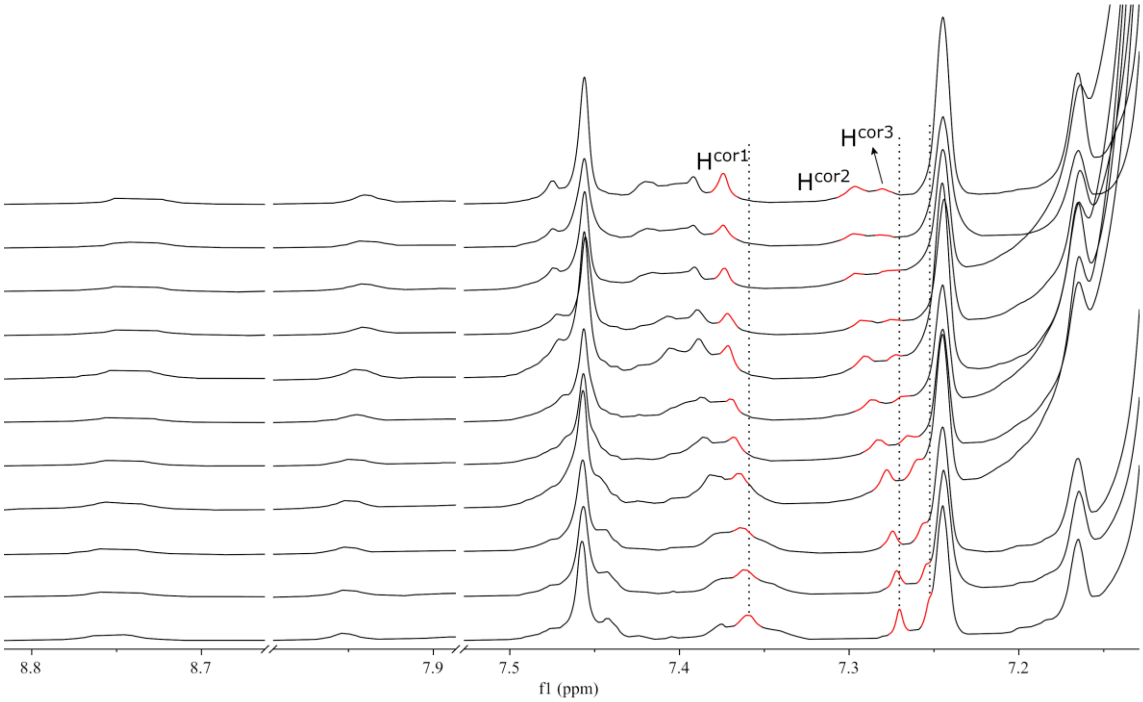

Figure S143. Stacked ^{1}H-NMR spectra for the titration of CAuxantphos with variable concentrations of C_{70} in toluene-d_8 at 298 K. The most significant chemical shifts of the corannulene unit (H^{cor1}, H^{cor2}, H^{cor3}) have been labelled.

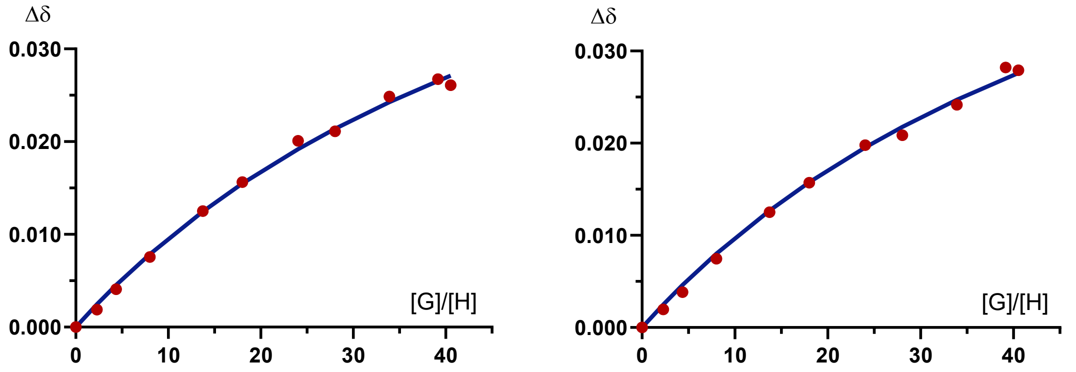

Figure S144. Non-linear regressions for selected protons (left plot: H^{cor2}, right plot: H^{cor3}) for the titration of complex CAuxantphos with C_{70}.

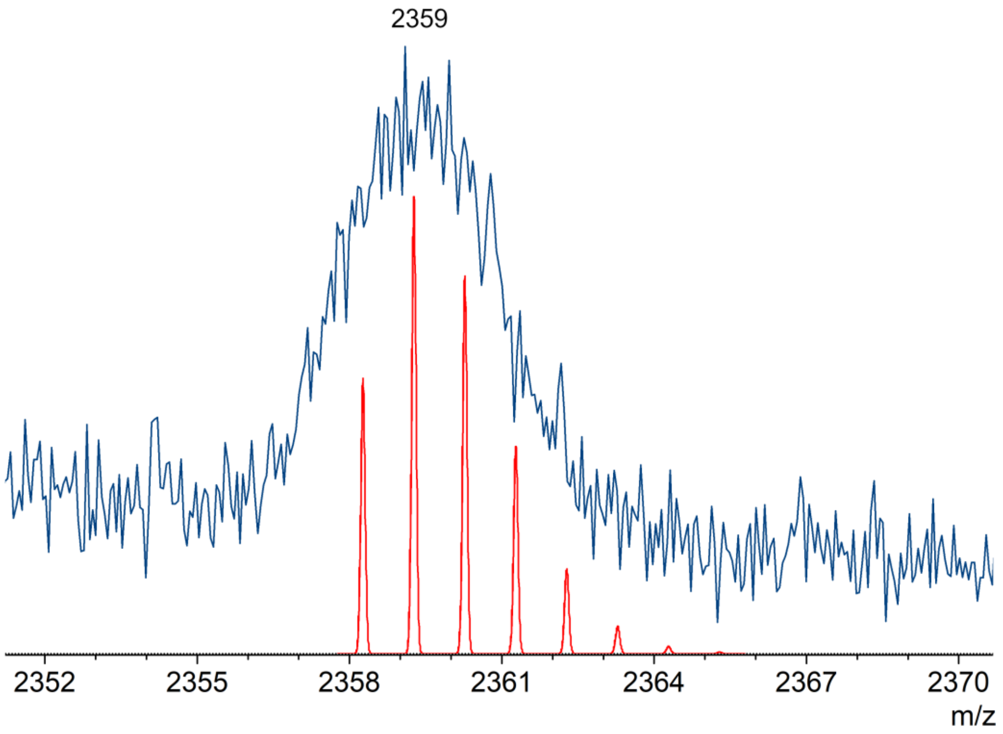

Figure S145. Detection of adduct C_{70}@CAuxantphos in LRMS (MALDI-TOF, linear detection, positive mode). Calculated (red), measured (blue).

Computational Calculations details

In order to obtain the optimized geometries of complexes **CAudppe**, **CAudppf**, **CAudppbenz** and **CAuxantphos** reported in this work, calculations were first performed using Grimme's GFN2-xTB[6] semiempirical quantum mechanical method with toluene as the solvent of choice. Two possible conformers were defined (v_1 and v_2) depending on the relative orientation of the corannulene moieties (exchangeable by C-C single bond rotation and corannulene face inversion). The most stable conformer in each case was used for further optimization by DFT methods. A previous study was carried out by testing CAM-B3LYP,[7] ωB97XD,[8] PBE0[9] and TPSSh[10] on complex **PAudppe** in order to find the best functional in terms on accuracy (based on RMSD criteria) and required time to completion. The best performance was found for the PBE0-D3BJ/LANL2DZ//Def2TZVP/PCM(toluene)[9,11] level of theory. Gaussian 16 Rev C.01 package was used.[12] The resulting optimized structures were confirmed as minima by vibrational analysing using GFN2-xTB and their geometries are shown below.

CAudppe – v_2 (G = -3643.039183 a.u.)

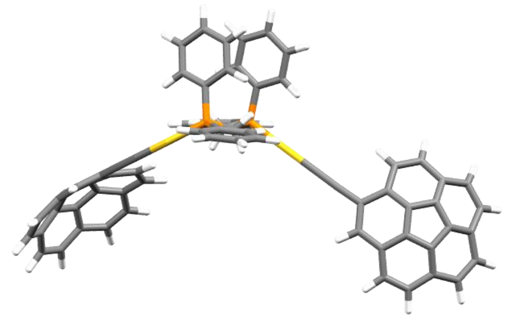

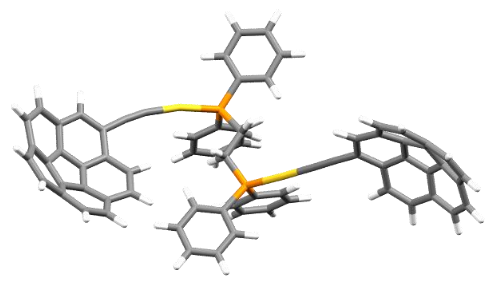

Au	-3.36263100	-2.19194700	-1.59153100	H	2.37383000	-5.11442900	-3.93323000	H	-4.73031400	6.03465800	2.15206100
Au	2.29294000	-0.12445100	0.98683100	H	1.38020100	-3.90393300	-2.02354600	H	-3.85270100	3.40937200	1.71922300
C	-4.89748800	-0.94169900	-1.58424800	H	-3.75164400	-4.11013300	0.46071800	H	-6.39989600	7.67884200	1.50590400
C	3.99196900	0.50515000	0.18345200	H	-4.07649700	-5.77450700	2.28755900	H	-8.64010400	8.11705800	-0.11922600
P	-1.48019600	-3.45126700	-1.23810000	H	-2.23108000	-7.33653500	2.88913400	H	-4.31012800	1.35862700	0.47266500
P	0.33604800	-0.92720800	1.85922100	H	-0.06490000	-7.23038300	1.66473200	H	-10.11376300	7.08630300	-1.75534100
C	-0.18604700	-2.39373900	-0.45824300	H	0.25522600	-5.58710700	-0.15777700	H	-10.62756800	4.72548600	-3.17673900
C	-0.85651200	-1.48753600	0.57862000	H	-1.48249700	-2.46880600	3.60430800	H	-7.95000900	0.54738200	-2.79424400
C	-0.66908000	-4.29936900	-2.62569300	H	-1.20723400	-4.61165000	4.81462500	H	-9.86876600	2.44489000	-3.53898300
C	-1.49020300	-4.89937700	-3.59288500	H	0.97948300	-5.80272300	4.76990800	C	7.52919800	0.85307400	-0.24037000
C	-0.92286200	-5.58711500	-4.66408300	H	2.89268700	-4.84215900	3.49621000	H	9.10813100	5.49768100	-3.78861800
C	0.46687100	-5.66943400	-4.78588000	H	2.61435700	-2.68863200	2.26927700	C	9.95111300	1.28418800	-0.26103200
C	1.28854200	-5.06248000	-3.83438300	H	-2.59130500	-0.26395300	2.10708200	C	10.16582500	3.99971400	-2.57833500
C	0.72528100	-4.38051400	-2.75435100	H	-3.78361000	1.31537000	3.57089100	H	12.88326300	3.04502800	1.53008200
C	-1.72756800	-4.73238100	0.04398700	H	-2.52573000	2.70031500	5.21711400	C	8.95597400	0.45883600	1.74334400
C	-2.94562600	-4.79514100	0.73430700	H	-0.04461000	2.50584000	5.36440100	C	11.34127000	4.68425700	-2.09520800
C	-3.12420500	-5.72976400	1.75680600	H	1.17076600	0.93803900	3.86854900	H	9.01590000	0.17890700	2.79803900
C	-2.09074900	-6.60501100	2.09155200	C	5.04641400	0.87884300	-0.32983100	C	9.97616300	2.72858700	-2.04719100
C	-0.87492500	-6.54781900	1.40366100	C	-5.77432000	-0.09522900	-1.41105800	C	12.09562400	4.19444900	-1.03353200
C	-0.69293400	-5.61736400	0.38323900	C	-7.13796000	3.91394600	1.07400100	C	11.74702700	2.97162200	-0.34868500
C	0.54881400	-2.45026600	2.84669500	C	-8.11253000	4.87448400	0.69602900	H	11.59469600	5.66580100	-2.50344000
C	-0.52442600	-2.99205200	3.57003400	C	-7.39910800	2.71842400	0.35251700	C	6.28713100	1.27967200	-0.88506900
C	-0.36786000	-4.19359400	4.25718500	C	-8.97516800	4.27237700	-0.25823400	C	11.29015400	1.62571300	1.68287800
C	0.85957600	-4.86236700	4.22923500	C	-8.53642500	2.94044800	-0.46780300	C	10.11402000	1.02714400	1.09550000
C	1.93125200	-4.32646200	3.51438800	C	-5.86496700	4.26717200	1.50807400	C	7.55428300	2.69063300	-2.50775700
C	1.77895100	-3.12130500	2.82485500	C	-7.87026500	6.24291200	0.73025200	C	6.32560200	2.17093100	-1.97547100
C	-0.62454900	0.21231900	2.90564400	C	-6.40075800	1.81261400	0.02903200	H	6.85046800	0.04154300	1.67180900
C	-2.02058400	0.32785800	2.82381400	C	-9.64279700	5.00587800	-1.23299500	H	6.93571000	4.40881500	-3.73042000
C	-2.69982300	1.22215200	3.65395400	C	-8.72975500	2.26446000	-1.66848900	C	8.69155600	1.20040200	-0.91218000
C	-1.99254300	2.00021900	4.57197200	C	-5.68480700	5.67863400	1.75646400	H	12.91311000	4.80922900	-0.64894300
C	-0.60155100	1.89079100	4.65598000	C	-4.87043600	3.23112200	1.36263800	C	10.74473900	2.22932900	-0.96245200
C	0.08156800	1.00703900	3.82258500	C	-6.63974500	6.61947700	1.38589900	C	12.06759600	2.55186000	0.99552200
H	0.31534800	-1.79524000	-1.23126600	C	-8.72608100	7.02777800	-0.12852900	C	7.72173800	0.37726800	1.10544100
H	0.56851100	-3.04780100	0.00529600	C	-5.12753600	2.05975200	0.65551500	C	8.71000700	2.09112500	-2.01738000
H	-1.29671600	-0.60712800	0.09011000	C	-6.70389100	0.92640100	-1.09657700	C	7.78275300	3.90568900	-3.25786000
H	-1.67183300	-2.01767200	1.09587900	C	-9.56961700	6.43767300	-1.06439700	C	9.02546300	4.52860900	-3.29026700
H	-2.57571000	-4.81382300	-3.50569100	C	-10.04662300	4.23957200	-2.38896700	H	11.52452100	1.42681600	2.73161600
H	-1.56737700	-6.04994500	-5.41280500	C	-7.83149000	1.16776000	-1.90367100	H	5.36927700	2.54478300	-2.34663300
H	0.91029700	-6.19962000	-5.63007200	C	-9.61333000	2.93464800	-2.59625800				

CAudppf – v_2 (G = -5213.549346 a.u.)

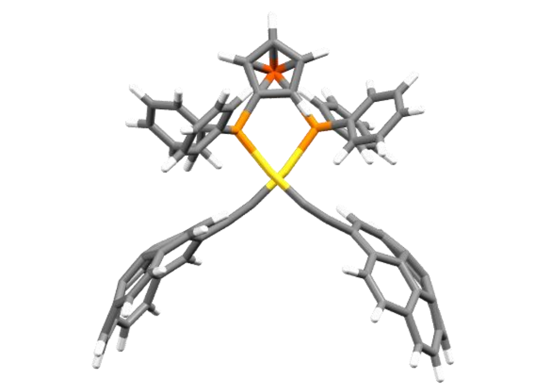

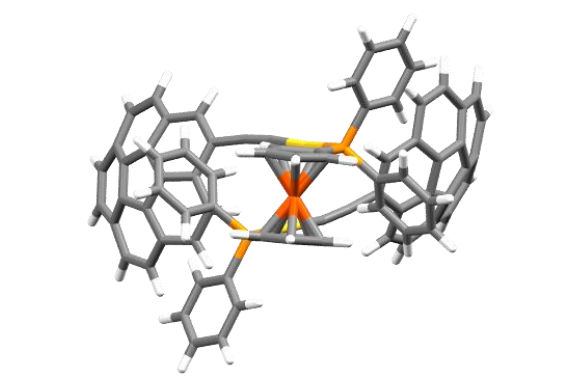

Au	-0.78453600	-0.61984500	-1.47525400
Fe	-0.39697100	-4.86096500	0.07875900
P	-2.33774300	-2.29396100	-1.27761400
C	0.74028100	0.64341000	-1.66152900
C	1.82524100	1.21632300	-1.55140400
C	-3.40785500	-2.30776000	0.19521100
C	-3.11227200	-1.44787400	1.25923600
H	-2.26198300	-0.76522000	1.19824100
C	-3.91407600	-1.44801400	2.40212300
H	-3.67903400	-0.76077700	3.21639800
C	-5.01099800	-2.30640200	2.48633600
H	-5.64211400	-2.30208100	3.37667900
C	-5.31710700	-3.15798100	1.41900900
H	-6.18655400	-3.81529000	1.47531400
C	-4.52489100	-3.15490400	0.27201200
H	-4.79081600	-3.78996100	-0.57526900
C	-3.52682600	-2.32935400	-2.66263100
C	-4.52426300	-1.34153100	-2.69900600
H	-4.59872100	-0.60901400	-1.89198500
C	-5.41904400	-1.29361600	-3.76581100
H	-6.19296600	-0.52441400	-3.78542700
C	-5.32343300	-2.22414200	-4.80537000
H	-6.02453300	-2.18402100	-5.64055000
C	-4.32910300	-3.20295200	-4.77383700
H	-4.25021900	-3.93010100	-5.58377300
C	-3.43009500	-3.25784300	-3.70579300
H	-2.65350700	-4.02386800	-3.68404600
C	-1.49893700	-3.89174800	-1.34676500
C	-0.13359900	-4.04813800	-1.78436900
H	0.53757900	-3.23157900	-2.03436400
C	0.17585700	-5.44078700	-1.80408900
H	1.14020800	-5.87082800	-2.06074200
C	-0.98928500	-6.15980500	-1.38031800
H	-1.06656400	-7.23731600	-1.25967600
C	-2.02176100	-5.21095900	-1.09094700
H	-3.01788000	-5.44095300	-0.72482900
Au	0.77936600	-0.65918300	1.40784600
P	1.99779500	-2.59047000	1.20843300
C	-0.47927000	0.82363100	1.82740000
C	-1.41647000	1.60021700	2.01650000
C	3.00963800	-2.93872300	-0.26649500
C	2.87804600	-2.12320000	-1.39607300
H	2.19411000	-1.27146400	-1.38138500
C	3.63343500	-2.38889900	-2.54040000
H	3.53059600	-1.73839800	-3.41037500
C	4.51992700	-3.46627900	-2.56056300
H	5.11287100	-3.67084000	-3.45368800
C	4.66718200	-4.27203200	-1.42615500
H	5.37663100	-5.10139300	-1.43189800
C	3.92259900	-4.00565700	-0.27839400
H	4.07153400	-4.60950800	0.61875000
C	3.20539900	-2.73178400	2.57217300
C	4.37967200	-1.96532000	2.49492300
H	4.57767500	-1.35604400	1.61010800
C	5.29667200	-1.98354400	3.54356800
H	6.20686900	-1.38533800	3.47387900
C	5.04898900	-2.76241500	4.67834500
H	5.76723100	-2.77485500	5.49965500
C	3.88131900	-3.52261400	4.75900100
H	3.68395300	-4.13102200	5.64322000
C	2.95850700	-3.50839900	3.71013300
H	2.04485700	-4.10098700	3.77770500
C	0.88004400	-3.99465500	1.42335800
C	-0.48681000	-3.85337800	1.86209200
H	-0.98924800	-2.90597600	2.03532500
C	-1.05203200	-5.15574600	2.00099000
H	-2.07751000	-5.37302000	2.28723400
C	-0.04657400	-6.11504300	1.65097300
H	-0.17374600	-7.19430200	1.62614100
C	1.14304000	-5.40599600	1.28788200
H	2.07414600	-5.85147500	0.95102200
C	6.76050700	3.42065700	-0.32969400
C	5.72518600	2.49967600	-0.63099000
C	6.81324300	4.37926800	-1.37642300
C	5.13274600	2.88938900	-1.86122200
C	5.80775000	4.05048600	-2.32309500
C	7.15427400	3.71000700	0.97260700
C	5.01238500	1.82344400	0.35498300
C	7.26396000	5.67967200	-1.18082200
C	3.81074400	2.61971200	-2.18622900
C	5.19564300	5.00324800	-3.12977700
C	6.58107800	2.84718000	1.98009200
C	7.82400600	4.97885400	1.13065000
C	5.56101600	1.94863200	1.68738000
C	3.72244800	1.34570700	-0.05046600
C	7.87749200	5.91736800	0.10468800
C	6.79652700	6.63138900	-2.16143300
C	3.12603900	1.70564200	-1.27760700
C	3.24050600	3.48025900	-3.19061800
C	5.81208300	6.30897300	-3.08959500
C	3.90198300	4.61843000	-3.64201400
H	6.87871800	2.97330000	3.02411500
H	8.21404200	5.25941000	2.11222800
H	5.08958000	1.40668000	2.51132200
H	3.10564300	0.76937100	0.64558200
H	8.30831900	6.89844900	0.31915500
H	7.14859300	7.66486600	-2.11444000
H	2.21857300	3.29150500	-3.52648500
H	5.42791900	7.10165000	-3.73637200
H	3.37530700	5.28927000	-4.32513500
C	-5.31346700	3.08492800	-1.18447000
H	-3.49260300	5.62771400	4.73153700
H	-3.86401300	1.98472700	-2.41937600
C	-5.99375400	3.95412000	-2.11631300
H	-6.87963200	8.00836200	1.96892700
C	-3.56066300	2.28131100	1.00135100
C	-2.59847200	2.37537800	2.10240100
C	-3.32486400	1.86062100	-0.35472700
H	-7.55930900	7.48360500	-0.30578500
C	-5.16329600	6.26735300	3.55916800
C	-4.77141200	2.92086700	1.21720700
H	-5.18966400	7.23522800	4.06596200
C	-6.72700000	5.05800800	-1.69410500
H	-5.84511900	3.80813400	-3.18920000
C	-2.82888100	3.26908300	3.16538500
C	-4.16261700	2.24054800	-1.39938700
C	-4.19287000	5.34738500	3.94095900
C	-3.99932900	4.10017300	3.23573800
H	-2.39917200	1.32660300	-0.58964200
C	-6.73835700	6.98700600	1.60669000
C	-6.85289600	5.40349400	-0.29749800
H	-7.12919200	5.73577100	-2.45121100
C	-5.97813100	4.74612300	1.91258900
C	-5.62630700	3.31180500	0.15189500
C	-7.12702600	6.68709600	0.30482200
C	-4.99209000	3.80599100	2.30549900
C	-6.37284900	4.44010100	0.58255200
C	-6.03994700	6.03360900	2.43527000
H	-2.03118600	3.38563900	3.90177300

CAudppbenz – v_1 (G = -3795.326357 a.u.)

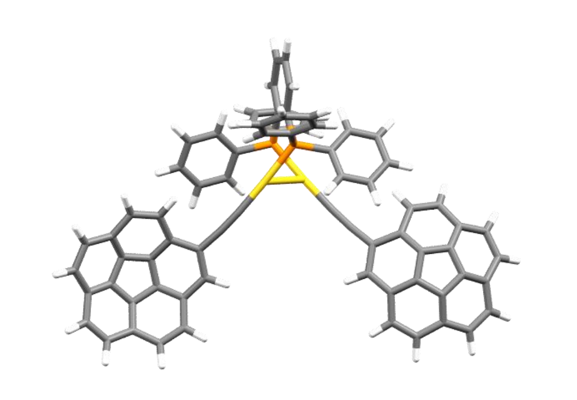

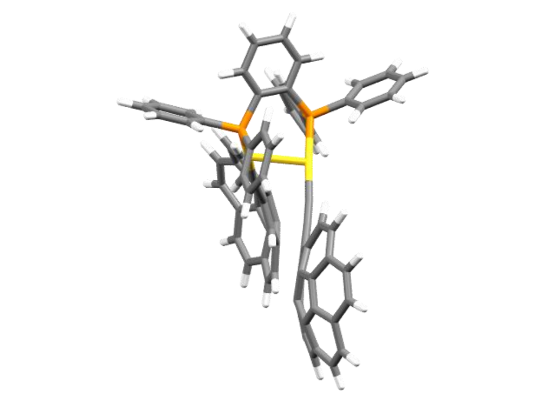

Au	1.06186300	-1.00052000	1.18808400	H	2.73504800	-4.26328900	-0.77172400	C	4.19321500	2.19717600	0.06346900
Au	-1.06227100	-1.00209000	-1.18916300	C	4.41322000	-2.97746500	-1.21282800	C	6.13722000	0.52397900	0.56942600
C	2.34436600	0.46467900	0.81558300	H	5.14773200	-3.68094300	-0.81710100	H	8.89856700	7.47194900	0.37096300
C	-2.34490000	0.46315700	-0.81725900	C	4.82819300	-1.75192900	-1.73713900	H	6.13489900	7.71190200	-0.03667200
P	-0.32645000	-2.75134700	1.70713500	H	5.88630600	-1.48711500	-1.73983500	H	10.69109400	5.89390300	0.82699300
P	0.32597000	-2.75342600	-1.70650700	C	3.88457200	-0.84660300	-2.22902200	H	11.26885900	3.18791000	1.27636300
C	-2.11057100	-2.39570600	1.68180600	H	4.20415500	0.12501900	-2.60707800	H	4.04926500	6.46837200	-0.14317300
C	-2.52796000	-1.15931600	2.19836200	C	2.52953900	-1.16366200	-2.19610200	H	2.91490100	3.91316500	0.04781800
H	-1.78461900	-0.43603200	2.53915000	H	1.78730300	-0.43976100	-2.53797500	H	10.28839000	0.97086900	1.45197100
C	-3.88262500	-0.84083400	2.23248600	C	0.00298800	-3.32583600	-3.41359400	H	7.87764400	-0.45298800	1.34225700
H	-4.20082200	0.13129900	2.61040300	C	-1.32984500	-3.38297200	-3.84936100	H	5.46640100	-0.23108500	0.98454100
C	-4.82768100	-1.74541600	1.74198900	H	-2.12951600	-3.06344600	-3.17704400	C	-3.92731100	3.56161800	0.15820800
H	-5.88551500	-1.47951100	1.74555500	C	-1.62641100	-3.82395600	-5.13771300	H	-6.13338300	7.71147600	0.03408800
C	-4.41449600	-2.97162500	1.21786200	H	-2.66470000	-3.86340400	-5.47072700	C	-10.31368800	3.00162400	-0.77942300
H	-5.15012700	-3.67450900	0.82316400	C	-0.59543800	-4.19425400	-6.00624900	H	-4.04806700	6.46738900	0.14023600
C	-3.05832100	-3.30255600	1.19016700	H	-0.82817600	-4.52702100	-7.01906700	H	-7.87890000	-0.45333200	-1.34200600
H	-2.73812900	-4.25941500	0.77571700	C	0.73270300	-4.12344700	-5.58200100	C	-8.44688300	3.74033700	0.50792500
C	-0.00173600	-3.32266500	3.41428200	H	1.54071400	-4.40016300	-6.26120500	C	-7.51035500	0.40161500	-0.76935600
C	-1.03251800	-3.68751400	4.29070500	C	1.03455600	-3.69339900	-4.28797300	C	-4.19330000	2.19617200	-0.06522100
H	-2.07132300	-3.62629100	3.96293300	H	2.07293600	-3.63366200	-3.95859100	C	-9.77520200	5.52148100	-0.36135400
C	-0.72931500	-4.11677500	5.58468600	C	-3.17455000	1.31183100	-0.49254800	C	-6.47421200	2.56810500	0.61142200
H	-1.53670700	-4.39137900	6.26548200	C	3.17410500	1.31322100	0.49074400	C	-6.18336900	3.93813700	0.84380200
C	0.59938500	-4.18949800	6.00683900	C	7.40121800	4.66224300	-0.77754200	C	-4.95298100	4.50082000	0.51773000
H	0.83319500	-4.52165500	7.01961000	C	8.44743100	3.73998300	-0.50867800	C	-9.61239500	4.10096600	-0.15852800
C	1.62959200	-3.82189900	5.13624100	C	6.18408500	3.93833000	-0.84536100	H	-10.28927100	0.97121600	-1.45136900
H	2.66835100	-3.86284300	5.46760900	C	7.87485200	2.44511100	-0.40664900	C	-6.13793800	0.52337700	-0.57005700
C	1.33170700	-3.38169300	3.84793400	C	6.47446400	2.56827700	-0.61252300	C	-9.75309100	1.73259000	-0.87934900
H	2.13085300	-3.06424900	3.17400700	C	7.46174400	5.99728500	-0.39258300	C	-4.98186700	5.93540000	0.33734700
C	-0.12580300	-4.28436900	0.69817900	C	9.61282400	4.10046100	0.15806500	H	-2.91448400	3.91179700	-0.05043600
C	-0.24410200	-5.51379000	1.36459900	C	4.95375000	4.50144800	-0.51984100	C	-8.74894400	6.42424400	-0.10030700
H	-0.42758300	-5.52020400	2.43909400	C	8.43433900	1.43569800	0.36885600	C	-8.43473100	1.43581100	-0.36899400
C	-0.12542000	-6.72715900	0.68738500	C	5.56083000	1.68759800	-0.05372500	C	-5.56101700	1.68701900	0.05255300
H	-0.22515600	-7.66503200	1.23585900	C	8.75004800	6.42396600	0.09893600	C	-7.40032000	4.66237400	0.77619300
C	0.11754800	-6.72803600	-0.68322600	C	6.17646100	6.64689200	-0.27816300	C	-7.46059700	5.99733200	0.39090000
H	0.21543800	-7.66661800	-1.23082100	C	9.77596500	5.52098400	0.36056500	H	-10.69038100	5.89453400	-0.82757700
C	0.23865000	-5.51554100	-1.36157600	C	10.31360100	3.00109000	0.77948900	C	-6.17516800	6.64654400	0.27588200
H	0.42220600	-5.52331300	-2.43604500	C	4.98298100	5.93606800	-0.33983200	H	-11.26905600	3.18857900	-1.27603200
C	0.12271600	-4.28527200	-0.69631400	C	3.92769300	3.56263700	-0.16040900	H	-5.46747100	-0.23203000	-0.98511800
C	2.11036100	-2.39934300	-1.67931700	C	9.75261400	1.73224200	0.87957000	C	-7.87470300	2.44527700	0.40605300
C	3.05667200	-3.30696200	-1.18630900	C	7.50953700	0.40188300	0.76921100	H	-8.89725900	7.47219600	-0.37256400

CAuxantphos – v_1 (G = -4217.863831 a.u.)

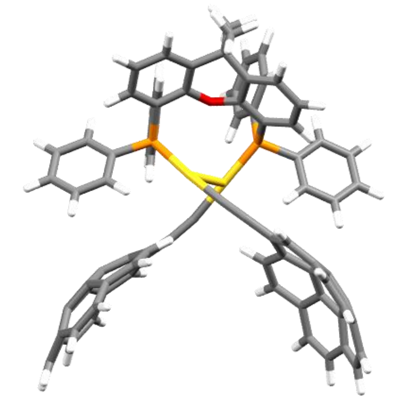

Au	0.59379200	-0.83545700	-1.22396100	C	3.85649600	-3.83019400	2.59622200	C	-3.06187400	-1.64129800	-0.83364900
Au	0.88616700	0.24405200	1.57418900	H	4.89969100	-4.07516600	2.80173200	C	-3.04973400	-2.06079900	1.74671000
P	1.54071600	-2.85909100	-0.67180900	C	2.91883300	-3.82384300	3.63024700	C	-7.26372000	-3.24784000	-2.67063000
P	2.95424800	1.17472600	1.19695400	H	3.22630100	-4.07340300	4.64750900	C	-8.70386800	-4.16421800	-0.69560500
O	3.85052200	-1.00535400	-0.81726700	C	1.58816200	-3.49180400	3.36311000	C	-3.84660500	-2.55307300	2.79647700
C	3.00655700	-3.03794500	-1.73951700	H	0.85057000	-3.47358300	4.16683700	C	-6.14360100	-3.62570900	3.40165600
C	3.95524600	-1.99988400	-1.78037600	C	1.19123900	-3.17402100	2.06514800	C	-8.72530800	-4.36969800	0.68039900
C	4.23787500	0.27014000	-1.19062200	H	0.15330500	-2.90414400	1.86697900	C	-7.35038400	-4.08047600	2.88022100
C	3.73821200	1.36304600	-0.45585000	C	2.85148700	2.89807000	1.79565600	H	-3.56581600	-1.12617500	-2.84496500
C	4.01227500	2.64945300	-0.94177500	C	1.70428700	3.62730900	1.44374300	H	-5.98189400	-2.06995300	-3.91355900
H	3.62071100	3.51350500	-0.40738400	H	0.92294900	3.15525200	0.84539800	H	-2.29474900	-0.86472400	-0.78625500
C	4.78288200	2.83793300	-2.08615500	C	1.55914600	4.94992300	1.85761400	H	-8.11093600	-3.09475400	-3.34377200
H	4.95694000	3.84611300	-2.46298000	H	0.66239500	5.50385300	1.57429600	H	-9.65674600	-4.07821500	-1.22360400
C	5.37960700	1.74255300	-2.70663200	C	2.55219800	5.54742800	2.63901100	H	-3.62598300	-2.19427800	3.80398300
H	6.04901600	1.90370900	-3.55118000	H	2.43468400	6.57923000	2.97437700	H	-6.10972900	-3.33193600	4.45364000
C	5.14506400	0.44347300	-2.24750900	C	3.69010900	4.82156200	2.99940700	H	-9.69465700	-4.43705800	1.18058700
C	5.92375400	-0.77906100	-2.72807700	H	4.46251000	5.28511800	3.61556200	H	-8.21781500	-4.12763300	3.54331900
C	4.95375700	-1.95059300	-2.75851500	C	3.84632300	3.49990100	2.57569100	C	-4.84770900	6.78233800	1.82148300
C	5.01722600	-2.99762300	-3.68499100	H	4.73541000	2.93550100	2.86053300	C	-4.26823000	5.61964500	2.31892200
H	5.78166600	-2.98138900	-4.46140300	C	4.28388800	0.40067800	2.18071700	C	-1.02895300	4.31942200	-2.80104100
C	4.12188400	-4.06466100	-3.63081600	C	3.92534400	-0.30143200	3.33914600	C	-1.73012400	6.12699300	-1.40654200
H	4.19493100	-4.87628900	-4.35555200	H	2.86791800	-0.42086700	3.58630500	H	-4.77580000	5.09135800	3.12972100
C	3.11496100	-4.07995500	-2.66784200	C	4.91101600	-0.85931700	4.15287200	C	-3.35346700	7.86197000	-2.06350200
H	2.38074200	-4.88579500	-2.65030700	H	4.62126100	-1.40730800	5.05051300	C	-1.65835200	5.27173800	-0.27491700
C	6.59825900	-0.54842100	-4.08277200	C	6.25827800	-0.73700200	3.80782900	H	-0.78329400	3.89812900	-3.77798100
H	7.18240100	-1.42973800	-4.37843900	H	7.02789300	-1.18236400	4.44069800	C	-4.98823100	8.34796500	-0.26320600
H	7.30521600	0.28956900	-4.02349400	C	6.62027600	-0.04892900	2.64665000	C	-2.12821800	6.49175200	-3.72898100
H	5.86454500	-0.33393300	-4.87215900	H	7.67200700	0.04594600	2.37067900	H	-5.78812400	7.12357900	2.26138700
C	7.01854400	-1.08169100	-1.66911000	C	5.63862000	0.52387900	1.83749300	C	-3.08328700	6.99957400	0.23167100
H	6.57659900	-1.25840600	-0.67883300	H	5.92504800	1.06643100	0.93527000	C	-1.39449100	3.91408300	-0.37180500
H	7.71269000	-0.23205300	-1.59084600	C	-0.10941500	0.95591800	-1.71444600	H	-5.13675600	9.13019400	-2.24881400
H	7.58634900	-1.97757300	-1.95986600	C	-0.89909800	-0.54965100	1.95356200	C	-4.52847200	8.53483900	-1.56330000
C	0.46283200	-4.28549400	-1.01975800	C	-1.94092600	-1.20724100	1.96693300	C	-3.10209600	5.02353000	1.70923500
C	-0.70214900	-4.07602100	-1.77053600	C	-0.49746800	2.12120000	-1.80332200	C	-0.94026100	3.46086800	-1.68905800
H	-0.92729600	-3.07567200	-2.14460400	C	-5.07308000	-3.53923200	-1.00330100	C	-2.49224800	5.81295600	0.73972000
C	-1.58745300	-5.12947100	-2.00121500	C	-4.32740800	-3.38386000	0.19517900	C	-4.31850000	7.46748300	0.66525600
H	-2.50768000	-4.94719700	-2.55806800	C	-6.31806500	-4.13479500	-0.66856700	H	-1.97691100	6.23187200	-4.77954700
C	-1.30817500	-6.39789900	-1.49017200	C	-5.10938500	-3.88632100	1.26903600	C	-2.99216100	7.53837900	-3.42401200
H	-2.00729600	-7.21887200	-1.65770400	C	-6.34014400	-4.34627900	0.73630700	C	-1.84182300	3.11914600	0.74360000
C	-0.14337300	-6.61418100	-0.74674900	C	-4.91297800	-2.69814800	-2.10026100	H	-3.03945500	2.97360800	2.50576600
H	0.06979600	-7.60381900	-0.33926700	C	-3.38188400	-2.38439800	0.35674600	C	-2.65334100	3.65182800	1.74113100
C	0.73830500	-5.56175500	-0.50428900	C	-7.47322800	-3.92872800	-1.41400500	H	-5.93937000	8.80385700	0.02259700
H	1.63110700	-5.72383000	0.10248400	C	-4.98334700	-3.40407000	2.56844600	C	-1.53957800	5.65963900	-2.70354700
C	2.13042700	-3.19005800	1.02253800	C	-7.51942400	-4.36217100	1.47385700	H	-3.48518100	8.06143100	-4.24727600
C	3.46518700	-3.51545000	1.29382800	C	-3.79077900	-1.79447800	-2.01008100	C	-2.61346600	7.19131000	-1.09528100
H	4.19956600	-3.53075500	0.48762100	C	-6.04509800	-2.66231800	-2.99742100	H	-1.61955600	2.04944900	0.76076300

CAuxantphos – v_2 (G = -4217.867132 a.u.)

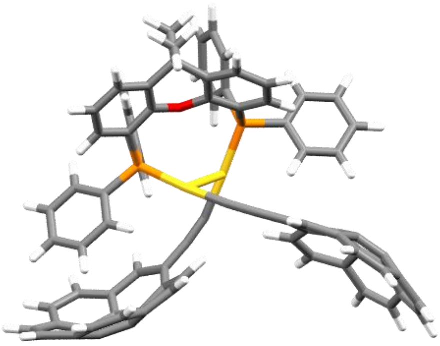

Au	-0.72996300	0.16557700	1.00131500	C	-3.39488900	4.14496500	-2.39991000	C	4.06203300	-1.70307800	0.25196700
Au	0.52528000	0.89069300	-1.62760300	H	-3.59498100	5.21739100	-2.41798200	H	9.12801300	-5.76398400	1.85704100
P	-2.69208300	1.22961900	0.42896400	C	-3.35768700	3.41644700	-3.58971400	C	8.58631300	-5.04082800	1.24224800
P	1.47423800	2.85109200	-0.90048900	H	-3.53739100	3.91730400	-4.54282700	C	5.33799900	-3.03864800	-1.99925100
O	-0.83209800	3.43421700	1.09989400	C	-3.08384800	2.04670500	-3.55872400	C	3.50493300	-1.47643000	1.58812600
C	-2.93102100	2.48624300	1.72718900	H	-3.04234400	1.47154700	-4.48520200	C	6.04639200	-2.61222800	-0.88059800
C	-1.88690900	3.38834600	2.00290000	C	-2.85525300	1.40332500	-2.34339700	C	7.69711800	-4.43977400	-0.99324800
C	0.42030900	3.72432400	1.61264200	H	-2.62825900	0.33699000	-2.32651800	H	6.02696700	-3.05927600	4.66005400
C	1.55520100	3.34184800	0.86973600	C	3.23127700	2.81973300	-1.39859700	C	7.44022900	-3.83845300	3.25628100
C	2.80986800	3.50267700	1.47505800	C	3.92698500	1.61532600	-1.20832400	C	3.35879800	-1.94565000	-0.98204200
H	3.70357700	3.19477100	0.93480500	H	3.41006400	0.74920500	-0.79177400	H	3.35681000	-2.82246000	-2.93005000
C	2.93069900	4.05531400	2.74723400	C	5.27445300	1.52107700	-1.55082500	C	3.97103800	-2.57874900	-2.05999400
H	3.91398400	4.14118700	3.21032400	H	5.80209400	0.57870700	-1.39341500	H	8.98496000	-6.16014300	-0.53712200
C	1.80212000	4.55213500	3.39530100	C	5.93097300	2.62553600	-2.10098900	C	5.61878600	-2.26470800	2.65248100
H	1.91370700	5.05777400	4.35399200	H	6.98305100	2.55055100	-2.38097200	H	7.89832600	-4.47132900	4.02048000
C	0.53333200	4.42663700	2.82292100	C	5.23885600	3.82247600	-2.30184300	C	7.27659800	-3.06061400	1.00798500
C	-0.71076700	5.13039600	3.36073800	H	5.74918100	4.68299800	-2.73793700	H	2.29276300	-1.71323600	-1.04298000
C	-1.89106900	4.19515100	3.14487300	C	3.89209900	3.92586000	-1.94693500	C	-1.11496100	-3.45316200	0.22046500
C	-2.99595600	4.11520900	4.00059200	H	3.35456100	4.86186100	-2.10553800	C	-5.14276200	-5.24509100	-0.84857000
H	-3.02283400	4.72812100	4.90119100	C	0.77526300	4.33483500	-1.70195800	H	-6.01686900	-2.87018800	-3.76180700
C	-4.06689900	3.26822600	3.71975000	C	0.17763600	4.18413500	-2.96049700	H	-2.87212300	-4.94181500	3.80624900
H	-4.92326000	3.22905500	4.39390200	H	0.08601100	3.18513800	-3.39297300	C	-2.07807400	-2.78591400	-2.10980500
C	-4.03066900	2.45055900	2.59193000	C	-0.31345900	5.29836000	-3.64003600	C	-4.67340700	-5.52194000	2.80809900
H	-4.84168800	1.74984700	2.39114000	H	-0.77964200	5.17033900	-4.61793500	H	-0.81926000	-3.74360300	2.31411100
C	-0.56073900	5.54648200	4.82629800	C	-0.23003600	6.56634200	-3.06150200	H	-8.41922900	-5.09264100	-0.01100800
H	-1.45462200	6.08445400	5.16815700	H	-0.62372200	7.43605600	-3.59043300	C	-4.61397700	-5.59381400	0.42370900
H	0.28446000	6.23686700	4.94683700	C	0.35210400	6.71974300	-1.80053200	C	-3.24497200	-2.83074100	-2.89832000
H	-0.40225400	4.67902100	5.48192100	H	0.41585600	7.70836600	-1.34245100	C	-7.33979700	-5.23843500	0.08177800
C	-0.93612700	6.40326200	2.50006700	C	0.85971900	5.61008600	-1.12388800	C	-5.40090800	-5.66540300	1.56832600
H	-1.05469900	6.14927600	1.43765600	H	1.32126100	5.73426100	-0.14309300	C	-3.24847800	-5.20435800	0.45306900
H	-0.07694100	7.08287800	2.59913200	C	1.00607000	-0.62967800	1.54656800	C	-6.82293500	-5.57760800	1.32882700
H	-1.84334600	6.92786200	2.83369200	C	-0.35211000	-0.79643600	-2.22075700	H	-5.20735500	-5.61939900	3.75655900
C	-4.14517400	0.13470200	0.47602400	C	-1.12170200	-1.75675000	-2.28719900	C	-3.33720500	-5.13456900	2.83640200
C	-4.00727300	-1.13532200	1.05481800	C	2.15948200	-1.04374700	1.66879500	H	-3.34337000	-2.08401800	-3.68922900
H	-3.04107400	-1.43851400	1.46422400	C	7.10707400	-4.75535100	-2.27336900	C	-1.42513200	-4.02586700	1.44972300
C	-5.08877400	-2.01589100	1.07691300	C	5.98468400	-4.08790200	-2.75270300	C	-6.75990600	-4.14896800	-2.21720900
H	-4.97181900	-3.00461700	1.52023900	C	4.27334400	-1.76435900	2.73164700	C	-1.94480200	-3.65456000	-0.94003000
C	-6.30847000	-1.63700500	0.51424500	C	6.18774800	-2.23356200	1.38272000	C	-2.93303100	-4.61782200	-0.80237600
H	-7.14324600	-2.33886700	0.51634800	H	5.52359200	-4.44334200	-3.67742700	C	-5.74263700	-3.55129700	-2.95252300
C	-6.45011700	-0.37365100	-0.06732200	C	7.87159600	-3.96304200	1.88347200	C	-4.10483800	-4.64388900	-1.60453300
H	-7.40054500	-0.08121900	-0.51710700	C	5.42387500	-1.95999800	0.21710100	C	-4.34950800	-3.69642900	-2.59426200
C	-5.37337800	0.51198800	-0.08937900	H	3.77598600	-1.68565700	3.70051100	C	-2.59335800	-4.85463100	1.62991800
H	-5.47804600	1.49035800	-0.56182400	C	8.50439400	-5.26795800	-0.12822600	H	-7.51475900	-5.68500700	2.16790300
C	-2.90047100	2.13197300	-1.14498500	C	6.36955900	-3.02961200	3.62282100	C	-6.48761700	-4.95096600	-1.04734600
C	-3.16845700	3.50628600	-1.17961600	H	7.48547000	-5.60932900	-2.84067900	H	-7.79563000	-3.91801700	-2.47893300
H	-3.21069300	4.07951500	-0.25302100	C	7.19190200	-3.29314400	-0.39098400	H	-0.28413400	-2.74681300	0.16766700

In order to obtain the geometries of the supramolecular adducts with fullerene C_{60}, namely C_{60}@**CAudppe**, C_{60}@**CAudppf**, C_{60}@**CAudppbenz** and C_{60}@**CAuxantphos**, a preoptimization with GFN2 - xTB was carried out by using the most stable geometry of parent hosts as starting structures, to which a molecule of C_{60} was placed within the cavity of both corannulene moieties. In some cases, slight bond rotations had to be made to avoid atom overlapping. Resulting structures were optimized by DFT methods, using PBE0-D3BJ/LANL2DZ//Def2TZVP/PCM(toluene)[9,11] in Gaussian 16 Rev C.01.[12] All geometries were minimized with no restrictions, and the minima were confirmed by vibrational analysis using GFN2-xTB. All the optimized structures are shown below.

C$_{60}$@CAudppe – v$_2$ (G = -5927.650537 a.u.)

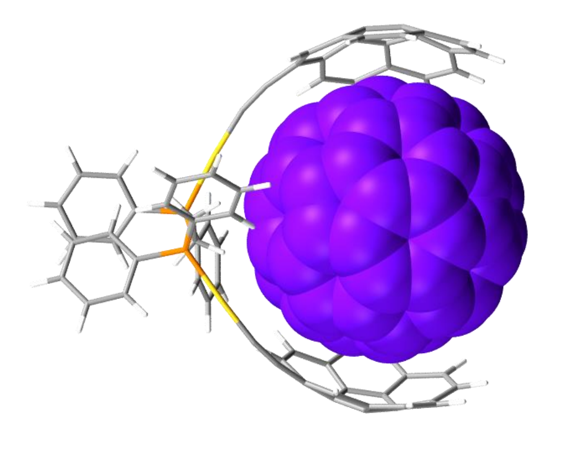

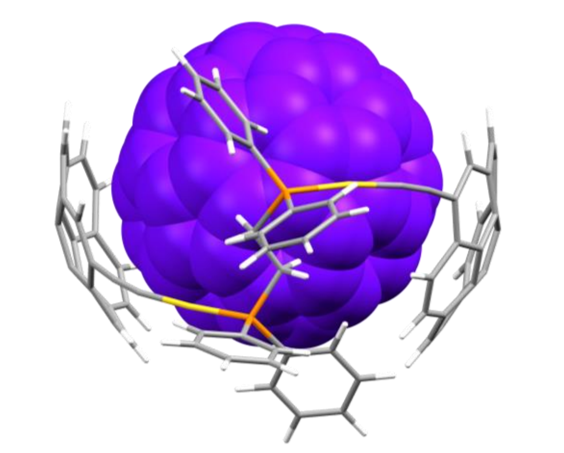

Au	-4.226618	1.531369	-1.79108
Au	-2.076482	-3.477682	1.740396
C	-3.583949	3.363818	-1.388595
C	-0.55445	-4.68958	1.353051
P	-4.842354	-0.660932	-1.969534
P	-3.729114	-1.942979	2.072674
C	-3.995851	-1.626406	-0.669441
C	-3.987467	-0.848772	0.637545
C	-4.403624	-1.524535	-3.495427
C	-5.253761	-2.416517	-4.139641
C	-4.823648	-3.077171	-5.281075
C	-3.547266	-2.856523	-5.776329
C	-2.698532	-1.962211	-5.137937
C	-3.127761	-1.290681	-4.007052
C	-6.605811	-0.943842	-1.670674
C	-7.493759	0.098148	-1.93265
C	-8.852305	-0.067725	-1.715968
C	-9.334444	-1.272023	-1.225283
C	-8.456646	-2.3118	-0.955096
C	-7.099107	-2.151402	-1.178388
C	-5.380529	-2.664066	2.294581
C	-6.463597	-1.886225	2.69912
C	-7.72814	-2.44236	2.780475
C	-7.922496	-3.779001	2.456971
C	-6.849299	-4.559316	2.056624
C	-5.579273	-4.005297	1.97942
C	-3.457609	-0.80007	3.447521
C	-3.937607	0.508703	3.434444
C	-3.699985	1.348725	4.510524
C	-2.973272	0.893374	5.601025
C	-2.481961	-0.403996	5.614614
C	-2.720802	-1.247472	4.542063
H	-2.974514	-1.810333	-1.007656
H	-4.475274	-2.603282	-0.573259
H	-3.191918	-0.100154	0.624416
H	-4.93262	-0.318163	0.785511
H	-6.252378	-2.590426	-3.756521
H	-5.489734	-3.768362	-5.784179
H	-3.214535	-3.377451	-6.666521
H	-1.702105	-1.77998	-5.523072
H	-2.473587	-0.573265	-3.522851
H	-7.110608	1.045068	-2.297497
H	-9.533201	0.749435	-1.922605
H	-10.396107	-1.399186	-1.047693
H	-8.825746	-3.251202	-0.561668
H	-6.431231	-2.975169	-0.958174
H	-6.318348	-0.844449	2.962833
H	-8.565439	-1.832084	3.097829
H	-8.913367	-4.213309	2.52524
H	-6.995102	-5.60527	1.81319
H	-4.732049	-4.613929	1.680647
H	-4.486228	0.889622	2.581317
H	-4.076979	2.364612	4.491229
H	-2.782549	1.554865	6.43817
H	-1.902863	-0.758701	6.458905
H	-2.321294	-2.25605	4.540654
C	0.501488	-5.201024	1.025231
C	-3.018388	4.373818	-1.008198
C	-0.403206	6.133347	2.678875
C	0.831347	6.810278	2.576251
C	-1.096866	6.304945	1.458713
C	0.898876	7.404173	1.297377
C	-0.293183	7.095466	0.608746
C	-0.580122	5.036001	3.491707
C	1.952329	6.428958	3.279918
C	-1.990747	5.372883	0.986498
C	2.091554	7.651213	0.653893
C	-0.353706	7.003881	-0.765246
C	0.511148	4.769856	4.388391
C	-1.668235	4.178195	3.112325
C	1.716012	5.431397	4.287477
C	3.195771	6.874854	2.713945
C	-2.336366	4.336131	1.917497
C	-2.197435	5.38448	-0.451272
C	3.261305	7.455422	1.464918
C	1.982989	7.786659	-0.773345
C	-1.413163	6.189542	-1.274855
C	0.820967	7.480415	-1.447389
H	0.429411	3.951308	5.096319
H	-1.891748	3.305994	3.717262
H	2.536003	5.111208	4.921953
H	4.124172	6.660574	3.233212
H	-3.06402	3.590726	1.615074
H	4.239284	7.674627	1.04896
H	2.865099	8.037889	-1.353342
H	-1.532632	6.068647	-2.345751
H	0.833215	7.498443	-2.532192
C	2.888362	-5.408083	1.610104
H	5.484086	-4.910158	-3.791342
C	5.309257	-5.120959	1.727655
C	6.167598	-5.006744	-1.71745
H	8.178932	-2.616632	2.173933
C	3.979043	-4.219348	3.467459
C	7.343799	-4.182696	-1.681128
H	3.868592	-3.638163	4.377185
C	5.748329	-5.462616	-0.487074
C	7.878601	-3.724197	-0.495498
C	7.290829	-4.042562	0.77679
H	7.770022	-3.822856	-2.61196
C	1.817615	-5.55615	0.641048
C	6.423575	-3.467889	3.007599
C	5.266744	-4.303891	2.835652
C	3.442704	-5.860381	-1.200918
C	2.104745	-5.793363	-0.702664
H	1.879734	-4.549933	3.334278
H	3.220562	-5.61933	-3.362146
C	4.150996	-5.669325	1.132094
H	8.706071	-3.023562	-0.539453
C	6.294637	-4.99228	0.726105
C	7.385834	-3.342856	2.028507
C	2.84568	-4.740459	2.879924
C	4.425894	-5.88374	-0.235432
C	3.920992	-5.598929	-2.533638
C	5.214963	-5.192368	-2.778587
H	6.495808	-2.834743	3.885973
H	1.271627	-5.778867	-1.396256
C	1.144354	-2.191076	0.164976
C	0.073117	-1.257935	0.425857
C	-0.468793	-0.837698	-0.847695
C	0.265118	-1.51753	-1.893097
C	1.261201	-2.354855	-1.264927
C	-0.375168	2.693848	-0.505978
C	-0.260307	2.538161	-1.936136
C	-0.568902	1.165605	-2.261177
C	-0.877891	0.474649	-1.027835
C	-0.761295	1.423192	0.057682
C	1.174303	1.210323	-3.985138
C	2.24318	0.272465	-4.241522
C	1.863044	-1.004677	-3.681021
C	0.559072	-0.855972	-3.075461
C	0.133899	0.51405	-3.262482
C	4.195363	-1.371664	-3.012752
C	4.736689	-1.791306	-1.740344
C	3.697129	-2.487213	-1.020171
C	2.511135	-2.496517	-1.845231
C	2.818299	-1.808097	-3.077406
C	4.503571	-1.475763	1.060541
C	3.771054	-0.798778	2.106346
C	2.394345	-1.234556	2.040778
C	2.27875	-2.178733	0.95392
C	3.581985	-2.33053	0.350416
C	1.676607	1.036601	2.616745
C	0.681797	1.875045	1.98888
C	-0.242361	1.0194	1.27852
C	0.182099	-0.349255	1.467231
C	1.368221	-0.339054	2.295623
C	2.423475	3.533948	1.511137
C	2.731042	4.224442	0.279417
C	1.546745	4.211725	-0.546346
C	0.507703	3.513367	0.173963
C	1.049907	3.094187	1.444672
C	1.658541	4.062543	-1.918032
C	2.961347	3.914455	-2.523173
C	2.844779	2.96507	-3.607105
C	1.468786	2.52825	-3.672281
C	0.734626	3.207426	-2.628522
C	3.869528	2.06547	-3.854558
C	5.055378	2.076272	-3.02849
C	5.48173	0.708438	-2.841674
C	4.559258	-0.14779	-3.551925
C	3.563017	0.691332	-4.178395
C	6.000624	0.305224	-1.621371
C	6.117813	1.253963	-0.536924
C	5.81053	0.563789	0.694927
C	5.503088	-0.810733	0.370902
C	5.620994	-0.970448	-1.059619
C	5.108087	1.215099	1.697057
C	4.68206	2.582924	1.508909
C	3.377596	2.731358	2.114541
C	2.99756	1.456631	2.678974
C	4.066316	0.518796	2.418923
C	4.977015	3.245373	0.327339
C	5.709381	2.566125	-0.716812
C	5.166736	2.985859	-1.988965
C	4.099142	3.925253	-1.731078
C	3.981382	4.084925	-0.2999

C_{60}@CAudppf – v_2 (G = -7498.142565 a.u.)

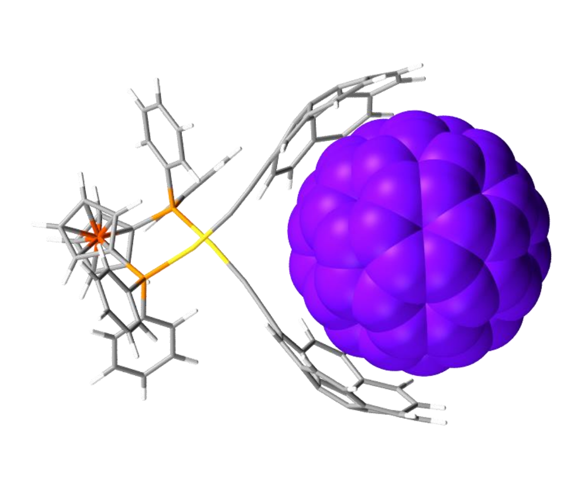

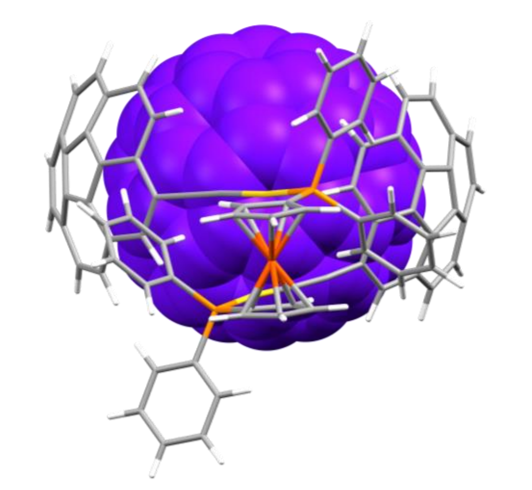

Au	-3.259691	-0.200576	1.370511	C	-6.751481	-0.629924	-1.584594	C	1.596759	-6.145885	0.273312
Fe	-7.615613	-0.102351	0.194345	H	-5.823003	-1.167273	-1.693989	C	2.902153	-6.372358	-1.784727
P	-4.95036	-1.702391	1.689792	C	-8.042442	-1.1959	-1.487625	H	0.011668	-2.907391	-3.911345
C	-1.894287	1.243355	1.277935	H	-8.266476	-2.251493	-1.49273	C	6.059173	-0.549459	3.017518
C	-1.161662	2.211128	1.179652	C	-8.97515	-0.136442	-1.33148	C	5.218277	-1.702937	2.792469
C	-5.117361	-3.08518	0.537854	H	-10.040897	-0.243224	-1.197872	C	5.959688	-2.644634	1.984435
C	-4.362678	-3.085513	-0.62892	C	-8.260681	1.086632	-1.324897	C	7.25859	-2.072468	1.710244
H	-3.67662	-2.272164	-0.838868	H	-8.693167	2.06631	-1.202992	C	7.319924	-0.777805	2.348858
C	-4.472001	-4.13529	-1.528966	C	2.5902	6.216385	0.836568	C	3.63438	-3.304245	-0.619158
H	-3.866403	-4.126805	-2.427608	C	1.362105	5.525656	0.797722	C	4.895166	-3.533279	-1.287344
C	-5.335003	-5.186581	-1.264892	C	3.138891	6.05769	2.127257	C	5.930725	-3.582315	-0.281563
H	-5.416552	-6.011029	-1.963881	C	1.15183	4.937115	2.063007	C	5.309022	-3.382738	1.008478
C	-6.080843	-5.197691	-0.091567	C	2.248588	5.269384	2.886695	C	3.890245	-3.210551	0.79788
H	-6.740241	-6.030212	0.125193	C	3.36415	6.428356	-0.282294	C	7.440422	-2.417763	-1.822382
C	-5.967656	-4.15609	0.812744	C	0.838459	4.997511	-0.361713	C	8.279883	-1.263711	-1.596107
H	-6.51821	-4.189204	1.746381	C	4.49203	6.104017	2.374145	C	8.535963	-1.169181	-0.177095
C	-4.813043	-2.520138	3.302631	C	0.411467	3.794641	2.246308	C	7.854749	-2.264457	0.473947
C	-3.828792	-3.495647	3.45963	C	2.659469	4.479846	3.937134	C	7.177364	-3.036087	-0.543166
H	-3.200658	-3.774859	2.620048	C	2.723081	6.081886	-1.522897	C	8.400535	1.273096	-0.34542
C	-3.649921	-4.108398	4.687063	C	4.750615	6.688601	-0.006034	C	7.660395	2.215635	0.462023
H	-2.884293	-4.867175	4.800391	C	1.523575	5.403431	-1.561373	C	7.397109	1.596912	1.741818
C	-4.446861	-3.750605	5.767219	C	-0.102481	3.936128	-0.168956	C	7.974403	0.272501	1.724596
H	-4.305226	-4.231206	6.728253	C	5.286866	6.534873	1.256131	C	8.59485	0.072587	0.435117
C	-5.422024	-2.777648	5.615027	C	4.895726	5.458569	3.593955	C	5.188617	2.660248	1.766733
H	-6.04449	-2.494784	6.455993	C	-0.319943	3.344031	1.074802	C	3.890473	2.090331	2.040323
C	-5.606658	-2.16077	4.385473	C	0.687946	3.081629	3.460882	C	4.083593	0.892323	2.824377
H	-6.369273	-1.399513	4.270313	C	4.025178	4.688357	4.336934	C	5.503769	0.720484	3.033136
C	-6.520837	-0.840514	1.746307	C	1.757497	3.409606	4.267825	C	6.186489	1.815029	2.380145
C	-6.628623	0.580638	1.851672	H	3.241225	6.263897	-2.458775	C	2.239201	-0.167144	1.601054
H	-5.798224	1.26948	1.838943	H	5.423729	6.914435	-0.826779	C	2.181775	-1.460759	0.960561
C	-7.998716	0.916756	1.933901	H	1.146521	5.07665	-2.524809	C	3.179372	-2.307322	1.571008
H	-8.396795	1.918425	1.983496	H	-0.590944	3.481731	-1.024145	C	3.856863	-1.537619	2.589307
C	-8.751024	-0.287165	1.883721	H	6.359418	6.646724	1.37656	C	3.276214	-0.213251	2.608033
H	-9.827686	-0.36373	1.889084	H	5.938273	5.490154	3.893242	C	1.737277	-0.351943	-1.180789
C	-7.84704	-1.371486	1.765155	H	0.103405	2.19762	3.691235	C	2.359451	-0.550805	-2.470623
H	-8.113422	-2.412289	1.672852	H	4.41885	4.14523	5.190004	C	2.943292	-1.871787	-2.484279
Au	-3.577936	0.63705	-1.810832	H	1.978876	2.769032	5.115219	C	2.681152	-2.489088	-1.203612
P	-5.474208	1.904265	-1.674677	C	0.451004	-4.648113	1.83476	C	1.937302	-1.550483	-0.399472
C	-2.155323	-0.738626	-2.023913	H	2.128382	-4.585103	-4.608347	C	4.148906	-2.095353	-3.127357
C	-1.452398	-1.734015	-2.049001	H	-0.541777	-2.929477	2.756122	C	4.830762	-1.000427	-3.777566
C	-5.580258	3.171601	-0.389982	C	1.444839	-5.054984	2.790935	C	6.25036	-1.170824	-3.566995
C	-4.521497	3.307088	0.500582	H	4.931923	-7.067498	-1.37373	C	6.445798	-2.371966	-2.786805
H	-3.643802	2.677199	0.409969	C	-0.664821	-3.563016	-0.607124	C	5.147225	-2.944108	-2.514926
C	-4.583273	4.24723	1.519706	C	-0.721532	-2.939907	-1.920605	C	7.056706	-0.064987	-3.350671
H	-3.749143	4.337975	2.205599	C	-0.92651	-2.972066	0.672106	C	6.478145	1.258986	-3.334735
C	-5.700104	5.056477	1.648751	H	4.738046	-7.069729	1.028177	C	7.156832	2.030025	-2.318815
H	-5.75013	5.790887	2.444457	C	2.95581	-5.855613	-3.125574	C	8.154481	1.182409	-1.706174
C	-6.751934	4.940224	0.74624	C	-0.027806	-4.779348	-0.564749	C	8.092637	-0.112371	-2.344228
H	-7.61694	5.587645	0.833644	H	3.840098	-6.032285	-3.728995	C	6.445548	2.933433	-1.544998
C	-6.691177	4.007563	-0.274024	C	2.498529	-5.87463	2.445532	C	5.025438	3.104271	-1.754271
H	-7.498341	3.946843	-0.99448	H	1.432943	-4.623407	3.786568	C	4.405796	3.299653	-0.46391
C	-5.856294	2.799017	-3.206808	C	0.006162	-3.469481	-2.984129	C	5.44076	3.252642	0.541605
C	-5.339282	4.076161	-3.409196	C	-0.399778	-3.489716	1.837233	C	6.701977	3.026337	-0.125029
H	-4.762381	4.557475	-2.627418	C	1.975485	-5.02786	-3.629811	C	3.163728	2.749099	-0.203398
C	-5.565258	4.736296	-4.605897	C	0.837091	-4.627718	-2.845501	C	2.482649	1.9805	-1.219679
H	-5.16208	5.731257	-4.754222	H	-1.449734	-2.021574	0.712652	C	1.796621	0.887563	-0.568739
C	-6.304261	4.12638	-5.609386	C	3.971202	-6.819537	-0.934524	C	2.056771	0.980866	0.849711
H	-6.480932	4.644607	-6.544645	C	2.667668	-6.37457	1.108222	C	2.900288	2.131647	1.074337
C	-6.815506	2.851941	-5.413708	H	3.273113	-6.056623	3.183387	C	3.072997	1.795134	-2.458903
H	-7.391765	2.370765	-6.195366	C	1.709965	-6.144871	-1.134023	C	4.374029	2.365849	-2.729709
C	-6.592318	2.187837	-4.217909	C	0.519955	-5.305166	0.625814	C	5.116184	1.42259	-3.534796
H	-6.993999	1.192195	-4.069469	C	3.860795	-6.819664	0.440579	C	4.274832	0.269596	-3.761732
C	-6.871589	0.790134	-1.482188	C	0.704432	-5.302671	-1.651738	C	3.012456	0.499251	-3.096939

C_{60}@CAudppbenz – v_1 (G = -6079.940677 a.u.)

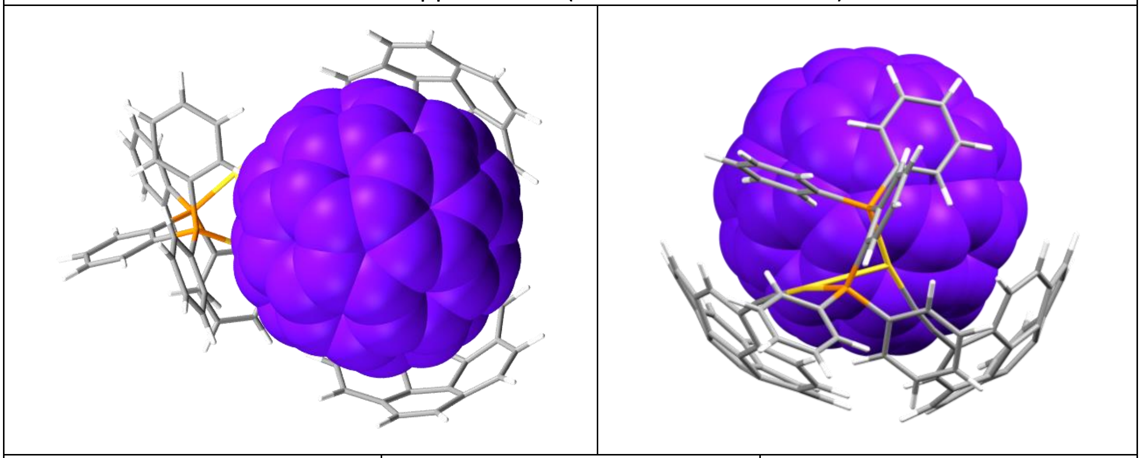

Au	4.547791	-1.468811	-0.706431	C	1.126865	2.381365	-2.319185	C	-6.37069	0.318363	3.736504
Au	3.012926	1.137242	-0.049632	C	2.066782	-3.390267	-1.340751	C	-5.723356	-0.795674	4.39217
C	3.052963	-2.728326	-1.067388	C	-2.804073	-5.521603	-2.801009	C	-4.543094	-0.296318	5.06362
C	1.933806	1.869725	-1.562093	C	-3.149189	-4.536895	-3.752372	C	-4.458742	1.125939	4.820478
P	6.227433	0.088129	-0.603628	C	-1.431945	-5.382972	-2.509442	C	-5.504471	-3.136345	1.725041
P	4.054202	0.634916	1.914219	C	-1.988055	-3.790424	-4.047483	C	-4.374411	-3.517847	2.546284
C	5.861868	1.490858	-1.692715	C	-0.927004	-4.311359	-3.276163	C	-4.495324	-2.83821	3.818015
C	5.213733	1.222991	-2.8991	C	-3.708998	-6.050274	-1.909239	C	-5.70026	-2.039672	3.783139
H	4.942593	0.203033	-3.149583	C	-4.418568	-4.019665	-3.862569	C	-6.323541	-2.223966	2.490632
C	4.868089	2.259107	-3.746609	C	-0.880294	-5.754077	-1.303303	C	-2.060083	-2.537274	3.860025
H	4.337878	2.046111	-4.667124	C	-2.027761	-2.481835	-4.470719	C	-1.277468	-1.349022	4.124424
C	5.162408	3.570631	-3.398988	C	0.155093	-3.555435	-2.893108	C	-2.097281	-0.436967	4.891176
H	4.868719	4.383036	-4.053885	C	-5.076041	-5.674923	-2.156552	C	-3.387411	-1.061183	5.098012
C	5.812625	3.84154	-2.205675	C	-3.113437	-6.642748	-0.740762	C	-3.363432	-2.358877	4.460601
H	6.03604	4.865032	-1.926897	C	-5.413548	-4.710299	-3.085372	C	-1.112174	1.436428	3.648182
C	6.165851	2.805374	-1.353239	C	-4.481383	-2.729196	-4.501171	C	-1.760323	2.551004	2.991946
H	6.654296	3.029091	-0.412538	C	-1.770717	-6.502183	-0.452791	C	-3.064781	2.727189	3.59103
C	7.78799	-0.616447	-1.202802	C	0.369525	-5.125543	-0.996259	C	-3.223411	1.725084	4.62135
C	8.600372	0.038835	-2.119969	C	-3.346996	-2.000000	-4.788907	C	-2.016565	0.927094	4.656388
H	8.301538	1.002806	-2.516384	C	-0.806556	-1.750367	-4.25853	C	-3.976103	3.290021	1.380349
C	9.789193	-0.546781	-2.535917	C	0.89064	-4.070717	-1.747734	C	-5.157887	2.792951	0.707906
H	10.415301	-0.034882	-3.257958	C	0.233102	-2.258064	-3.505408	C	-6.057954	2.281382	1.71832
C	10.17012	-1.780778	-2.033171	H	-5.862948	-6.083145	-1.530127	C	-5.434775	2.465695	3.012172
H	11.096801	-2.237561	-2.361924	H	-3.74709	-7.132373	-0.007661	C	-4.147705	3.091122	2.803819
C	9.355576	-2.440674	-1.120156	H	-6.451414	-4.399518	-3.15034	C	-6.688116	0.467941	0.19511
H	9.642738	-3.413357	-0.737478	H	-5.447726	-2.265784	-4.67266	C	-6.772875	-0.953797	0.44007
C	8.164244	-1.866482	-0.712775	H	-1.4066	-6.885981	0.495519	C	-6.945516	-1.156968	1.861977
H	7.511645	-2.393213	-0.023405	H	0.89957	-5.391444	-0.088049	C	-6.968286	0.141641	2.497601
C	6.710162	0.888432	0.975605	H	-3.470939	-0.991994	-5.170494	C	-6.810574	1.145087	1.467444
C	8.03831	1.27089	1.131551	H	-0.734868	-0.725536	-4.606315	C	-5.085435	-1.317943	-1.305922
H	8.75687	1.034272	0.355616	H	1.082248	-1.621408	-3.278416	C	-3.87922	-2.11468	-1.264744
C	8.460444	1.957013	2.261909	C	-0.643051	2.256383	-3.991248	C	-4.034076	-3.115813	-0.237221
H	9.501021	2.245179	2.357757	H	-5.001068	1.447103	-5.415739	C	-5.337399	-2.939217	0.363004
C	7.552789	2.269505	3.257507	C	-4.935131	5.995302	-1.019822	C	-5.98669	-1.826759	-0.294467
H	7.871722	2.811585	4.140691	H	-2.660109	0.875476	-5.527182	C	-2.951275	-3.483215	0.545716
C	6.228045	1.876819	3.127607	H	-0.962658	5.978781	0.441007	C	-1.662456	-2.864181	0.334384
H	5.518491	2.112144	3.912108	C	-3.900272	5.198006	-2.99779	C	-1.040067	-2.677643	1.629537
C	5.793094	1.182478	2.004423	C	-1.186546	5.621158	-0.559132	C	-1.94355	-3.186734	2.639793
C	4.130687	-1.081488	2.488937	C	0.021318	2.965876	-2.987754	C	-3.125422	-3.685839	1.968791
C	5.280573	-1.641163	3.042653	C	-6.17938	4.574082	-2.796649	C	-0.296075	-1.536735	1.879748
H	6.191597	-1.060775	3.129271	C	-1.688336	4.633367	-3.080073	C	-0.131637	-0.53424	0.847019
C	5.264047	-2.958073	3.483944	C	-2.365361	3.885844	-4.064582	C	-0.159049	0.762417	1.486104
H	6.164423	-3.392456	3.907935	C	-1.923686	2.649388	-4.485532	C	-0.329962	0.562123	2.91058
C	4.10539	-3.714563	3.386609	C	-5.07375	5.340858	-2.291206	C	-0.417699	-0.859031	3.15247
H	4.100808	-4.744996	3.726411	H	-3.657153	6.660976	0.539285	C	-0.784226	1.825298	0.859034
C	2.955883	-3.155419	2.840527	C	-0.271445	4.801532	-1.184305	C	-1.405609	1.642734	-0.432131
H	2.046817	-3.737524	2.740727	C	-3.699066	6.262251	-0.469226	C	-2.60646	2.442954	-0.471426
C	2.972866	-1.84902	2.386012	C	-2.939108	1.830188	-5.094717	C	-2.730164	3.114474	0.804861
H	2.078559	-1.429776	1.941705	H	-0.219676	1.300175	-4.273649	C	-1.602876	2.73381	1.626438
C	3.186286	1.508504	3.247994	C	-6.016933	3.625649	-3.785256	C	-3.738012	1.969776	-1.120429
C	2.616613	2.752523	2.974905	C	-2.479262	5.889971	-1.130974	C	-3.715042	0.672681	-1.760382
H	2.709263	3.17871	1.980979	C	-0.550163	4.183007	-2.450275	C	-5.005039	0.045355	-1.545236
C	1.900932	3.420634	3.953995	C	-3.734114	4.226039	-4.009246	C	-5.824133	0.957389	-0.775391
H	1.447705	4.3795	3.727654	C	-4.733249	3.35065	-4.371046	C	-5.041482	2.145148	-0.512754
C	1.734195	2.847771	5.208545	H	-7.151246	4.66506	-2.322621	C	-2.561389	-0.094891	-1.715377
H	1.151646	3.361156	5.966027	C	-4.275651	2.158203	-5.032452	C	-1.379315	0.402013	-1.044832
C	2.294934	1.609153	5.483819	H	-5.820366	6.195168	-0.424632	C	-0.735564	-0.710976	-0.387142
H	2.157275	1.152556	6.457554	H	0.640983	4.528527	-0.665525	C	-1.519265	-1.897307	-0.647898
C	3.022777	0.940539	4.508333	C	-2.634877	5.457924	-2.428471	C	-2.647746	-1.516366	-1.464682
H	3.442025	-0.036473	4.722148	C	-5.587924	1.505367	4.000183	H	-6.868189	3.008366	-4.054334

C_{60}@CAuxantphos - v_2 (G = -6502.514369 a.u.)

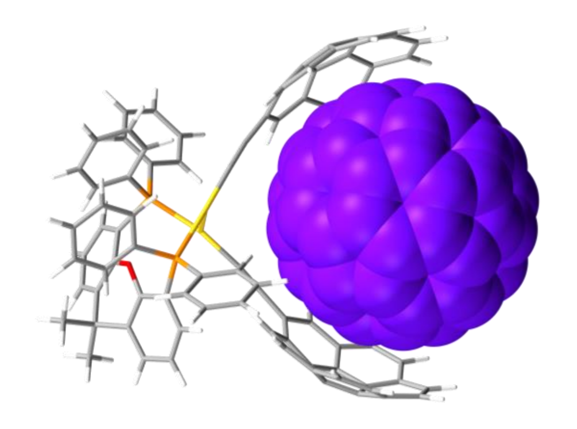

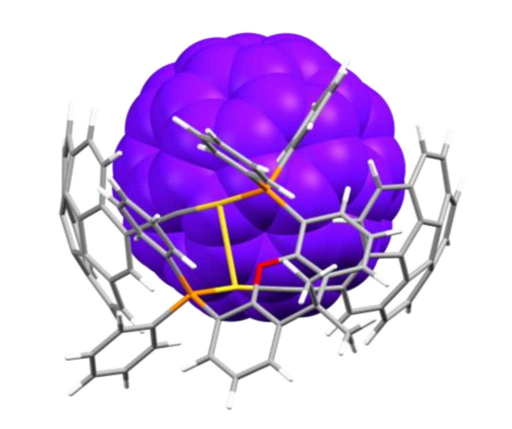

Au	3.324204	-0.374086	-1.578160	C	5.32015	-0.53414	3.88020	C	-1.137175	-4.974595	-2.074953
Au	2.790141	-0.707033	1.356404	H	4.39153	-1.09474	3.85912	C	-2.935863	-6.055648	2.127471
P	5.082179	-1.832493	-1.266494	C	6.34639	-0.91561	4.72701	H	-6.869438	-6.375795	-0.919039
P	4.039512	1.075631	2.056814	H	6.21334	-1.77099	5.37863	C	-3.846894	-5.929070	-2.477480
O	6.063155	0.723950	-0.201475	C	7.54609	-0.21897	4.72565	H	-3.671402	-4.879509	-4.386460
C	6.479769	-0.864802	-1.899711	H	8.35238	-0.52449	5.38260	H	-0.097198	-4.223464	2.731008
C	6.715501	0.404358	-1.366261	C	7.71475	0.86522	3.87724	C	-5.280732	3.663806	-0.451786
C	5.722102	2.033764	-0.006413	H	8.65151	1.41073	3.86969	C	-6.469189	3.285023	0.276757
C	4.705929	2.322488	0.910247	C	6.68100	1.26377	3.04340	C	-6.124853	3.197501	1.678436
C	4.277296	3.643427	1.006861	H	6.81069	2.12383	2.39733	C	-4.723464	3.523588	1.814912
H	3.481584	3.896059	1.693757	C	2.09020	1.09500	-2.07348	C	-4.201882	3.810404	0.497970
C	4.852204	4.637737	0.237025	C	1.69960	-2.29268	0.84706	C	-7.378832	-0.025202	2.498962
H	4.474110	5.650554	0.295544	C	1.05694	-3.22688	0.40215	C	-6.299942	0.121302	3.448922
C	5.939444	4.340538	-0.565158	C	1.41726	2.07169	-2.35105	C	-5.872895	1.501557	3.430760
H	6.430542	5.137532	-1.107816	C	-2.72910	6.94253	1.03048	C	-6.687425	2.208301	2.469226
C	6.416879	3.040669	-0.670772	C	-1.55831	6.38948	1.50462	C	-7.618387	1.264575	1.893292
C	7.727357	2.678360	-1.347360	C	-0.21441	3.33505	-3.66290	C	-3.554061	0.756570	3.716819
C	7.553433	1.318581	-1.988179	C	-1.07414	5.39657	-2.86681	C	-2.364846	1.134740	2.988304
C	8.209147	0.916450	-3.146365	H	-1.43920	6.27517	2.57730	C	-2.604724	2.426815	2.382651
H	8.865377	1.609249	-3.656711	C	-3.39876	6.12237	-3.11332	C	-3.941983	2.845921	2.737977
C	8.040991	-0.357719	-3.659756	C	-0.22462	5.30748	-1.74423	C	-4.528428	1.812858	3.561182
H	8.569428	-0.654876	-4.557327	H	-0.20816	2.51420	-4.37126	C	-1.345126	1.696628	0.407481
C	7.170184	-1.241229	-3.045290	C	-4.29851	7.09632	-1.03703	C	-1.692168	1.783695	-0.991103
H	6.992516	-2.218778	-3.475531	C	-2.38869	4.41362	-4.57264	C	-2.664374	2.840170	-1.146443
C	8.169866	3.733511	-2.350742	H	-3.48572	7.24195	1.74850	C	-2.922312	3.405684	0.157603
H	9.131377	3.466551	-2.790513	C	-1.97522	6.66767	-1.19746	C	-2.108699	2.697800	1.118411
H	8.313047	4.695495	-1.857118	C	0.56190	4.21016	-1.48788	C	-3.801286	1.501060	-2.853337
H	7.440988	3.857371	-3.153931	H	-5.48084	6.67060	-2.74609	C	-5.203071	1.175867	-2.990233
C	8.799428	2.556072	-0.246804	C	-4.47508	6.67525	-2.33871	C	-5.964582	2.177654	-2.278201
H	8.527091	1.792035	0.484206	C	-0.56378	5.84072	0.62314	C	-5.033287	3.120158	-1.701919
H	8.914320	3.508775	0.275978	C	0.62306	3.22536	-2.55318	C	-3.697876	2.701249	-2.056462
H	9.759814	2.278252	-0.687653	C	-0.77885	6.09808	-0.71276	C	-7.532697	0.434794	-1.561031
C	4.987458	-3.301514	-2.320287	C	-3.02588	7.00783	-0.37488	C	-8.053734	0.146655	-0.244664
C	3.998337	-3.363509	-3.297688	H	-2.52604	3.69139	-5.37068	C	-7.948406	1.348649	0.550327
H	3.303596	-2.536720	-3.402984	C	-3.44514	5.23697	-4.24552	C	-7.362441	2.380192	-0.275043
C	3.897650	-4.477253	-4.116974	C	0.96226	4.05920	-0.11937	C	-7.105616	1.815285	-1.579833
H	3.119683	-4.523094	-4.869821	H	0.69746	4.60749	1.91286	C	-7.064291	-2.093659	-0.375020
C	4.781611	-5.533936	-3.961683	C	0.43461	4.84143	0.88647	C	-6.249090	-2.800280	0.587257
H	4.696805	-6.408652	-4.596099	H	-5.17190	7.40685	-0.47283	C	-6.506511	-2.235968	1.892584
C	5.767891	-5.478893	-2.985516	C	-1.17404	4.38711	-3.80023	C	-7.478954	-1.178366	1.736830
H	6.453685	-6.308347	-2.858126	H	-4.37119	5.13162	-4.80150	C	-7.823648	-1.090683	0.335869
C	5.870055	-4.368991	-2.164149	C	-2.15696	6.23410	-2.52863	C	-5.470798	-2.094792	2.803521
H	6.628953	-4.334098	-1.390765	H	1.61589	3.23317	0.13604	C	-4.134223	-2.512673	2.449292
C	5.638818	-2.510822	0.319431	C	-0.78808	-4.85683	2.18533	C	-3.202555	-1.569998	3.024998
C	6.890852	-2.211105	0.846173	C	-3.06748	-6.32427	-1.41275	C	-3.963963	-0.567628	3.734951
H	7.541468	-1.509525	0.339303	H	-1.42145	-4.08474	-4.05071	C	-5.365937	-0.891598	3.597932
C	7.317195	-2.821999	2.015081	H	-4.64582	-6.01870	3.48614	C	-2.063095	-1.205794	2.323930
H	8.291287	-2.578552	2.422216	C	0.18578	-4.20812	-0.12959	C	-1.809725	-1.765316	1.017295
C	6.497547	-3.733434	2.662055	C	-5.28627	-6.46048	1.51292	C	-1.216396	-0.740202	0.192990
H	6.834101	-4.212167	3.574672	H	-2.04705	-5.09254	3.87624	C	-1.110143	0.461107	0.987656
C	5.243872	-4.027607	2.144788	H	-5.94468	-5.82684	-3.07647	C	-1.634469	0.174066	2.306049
H	4.594364	-4.733575	2.648893	C	-3.60905	-6.64507	-0.14912	C	-1.545451	-0.655888	-1.149297
C	4.812288	-3.420600	0.977623	C	-0.07637	-4.20909	-1.49915	C	-2.481153	-1.596917	-1.723115
H	3.830069	-3.650515	0.581954	C	-5.26154	-6.07175	-2.26970	C	-3.295128	-0.892377	-2.686236
C	3.059558	2.058771	3.228671	C	-4.95866	-6.58709	0.11904	C	-2.866703	0.488490	-2.704278
C	1.671726	2.044895	3.118182	C	-2.62341	-6.38582	0.82772	C	-1.787636	0.632999	-1.753029
H	1.198000	1.440679	2.352204	C	-5.79065	-6.38484	-1.03552	C	-4.639808	-1.205099	-2.819652
C	0.895754	2.793094	3.991143	H	-6.32791	-6.45933	1.81701	C	-5.226713	-2.235784	-1.994473
H	-0.183342	2.772236	3.898722	C	-4.32415	-6.20960	2.46751	C	-6.563438	-1.816902	-1.637777
C	1.501635	3.554044	4.978812	H	0.45746	-3.48363	-2.10331	C	-6.802303	-0.526055	-2.242280
H	0.894384	4.133219	5.664930	C	-1.90127	-5.34846	2.83186	C	-5.613452	-0.147895	-2.972679
C	2.885495	3.566068	5.097135	C	-3.14434	-5.18651	-3.48893	C	-4.445019	-2.908678	-1.069762
H	3.360104	4.154202	5.873943	C	-0.59139	-5.03443	0.77437	C	-3.046175	-2.582492	-0.932524
C	3.663135	2.821880	4.226350	C	-1.47352	-5.89665	0.16667	C	-2.701844	-2.668708	0.467371
H	4.742400	2.826522	4.326263	C	-1.85689	-4.73436	-3.29850	C	-3.888418	-3.048253	1.195984
C	5.477504	0.565812	3.040767	C	-1.74753	-5.86513	-1.21791	C	-4.966825	-3.197349	0.246704

Table S8. Intramolecular Au(I)-Au(I) distances in computed C_{60} adducts of reported hosts in this work. Empirical aurophilic interaction energy based on Equation 9.[13]

Assembly	d(Au-Au) / Å	E_{Au-Au} / kcal·mol^{-1}
C_{60}@**CAudppe**	6.50	0
C_{60}@**CAudppf**	3.31	-2.9
C_{60}@**CAudppbenz**	3.00	-6.0
C_{60}@**CAuxantphos**	3.10	-8.3

$$E_{Au-Au} = 1.27 \times 10^{6}\, e^{-3.5d(Au-Au)} \quad \text{eq. 9}$$

Non-covalent interactions were obtained according to Yang and collaborators' scheme with the help of the NCIPlot package[14] by locating critical points defined as regions of decreased reduced density gradient where electronic density values are low. Calculations were carried out with promolecular densities, gradient isosurfaces were plotted with an isovalue of 0.3 a.u. and colored on a RGB scale according to values of the sign of λ_2 (second eigenvalue of the electron-density Hessian). Red indicates repulsion, green means weak attraction, and blue represents strong attraction. Additionally, a topology analysis was performed using AIM theory[15] using Multiwfn 3.8 software package,[16] to search critical points (CPs) between the gold atoms.

The electronic interaction energy in each adduct (Table S9) was determined by the Counterpoise method[17] according to Equation 10.

$$E_{int} = E_{HG}^{HG}(HG) - \left(E_{H}^{HG}(HG) + E_{G}^{HG}(HG)\right) \quad \text{eq. 10}$$

Where H and G stand for host and guest, respectively; superscript refers to the basis set used (from the adduct in all cases, whereas subscript denotes each fragment. The optimized geometry whose fragments are used is given in parenthesis (the adduct in all cases).

Electronic deformation energy, understood as the difference in energy between the geometry of a chemical species in the supramolecular adduct and the optimized geometry of that same species, is defined by Equation 11.

$$E_{def} = E_{H}(HG) - E_{H}(H) \quad \text{eq. 11}$$

Here, subscripts and parenthesis have the same meaning as those described for Equation 5. The electronic deformation energy of the guest was assumed to be near zero in all cases.

Table S9. Summary of interaction energies of the different adducts computed by Counterpoise method.

Adduct	E_{int} / kcal·mol^{-1} (Counterpoise)	E_{def} / kcal·mol^{-1}
C_{60}@**CAudppe**	-45.4	10.4
C_{60}@**CAudppf**	-33.9	7.2
C_{60}@**CAudppbenz**	-42.1	10.0
C_{60}@**CAuxantphos**	-38.3	14.0

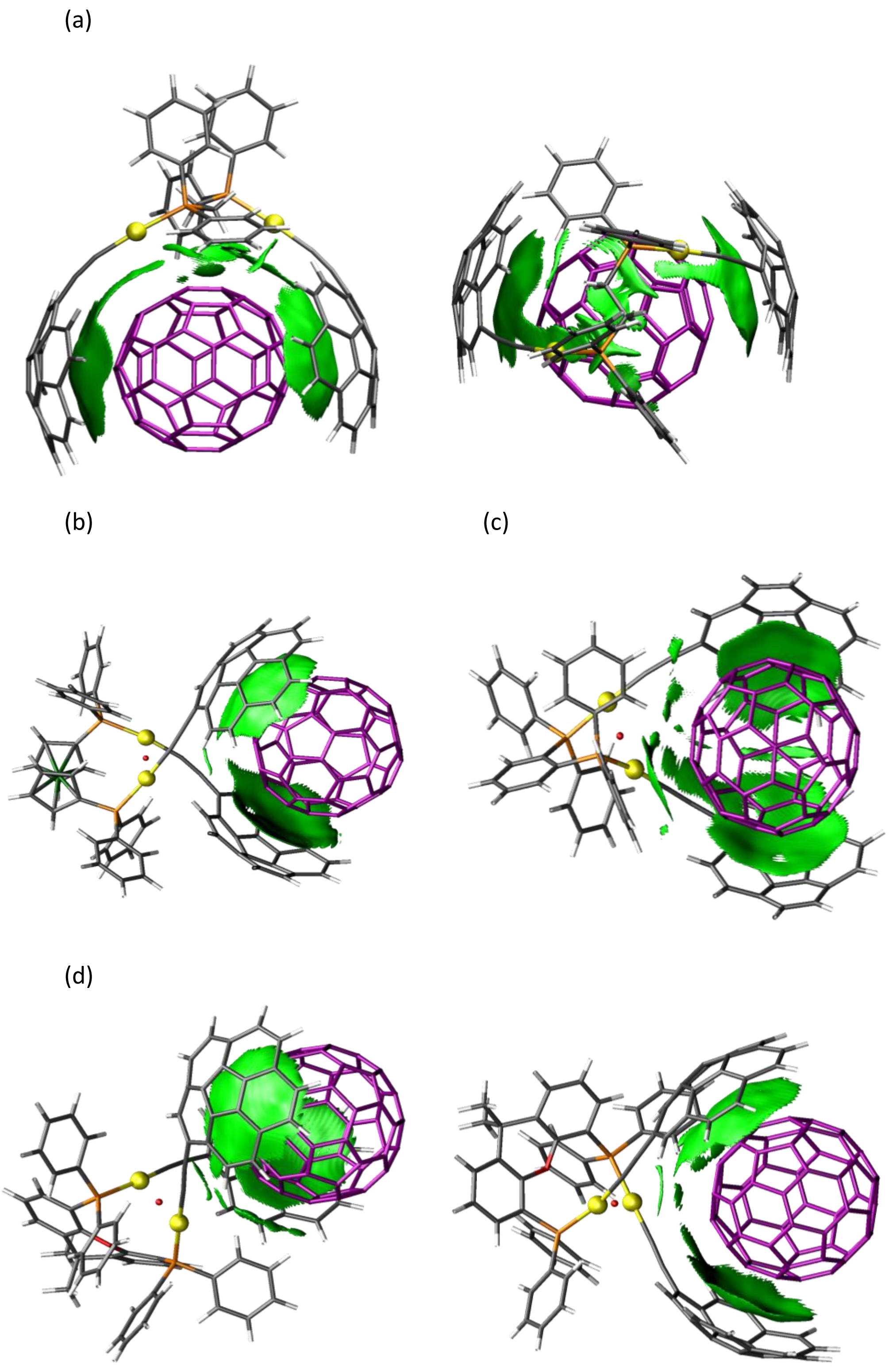

Figure S 146. NCI plots (isovalue = 0.3 a.u.) of the supramolecular adducts formed between (a) C_{60}@**CAudppe**, (b) C_{60}@**CAudppf**, (c) C_{60}@**CAudppbenz** and (d) C_{60}@**CAuxantphos**. Red dots represent critical points between gold(I) atoms.

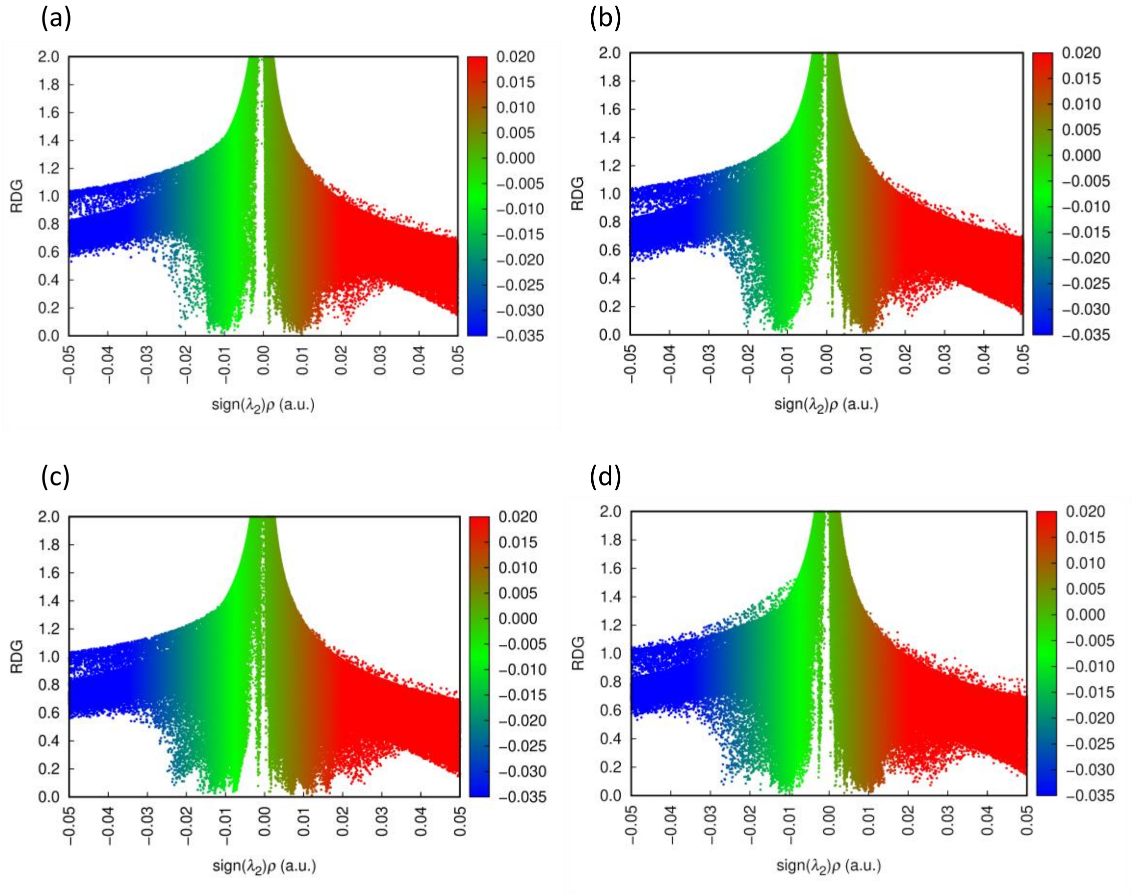

Figure S 147. Plot of the reduced density gradient versus the electron density multiplied by the sign of the second Hessian eigenvalue (λ_2) of supramolecular adducts (a) C_{60}@**CAudppe**, (b) C_{60}@**CAudppf**, (c) C_{60}@**CAudppbenz** and (d) C_{60}@**CAuxantphos**.

As discussed in the main text, the optimized geometry of C_{60}@**CAudppbenz** substantially differs from the ones observed for all the other examples within the family as a bent arrangement between corannulene units is obtained, instead of a tweezer-like arrangement. Given that the theoretical method usually maximizes intermolecular contacts, this structure results as the most stable one, therefore providing such a relatively high interaction energy by the Counterpoise method and more extended areas in the NCI plot. A second structure was thus obtained by reoptimizing it using the same level of theory as described above. This provides a lower interaction energy (-29.8 kcal/mol) and higher deformation energy (27.5 kcal/mol). Refer to Table S9 for comparison. The coordinates are shown below.

C_{60}@CAuxantphos – v_3

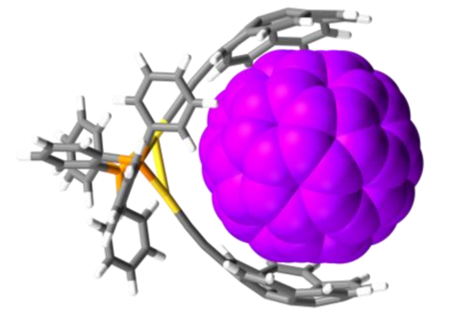

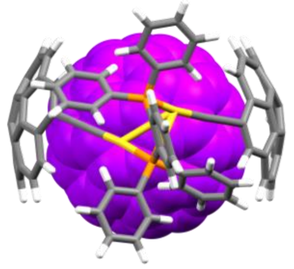

Au	3.357107	4.695426	6.003563	C	5.711923	3.059610	7.333391	C	3.711363	2.028806	16.415895
Au	1.822363	6.976115	7.512417	C	8.588417	0.055122	10.858827	C	3.529135	3.456974	16.492829

C	4.823867	3.717943	6.836518
C	0.178591	6.640259	8.502028
P	1.829315	5.678452	4.658098
P	3.678080	7.601396	6.424964
C	0.165694	6.293508	5.032312
C	-0.694416	5.383687	5.645980
H	-0.339911	4.381190	5.859137
C	-1.973315	5.773934	5.987921
H	-2.642478	5.075736	6.468491
C	-2.393252	7.069326	5.724950
H	-3.389915	7.377614	6.004213
C	-1.539181	7.971207	5.111503
H	-1.872286	8.978908	4.907704
C	-0.255966	7.589922	4.761117
H	0.414059	8.293484	4.282043
C	1.506099	4.610980	3.217374
C	0.283726	4.538982	2.555781
H	-0.555193	5.134806	2.889390
C	0.142894	3.698512	1.464766
H	-0.808023	3.642049	0.954537
C	1.212146	2.934587	1.025888
H	1.094505	2.281157	0.173841
C	2.431548	3.004536	1.682747
H	3.266030	2.407265	1.344855
C	2.576745	3.834403	2.777608
H	3.521331	3.885586	3.308844
C	2.709867	7.048247	3.862577
C	2.574905	7.316948	2.502631
H	1.833156	6.784553	1.922380
C	3.385356	8.259368	1.895694
H	3.270083	8.465329	0.841721
C	4.350161	8.928813	2.634105
H	4.996974	9.648869	2.154776
C	4.476164	8.687855	3.989755
H	5.210644	9.228010	4.573420
C	3.659973	7.746912	4.614646
C	5.325581	7.048340	6.918279
C	6.362531	6.750779	6.043208
H	6.201469	6.770156	4.973411
C	7.605163	6.425007	6.556627
H	8.419723	6.199033	5.883823
C	7.810434	6.392642	7.926775
H	8.792012	6.159140	8.315018
C	6.763235	6.651317	8.799421
H	6.915882	6.600533	9.868348
C	5.519392	6.976556	8.296490
H	4.687634	7.186462	8.960797
C	3.798929	9.395453	6.777290
C	2.626841	10.114352	6.546360
H	1.743229	9.586773	6.201397
C	2.588523	11.477028	6.762965
H	1.677950	12.028714	6.578749
C	3.719780	12.132802	7.225949
H	3.691624	13.198004	7.403080
C	4.882496	11.421270	7.468321
H	5.762483	11.931087	7.833907
C	4.929561	10.055207	7.243423
H	5.844683	9.510237	7.432210
C	-0.797512	6.377482	9.170359
C	7.819805	-1.124063	10.742154
C	8.315368	0.867077	9.736945
C	7.070953	-1.042058	9.548027
C	7.370044	0.194375	8.929323
C	8.984821	0.570005	12.079321
C	7.401445	-1.854404	11.839167
C	8.413772	2.247239	9.777425
C	5.864261	-1.695295	9.378386
C	6.456466	0.852891	8.129527
C	8.741536	-0.298195	13.191146
C	9.318859	1.963395	12.047634
C	7.993541	-1.446701	13.077812
C	6.272613	-2.699105	11.586075
C	9.052118	2.755849	10.957298
C	7.614863	2.927322	8.813159
C	5.547181	-2.625370	10.420242
C	5.035465	-1.140777	8.349917
C	6.659552	2.280062	8.037747
C	5.313778	0.071552	7.764579
H	9.078139	0.007578	14.173853
H	9.705704	2.427005	12.946509
H	7.764659	-2.009275	13.974119
H	5.921721	-3.350320	12.376751
H	9.233236	3.820989	11.032189
H	7.638846	4.007524	8.773112
H	4.647533	-3.221241	10.329634
H	4.106338	-1.638445	8.102124
H	4.595859	0.501109	7.077318
C	-2.418902	4.754058	9.930083
H	-3.803446	1.189505	12.590538
C	-2.108302	6.083370	16.204813
H	-3.329096	2.054017	10.387477
H	-0.360226	8.837065	13.501585
C	-3.315399	5.421346	14.283677
C	-1.077858	8.103255	13.156969
C	-1.806131	5.992721	10.083673
C	-3.163664	3.724558	15.923072
C	-2.865726	6.242909	12.198728
C	-3.451752	4.965029	12.048288
C	-3.183067	4.143149	10.966642
C	-2.917677	5.089435	15.565573
H	-0.913132	7.825491	16.037346
C	-1.133492	7.811062	11.815199
C	-1.597701	7.162440	15.523141
C	-3.447946	2.753287	11.205874
H	-2.169672	4.175784	9.048065
C	-3.560976	2.784565	15.000922
C	-1.835889	7.368844	14.125456
C	-1.964273	6.767881	11.293337
C	-3.723895	4.456379	13.336678
C	-3.756398	3.102837	13.618917
H	-2.937926	3.401126	16.931415
C	-3.715896	2.260931	12.459643
H	-1.810824	5.929584	17.234500
H	-0.454547	8.313643	11.138121
C	-2.788978	6.527656	13.581999
C	1.422555	2.471143	16.305371
C	2.409637	1.419680	16.300020
C	2.114888	3.730548	16.424669
C	5.433748	0.514190	13.702502
C	6.124636	1.773060	13.821850
C	5.727221	2.384231	15.066091
C	4.789502	1.502514	15.716138
C	4.608371	0.346670	14.872943
C	5.766060	4.578144	13.972574
C	4.779867	5.629761	13.977381
C	3.955260	5.461559	15.147847
C	4.432036	4.306063	15.866608
C	5.551381	3.759804	15.140468
C	2.003847	6.166092	13.842133
C	0.703803	5.554842	13.727107
C	0.486784	4.738923	14.894263
C	1.655175	4.842995	15.732016
C	2.593276	5.724827	15.081461
C	-0.654905	3.085266	13.493306
C	-0.474546	1.657183	13.416048
C	0.114788	1.215799	14.655135
C	0.296513	2.372002	15.498352
C	-0.180370	3.527079	14.779916
C	1.462947	-0.407343	13.406011
C	2.877304	-0.681224	13.474396
C	3.354330	-0.239822	14.761760
C	2.234950	0.306868	15.488002
C	1.065803	0.203286	14.650114
C	3.082093	-0.074997	11.108794
C	4.016564	0.809456	10.461994
C	5.185903	0.909197	11.297832
C	4.973093	0.090800	12.463479
C	3.671793	-0.517983	12.347533
C	5.847224	2.121760	11.413722
C	5.374553	3.276834	10.692180
C	5.558197	4.431189	11.533957
C	6.147323	3.989520	12.774099
C	6.328465	2.561318	12.697704
C	4.609195	5.445710	11.537478
C	3.441121	5.344580	10.696623
C	2.322255	5.889259	11.425936
C	2.797933	6.328983	12.714031
C	4.211844	6.054796	12.783000
C	1.073422	5.296004	11.318724
C	0.890851	4.143025	10.475239
C	-0.045792	3.261638	11.125187
C	-0.440663	3.869445	12.370752
C	0.250818	5.126069	12.488476
C	0.123766	1.886801	11.048838
C	1.243280	1.341666	10.321563
C	1.720068	0.187206	11.041497
C	0.894953	0.018732	12.212335
C	-0.092056	1.069594	12.217577
C	2.146366	2.192357	9.695581
C	1.964358	3.621077	9.769021
C	3.266318	4.231730	9.884077
C	4.250777	3.180215	9.882574
C	3.558814	1.920959	9.769014
H	-3.633565	1.749847	15.312059

Energy Decomposition Analysis (EDA)[18] was calculated in the gas phase over the optimized structures of the supramolecular adducts with the same level of theory (PBE0-D3BJ/LANL2DZ//Def2TZVP)[9,11] in ADF suite.[19] The interaction energy contributions, as defined by the software, are as follows (Equation 12):

$$E_{int} = V_{elstat} + E_{Pauli} + E_{oi} + E_{disp} \qquad \text{eq. 12}$$

Where V_{elstat} refers to the electrostatic classical-like Coulombic interactions E_{Pauli} represents the Pauli exchange-type repulsions between filled orbitals, E_{oi} (orbital interactions or charge transfer) cover the attractive interactions between filled and unfilled orbitals of different fragments and E_{disp} corresponds to London-type weak interactions between polarizable electron clouds. Computed energies are shown in Table S11. For a discussion, refer to the main text.

Table S10. Summary of EDA contributions and interaction energies of computed structures in all supramolecular adducts.[a]

Adduct	V_{elstat}[b]	E_{Pauli}	E_{oi}[b]	E_{disp}[b]	E_{int}
C_{60}@CAudppe	-24.36 (32.9)	41.87	-12.47 (16.8)	-37.27 (50.3)	-32.23
C_{60}@CAudppf	-23.70 (32.4)	40.84	-12.63 (17.3)	-36.79 (50.3)	-32.28
C_{60}@CAudppbenz	-21.01 (30.6)	38.41	-11.75 (17.1)	-35.81 (52.2)	-30.16
C_{60}@CAuxantphos	-22.38 (31.2)	41.39	-12.24 (17.1)	-37.14 (51.8)	-30.37

[a] in kcal/mol

[b] Values in parenthesis indicate the percentage of contribution among all the attractive (negative) energies.

The same analysis was also carried out to extract information about the intramolecular aurophilic interaction by using Cockroft's fragmentation pattern[20] on the optimized geometries of all inclusion complexes after guest removal (Figure S148).

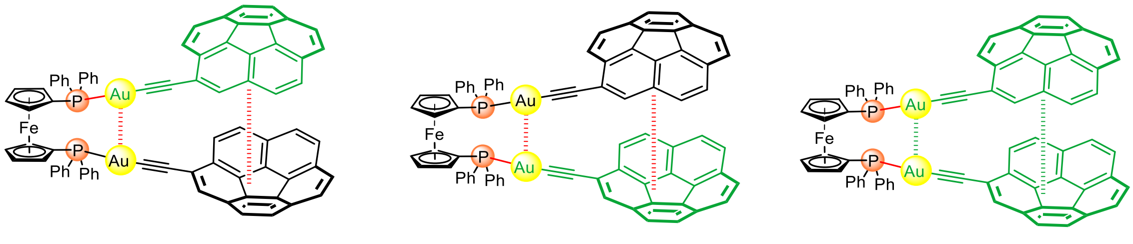

Figure S148. Fragmentation pattern for host **CAudppf** using the minimum obtained by geometry optimization of the corresponding C_{60} adduct. Green and black correspond to the two fragments treated by each calculation whereas red solid and dotted lines correspond to the bonds/interactions that are being assessed.

Therefore, it is possible to clear out the diphosphine ligand and know the Au-CC-corannulene contributions by the following Equation 13.

$$\Delta\Delta E_{[Au-CC-cora]2} = (A + B - AB)/2 \quad \text{eq. 13}$$

To isolate the contribution of corannulene alkynyl units, the dimer depicted in Figure S149 was used by using the parent host structure, removing the metal fragment, and saturating the sp carbons with hydrogens. The H – C_{sp} distance was reoptimized freezing the rest of the system.

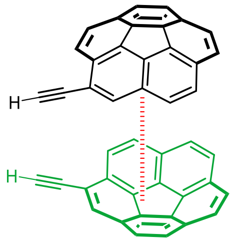

[H-CC-cora]$_2$

Figure S149. Fragment [H-CC-cora]2. For color coding, refer to **Figure S141**.

Finally, the Au(I)-Au(I) interaction was calculated according to Equation 14.

$$\Delta\Delta E_{[Au]2} = \Delta\Delta E_{[Au-CC-cora]2} - \Delta E_{[H-CC-cora]2} \quad \text{eq. 14}$$

This formalism was applied to all hosts and the results are gathered in Table S11.

Table S11. Summary of EDA contributions and interaction energies of computed structures in all hosts with the geometry of the supramolecular adducts after C_{60} removal according to Cockroft's fragmentation pattern as described above.[a]

Host	V_{elstat} [b]	E_{Pauli}	E_{oi} [b]	E_{disp} [b]	E_{int}
CAudppe					
$[Au\text{-}CC\text{-}cora]_2$	1.39[c]	-0.745[c]	-0.22 (48)	-0.24 (52)	0.20
$[H\text{-}CC\text{-}cora]_2$	-0.03 (27)	0	0 (0)	-0.8 (73)	-0.11
$[Au]_2$	1.42[c]	-0.75[c]	-0.22 (58)	-0.16 (42)	**0.31**
CAudppf					
$[Au\text{-}CC\text{-}cora]_2$	-9.46 (45)	13.82	-7.04 (33)	-4.61 (22)	-7.29
$[H\text{-}CC\text{-}cora]_2$	-0.04 (2)	0.23	-0.34 (21)	-1.26 (77)	-1.41
$[Au]_2$	-9.42 (49)	13.59	-6.70 (34)	-3.35 (17)	**-5.88**
CAudppbenz					
$[Au\text{-}CC\text{-}cora]_2$	-5.83 (41)	5.92	-3.45 (25)	-4.82 (34)	-8.18
$[H\text{-}CC\text{-}cora]_2$	-0.30 (6)	3.01	-1.72 (34)	-3.04 (60)	-2.05
$[Au]_2$	-5.53 (61)	2.91	-1.73 (18)	-1.78 (20)	**-6.13**
CAuxantphos					
$[Au\text{-}CC\text{-}cora]_2$	-17.46 (64)	18.47	-6.62 (25)	-2.96 (11)	-8.57
$[H\text{-}CC\text{-}cora]_2$	-0.01 (2)	-0.08[c]	-0.07 (12)	-0.51 (86)	-0.67
$[Au]_2$	-17.45 (66)	18.55	-6.55 (25)	-2.45 (9)	**-7.90**

[a] in kcal/mol. Overall Au(I)-Au(I) interaction energies are highlighted in bold.
[b] Values in parenthesis indicate the percentage of contribution among all the attractive (negative) energies.
[c] These values resulted with an unusual sign and, therefore, they were not used for the contribution percentage calculation.

Overall, Au(I)-Au(I) interaction energies follow the trend obtained by the empirical method (Table S8), being near zero for host **CAudppe** and high for **CAudppbenz** and **CAuxantphos** complexes. Interestingly, computed aurophilic interaction for compound **CAudppf** is more stabilizing than expected, reinforcing once more the idea of intramolecular gold bonding turn-on. EDA shows that dispersive forces are less important than orbital and electrostatic, as already shown by Cockroft[20] and Nitsch and Guerra,[21] confirming the latest findings in this regard.

Both EDA-based aurophilic interaction energies (Equation 14) and the ones obtained by the empirical Equation 9 correlate moderately well, with the exception of host **CAudppf**, whose EDA energy predicts a more stabilizing situation (Figure S150).

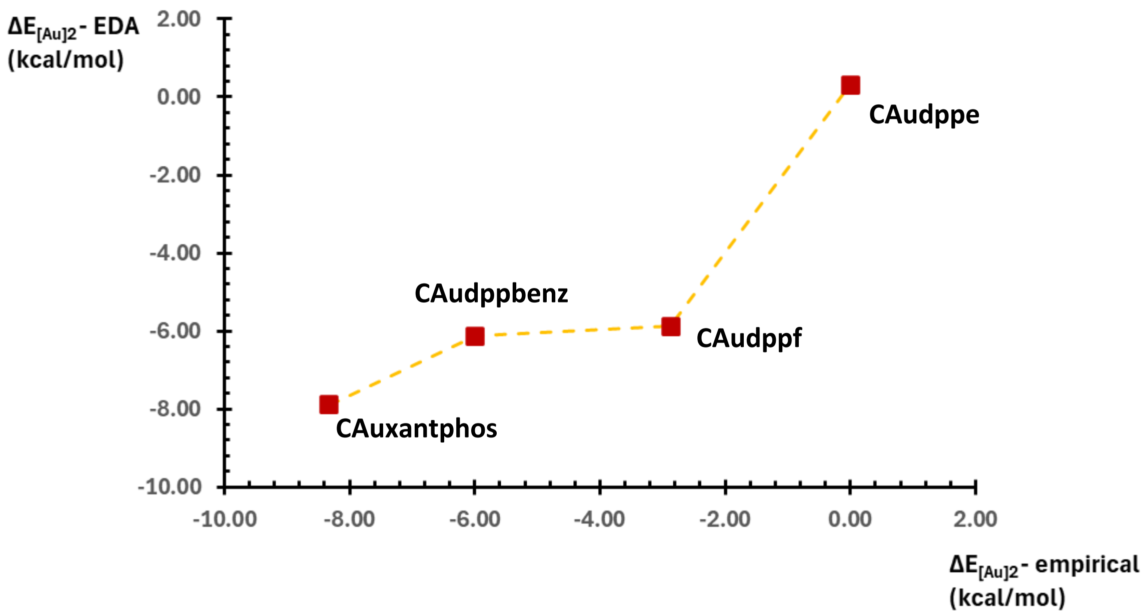

Figure S150. Correlation of the Au(I)-Au(I) interaction energies obtained by EDA (Equation 14, Table S12) and by the empirical formula (Equation 9, Table S9).

A real-space quantum topology analysis based on the Quantum Theory of Atoms in Molecules (QTAIM) called Interacting Quantum Atoms (IQA)[22] was calculated, again, in the gas phase over the optimized structures of the supramolecular adducts with the same level of theory (PBE0-D3BJ/LANL2DZ//Def2TZVP)[9,11] in ADF suite.[19] The Au(I)-Au(I) interaction energy is shown in the following table.

Table S12. Au(I)-Au(I) interaction energies by IQA analysis of computed structures in all hosts with the geometry of the supramolecular adducts after C_{60} removal.

**Adduct**	**E_{int} / kcal·mol^{-1} (IQA)**
C_{60}@**CAudppe**	3.3
C_{60}@**CAudppf**	-8.8
C_{60}@**CAudppbenz**	-16.0
C_{60}@**CAuxantphos**	-19.8

IQA-based aurophilic interactions (Table S12) correlate very well with empirical estimation (Table S8) since a quasi-linear trend is observed (Figure S151). Consequently, there is a similar correlation with the energies obtained by EDA calculations (Table S11) if compared to the EDA-empirical energy correlation (Figure S150). Unlike in EDA, a more stabilizing situation for host **CAudppf** is therefore not observed.

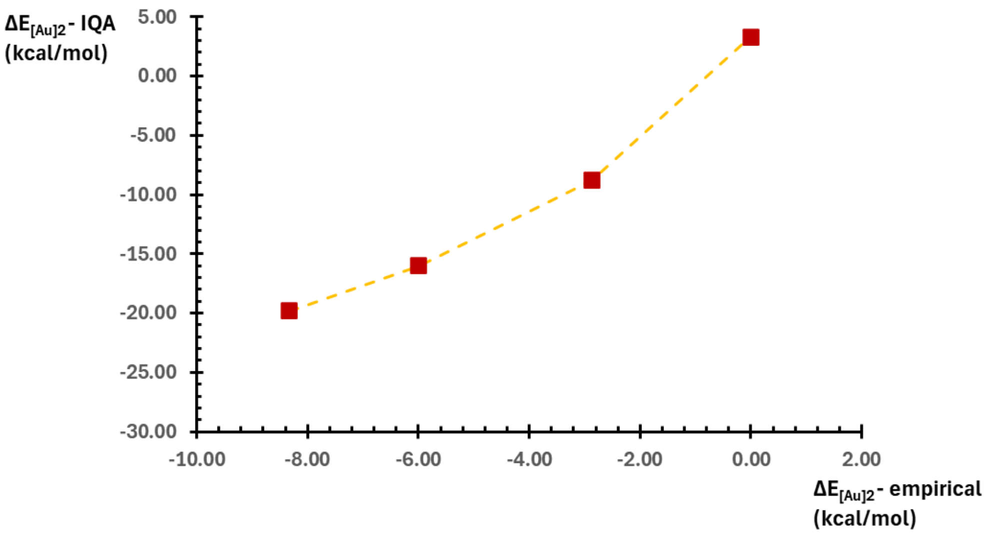

Figure S151. Correlation of the Au(I)-Au(I) interaction energies obtained by IQA (Table S12) and by the empirical formula (Equation 9, Table S8).

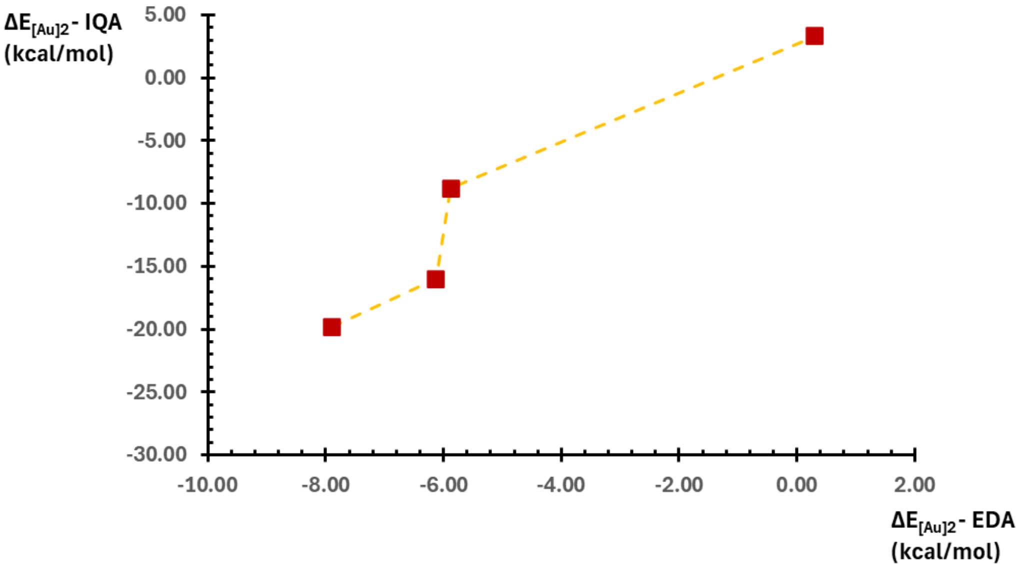

Figure S152. Correlation of the Au(I)-Au(I) interaction energies obtained by IQA (Table S12) and by EDA (Equation 14, Table S11).

For compound **CAudppf** a Potential Energy Surface (PES) rigid scan was performed to evaluate the energy barrier for the conversion between the *anti* and *syn* conformers, using PM6[23] semi-empirical method in Gaussian 16 Rev C.01.[12] To do so, the dihedral angle formed by the atoms labeled in Figure S153 was varied 360° in 36 steps of 10° each.

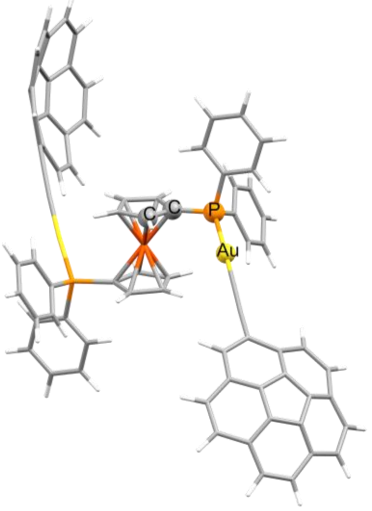

Figure S153. Initial structure of complex **CAudppf** for the rigid scan. The atoms involved in the dihedral angle that has been modified are labelled.

The resulting PES is shown in Figure S154. The optimized structures with GFN2 – xTB[6] of selected minima and maxima are also shown.

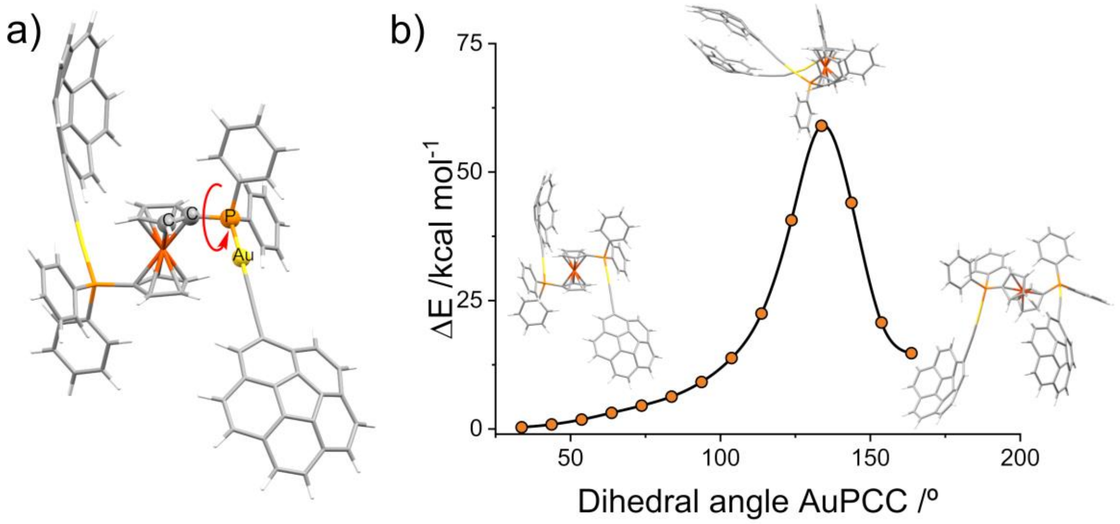

Figure S154. (a) Anti conformation of compound CAudppf. Highlighted atoms and red arrow represent the dihedral angle subject to torsion to reach the corresponding *syn* conformation for barrier height estimation. (b) Potential energy scan using the semiempirical PM6 method. Some key structures are displayed in the graph.

To estimate computationally the oxidation potential of Au(I) species, the following thermochemical cycle[24] was applied for the simplest complexes, **PAuPPh3** and **CAuPPh3** (Scheme S1).

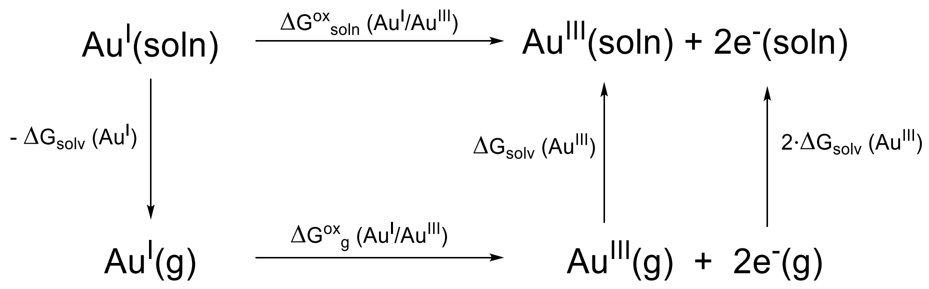

Scheme S1. Thermochemical cycle for the two-electron oxidation of Au(I) species.

According to above scheme the Gibbs free energy of the oxidation process can be described as (Equation 15):

$$\Delta G_{soln}^{ox}(Au^I/Au^{III}) = -\Delta G_{solv}(Au^I) + \Delta G_g^{ox}(Au^I/Au^{III}) + \Delta G_{solv}(Au^{III}) + 2 \cdot \Delta G_{solv}(e^-) \qquad \text{eq. 15}$$

Where all solvation terms can be calculated according to Equation 16 for any species X.

$$\Delta G_{solv}(X) = G_{soln}(X) - G_g(X) \qquad \text{eq. 16}$$

And the oxidation term in gas phase was estimated based on a simpler version of the current method[24a,25] according to Equation 17.

$$\Delta G_g^{ox}(Au^I/Au^{III}) = G_g(Au^{III}) + 2 \cdot G_g(e^-) - G_g(Au^I) \qquad \text{eq. 17}$$

All calculations were done on the optimized structures at the same DFT level of theory with the unrestricted approach (UPBE0-D3BJ/LANL2DZ//Def2TZVP). With respect to solvent considerations, the SMD model was applied using DMF as the solvent of choice. $G_g(e^-)$ and $\Delta G_{solv}(e^-)$ are tabulated values obtained by highly accurate methods[26] being -0.9 kcal/mol and -21.5 kcal/mol, respectively.

This gave Gibbs free energy values of 204.7 kcal/mol and 115.0 kcal/mol for complexes **PAuPPh3** and **CAuPPh3**, respectively. They can readily be converted into absolute oxidation potentials by Equation 18.

$$E_{ox}^{\circ}(Au^I/Au^{III}) = -\frac{\Delta G_{soln}^{ox}(Au^I/Au^{III})}{nF} \qquad \text{eq. 18}$$

Being n the number of electrons ($n = 2$) and F the Faraday constant.

This furnishes absolute oxidation potentials of -4.44 V and -2.49 V for complexes **PAuPPh3** and **CAuPPh3**, respectively. Taking into account the absolute reduction potential of the Fc^+/Fc couple[37] (4.980 V), used as a reference in the experimental study, the final oxidation potentials for **PAuPPh3** and **CAuPPh3** species resulted in 0.54 V and 2.49 V, respectively. Albeit this method is just an estimation, the oxidation potential value for compound **PAuPPh3** is close to the experimental value (0.90 V) and, more importantly, it furnishes an oxidation potential for compound **CAuPPh3** which turned to be experimentally inaccessible, requiring an excess of voltage (ΔV) of 1.95 V with respect to the oxidation of the former complex, clearly falling outside the allowed electrochemical window.

Regarding the geometry of the supramolecular adduct formed between host **$CAuPPh_3$** and C_{70}, three starting structures (namely v_1, v_2 and v_3) of the complex **C_{70}@$CAuPPh_3$**, whose difference relies on the relative cartesian orientations of C_{70}, were used for the optimization with GFN2-

xTB. This provided an assortment of three inclusion complexes whose relative Gibbs free energy was not higher than 2.6 kcal·mol^{-1}. Only the most stable one (lowest in energy) is shown.

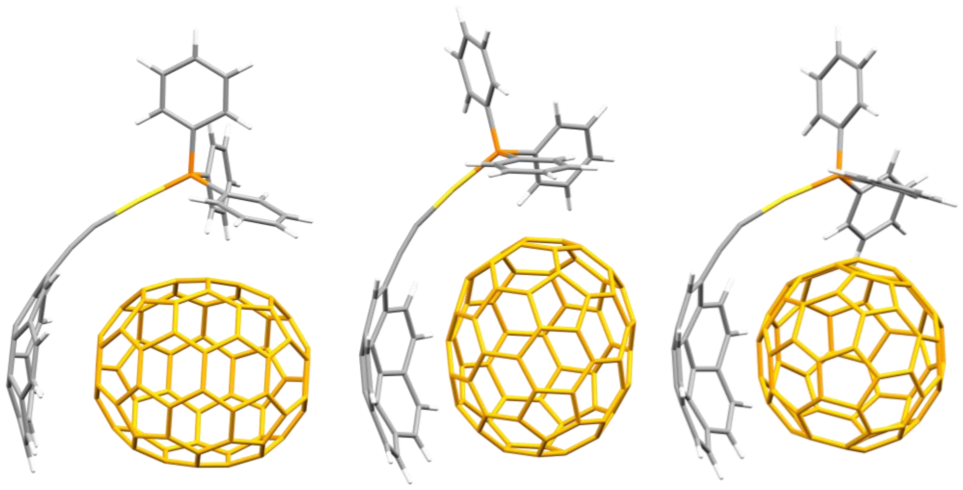

Figure S155. Starting geometries of C_{70}@**CAuPPh$_3$** with different orientations of C_{70}.

C_{70}@CAuPPh$_3$ v2 (G = -259.213944 a.u.)

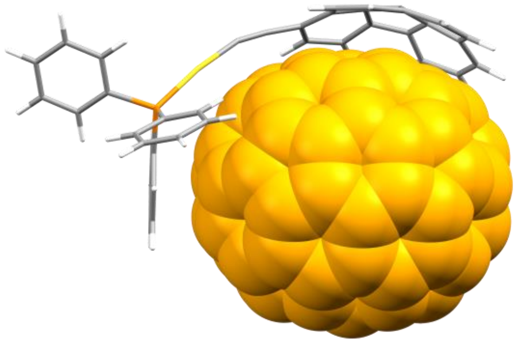

Au	5.722619	6.871593	16.845077	C	9.492116	12.865366	22.553589	C	0.162039	14.488733	20.825841
C	7.293282	7.757313	17.608597	C	8.872401	11.200472	17.153941	C	4.352044	9.452442	17.799577
C	8.019126	8.480325	18.260215	C	8.753405	9.405359	19.037708	C	4.396310	10.610122	16.943627
C	2.842407	6.870112	14.864287	C	8.975877	12.548087	16.899784	C	3.127864	8.880073	18.132533
C	3.183169	8.193992	14.611316	C	9.203520	14.888270	18.016874	C	3.225894	11.143780	16.436975
H	3.995779	8.647894	15.162382	C	8.794205	9.231616	20.418065	C	3.062874	12.573222	16.383044
C	2.491521	8.918077	13.656640	C	8.959473	10.467874	22.693378	C	4.110368	13.450623	16.738603
H	2.753245	9.949789	13.471079	C	9.289758	15.543200	19.222662	C	3.716617	14.737836	17.163502
C	1.469287	8.316762	12.941162	C	9.327967	15.204114	21.804873	C	1.347050	14.056615	17.369201
H	0.928769	8.880322	12.194837	C	9.067508	11.705507	23.281328	C	1.705184	12.870757	16.691765
C	1.143089	6.988600	13.173911	C	9.267597	14.257987	22.802186	C	2.358874	15.035313	17.473629
H	0.352853	6.518868	12.606203	H	8.427732	10.557232	16.405064	C	3.864359	8.645406	20.457067
C	1.824064	6.261966	14.132841	H	8.611560	12.928802	15.953863	C	5.123185	9.248504	20.109464
H	1.572028	5.225585	14.316575	H	8.862083	15.439776	17.149779	C	2.880837	8.466858	19.491871
C	2.546587	5.846048	17.530897	H	8.330754	8.340935	20.825431	C	5.361416	9.646947	18.809233
C	1.211023	6.220232	17.442941	H	8.540685	9.653381	23.271387	C	6.023367	10.897796	18.560519
H	0.813158	6.616393	16.518980	H	9.015215	16.589687	19.266965	C	6.541970	11.682096	19.611202
C	0.391269	6.098675	18.552168	H	9.055043	16.224963	22.040159	C	6.683404	13.057070	19.337939
H	-0.645831	6.394207	18.483880	H	8.733673	11.825560	24.304434	C	5.339374	12.889404	17.268652
C	0.895752	5.610572	19.746601	H	8.951556	14.566228	23.790927	C	5.436084	11.488289	17.409086
H	0.251534	5.524657	20.609257	C	1.175258	9.233447	21.071813	C	6.092225	13.650951	18.187088
C	2.229898	5.244151	19.839493	C	2.196927	9.422522	22.065015	C	6.296406	11.263068	20.976599
H	2.627630	4.872591	20.772636	C	1.507885	8.767226	19.804527	C	5.548931	10.086507	21.197800
C	3.054532	5.366642	18.737648	C	3.515638	9.134790	21.763862	C	6.204310	12.249069	21.979885
H	4.102852	5.097879	18.801823	C	4.559373	10.015356	22.219174	C	6.099168	15.248794	19.891891
C	3.827247	4.302775	15.500630	C	4.282905	11.119707	23.054423	C	6.685763	14.034495	20.391165
C	3.120199	3.219929	16.009819	C	5.214353	12.179148	23.001671	C	6.449781	13.637003	21.693024
H	2.449114	3.351726	16.847773	C	-0.000010	10.411217	18.793449	C	5.287327	16.023742	20.710905
C	3.278548	1.969798	15.435846	C	-0.334202	10.894592	20.106433	C	3.710544	16.342133	18.863206
H	2.727556	1.127928	15.829681	C	0.906854	9.368618	18.640786	C	4.553182	15.535647	18.021550
C	4.137475	1.795428	14.363221	C	0.242057	10.317969	21.222707	C	5.723674	14.999838	18.525729
H	4.256302	0.817031	13.921517	C	0.678362	11.153730	22.310371	C	4.065705	16.581841	20.185801
C	4.849459	2.873644	13.858543	C	0.443074	12.545611	22.312747	C	1.748300	16.205129	20.884768
H	5.523271	2.736863	13.025383	C	1.331727	13.318065	23.091329	C	1.388769	15.951472	19.515586
C	4.699941	4.122659	14.428701	C	2.898507	11.423341	23.368593	C	2.350530	16.018493	18.524815
H	5.256272	4.972921	14.051273	C	1.884627	10.602464	22.828720	C	3.062998	16.511389	21.218753
P	3.745573	5.991178	16.172540	C	2.538083	12.766615	23.609873	C	2.924704	15.027712	23.161994
C	9.852111	11.499466	19.284501	C	1.959911	10.547382	16.769483	C	1.567478	14.709198	22.807294
C	9.950516	12.885740	19.024922	C	1.024281	11.626518	16.938370	C	0.990724	15.286576	21.691452
C	9.872074	11.317577	20.685588	C	1.908305	9.438169	17.606863	C	3.664206	15.910118	22.382638
C	10.031642	13.557264	20.263965	C	0.063002	11.559614	17.929928	C	5.614374	14.437239	22.547746
C	9.977661	12.588142	21.289253	C	-0.245255	12.734090	18.703058	C	4.841991	13.525438	23.349140
C	9.215639	10.630282	18.421487	C	0.337589	13.985844	18.409673	C	3.523879	13.814934	23.649966
C	9.430034	13.478401	17.889961	C	0.407629	14.898605	19.484345	C	5.038912	15.608437	22.069459
C	9.259082	10.243565	21.307631	C	-0.161779	13.151189	21.140380				
C	9.608787	14.861400	20.442669	C	-0.490455	12.324181	20.044550				

Although statistical factors in ^{1}H NMR titration experiments discard the possibility of formation of the adduct C_{70}@(**CAuPPh$_3$**)$_2$, the optimization of an assortment of six inclusion complexes has been performed using GFN2-xTB, which emerge from de relative disposition of the two **CAuPPh$_3$** molecules (*syn* or *anti*) and the relative cartesian orientations of C_{70} (Figure S147). Only the most stable inclusion complex for the *anti* and *syn* arrangements are shown. There is only an energy difference of 1 kcal mol^{-1} between those adducts, being the one with a *syn* disposition the most stable.

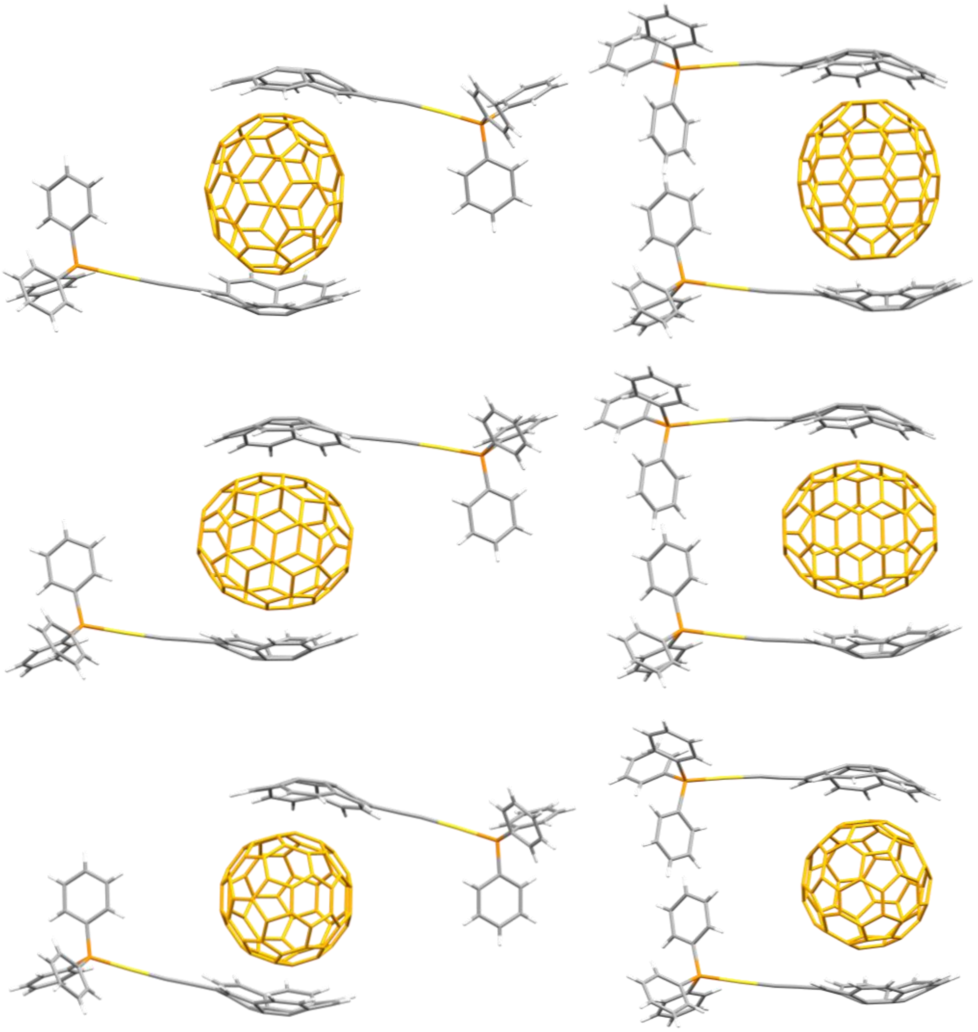

Figure S156. Starting geometries of C_{70}@(**CAuPPh$_3$**)$_2$ with different relative orientations of **CAuPPh$_3$** (*anti* on the left column and *syn* on the right column) with different orientations of C_{70} namely *anti* v_1-v_3 and *syn* v_1-v_3.

C_{70}@$(CAuPPh_3)_2$ *anti* v_2 (G = -358.587822 a.u.)

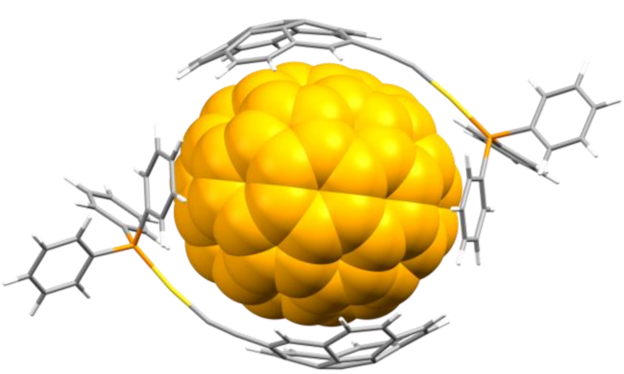

Au	5.726429	6.886669	16.810228	C	-2.344712	12.848149	23.581043	C	4.577303	10.025397	22.176602
C	7.299803	7.770049	17.567579	H	4.149056	18.136245	30.506426	C	4.316475	11.128108	23.019347
C	8.020150	8.486959	18.231406	C	-3.779759	12.620757	20.204540	C	5.249722	12.185791	22.955478
C	2.832067	6.841526	14.843685	C	6.305029	15.615856	25.362892	C	-0.032948	10.443418	18.821523
C	3.141237	8.173762	14.592741	H	7.194208	15.949426	24.847546	C	-0.346009	10.924466	20.139868
H	3.935829	8.649278	15.151730	C	3.356237	18.227579	29.777903	C	0.866925	9.398121	18.651383
C	2.441008	8.878972	13.630324	C	-3.759584	11.361014	19.568615	C	0.245393	10.343222	21.244796
H	2.678443	9.916806	13.446173	H	0.525308	18.530735	27.182442	C	0.703296	11.174161	22.326217
C	1.442034	8.250806	12.904924	C	2.239253	18.995879	30.061764	C	0.476110	12.565172	22.333040
H	0.896092	8.799002	12.151000	C	-3.394078	11.192919	18.246347	C	1.374311	13.332036	23.102365
C	1.146184	6.915549	13.137517	C	2.447593	17.693895	27.624082	C	2.937811	11.434104	23.354985
H	0.373678	6.425239	12.562709	H	4.431776	21.189068	24.593063	C	1.913836	10.617528	22.827751
C	1.835455	6.207533	14.104575	C	5.164867	16.399518	25.325546	C	2.584599	12.778581	23.603595
H	1.606786	5.165657	14.287731	H	-1.932001	7.532162	21.306895	C	1.896531	10.578902	16.767846
C	2.570963	5.845139	17.525966	H	-2.747887	9.625992	16.887943	C	0.965837	11.659903	16.953459
C	1.230106	6.201120	17.446003	P	2.443116	16.880852	25.995575	C	1.854006	9.467431	17.603083
H	0.818625	6.580713	16.520958	C	3.754452	20.386101	24.339998	C	0.019703	11.593493	17.959645
C	0.423543	6.080919	18.564642	C	1.218677	19.111098	29.129568	C	-0.274346	12.765966	18.739635
H	-0.618558	6.358111	18.502376	C	-0.900825	14.514301	25.000575	C	0.307393	14.016198	18.441089
C	0.945047	5.615101	19.760167	C	2.703440	18.299674	24.891749	C	0.397502	14.925629	19.517522
H	0.310646	5.530836	20.630385	H	3.111093	14.425316	27.156766	C	-0.144859	13.173458	21.173546
C	2.284408	5.266676	19.844568	C	-3.273515	13.646895	18.137229	C	-0.498905	12.353084	20.083541
H	2.695880	4.911985	20.778339	C	4.018186	15.975193	25.984995	C	0.175946	14.510568	20.860295
C	3.096170	5.385630	18.732960	H	-2.444429	15.470681	22.369728	C	4.301198	9.476046	17.759613
H	4.148290	5.130460	18.789920	H	0.344577	19.705470	29.352385	C	4.336445	10.634782	16.905396
C	3.849813	4.291413	15.504976	C	-2.772106	8.899807	18.879967	C	3.080452	8.905072	18.108911
C	3.144075	3.211643	16.022621	H	-2.839370	15.830242	20.006559	C	3.159409	11.172903	16.418382
H	2.466216	3.351222	16.853928	H	-1.701686	8.652824	23.432193	C	2.999616	12.602805	16.370464
C	3.312939	1.954948	15.466493	C	-3.252797	12.418965	17.518559	C	4.054921	13.476460	16.712498
H	2.762903	1.115564	15.867082	C	-2.233401	9.156417	22.634157	C	3.671013	14.763483	17.146831
C	4.180951	1.770798	14.402771	C	-2.974651	13.371084	22.388246	C	1.302207	14.087649	17.386616
H	4.307783	0.787381	13.974672	C	3.067410	20.411559	23.135048	C	1.647135	12.902890	16.700212
C	4.891724	2.845766	13.889736	H	2.161045	19.503229	31.011986	C	2.318779	15.063292	17.478197
H	5.572820	2.701373	13.063793	H	-1.638735	11.152938	24.646816	C	3.851538	8.662329	20.421404
C	4.731696	4.101319	14.442397	C	-3.055498	14.857474	20.430161	C	5.106479	9.263346	20.056569
H	5.287279	4.949190	14.058736	C	-2.218575	11.477656	23.790734	C	2.852587	8.489483	19.470576
P	3.754797	5.987695	16.154972	H	3.214710	21.233019	22.449193	C	5.325687	9.664968	18.754115
C	9.853366	11.485740	19.314366	H	-2.377629	7.940090	18.571925	C	5.989521	10.913470	18.498225
C	9.962929	12.874784	19.075443	C	2.186747	19.391797	22.814434	C	6.525783	11.693659	19.543523
C	9.859396	11.283661	20.712773	C	3.465941	17.573796	28.562261	C	6.666388	13.069289	19.271832
C	10.037031	13.528170	20.324514	C	-3.533515	10.376908	20.556201	C	5.290605	12.910748	17.222416
C	9.967207	12.544605	21.335255	H	1.643985	19.414384	21.880507	C	5.386049	11.508905	17.357345
C	9.219284	10.632964	18.433550	C	-2.985433	9.860113	17.918069	C	6.059255	13.667910	18.131630
C	9.455891	13.486570	17.944470	C	-2.833886	14.655743	21.772487	C	6.299146	11.271811	20.911658
C	9.233551	10.205078	21.313854	C	1.998537	18.340392	23.694144	C	5.551653	10.096714	21.140804
C	9.620701	14.832160	20.517846	H	-3.029878	12.374066	16.459912	C	6.224397	12.255396	21.919082
C	9.471717	12.806767	22.598807	H	7.197676	13.801533	26.066469	C	6.095361	15.261143	19.840659
C	8.890329	11.222912	17.171367	C	-3.434656	13.790412	19.553827	C	6.686154	14.044056	20.327659
C	8.746533	9.401427	19.027816	C	4.017421	14.756351	26.662173	C	6.467889	13.643494	21.631847
C	9.004298	12.573332	16.937336	Au	0.602514	15.639240	25.548981	C	5.297669	16.036368	20.674262
C	9.237403	14.895851	18.089270	C	-3.414508	11.027232	21.802327	C	3.694594	16.363440	18.850900
C	8.772742	9.208073	20.405930	H	4.340533	16.975429	28.345408	C	4.522204	15.556552	17.994743
C	8.920743	10.411800	22.699141	H	5.167956	17.330581	24.776334	C	5.699109	15.016814	18.479728
C	9.316807	15.533117	19.304943	C	-1.618573	13.742588	24.399626	C	4.070065	16.599662	20.169049
C	9.329811	15.157066	21.882290	H	4.105749	19.315336	26.164223	C	1.763503	16.223950	20.901281
C	9.030757	11.640319	23.305305	C	5.159792	13.980163	26.696922	C	1.382117	15.975309	19.536613
C	9.255714	14.197020	22.864983	H	-3.065370	14.529829	17.545854	C	2.329154	16.044044	18.531736
H	8.446746	10.593331	16.410260	C	3.575549	19.335697	25.220724	C	3.083474	16.529493	21.216524
H	8.649135	12.969758	15.994295	C	6.305911	14.410940	26.046125	C	2.970637	15.041620	23.160086
H	8.906333	15.461511	17.227215	H	5.155538	13.036430	27.222863	C	1.602978	14.727777	22.828448
H	8.300828	8.313918	20.795834	C	-3.565013	12.415341	21.586733	C	1.015684	15.304671	21.716034
H	8.488656	9.592482	23.260422	C	-2.936147	9.161481	20.277851	C	3.700371	15.923137	22.369747
H	9.047451	16.580426	19.361297	H	1.301217	17.546808	23.459176	C	5.647233	14.443171	22.500269
H	9.062193	16.176974	22.128457	C	1.319225	18.457810	27.916937	C	4.885938	13.531909	23.311367
H	8.684520	11.747250	24.325835	C	1.172922	9.256373	21.077238	C	3.573421	13.823243	23.631137
H	8.937587	14.490648	23.857174	C	2.210922	9.439173	22.055569	C	5.068661	15.618116	22.034660
C	-2.364348	8.518096	21.423766	C	1.484704	8.792068	19.804591				
C	-2.662514	10.510369	22.843429	C	3.524283	9.148242	21.734587				

C_{70}@$(CAuPPh_3)_2$ *syn* v_3 (G = -358.589483 a.u.)

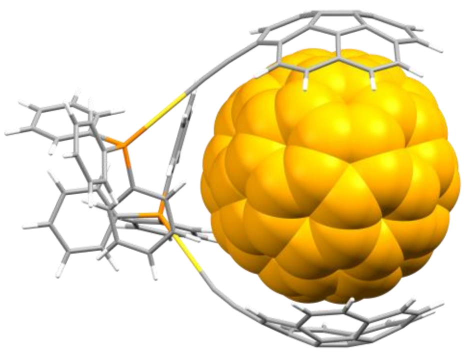

Au	8.341135	13.540122	25.472128	C	-1.183801	15.709993	24.012823	C	5.826555	11.883620	18.884612
C	9.186641	13.511182	23.706497	H	3.573293	16.829395	30.953447	C	5.849793	10.523846	19.256343
C	9.464136	13.383848	22.532875	C	-2.811656	15.138818	20.767739	C	6.521434	10.230614	20.461681
C	7.928780	12.026319	28.451890	C	8.354373	17.486667	26.146379	C	0.186207	12.744123	19.242091
C	7.892648	10.819315	27.755319	H	9.283351	17.600887	26.685727	C	0.447499	11.380933	18.867532
H	7.558614	10.810219	26.723999	C	4.032575	17.643987	30.412162	C	1.027372	13.762027	18.808943
C	8.278131	9.648294	28.379390	C	-3.350428	13.856539	20.526355	C	1.536226	11.078707	18.069695
H	8.244678	8.713321	27.839207	H	5.656350	20.563818	28.477082	C	2.333549	9.915865	18.361622
C	8.713968	9.678783	29.695605	C	4.648459	18.678696	31.098829	C	1.974090	9.013930	19.386479
H	9.022770	8.765178	30.181982	C	-3.161617	13.176903	19.337068	C	3.031951	8.259741	19.939407
C	8.757965	10.878847	30.387075	C	4.582913	18.706968	28.324776	C	4.747565	9.672006	18.850329
H	9.099503	10.899882	31.411803	H	6.503685	22.560192	24.215377	C	3.695834	10.238854	18.098187
C	8.364233	12.054827	29.771945	C	7.192039	18.029451	26.663784	C	4.393961	8.582737	19.675971
H	8.396455	12.990502	30.313509	H	-3.150846	10.300405	23.414792	C	0.962062	14.775341	21.035921
C	5.590363	13.476070	27.732498	H	-3.262973	11.160338	18.537668	C	0.097305	13.715620	21.478701
C	4.911492	12.495227	28.447510	P	4.445023	18.662450	26.510572	C	1.422934	14.801042	19.726067
H	5.457894	11.708058	28.948217	C	5.717848	22.204088	24.865834	C	-0.284134	12.721292	20.600367
C	3.529339	12.528358	28.515147	C	5.235061	19.723301	30.400331	C	-0.327634	11.355181	21.048714
H	3.003381	11.765042	29.070287	C	0.859878	16.997623	25.038979	C	-0.086333	11.013690	22.396247
C	2.821801	13.528349	27.868712	C	4.663840	20.406825	26.053283	C	0.370841	9.698532	22.626318
H	1.742723	13.548018	27.914320	H	5.034068	17.073260	24.227759	C	0.831012	9.330463	20.223824
C	3.496127	14.502231	27.148357	C	-2.060833	15.221646	18.528390	C	0.123504	10.528174	19.981302
H	2.938606	15.268472	26.623999	C	5.998162	17.894243	25.961354	C	0.822319	8.870990	21.558820
C	4.875210	14.476694	27.075888	H	-0.269631	17.666904	22.092881	C	3.656503	15.421549	20.517485
H	5.413568	15.224835	26.505398	H	5.714661	20.529874	30.936008	C	3.172417	15.391097	21.872103
C	7.947170	14.883052	28.561510	C	-3.471356	11.118765	20.647062	C	2.800952	15.129946	19.460717
C	7.104025	15.703228	29.300841	H	-0.647625	17.468066	19.708825	C	1.851607	15.072840	22.125061
H	6.038212	15.521804	29.316387	H	-2.367229	11.793013	25.142612	C	1.527510	14.212230	23.230525
C	7.636866	16.757272	30.023665	C	-2.580972	13.970690	18.294923	C	2.514087	13.760118	24.130462
H	6.980169	17.392641	30.599713	C	-2.686531	12.214354	24.197343	C	2.223150	12.557045	24.808634
C	9.000851	16.997007	30.012610	C	-1.620062	16.079806	22.685474	C	0.314215	12.058973	23.317916
H	9.408169	17.821068	30.579889	C	4.764583	23.085303	25.348399	C	0.446589	13.376034	22.831779
C	9.844551	16.181163	29.273011	H	4.668708	18.673599	32.179020	C	1.141195	11.722257	24.410323
H	10.908883	16.367033	29.262573	H	-1.183436	14.266107	25.569731	C	4.550795	13.781574	18.401674
C	9.318987	15.133176	28.543159	C	-1.216299	16.838363	20.381638	C	5.426742	14.078586	19.502082
H	9.964282	14.495215	27.950068	C	-1.631297	14.541169	24.622010	C	3.258193	14.292796	18.379849
P	7.395133	13.495802	27.528009	H	4.808868	24.129740	25.076971	C	4.990270	14.883300	20.537866
C	9.790865	11.581921	19.357901	H	-3.499721	10.036334	20.661243	C	5.327891	14.541981	21.895345
C	9.478724	10.323155	18.793298	C	3.747930	22.627549	26.174177	C	6.179015	13.457062	22.192194
C	9.368092	12.582455	18.453700	C	3.995413	17.659866	29.029794	C	6.027624	12.888010	23.475153
C	8.849548	10.548851	17.550253	C	-3.488596	13.199212	21.768706	C	3.901162	14.091741	23.865077
C	8.784299	11.943769	17.339136	H	2.998961	23.312724	26.543722	C	4.206981	14.855971	22.718336
C	9.846809	11.776519	20.723343	C	-3.336541	11.759090	19.436788	C	4.904371	13.195818	24.293733
C	9.243133	9.193037	19.553874	C	-1.002500	16.954452	21.735465	C	6.632066	12.613027	21.104687
C	8.994496	13.851350	18.863426	C	3.691264	21.291632	26.518989	C	6.211304	12.908583	19.792351
C	7.938740	9.665823	17.000005	H	-2.462490	13.533282	17.311491	C	6.904545	11.255965	21.368034
C	7.810426	12.535902	16.556167	H	9.241628	16.395897	24.527596	C	5.524863	10.986556	24.741865
C	9.838876	10.570483	21.496182	C	-2.059106	15.820598	19.829936	C	6.406450	11.522375	23.739212
C	9.606091	13.138130	21.147731	C	5.972464	17.190589	24.758508	C	6.840336	10.721029	22.701950
C	9.565434	9.342731	20.941702	Au	2.518272	17.790098	25.706712	C	5.105184	9.661847	24.669483
C	8.484952	8.179815	18.881449	C	-3.036159	14.074343	22.779225	C	2.828281	10.344554	25.260026
C	9.231873	14.125453	20.241066	H	3.500073	16.863987	28.488129	C	3.266823	11.712284	25.326987
C	8.170485	14.550342	17.917529	H	7.212286	18.560667	27.605409	C	4.588252	12.026704	25.071609
C	7.864073	8.404662	17.675733	C	-0.115757	16.425812	24.601467	C	3.726643	9.334601	24.933863
C	7.069384	10.251560	16.023939	H	6.414565	20.178134	24.828540	C	2.055703	8.310472	23.464270
C	7.610659	13.929165	16.827516	C	7.137781	16.647420	24.249925	C	1.132429	9.360954	23.800583
C	7.010822	11.609423	15.812587	H	-1.549134	15.730335	17.720932	C	1.511548	10.357834	24.680126
H	9.960816	10.648009	22.569285	C	5.670535	20.863726	25.210847	C	3.331838	8.295116	24.016125
H	9.484783	8.481725	21.593408	C	8.331133	16.804397	24.939181	C	4.275306	7.695145	21.836876
H	8.313181	7.237464	19.386506	H	7.120593	16.098056	23.320065	C	2.953364	7.718348	21.270981
H	8.963042	15.097465	20.638238	C	-2.608833	15.270979	22.161032	C	1.865070	8.019553	22.068724
H	7.900417	15.578567	18.125203	C	-3.449544	11.822793	21.893539	C	4.466100	7.980151	23.184428
H	7.222815	7.632742	17.269037	H	2.897265	20.919997	27.156477	C	6.418094	9.349287	22.625039
H	6.368707	9.613455	15.500312	C	5.206291	19.741627	29.017374	C	6.210812	9.055381	21.232038
H	6.917905	14.486903	16.209472	C	2.406650	12.128322	17.612743	C	5.162591	8.243006	20.845317
H	6.265454	11.996747	15.129069	C	3.746396	11.603752	17.643169	C	5.562390	8.825401	23.587901
C	-3.131740	11.364381	23.213870	C	2.161387	13.447801	17.976789				
C	-2.504162	13.620266	23.973755	C	4.796473	12.413653	18.031312				

References

1. (a) Williams, D. B. G.; Lawton, M. Drying of Organic Solvents: Quantitative Evaluation of the Efficiency of Several Desiccants. *J Org Chem* **2010**, *75* (24), 8351–8354. (b) Armarego, W. L. F.; Chai, C. L. L. *Purification of Laboratory Chemicals*, 7th ed.; Elsevier, 2013.
2. Elgrishi, N.; Rountree, K. J.; McCarthy, B. D.; Rountree, E. S.; Eisenhart, T. T.; Dempsey, J. L. A Practical Beginner's Guide to Cyclic Voltammetry. *J. Chem. Educ.* **2018**, *95*, 197–206.
3. (a) Jones, C. S.; Elliott, E.; Siegel, J. S. Synthesis and Properties of Monosubstituted Ethynylcorannulenes. *Synlett* **2004**, 187–191. (b) Sacristán-Martín, A.; Barbero, H.; Ferrero, S.; Miguel, D.; García-Rodríguez, R.; Álvarez, C. M. ON/OFF Metal-Triggered Molecular Tweezers for Fullerene Recognition. *Chem. Commun.* **2021**, *57*, 11013–11016.
4. (a) Wang, M.-Z.; Wong, M.-K.; Che, C.-M. Gold(I)-Catalyzed Intermolecular Hydroarylation of Alkenes with Indoles under Thermal and Microwave-Assisted Conditions. *Chem. – Eur. J.* **2008**, *14*, 8353–8364. (b) Tamai, T.; Fujiwara, K.; Higashimae, S.; Nomoto, A.; Ogawa, A. Gold-Catalyzed Anti -Markovnikov Selective Hydrothiolation of Unactivated Alkenes. *Org. Lett.* **2016**, *18*, 2114–2117.
5. (a) Thordarson, P. Determining Association Constants from Titration Experiments in Supramolecular Chemistry. *Chem. Soc. Rev.* **2011**, *40*, 1305–1323. (b) Thordarson, P. Binding Constants and Their Measurement. In *Supramolecular Chemistry*; Gale, P. A., Steed, J. W., Eds.; Wiley: Chichester, UK, 2012; pp 239–274. (c) Brynn Hibbert, D.; Thordarson, P. The Death of the Job Plot, Transparency, Open Science and Online Tools, Uncertainty Estimation Methods and Other Developments in Supramolecular Chemistry Data Analysis. *Chem. Commun.* **2016**, *52*, 12792–12805.
6. Bannwarth, C.; Ehlert, S.; Grimme, S. GFN2-XTB—An Accurate and Broadly Parametrized Self-Consistent Tight-Binding Quantum Chemical Method with Multipole Electrostatics and Density-Dependent Dispersion Contributions. *J. Chem. Theory Comput.* **2019**, *15*, 1652–1671.
7. Yanai, T.; Tew, D. P.; Handy, N. C. A New Hybrid Exchange–Correlation Functional Using the Coulomb-Attenuating Method (CAM-B3LYP). *Chem. Phys. Lett.* **2004**, *393*, 51–57.
8. Chai, J.-D.; Head-Gordon, M. Long-Range Corrected Hybrid Density Functionals with Damped Atom–Atom Dispersion Corrections. *Phys. Chem. Chem. Phys.* **2008**, *10*, 6615.
9. (a) Ernzerhof, M.; Scuseria, G. E. Assessment of the Perdew–Burke–Ernzerhof Exchange-Correlation Functional. *J. Chem. Phys.* **1999**, *110*, 5029–5036. (b) Adamo, C.; Barone, V. Toward Reliable Density Functional Methods without Adjustable Parameters: The PBE0 Model. *J. Chem. Phys.* **1999**, *110*, 6158–6170.
10. (a) Staroverov, V. N.; Scuseria, G. E.; Tao, J.; Perdew, J. P. Comparative Assessment of a New Nonempirical Density Functional: Molecules and Hydrogen-Bonded Complexes. *J. Chem. Phys.* **2003**, *119*, 12129–12137. (b) Tao, J.; Perdew, J. P.; Staroverov, V. N.; Scuseria, G. E. Climbing the Density Functional Ladder: Nonempirical Meta–Generalized Gradient Approximation Designed for Molecules and Solids. *Phys. Rev. Lett.* **2003**, *91*, 146401.
11. (a) Grimme, S.; Ehrlich, S.; Goerigk, L. Effect of the Damping Function in Dispersion Corrected Density Functional Theory. *J. Comput. Chem.* **2011**, *32*, 1456–1465. (b) Hay, P. J.; Wadt, W. R. Ab Initio Effective Core Potentials for Molecular Calculations. Potentials for the Transition Metal Atoms Sc to Hg. *J. Chem. Phys.* **1985**, *82*, 270–283. (c) Weigend, F.; Ahlrichs, R. Balanced Basis Sets of Split Valence, Triple Zeta Valence and

Quadruple Zeta Valence Quality for H to Rn: Design and Assessment of Accuracy. *Phys. Chem. Chem. Phys.* **2005**, *7*, 3297. (d) Scalmani, G.; Frisch, M. J. Continuous Surface Charge Polarizable Continuum Models of Solvation. I. General Formalism. *J. Chem. Phys.* **2010**, *132*, 114110.

12. Gaussian 16, R. C., M. J. Frisch, G. W. Trucks, H. B. Schlegel, G. E. Scuseria, M. A. Robb, J. R. Cheeseman, G. Scalmani, V. Barone, G. A. Petersson, H. Nakatsuji, X. Li, M. Caricato, A. V. Marenich, J. Bloino, B. G. Janesko, R. Gomperts, B. Mennucci, H. P. Hratchian, J. V. Ortiz, A. F. Izmaylov, J. L. Sonnenberg, D. Williams-Young, F. Ding, F. Lipparini, F. Egidi, J. Goings, B. Peng, A. Petrone, T. Henderson, D. Ranasinghe, V. G. Zakrzewski, J. Gao, N. Rega, G. Zheng, W. Liang, M. Hada, M. Ehara, K. Toyota, R. Fukuda, J. Hasegawa, M. Ishida, T. Nakajima, Y. Honda, O. Kitao, H. Nakai, T. Vreven, K. Throssell, J. A. Montgomery, Jr., J. E. Peralta, F. Ogliaro, M. J. Bearpark, J. J. Heyd, E. N. Brothers, K. N. Kudin, V. N. Staroverov, T. A. Keith, R. Kobayashi, J. Normand, K. Raghavachari, A. P. Rendell, J. C. Burant, S. S. Iyengar, J. Tomasi, M. Cossi, J. M. Millam, M. Klene, C. Adamo, R. Cammi, J. W. Ochterski, R. L. Martin, K. Morokuma, O. Farkas, J. B. Foresman, and D. J. Fox, Gaussian, Inc., Wallingford CT.
13. (a) Schwerdtfeger, P.; Bruce, A. E.; Bruce, M. R. M. Theoretical Studies on the Photochemistry of the Cis-to-Trans Conversion in Dinuclear Gold Halide Bis(Diphenylphosphino)Ethylene Complexes. *J. Am. Chem. Soc.* **1998**, *120*, 6587–6597. (b) Pyykkö, P. Theoretical Chemistry of Gold. *Angew. Chem. Int. Ed.* **2004**, *43*, 4412–4456. (c) Seifert, T. P.; Naina, V. R.; Feuerstein, T. J.; Knöfel, N. D.; Roesky, P. W. Molecular Gold Strings: Aurophilicity, Luminescence and Structure–Property Correlations. *Nanoscale* **2020**, *12*, 20065–20088. (d) de Aquino, A.; Ward, J. S.; Rissanen, K.; Aullón, G.; Lima, J. C.; Rodríguez, L. Intra- vs Intermolecular Aurophilic Contacts in Dinuclear Gold(I) Compounds: Impact on the Population of the Triplet Excited State. *Inorg. Chem.* **2022**, *61*, 20931–20941.
14. Contreras-García, J.; Johnson, E. R.; Keinan, S.; Chaudret, R.; Piquemal, J.-P.; Beratan, D. N.; Yang, W. NCIPLOT: A Program for Plotting Noncovalent Interaction Regions. *J. Chem. Theory Comput.* **2011**, *7*, 625–632.
15. Bader, R. F. W. Atoms in Molecules. *Acc. Chem. Res.* **1985**, *18*, 9–15.
16. Lu, T.; Chen, F. Multiwfn: A Multifunctional Wavefunction Analyzer. *J. Comput. Chem.* **2012**, *33*, 580–592.
17. Boys, S. F.; Bernardi, F. The Calculation of Small Molecular Interactions by the Differences of Separate Total Energies. Some Procedures with Reduced Errors. *Mol. Phys.* **1970**, *19*, 553–566.
18. (a) Su, P.; Li, H. Energy Decomposition Analysis of Covalent Bonds and Intermolecular Interactions. *J Chem Phys* **2009**, *131*, 014102. (b) Zhao, L.; von Hopffgarten, M.; Andrada, D. M.; Frenking, G. Energy Decomposition Analysis. *WIREs Comput. Mol. Sci.* **2018**, *8*, 1–37.
19. ADF 2022.1, SCM, Theoretical Chemistry, Vrije Universiteit, Amsterdam, The Netherlands, http://www.scm.com. Contributors: E.J. Baerends, T. Ziegler, A.J. Atkins, J. Autschbach, O. Baseggio, D. Bashford, A. Bérces, F.M. Bickelhaupt, C. Bo, P.M. Boerrigter, C. Cappelli, L. Cavallo, C. Daul, D.P. Chong, D.V. Chulhai, L. Deng, R.M. Dickson, J.M. Dieterich, F. Egidi, D.E. Ellis, M. van Faassen, L. Fan, T.H. Fischer, A. Förster, C. Fonseca Guerra, M. Franchini, A. Ghysels, A. Giammona, S.J.A. van Gisbergen, A. Goez, A.W. Götz, J.A. Groeneveld, O.V. Gritsenko, M. Grüning, S. Gusarov, F.E. Harris, P. van den Hoek, Z. Hu, C.R. Jacob, H. Jacobsen, L. Jensen, L. Joubert, J.W. Kaminski, G. van Kessel, C. König, F. Kootstra, A. Kovalenko, M.V. Krykunov, P. Lafiosca, E. van Lenthe,

D.A. McCormack, M. Medves, A. Michalak, M. Mitoraj, S.M. Morton, J. Neugebauer, V.P. Nicu, L. Noodleman, V.P. Osinga, S. Patchkovskii, M. Pavanello, C.A. Peeples, P.H.T. Philipsen, D. Post, C.C. Pye, H. Ramanantoanina, P. Ramos, W. Ravenek, M. Reimann, J.I. Rodríguez, P. Ros, R. Rüger, P.R.T. Schipper, D. Schlüns, H. van Schoot, G. Schreckenbach, J.S.

20. Zheng, Q.; Borsley, S.; Nichol, G. S.; Duarte, F.; Cockroft, S. L. The Energetic Significance of Metallophilic Interactions. *Angew. Chem. Int. Ed.* **2019**, *58*, 12617–12623.
21. Brands, M. B.; Nitsch, J.; Guerra, C. F. Relevance of Orbital Interactions and Pauli Repulsion in the Metal-Metal Bond of Coinage Metals. *Inorg. Chem.* **2018**, *57*, 2603–2608.
22. (a) Blanco, M. A.; Pendás, A. M.; Francisco, E. Interacting Quantum Atoms: A Correlated Energy Decomposition Scheme Based on the Quantum Theory of Atoms in Molecules. *J. Chem. Theory Comput.* **2005**, *1*, 1096–1109. (b) Jiménez-Grávalos, F.; Díaz, N.; Francisco, E.; Martín-Pendás, Á.; Suárez, D. Interacting Quantum Atoms Approach and Electrostatic Solvation Energy: Assessing Atomic and Group Solvation Contributions. *ChemPhysChem* **2018**, *19*, 3425–3435. (c) Martín Pendás, A.; Francisco, E. Real Space Bond Orders Are Energetic Descriptors. *Phys. Chem. Chem. Phys.* **2018**, *20* (23), 16231–16237. (d) Guevara-Vela, J. M.; Hess, K.; Rocha-Rinza, T.; Martín Pendás, Á.; Flores-Álamo, M.; Moreno-Alcántar, G. Stronger-Together: The Cooperativity of Aurophilic Interactions. *Chem. Commun.* **2022**, *58*, 1398–1401.
23. Stewart, J. J. P. Optimization of Parameters for Semiempirical Methods V: Modification of NDDO Approximations and Application to 70 Elements. *J. Mol. Model.* **2007**, *13*, 1173–1213.
24. (a) Namazian, M.; Lin, C. Y.; Coote, M. L. Benchmark Calculations of Absolute Reduction Potential of Ferricinium/Ferrocene Couple in Nonaqueous Solutions. *J. Chem. Theory Comput.* **2010**, *6*, 2721–2725. (b) Psciuk, B. T.; Schlegel, H. B. Computational Prediction of One-Electron Reduction Potentials and Acid Dissociation Constants for Guanine Oxidation Intermediates and Products. *J. Phys. Chem. B* **2013**, *117*, 9518–9531.
25. (a) Bartmess, J. E. Thermodynamics of the Electron and the Proton. *J. Phys. Chem.* **1994**, *98*, 6420–6424. (b) Shankar, R.; Senthilkumar, K.; Kolandaivel, P. Calculation of Ionization Potential and Chemical Hardness: A Comparative Study of Different Methods. *Int. J. Quantum Chem.* **2009**, *109*, 764–771.
26. Marković, Z.; Tošović, J.; Milenković, D.; Marković, S. Revisiting the Solvation Enthalpies and Free Energies of the Proton and Electron in Various Solvents. *Comput. Theor. Chem.* **2016**, *1077*, 11–17.
